# Recommendations for Stress Ulcer Prophylaxis in Critically Ill Adults: A Contextualized Clinical Practice Guideline From the Saudi Critical Care Society and the Scandinavian Society of Anaesthesiology and Intensive Care Medicine, Endorsed by the Kuwait Anesthesia and Critical Care Society

**DOI:** 10.1111/aas.70201

**Published:** 2026-02-08

**Authors:** Marwa Amer, Waleed Alhazzani, Fayez Alshamsi, Anders Granholm, Yaseen M. Arabi, Klaus T. Olkkola, Marius Rehn, Abdulrahman Al‐Fares, Rakan M. Alqahtani, Ahmed Aljedai, Haifa F. Alotaibi, Amr Arafat, Shadan AlMuhaidib, Ali Al Bshabshe, Ville Jalkanen, Martin Ingi Sigurðsson, Michelle S. Chew, Maija‐Liisa Kalliomäki, Hasan M. Al‐Dorzi, Fawziah Alkhaldi, Haifa Algethamy, Majid A. Almadi, Namareq Aldardeer, Abdullah M. Alhammad, Awad Al‐Omari, Faisal A. Al‐Suwaidan, Mohammed Alshahrani, Morten Hylander Møller

**Affiliations:** ^1^ Medical/Critical Pharmacy Division King Faisal Specialist Hospital and Research Center Riyadh Saudi Arabia; ^2^ College of Medicine and Pharmacy Alfaisal University Riyadh Saudi Arabia; ^3^ Research and Innovation Institute Ministry of Defense Health Services Riyadh Saudi Arabia; ^4^ Critical Care and Internal Medicine Department, College of Medicine Imam Abdulrahman Bin Faisal University Dammam Saudi Arabia; ^5^ Department of Internal Medicine, College of Medicine and Health Sciences United Arab Emirates University Al Ain UAE; ^6^ Department of Intensive Care Copenhagen University Hospital—Rigshospitalet Copenhagen Denmark; ^7^ Section of Biostatistics, Department of Public Health University of Copenhagen Copenhagen Denmark; ^8^ Intensive Care Department, Ministry of National Guard Health Affairs, King Abdullah International Medical Research Center, King Saud Bin Abdulaziz University for Health Sciences Riyadh Saudi Arabia; ^9^ Department of Anaesthesiology, Intensive Care and Pain Medicine University of Helsinki and Helsinki University Hospital Helsinki Finland; ^10^ Division of Prehospital Services, Air Ambulance Department Oslo University Hospital Oslo Norway; ^11^ The Norwegian Air Ambulance Foundation Oslo Norway; ^12^ Institute of Clinical Medicine University of Oslo Oslo Norway; ^13^ Department of Anesthesia, Critical Care Medicine and Pain Medicine Al‐Amiri Hospital, Minister of Health Kuwait City Kuwait; ^14^ Kuwait Extracorporeal Life Support Program Al‐Amiri Center for Advance Respiratory and Cardiac Failure, Ministry of Health Kuwait City Kuwait; ^15^ Department of Critical Care Medicine, College of Medicine King Saud University Riyadh Saudi Arabia; ^16^ Adult Cardiac Surgery Department Prince Sultan Cardiac Center Riyadh Saudi Arabia; ^17^ Department of Medicine/Adult Critical Care, College of Medicine King Khalid University Abha Saudi Arabia; ^18^ Department of Intensive Care Medicine Tampere University Hospital Tampere Finland; ^19^ Faculty of Medicine University of Iceland Reykjavík Iceland; ^20^ Division of Anaesthesia and Intensive Care Medicine Landspitali—The National University Hospital of Iceland Reykjavík Iceland; ^21^ Department of Perioperative Medicine and Intensive Care Karolinska University Hospital Stockholm Sweden; ^22^ Department of Anaesthesia Tampere University Hospital Tampere Finland; ^23^ Department of Intensive Care Nursing King Faisal Specialist Hospital and Research Centre Riyadh Saudi Arabia; ^24^ Department of Anesthesia and Critical Care King Abdulaziz University Jeddah Saudi Arabia; ^25^ Division of Gastroenterology, Department of Medicine, College of Medicine, King Khalid University Hospital, King Saud University Medical City King Saud University Riyadh Saudi Arabia; ^26^ Division of Gastroenterology, The McGill University Health Center, Montreal General Hospital McGill University Montreal Canada; ^27^ Medical and Clinical Affairs Department King Faisal Specialist Hospital and Research Center‐ Jeddah Saudi Arabia; ^28^ Department of Clinical Pharmacy, College of Pharmacy King Saud University Riyadh Saudi Arabia; ^29^ Almana Medical Group Al Khobar Saudi Arabia; ^30^ Neurology Clinical Lead Ministry of Health Riyadh Saudi Arabia; ^31^ Division of Neurology, Security Forces Hospitals Program General Directorate of Medical Services, Ministry of Interior Riyadh Saudi Arabia; ^32^ College of Medicine Princess Nourah Bint Abdulrahman University Riyadh Saudi Arabia; ^33^ College of Medicine Dar Al‐Uloom University Riyadh Saudi Arabia; ^34^ Department of Emergency and Critical Care, King Fahd Hospital of the University Imam Abdulrahman Bin Faisal University Dammam Saudi Arabia; ^35^ Department of Clinical Medicine University of Copenhagen Copenhagen Denmark

**Keywords:** gastrointestinal bleeding, GRADE, guideline adaptation, intensive care, stress ulcer prophylaxis

## Abstract

**Background:**

Critically ill adults are at risk for stress‐related upper gastrointestinal bleeding (UGIB). Regional variations in gastrointestinal bleeding incidence, infection epidemiology, formulary access, and the relative value placed on bleeding versus infection outcomes by patients and clinicians necessitate contextualized recommendations. This guideline provides regionally adapted, evidence‐based recommendations for the use of stress ulcer prophylaxis (SUP) in Saudi Arabia, Kuwait, and the Nordic countries using the GRADE‐ADOLOPMENT methodology.

**Methods:**

A multidisciplinary panel from both regions prioritized PICO questions and ranked outcomes by patient importance. The 2024 Society of Critical Care Medicine (SCCM) and American Society of Health‐System Pharmacists (ASHP) guideline served as the source guideline and was evaluated for credibility; contextual fit in Saudi Arabia, Kuwait, and the Nordic countries; and alignment with the GRADE methodology. Evidence profiles and Evidence‐to‐Decision frameworks were adapted or developed, incorporating updated data, local epidemiology, drug access, and health system variables such as equity, cost, and feasibility.

**Results:**

The panel adopted the following five recommendations. In critically ill adults with coagulopathy, shock, or chronic liver disease, the panel suggests using SUP over no SUP (conditional recommendation; moderate certainty). In enterally fed patients at high risk of UGIB, the panel suggests using SUP over no SUP (conditional recommendation; very low certainty); in those at low risk, the panel suggests not using SUP (conditional recommendation; very low certainty). For patients receiving SUP, the panel suggests using a proton pump inhibitor (PPI) or a histamine‐2 receptor antagonist (H2RA) rather than sucralfate (conditional recommendation; low to moderate certainty) and suggests enteral or intravenous administration based on clinical feasibility (conditional recommendation; very low to low certainty). In critically ill adults receiving SUP, low‐dose PPI or H2RA therapy should be used rather than high‐dose regimens (best practice statement; not GRADEd). Regarding SUP discontinuation, the panel suggests discontinuing SUP in critically ill adults with resolved risk factors for UGIB (conditional recommendations; very low certainty) and in critically ill adults without UGIB risk factors but receiving SUP prior to intensive care unit (ICU) admission in the absence of an active indication (conditional recommendations; very low certainty). Differences in drug availability, ICU discharge practices, and health equity considerations shaped the panel's judgments and highlighted key implementation challenges.

**Conclusion:**

This guideline offers five context‐specific, evidence‐informed recommendations for SUP in critically ill adults in Saudi Arabia, Kuwait, and the Nordic countries. While grounded in the health system realities of these regions, the recommendations may inform practice in other settings with similar ICU structures and resource contexts. The panel also identified key research priorities to address remaining evidence gaps and support future updates.

**Abbreviations**
AE: adverse events
aOR: adjusted odds ratio
CEA: cost‐effectiveness analysis
CI: confidence interval
COI: conflict of interest
EtD: evidence‐to‐decision
GRADE: grading recommendations, assessment, development, and evaluation
H2RA: histamine‐2 receptor antagonist
HR: hazard ratio
HRQOL: health‐related quality of life
ICU: intensive care unit
iMV: invasive mechanical ventilation
IV: intravenous
LOS: length of stay
MD: mean difference
MV: mechanical ventilation
OR: odds ratios
PICO: population, intervention, comparison, and outcome(s)
PPI: proton pump inhibitor
RCT: randomized controlled trial
RR: relative risk
SCCM: Society of Critical Care Medicine
SCCS: Saudi Critical Care Society
SR: systematic review
SSAI: Scandinavian Society of Anaesthesiology and Intensive Care Medicine
SUP: stress ulcer prophylaxis
UGIB: upper gastrointestinal bleeding

## 1 Introduction

Critically ill patients are at increased risk of stress‐related upper gastrointestinal bleeding (UGIB) due to physiological stress, hypoperfusion, and mucosal ischemia [1]. Pharmacologic stress ulcer prophylaxis (SUP), primarily with proton pump inhibitors (PPIs) and histamine‐2 receptor antagonists (H2RAs), aims to reduce the risk of UGIB in the intensive care unit (ICU) setting [2].

The 2024 Society of Critical Care Medicine (SCCM) and American Society of Health‐System Pharmacists (ASHP) Guideline for the Prevention of Stress‐Related Gastrointestinal Bleeding in Critically Ill Adults offers comprehensive, evidence‐based recommendations for SUP [3]. However, its global applicability may be limited due to regional variability in healthcare infrastructure, patient populations, resource availability, and formulary access. Accordingly, regional adaptation is required to ensure feasibility, equity, and uptake in real‐world practice [4].

This contextualized guideline was jointly developed for Saudi Arabia, Kuwait, and the Nordic countries, which—despite geographical distance—share several system‐level features that support rigorous guideline adaptation. These include organized critical‐care societies and a strong culture of evidence‐based practice enabling transparent implementation and monitoring [5, 6]. No SUP guideline tailored to these regions previously existed. Healthcare systems in Saudi Arabia and Kuwait operate mixed public–private–military models with variable formulary access and capacity across sectors, whereas the Nordic countries have universal, tax‐funded systems with regional variation in hospital infrastructure and drug availability. These contextual distinctions influence the feasibility, acceptability, and equity of SUP implementation and justify regional adaptation to translate global evidence into practice. As described in prior GRADE‐ADOLOPMENT work, contextualization ensures that recommendations incorporate local epidemiology, healthcare infrastructure, patient values, and cost considerations, thereby enhancing real‐world applicability [7].

We aimed to develop context‐specific, evidence‐informed recommendations for SUP in critically ill adults across Saudi Arabia, Kuwait, and the Nordic countries to facilitate rapid, pragmatic, and regionally aligned implementation by integrating local epidemiology, healthcare systems, and resource constraints while applying the GRADE methodology.

## 2 Methods

### 2.1 Organization, Panel Composition, and Stakeholder Representation

The 2024 SCCM and ASHP Guideline for the Prevention of Stress‐Related Gastrointestinal Bleeding in Critically Ill Adults served as the source guideline for this contextualized adaptation [3]. The SCCM guideline, developed using the GRADE approach, addressed 13 PICO questions and provided accompanying evidence profiles and Evidence‐to‐Decision (EtD) frameworks. Our guideline was jointly developed by the Saudi Critical Care Society (SCCS) and the Scandinavian Society of Anaesthesiology and Intensive Care Medicine (SSAI). No commercial funding was received. All procedures adhered to established frameworks, including the AGREE II (Appraisal of Guidelines for Research and Evaluation, which assesses methodological rigor and transparency of guideline development), the Guidelines International Network McMaster checklist, and the RIGHT‐Ad@pt reporting standards (Supplemental Content 1) [8, 9]. The SCCS Guideline Chapter initiated the topic proposal and engaged with SSAI via teleconference to assess its regional relevance. Concurrently, SCCS leadership (MA and WA) engaged the Kuwait Anesthesia and Critical Care Society (KACCS), which formally endorsed the guideline after a structured review by its Critical Care Committee, given the similarities between Saudi and Kuwaiti populations and healthcare systems. A KACCS representative (AAF) actively participated in panel discussions and evidence table review. The guideline coordination group included Steering Committee members (MA, WA, and MHM) and two methodologists (MA and FA). The lead methodologist and chair (MA) oversaw the process, managed communications, supervised the adaptation workflows, coordinated panel meetings, and ensured adherence to GRADE‐ADOLOPMENT standards. The guideline involved a multidisciplinary panel composed of 28 stakeholders, including clinicians from critical care medicine, anesthesiology, gastroenterology, surgery, internal medicine, and emergency medicine, along with methodologists, pharmacists, and policy experts, ensuring balance in expertise, geography, and sector representation. As recommended in prior GRADE‐ADOLOPMENT applications, panel selection considered contextual familiarity, stakeholder relevance, and methodological diversity (Supplemental Content 2: 2.1–2.2).

### 2.2 Scope and Target Population

This guideline addresses SUP in critically ill adult patients admitted to ICUs across Saudi Arabia, Kuwait, and the Nordic countries. It includes medical, surgical, and neurocritical care subpopulations at increased UGIB risk and excludes pediatric and non‐ICU populations.

### 2.3 Target Users

The intended users are intensivists, anesthesiologists, pharmacists, ICU nurses, advanced practice providers, healthcare administrators, and policy‐makers involved in ICU planning and resource allocation. It is aligned with Saudi Arabia's Vision 2030 and Nordic intensive care quality‐improvement initiatives.

### 2.4 Conflict of Interest Management

All panelists completed an electronic conflict of interest (COI) form before the appointment. The Steering Committee reviewed and managed COIs throughout. Three panelists declared academic COIs, which were deemed secondary and managed per SCCS and SSAI policies [10].

### 2.5 Prioritization of PICO Questions

Following a structured prioritization process, we focused on five key PICO questions to strategically address the most critical evidence gaps (Table 1). The final set reflected consensus around the highest‐yield areas for contextualized guidance, including identification of high‐risk populations [11], SUP use stratified by risk and enteral feeding status, pharmacologic agent selection, optimal administration strategies, and SUP deprescribing criteria. All questions were formulated using the PICO (Population, Intervention, Comparator, Outcome) framework consistent with GRADE standards [12]. The “Time” and “Setting/Study design” elements occasionally included in extended PICOST formulations were not specified, as these contextual factors were systematically considered during evidence appraisal and within the EtD frameworks addressing resource use, equity, feasibility, and acceptability.

**TABLE 1:** Prioritized PICO questions for SUP guideline adaptation.

PICO	Population	Intervention	Comparator	Outcomes	Subgroup(s)
1. Should critically ill adults with coagulopathy, shock, or chronic liver disease receive SUP or no SUP to prevent UGIB?	Critically ill adults in ICU with coagulopathy, shock, or chronic liver disease^a^	SUP	No SUP	Clinically Important UGIB Overt UGIB Pneumonia *Clostridioides difficile* Infection Mortality	Neurocritical care patients Cardiac surgery patients
2. Should critically ill adults with UGIB risk factors who are enterally fed during ICU admission receive SUP or no SUP to prevent UGIB?	Critically ill adults with risk factors for UGIB^a^ who are enterally fed during ICU admission	SUP	No SUP	Clinically Important UGIB Overt UGIB Pneumonia *Clostridioides difficile* infection Mortality	Enterally fed patients at low risk of UGIB
3. In critically ill adults with risk factors for stress‐related UGIB, what are the comparative effectiveness and harms of SUP agents (PPIs, H2RAs, or sucralfate) when used at low doses versus high doses, or administered via enteral versus intravenous routes?	Critically ill adults in ICU with UGIB risk factors	Comparative effectiveness and safety of SUP agents (PPIs, H2RAs, or sucralfate)		Clinically Important UGIB Overt UGIB Pneumonia *Clostridioides difficile* infection Mortality	– Route of administration (enteral vs. IV) – Low‐dose vs. high‐dose SUP
4. Should critically ill adults whose UGIB risk factors are no longer present continue or discontinue SUP?	Critically ill adults whose UGIB risk factors are no longer present	Continued SUP	Discontinued SUP	Clinically Important UGIB Overt UGIB Pneumonia *Clostridioides difficile* infection Mortality	—
5. Should critically ill adults without current UGIB risk factors but receiving SUP before ICU admission continue or discontinue SUP?	Critically ill adults with resolved UGIB risk factors but receiving SUP before ICU admission	Continued SUP	Discontinued SUP	Clinically Important UGIB Overt UGIB Pneumonia *Clostridioides difficile* infection Mortality	—

Abbreviations: H2RA, histamine‐2 receptor antagonist; ICU, intensive care unit; IV, intravenous; PICO, population, intervention, comparator, and outcome(s); PPI, proton pump inhibitor; SUP, stress ulcer prophylaxis; UGIB, upper gastrointestinal bleeding.

^a^ Definitions of key risk factors were not consistent across trials and are provided here for interpretative guidance (SCCM–ASHP 2024; BMJ 2020 Rapid Recommendation). Coagulopathy = one or more of the following: platelet count < 50 × 10^9^/L, INR > 1.5, or prothrombin time > 20 s. Shock = one or more of the following: continuous vasopressor or inotrope infusion, SBP < 90 mmHg, MAP < 70 mmHg, or lactate ≥ 4 mmol/L. Chronic liver disease = one or more of portal hypertension; cirrhosis proven by biopsy or imaging; history of variceal bleeding or hepatic encephalopathy. Exact cut‐offs vary among studies; clinicians should apply local clinical judgment and laboratory standards.

### 2.6 Outcome Prioritization

Using structured surveys and discussions, panelists prioritized outcomes based on clinical relevance, resource implications, and patient impact. The five critical outcomes were clinically important UGIB, overt UGIB, pneumonia/ventilator‐associated pneumonia (VAP), *Clostridioides difficile* ( *C. difficile* ) infection, and mortality. Important outcomes included ICU and hospital length of stay, delirium, renal failure, diarrhea, and any UGIB. Biomarkers, such as gastric pH, were considered unimportant for decision‐making [13] (Supplemental Contents 2: 2.3 and 3. Outcomes and PICO Prioritization).

### 2.7 Evidence Assessment and Updating

#### 2.7.1 Evaluation of Existing Evidence Profiles (EPs)

Systematic reviews used in the source guideline were assessed for credibility using the AMSTAR 2 tool [14]. If deemed high quality and current, the evidence was adopted unchanged; otherwise, updates were conducted using Cochrane‐compliant methods [15].

#### 2.7.2 Identification of New Evidence

When source reviews lacked relevant outcomes or were outdated, targeted literature searches were performed. The methodologist and panel searched Ovid MEDLINE, Embase, and Cochrane Library, covering studies through July 27, 2025. Keywords included “stress ulcer prophylaxis,” “critically ill patients,” “PPIs,” “H2RAs,” and “enteral nutrition.” Included studies were randomized controlled trials (RCTs), cohort studies, and systematic reviews/meta‐analyses reporting relevant outcomes. New evidence was mapped to the five PICO questions, with updated profiles created as needed.

#### 2.7.3 Supplementary Evidence Sources

Panelists were encouraged to suggest newly published studies or context‐specific factors (e.g., medication availability, local ICU infrastructure, equity), which were integrated into EtD frameworks.

### 2.8 Statistical Analysis

When meta‐analysis was performed, RevMan software (version 5.3, Nordic Cochrane Centre, Copenhagen) was used to generate pooled estimates [16, 17]. The DerSimonian and Laird random‐effects model and inverse‐variance method were applied. For smaller datasets (< 5 trials), fixed‐ and random‐effects models were compared, with the more conservative estimate reported. Pooled estimates were expressed as relative risks (RRs) or odds ratios (ORs) with 95% confidence intervals (CIs) for dichotomous outcomes and mean differences (MDs) with 95% CIs for continuous outcomes [18]. Heterogeneity was assessed using the *Χ* ^2^ (*p* < 0.05), the I^2^ statistic (> 50%), and forest plots inspection. The evidence was narratively summarized for questions with insufficient quantitative data. For > 10 trials, funnel plots and Egger's test were used to assess publication bias. The longest follow‐up (hospital or ICU mortality) was used for mortality data.

### 2.9 Certainty of Evidence and Grading of Recommendations

The GRADE approach was used to assess certainty of evidence, rated as high, moderate, low, or very low based on risk of bias, imprecision, indirectness, inconsistency, and publication bias. The Guideline Development Tool (Evidence Prime, Hamilton, Ontario, Canada) was used to generate evidence profiles and summaries [19, 20, 21] (Supplemental Content 2: 2.4).

### 2.10 Recommendation Formulation and Voting Process

Recommendations were developed using the EtD frameworks, incorporating the balance of benefits and harms, certainty of evidence, patient values, costs, equity, feasibility, and acceptability (Supplemental Content 2: 2.4) [21]. Local data were included where available (e.g., drug costs from the Saudi Food and Drug Authority and the National Unified Procurement Company, Nordic formulary data, and healthcare access disparities) [22, 23]. The methodologist drafted preliminary recommendations, which were reviewed and discussed by the panel. Voting was conducted using Panel Voice (Evidence Prime, Hamilton, Ontario, Canada). In case of disagreement, the majority vote prevailed, and results were recorded. Panelists absent due to time zone differences were required to review recordings and provide input. Each recommendation was classified as adopted, adapted, or *de novo* based on the extent of contextual differences requiring modification from the original source recommendation [4]. A recommendation required ≥ 80% panel approval. Up to three voting rounds were permitted if consensus was not achieved. A strong recommendation was made according to GRADE if the panel was “confident that the desirable effects outweighed the undesirable effects” [20, 21]. A conditional recommendation was made if the panel concluded that the “desirable effects probably outweigh the undesirable effects,” but the trade‐offs were not well defined, and the recommendation may not be applicable to all patients [20, 21]. The terms “recommend” and “suggest” were used to reflect strong and conditional recommendations, respectively [24]. Best practice statements are ungraded recommendations reflecting the collective clinical experience and consensus of the expert panel. The application of such statements must be guided by the clinician's judgment and tailored to the individual patient's clinical context, treatment options, and available resources. Additional information on the GRADE approach and the implications of different recommendations for key stakeholders is presented in Supplemental Content 2: 2.5–2.6.

## 3 Results

**FIGURE 1:**
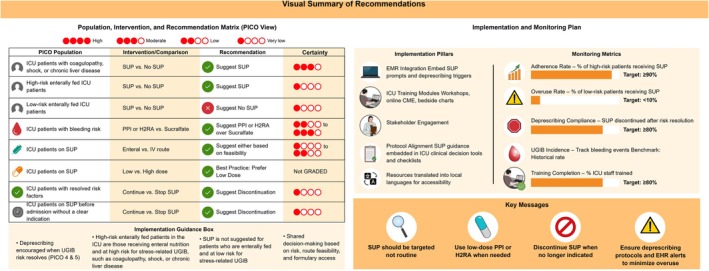
Visual summary of recommendations, implementation, and monitoring plan. Recommendations were formulated using the GRADE‐ADOLOPMENT methodology, incorporating evidence profiles, regional practice patterns, drug availability, and health system considerations. Certainty of evidence is rated as High (⊕⊕⊕⊕), Moderate (⊕⊕⊕◯), Low (⊕⊕◯◯), or Very low (⊕◯◯◯). The terms “suggest” and “recommend” reflect conditional and strong recommendations, respectively. For full EtD frameworks and evidence profiles, refer to Supplemental Content 2. Readers are directed to PICO 1 (Results section, Table 1 footnote, and EtD framework—Supplemental Content 2: 2.13) for detailed definitions of stress‐related UGIB risk factors. Summary of the implementation and monitoring plan for contextualized SUP guideline uptake is based on WHO's GRADE‐ADOLOPMENT framework. Key strategies include EHR integration, professional education, stakeholder endorsement, and dissemination of resources translated into local languages (e.g., Arabic, Danish, Swedish, Norwegian, Finnish, and Icelandic). Monitoring indicators and performance targets were informed by WHO recommendations and ICU quality standards: SUP adherence ≥ 90% reflects international benchmarks for evidence‐based ICU interventions; overuse < 10% aligns with safety and stewardship goals; deprescribing compliance and training coverage ≥ 80% are realistic targets promoting safe practice and system‐level uptake. UGIB incidence is monitored against institutional baselines to ensure no unintended harm. EHR, electronic health record; H_2_RA, histamine‐2 receptor antagonist; ICU, intensive care unit; PPI, proton pump inhibitor; SUP, stress ulcer prophylaxis; UGIB, upper gastrointestinal bleeding.

Table 2 and Figure 1 present a summary of the recommendations. The full GRADE evidence profiles and EtD frameworks are available in Supplemental Content 2. Across all PICOs, the panel's judgments reflected contextual adaptation to regional health‐system realities, including variation in formulary access, infection epidemiology, and ICU discharge practices. The extent of adaptation differed by question: while PICOs 1–3 primarily involved adoption or minor modification of the SCCM recommendations, PICOs 4 and 5 required *de novo* formulation to address local gaps in deprescribing and continuity‐of‐care practices.

**TABLE 2:** Summary of recommendations.

Recommendation	Strength and certainty of evidence	Practical considerations	GRADE evidence profile and EtD framework
Should critically ill adults with coagulopathy, shock, or chronic liver disease receive SUP or no SUP to prevent UGIB?			
Recommendation 1: In critically ill adults with coagulopathy, shock, or chronic liver disease, the panel suggests SUP over no SUP	(conditional recommendation, moderate certainty in evidence ⊕⊕⊕◯).^a^	In patients with coagulopathy, shock, and chronic CIGIB, risk is elevated, with benefits increasing as baseline bleeding risk rises. Clinicians may consider mechanically ventilated patients as an “at‐risk” population for stress‐related UGIB, warranting individualized assessment for SUP initiation. Clinicians and patients may opt not to use SUP in low‐risk populations (with a 1%–2% risk of clinically important GI bleeding) Some evidence suggests that PPIs may increase mortality in high illness acuity. Given these considerations, a risk‐stratified approach is essential. SUP should be reserved for those at the highest risk of GI bleeding, while deprescribing should be considered in low‐risk patients to avoid unnecessary exposure and resource waste. Cost‐effectiveness analysis (E‐REVISE) demonstrated that SUP in invasively mechanically ventilated adults is clinically beneficial (reduction in CIGIB) and economically favorable (shorter ICU/ward length of stay and overall cost savings) compared with no SUP.	https://guidelines.gradepro.org/profile/ah6MhhOknC0 NMA link https://gdt.gradepro.org/presentations/#/nma/nma_question_1f57d3a1‐f4e6‐43a3‐9f1e‐f7900d9566e3
Should critically ill adults with UGIB risk factors who are enterally fed receive SUP or no SUP to prevent UGIB?			
Recommendation 2: In critically ill adults who are enterally fed and at high risk of UGIB, the panel suggests using SUP over no SUP In critically ill adults who are enterally fed and at low risk of UGIB, the panel suggests not using SUP	(Conditional recommendation, very low certainty in evidence ⊕◯◯◯).^a^	The phrase “low or no risk factors” is used to reflect a category of patients at minimal baseline risk. For the definition of high‐risk patients, please refer to PICO 1, which includes criteria such as coagulopathy, shock, and chronic liver disease.	https://guidelines.gradepro.org/profile/c9yNAJo7aho
In critically ill adults with risk factors for stress‐related UGIB, what are the comparative effectiveness and harms of SUP agents (PPIs, H2RAs, or sucralfate) when used at low doses versus high doses or administered via enteral versus intravenous routes?^c^			
Recommendation 3.1: Agent selection In critically ill adults with UGIB risk factors, the panel suggests using either a PPI or an H2RA over sucralfate for SUP	(Conditional recommendation, low to moderate certainty in evidence ⊕⊕◯◯/⊕⊕⊕◯).^a^	The choice between PPI and H2RA may be guided by individual bleeding risk, illness severity, and local formulary availability. PPIs may offer greater benefit in higher‐risk patients, whereas H2RAs may be preferred in those with high illness acuity due to potential safety concerns with PPIs.	https://guidelines.gradepro.org/profile/Q9kbbmZzazo NMA link for the agents: https://gdt.gradepro.org/presentations/#/nma/nma_question_1f57d3a1‐f4e6‐43a3‐9f1e‐f7900d9566e3
Recommendation 3.2: Route of administration In critically ill adults receiving SUP, the panel suggests either the enteral or intravenous administration, depending on clinical context and feasibility	(conditional recommendation, very low to low certainty in evidence ⊕◯◯◯/⊕⊕◯◯)^a^	Evidence does not favor one route over the other. When feasible, the enteral route may reduce costs and simplify administration. Caution is advised in interpretation due to potential confounding by illness severity in available studies.	https://guidelines.gradepro.org/profile/Q9kbbmZzazo NMA link for the route of administration: https://gdt.gradepro.org/presentations/#/nma/nma_question_8d3fd775‐9e1b‐4580‐8660‐da9bf29d1f04
Recommendation 3.3: Dosing strategy In critically ill adults receiving stress ulcer prophylaxis, low‐dose PPI or H2RA therapy rather than high‐dose regimens should be used	(Best Practice Statement—not GRADEd).^a^	Higher doses have not shown a meaningful added benefit but may increase the cost and risk of adverse effects. Clinical judgment remains essential, particularly in patients who are NPO or exhibit altered pharmacokinetics	https://guidelines.gradepro.org/profile/Q9kbbmZzazo NMA link for the dose: https://gdt.gradepro.org/presentations/#/nma/nma_question_0bd7e2d0‐9cbf‐4cf1‐ada2‐ebf3d232498d
Should critically ill adults whose UGIB risk factors are no longer present continue or discontinue SUP?^c^			
Recommendation 4: In critically ill adults with resolved risk factors for UGIB, the panel suggests discontinuing SUP	(conditional recommendation, very low certainty in evidence ⊕◯◯◯).^b^	—	https://guidelines.gradepro.org/profile/yH7eqm4ECNQ
Should critically ill adults without current UGIB risk factors but receiving SUP before ICU admission continue or discontinue SUP?^c^			
Recommendation 5: In critically ill adults without UGIB risk factors but receiving SUP prior to ICU admission, the panel suggests discontinuing SUP in the absence of an active indication	(conditional recommendation, very low certainty in evidence ⊕◯◯◯).^b^	For patients already on PPIs or H2 blockers prior to ICU admission, the indication for continuation should be explicitly reviewed. If no longer needed, discontinuation is appropriate. Common valid indications may include recent UGIB, erosive esophagitis, active *Helicobacter pylori* therapy, or Zollinger‐Ellison syndrome. In cases where the indication is unclear (e.g., patients with limited histories or altered mental status), clinicians should defer discontinuation until outpatient history is clarified or consult the prior prescribing physician. These considerations are especially relevant during ICU transitions, where intensivists may not have complete access to long‐term GI histories.	https://guidelines.gradepro.org/profile/yH7eqm4ECNQ

Abbreviations: CIGIB, clinically important gastrointestinal bleeding; EtD, evidence‐to‐decision; GRADE, grading of recommendations, assessment, development, and evaluations; H2RA, histamine‐2 receptor antagonist; ICU, intensive care unit; NMA, network meta‐analysis; NPO, Nil per os (nothing by mouth); PICO, population; intervention; comparator; and outcome(s); PPI, proton pump inhibitor; SUP, stress ulcer prophylaxis; UGIB, upper gastrointestinal bleeding.

^a^ There are substantial changes in the evidence‐to‐decision (EtD) framework and evidence profiles of the source guideline. We updated it with current evidence syntheses and contextual data to support panel judgments and formulation of an adapted recommendation. The final direction of the adapted recommendation and the certainty of evidence were aligned with the source guideline recommendation.

^b^ EtD frameworks for PICOs 4 and 5 were developed *de novo*, independent of the source guidelines, to ensure contextual relevance and methodological rigor. The source guideline panel issued a Best Practice Statement, whereas our panel issued a formal conditional recommendation in the same direction. Consequently, the final recommendation certainty of evidence was modified to a “*de novo* recommendation”.

^c^ For PICOs 3–5, the EtD module's multi‐comparison function was used, enabling direct, simultaneous assessment of multiple pharmacologic interventions (PPIs, H2RAs, and sucralfate) against a common comparator.

### 3.1 PICO 1: Should Critically Ill Adults With Coagulopathy, Shock, or Chronic Liver Disease Receive SUP or no SUP to Prevent UGIB?

#### 3.1.1 Recommendation 1

In critically ill adults with coagulopathy, shock, or chronic liver disease, the panel suggests SUP over no SUP (conditional recommendation, moderate certainty of evidence).

Descriptions of coagulopathy, shock, and chronic liver disease are provided in Table 1 and detailed in the EtD framework (Supplement Content 2: 2.13).

#### 3.1.2 Rationale

We evaluated the network meta‐analysis (NMA) from the source guideline, supplemented by two PROSPERO‐registered NMAs (Wang et al., CRD42019126656 and CRD42020169989) [25, 26]. We also included PROSPERO‐registered pairwise systematic reviews and meta‐analyses (SRMAs CRD42023461695) to integrate newly published data, most notably from the REVISE trial, which was released after the source guideline's search cutoff [27]. Baseline risks for clinically important gastrointestinal bleeding (CIGIB) and overt GI bleeding were estimated using the same approach applied in prior guidelines [11], incorporating data from SUP‐ICU [28], REVISE [29], and supporting observational studies and meta‐analyses [30, 31, 32, 33] (Supplemental Content 2: 2.7–2.9).

PPIs likely reduce the risk of CIGIB. In patients at moderate, high, and highest baseline risk, PPIs are associated with absolute risk reductions of 1.2%, 2.3%, and 3.5%, respectively. This corresponds to a moderate effect at high risk and a large effect at the highest risk level (RR, 0.61; 95% CI, 0.42–0.89; moderate certainty of evidence) [25, 26, 27, 28, 29]. In a more recent SRMA including REVISE trial, PPIs reduce CIGIB (RR, 0.51; 95% CI, 0.34–0.76; high certainty of evidence) and yield absolute risk reductions of 1.7% (moderate risk), 2.9% (high risk), and 4.4% (highest risk), indicating a small‐to‐large effect depending on baseline risk [25, 26, 27, 28, 29]. PPIs also reduce overt GI bleeding (RR, 0.52; 95% CI, 0.38–0.70; high certainty of evidence) and yield absolute risk reductions of 2.6%, 6.0%, and 9.1% across moderate‐, high‐, and highest‐risk groups, indicating a moderate‐to‐large effect depending on the population [25, 26, 27, 28, 29].

H2RAs may also reduce clinically important and overt GI bleeding, but the evidence is of moderate certainty and the magnitude of effect is less certain.

PPIs likely result in little to no difference in duration of mechanical ventilation or ICU length of stay (low certainty of evidence).

PPIs likely result in little to no difference in mortality compared to no prophylaxis (RR, 0.99; 95% CI, 0.93–1.05; low certainty of evidence). However, subgroup analyses from the SUP‐ICU and REVISE trials suggest that the effect on mortality may differ by illness severity, with possible harm in patients with high severity and possible benefit in those with lower acuity [28, 29].

Similarly, PPIs likely result in little to no difference in the risk of pneumonia (RR, 1.00; 95% CI, 0.92–1.09; low certainty of evidence) and little to no difference in the risk of *C. difficile* infection (RR, 1.20; 95% CI, 0.66–2.16; low certainty of evidence) [25, 26, 27, 28, 29].

Other adverse events, such as hypomagnesemia and renal dysfunction, were not systematically reported across trials. Overall certainty of the evidence was rated as moderate and downgraded for imprecision, serious inconsistency, risk of bias, and indirectness for certain outcomes (e.g., *C. difficile* infection).

Taken together, the evidence suggests that while SUP confers moderate to large reductions in bleeding outcomes for high‐risk patients, potential harms are uncertain, and the balance of effects varies depending on individual bleeding risk (i.e., a risk‐stratified approach). The panel judged that the overall balance of benefits and harms probably favors the use of SUP in critically ill patients with coagulopathy, shock, or chronic liver disease, particularly those at moderate or high baseline risk of gastrointestinal bleeding. While SUP does not appear to reduce mortality, shorten ICU length of stay, or affect long‐term outcomes, its ability to prevent CIGIB and overt GI bleeding in high‐risk patients was considered a critical benefit.

From the values and preferences perspective, variability was identified in how patients might prioritize bleeding prevention over potential adverse effects. In a post hoc analysis of the SUP‐ICU trial, pantoprazole reduced GI bleeding but had no observed effect on long‐term outcomes, including employment status or healthcare costs [34]. These findings suggest that some patients may value immediate prevention of bleeding, while others may not accept the trade‐off in the absence of long‐term benefit. The panel noted that patients would typically require an absolute reduction in CIGIB by approximately 15–20 events per 1000 patients to justify prophylaxis. These estimates were informed by available regional studies and validated by panel input, reflecting contextual knowledge of ICU populations in Saudi Arabia, Kuwait, and the Nordic countries. For resource use, the acquisition cost of SUP medications—PPIs or H2RAs—is minimal relative to overall ICU expenses. However, evidence from the SUP‐ICU cost analysis suggests that pantoprazole use did not reduce total healthcare expenditures, resource utilization, or hospitalization duration. The cost‐effectiveness of SUP is therefore variable, depending on patient baseline risk, the agent used, and post‐ICU continuation patterns. SUP was considered widely available and generally acceptable to clinicians. However, concerns were raised about equity implications in lower‐resource settings, where SUP costs—even if modest—may influence care decisions. Panelists emphasized the importance of deprescribing practices and EMR‐based decision support to enable feasible and equitable implementation of risk‐based SUP strategies and reduce unnecessary use. Given these considerations, the panel suggests using SUP over no prophylaxis. Information for Evidence Profile, EtD Framework, and SUP in ICU subpopulations is available in Table 3 and Supplemental Content 2: 2.10–2.14 [35, 36, 37, 38, 39]. A key conclusion of the panel is that SUP should not be applied routinely to all patients in the ICU but rather tailored to patients with clearly defined risk factors. Clinicians should weigh individual bleeding risk against uncertain harms and consider deprescribing SUP in low‐risk individuals. Additionally, ICU protocols should include decision support to guide appropriate initiation, reassessment, and discontinuation of SUP.

**TABLE 3:** Summary of findings table.

Outcome no. of participants (studies)	Relative effect (95% CI)	Anticipated absolute effects (95% CI)			Certainty	Comment and interpretation
		No SUP	SUP	Difference		
PICO 1: Should critically ill adults with coagulopathy, shock, or chronic liver disease receive SUP or no SUP to prevent UGIB?						
Clinically important GI bleeding: Proton pump inhibitor vs. No prophylaxis NMA of RCTs No. of participants: 4317 (8 RCTs)	**RR 0.61** (0.42 to 0.89)^a^, ^b^	**Moderate**			⊕⊕⊕◯ Moderate^d^	**Moderate‐risk patients:** PPIs likely reduce GI bleeding slightly (ARR 1.2%). **High‐risk patients:** PPIs likely reduce GI bleeding (ARR 2.3%). **Highest‐risk patients:** PPIs likely result in a moderate to large reduction in GI bleeding (ARR 3.5%). **Low‐risk patients** (ARR ~0.5%, not shown but implied): PPIs likely result in little to no difference in GI bleeding.
		3.0%	**1.8%** (1.3 to 2.7)	**1.2% fewer** (1.7 fewer to 0.3 fewer)		
		**High**				
		6.0%^c^	**3.7%** (2.5 to 5.3)	**2.3% fewer** (3.5 fewer to 0.7 fewer)		
		**Highest**				
		9.0%^c^	**5.5%** (3.8 to 8)	**3.5% fewer** (5.2 fewer to 1 fewer)		
Clinically important GI bleeding: Proton pump inhibitor vs. No prophylaxis Pairwise SRMA NEJM 2024 (including REVISE) No. of participants: 9079 (9 RCTs)	**RR 0.51** (0.34 to 0.76)	**Moderate**			⊕⊕⊕⊕ High	**Moderate‐risk patients**: PPIs reduce GI bleeding slightly (ARR 1.7%). **High‐risk patients**: PPIs reduce GI bleeding (ARR 2.9%). **Highest‐risk patients**: PPIs result in a moderate to large reduction in GI bleeding (ARR 4.4%).
		3.5%	**1.8%** (1.2 to 2.7)	**1.7% fewer** (2.3 fewer to 0.8 fewer)		
		**High**				
		6.0%	**3.1%** (2 to 4.6)	**2.9% fewer** (4 fewer to 1.4 fewer)		
		**Highest**				
		9.0%	**4.6%** (3.1 to 6.8)	**4.4% fewer** (5.9 fewer to 2.2 fewer)		
Clinically important GI bleeding: Histamine‐2 receptor antagonist vs. No prophylaxis NMA No. of participants: 1242 (14 RCTs)	**OR 0.46** (0.27 to 0.79)^e^, ^f^	**Moderate**			⊕⊕⊕◯ Moderate^d^	**Moderate‐risk patients**: H2RAs likely reduce GI bleeding slightly (ARR 1.6%). **High‐risk patients**: H2RAs likely reduce GI bleeding (ARR 3.1%). **Highest‐risk patients**: H2RAs result in a large reduction in GI bleeding (ARR 4.6%).
		3.0%	**1.4%** (0.8 to 2.4)	**1.6% fewer** (2.2 fewer to 0.6 fewer)		
		**High**				
		6.0%	**2.9%** (1.7 to 4.8)	**3.1% fewer** (4.3 fewer to 1.2 fewer)		
		**Highest**				
		9.0%	**4.4%** (2.6 to 7.2)	**4.6% fewer** (6.4 fewer to 1.8 fewer)		
Overt GI bleeding: Proton pump inhibitor vs. No prophylaxis NMA No. of participants: 3867 (5 RCTs)	**OR 0.59** (0.45 to 0.76)^g^, ^h^	**Moderate**			⊕⊕⊕⊕ High	**Moderate‐risk patients**: PPIs reduce overt GI bleeding (ARR 2.9%). **High‐risk patients**: PPIs reduce overt GI bleeding (ARR 4.7%). **Highest‐risk patients**: PPIs result in a large reduction in overt GI bleeding (ARR 6.8%).
		7.5%	**4.6%** (3.5 to 5.8)	**2.9% fewer** (4 fewer to 1.7 fewer)		
		**High**				
		12.5%	**7.8%** (6 to 9.8)	**4.7% fewer** (6.5 fewer to 2.7 fewer)		
		**Highest**				
		19.0%	**12.2%** (9.5 to 15.1)	**6.8% fewer** (9.5 fewer to 3.9 fewer)		
Overt GI bleeding: Proton pump inhibitor vs. No prophylaxis Pairwise SRMA NEJM 2024 (including REVISE) No. of participants: 9391 (11 RCTs)	**RR 0.52** (0.38 to 0.70)	**Moderate**			⊕⊕⊕⊕ High	**Moderate‐risk patients**: PPIs reduce overt GI bleeding (ARR 2.6%). **High‐risk patients**: PPIs reduce overt GI bleeding (ARR 6.0%). **Highest‐risk patients**: PPIs result in a large reduction in overt GI bleeding (ARR 9.1%).
		5.4%	**2.8%** (2 to 3.8)	**2.6% fewer** (3.3 fewer to 1.6 fewer)		
		**High**				
		12.5%	**6.5%** (4.8 to 8.8)	**6.0% fewer** (7.8 fewer to 3.8 fewer)		
		**Highest**				
		19.0%	**9.9%** (7.2 to 13.3)	**9.1% fewer** (11.8 fewer to 5.7 fewer)		
Overt GI bleeding: Histamine‐2 receptor antagonist vs. No prophylaxis NMA No. of participants: 2428 (29 RCTs)	**OR 0.38** (0.24 to 0.59)^h^, ^i^	**Moderate**			⊕⊕⊕◯ Moderate^j^	**Moderate‐risk patients**: H2RAs likely reduce overt GI bleeding (ARR 4.5%). **High‐risk patients**: H2RAs likely reduce overt GI bleeding (ARR 7.4%). **Highest‐risk patients**: H2RAs likely result in a large reduction in overt GI bleeding (ARR 10.8%).
		7.5%	**3.0%** (1.9 to 4.6)	**4.5% fewer** (5.6 fewer to 2.9 fewer)		
		**High**				
		12.5%	**5.1%** (3.3 to 7.8)	**7.4% fewer** (9.2 fewer to 4.7 fewer)		
		**Highest**				
		19.0%	**8.2%** (5.3 to 12.2)	**10.8% fewer** (13.7 fewer to 6.8 fewer)		
Mortality: Proton pump inhibitor vs. No prophylaxis NMA No. of participants: 4194 (9 RCTs)	**OR 1.06** (0.90 to 1.28)^k^	**Risk per 1000**			⊕⊕⊕◯ Moderate^l^	PPIs likely result in little to no difference in mortality
		30.4%	**31.6%** (28.2 to 35.9)	**1.2% more** (2.2 fewer to 5.5 more)		
Mortality: Proton pump inhibitor vs. No prophylaxis Pairwise SRMA NEJM 2024 (including REVISE) No. of participants: 9463 (12 RCTs)	**RR 0.99** (0.93 to 1.05)	30.9%	**30.6%** (28.7 to 32.4)	**0.3% fewer** (2.2 fewer to 1.5 more)	⊕⊕◯◯ Low^m^	PPIs may result in little to no difference in mortality
Mortality‐ Histamine‐2 receptor antagonist vs. No prophylaxis NMA No. of participants: 1835 (22 RCTs)	**OR 0.96** (0.79 to 1.19)^n^	**Risk per 1000**			⊕⊕⊕◯ Moderate^l^	H2RAs likely result in little to no difference in mortality
		30.4%	**29.5%** (25.7 to 34.2)	**0.9% fewer** (4.7 fewer to 3.8 more)		
Pneumonia: Proton pump inhibitor vs. No prophylaxis NMA No. of participants: 3974 (6 RCTs)	**OR 1.39** (0.98 to 2.10)^o^	**Risk per 1000**			⊕⊕◯◯ Low^p^, ^q^	PPIs may increase pneumonia
		16.2%	**21.2%** (15.9 to 28.9)	**5.0% more** (0.3 fewer to 12.7 more)		
Pneumonia Proton pump inhibitor vs. No prophylaxis Pairwise SRMA NEJM 2024 (including REVISE) No. of participants: 8949 (8 RCTs)	**RR 1.00** (0.92 to 1.09)	23.8%	**23.8%** (21.9 to 25.9)	**0.0% fewer** (1.9 fewer to 2.1 more)	⊕⊕◯◯ Low^r^	PPIs may result in little to no difference in pneumonia
Pneumonia: Histamine‐2 receptor antagonist vs. No prophylaxis NMA No. of participants: 1159 (11 RCTs)	**OR 1.26** (0.89 to 1.85)^s^	**Risk per 1000**			⊕⊕◯◯ Low^p^, ^t^	H2RAs may increase pneumonia
		16.2%	**19.6%** (14.7 to 26.3)	**3.4% more** (1.5 fewer to 10.1 more)		
*Clostridium difficile* infection‐Proton pump inhibitor vs. No prophylaxis Pairwise SRMA NEJM 2024 (including REVISE) No. of participants: 8682 (6 RCTs)	**RR 1.20** (0.66 to 2.16)	0.7%	**0.8%** (0.5 to 1.5)	**0.1% more** (0.2 fewer to 0.8 more)	⊕⊕◯◯ Low^u^	PPIs may result in little to no difference in CDI
*Clostridium difficile* infection‐ Histamine‐2 receptor antagonist vs. No prophylaxis NMA No. of participants: (0 non‐randomized studies)	Indirect estimate was rated down because one of the direct estimates (PPIs vs. H2RAs) in the first order loop, which contributed to the indirect estimate, was rated down for risk of bias. No direct evidence. Odds ratio 0.94 (95% CI, 0.06 to 14.99). Difference: **0 fewer per 1000** (95% CI, 14 fewer to 226 more)				⊕◯◯◯ Very low^v^, ^w^	The evidence is very uncertain regarding the effect of H2RAs on CDI

Abbreviations: ARR, absolute risk reduction; CDI, *Clostridioides difficile* infection; CI, confidence interval; CIGIB, clinically important gastrointestinal bleeding; CrI, credible interval; GRADE, grading of recommendations, assessment, development, and evaluations; H2RA, histamine‐2 receptor antagonist; ICU, intensive care unit; LOS, length of stay; MD, mean difference; MV, mechanical ventilation; NMA, network meta‐analysis; OR, odds ratio; PICO, population, intervention, comparator, and outcome(s); PPI, proton pump inhibitor; RCT, randomized controlled trial; RR, risk ratio; SRMA, systematic review and meta‐analysis; SUP, stress ulcer prophylaxis; UGIB, upper gastrointestinal bleeding.

^a^ 2020 updated SR and NMA revealed the following for patients at highest or high risk of bleeding from the complete PEPTIC analysis: RR 0.46; 95% CrI, 0.29–0.66, moderate certainty evidence, with 32 fewer per 1000 for high‐risk patients and 49 fewer per 1000 for highest‐risk patients.

^b^ SCCM NMA conducted by the guideline panel was not stratified by baseline bleeding risk, which may influence the applicability of the estimates to populations at different risk levels. For the PPI versus placebo comparison, the direct estimate from conventional MA was 0.62 (95% CrI, 0.43–0.89), while the estimate from node splitting was 0.65 (95% CrI, 0.37–1.30), both with moderate certainty. The indirect estimate showed a lower effect size at 0.30 (95% CrI, 0.12–0.68), also rated as moderate certainty, and the NMA estimate was 0.52 (95% CrI, 0.30–0.81), with moderate certainty.

^c^ We grouped patients into categories according to risk of clinically important gastrointestinal bleeding: low risk (< 2%), moderate risk (2%–4%), high risk (> 4%–8%), and highest risk (> 8%). Subsequently, we calculated absolute effects for each category for clinically important gastrointestinal bleeding and overt bleeding. We used the event rate in the placebo group of the SUP‐ICU trial (refer to the guideline outline—PICO #1 Supporting Information).

^d^ Rated down by 1 level for imprecision. The 95% CI includes an unimportant difference in clinically important GI bleeding.

^e^ SCCM NMA conducted by the guideline panel was not stratified by baseline bleeding risk, which may influence the applicability of the estimates to populations at different risk levels. For CIGIB, the comparison between Placebo and H2RA showed a direct estimate from conventional meta‐analysis (MA) of 1.84 (95% CrI, 0.74–4.56) and from node splitting analysis of 1.78 (95% CrI, 0.84–3.88), both with high certainty of evidence. The indirect estimate was 0.83 (95% CrI, 0.31–1.70) with moderate certainty, while the NMA estimate was 1.22 (95% CrI, 0.74–1.98) with low certainty.

^f^ 2020 updated SR and NMA showed moderate certainty evidence that H2RAs probably reduce CIB (complete: 0.67, 0.48–0.94): 20 fewer for high‐risk patients.

^g^ SCCM NMA conducted by the guideline panel was not stratified by baseline bleeding risk, which may influence the applicability of the estimates to different patient populations. For Overt GIB: PPI vs. Placebo, the direct estimate from conventional MA was 0.62 (95% CrI, 0.43–0.89), while node splitting analysis yielded 0.65 (95% CrI, 0.37–1.30), both with moderate certainty. The indirect estimate was 0.30 (95% CrI, 0.12–0.68), also with moderate certainty, while the NMA estimate was 0.52 (95% CrI, 0.30–0.81), with moderate certainty.

^h^ 2020 updated SR and NMA showed that both PPIs (RR 0.50, 95% CrI, 0.31–0.72, moderate certainty) and H2RAs (0.66, 0.48–0.89, moderate certainty) likely reduce overt bleeding.

^i^ SCCM NMA conducted by the guideline panel was not stratified by baseline bleeding risk, which may influence the applicability of the estimates to different patient populations. For Overt GIB: Placebo and H2RA showed a direct estimate from conventional meta‐analysis (MA) of 1.84 (95% CrI, 0.74–4.55) and from node splitting analysis of 1.78 (95% CrI, 0.86–3.88), both with high certainty of evidence. The indirect estimate was 0.84 (95% CrI, 0.31–1.70) with moderate certainty, while the NMA estimate was 1.22 (95% CrI, 0.74–1.98) with low certainty.

^j^ Serious inconsistency I^2^ = 55%.

^k^ 2020 updated SR and NMA revealed the following for mortality, including data from the PEPTIC trial: RR 1.03; 95% CrI, 0.93–1.14, moderate certainty. SCCM NMA conducted by the panel showed similar results: in the PPI vs. Placebo comparison, the direct estimate from conventional MA was 1.03 (95% CrI, 0.94–1.14), while the estimate from node splitting was 1.04 (95% CrI, 0.81–1.22), both with high certainty. The indirect estimate was 0.98 (95% CrI, 0.80–1.19) with high certainty, and the NMA estimate was 1.02 (95% CrI, 0.92, 1.40) with moderate certainty. These results indicate that PPI use does not significantly impact overall mortality compared to placebo.

^l^ Rated down by 1 level for imprecision. The 95% CrI includes an important increase and reduction in mortality.

^m^ Rated down for imprecision and risk of bias. As the point estimate was very close to the null, we switched to rate certainty with little to no effect. There is uncertainty resulting from subgroup analysis based on disease severity.

^n^ 2020 updated SR and NMA revealed the following for mortality, including data from the PEPTIC trial: RR 0.98; 95% CrI, 0.89–1.08, moderate certainty. SCCM NMA conducted by the panel showed similar results: Placebo and H2RA demonstrated a direct estimate from conventional MA of 1.05 (95% CrI, 0.87–1.27) and from node splitting analysis of 1.06 (95% CrI, 0.88–1.25), with high certainty of evidence. The indirect estimate was 1.02 (95% CrI, 0.85–1.23) with high certainty, while the NMA estimate was 1.04 (95% CrI, 0.93–1.16) with moderate certainty.

^o^ 2020 updated SR and NMA revealed the following for pneumonia, including data from the PEPTIC trial: RR 1.08, 95% CrI, 0.88–1.45, low certainty. SCCM NMA conducted by the panel showed similar results‐ PPI vs. Placebo comparison, the direct estimate from conventional MA was 1.02 (95% CrI, 0.88–1.19), while the estimate from node splitting was 1.05 (95% CrI, 0.82–1.48), both with high certainty. The indirect estimate was 1.39 (95% CrI, 0.93–2.07) with high certainty, and the NMA estimate was 1.14 (95% CrI, 0.93–1.54) with moderate certainty. These results indicate that PPI use does not significantly impact pneumonia risk compared to placebo, although there is some uncertainty in indirect estimates.

^p^ Rated down by 1 level. We are skeptical of the result because the pooled result, including smaller studies, conflicts with the evidence from the largest study (SUP‐ICU).

^q^ Rated down by 1 level. The 95% CrI includes no difference in pneumonia.

^r^ Rated down twice for imprecision. The point estimate was very close to the null; we switched to rate certainty with little to no effect.

^s^ 2020 updated SR and NMA revealed the following for pneumonia, including data from the PEPTIC trial: RR 1.07; 95% CrI, 0.85–1.37, low certainty. SCCM NMA conducted by the panel showed similar results: Placebo and H2RA demonstrated a direct estimate from conventional meta‐analysis of 0.89 (95% CrI, 0.60–1.33) and from node splitting analysis of 0.86 (95% CrI, 0.62–1.23), both with high certainty of evidence. The indirect estimate was 1.09 (95% CrI, 0.72–1.54) with high certainty, while the NMA estimate was 0.96 (95% CrI, 0.73–1.21) with moderate certainty. These findings suggest no significant difference in pneumonia risk between H2RA and placebo.

^t^ Rated down by 1 level. The 95% CrI includes an important increase and reduction in pneumonia.

^u^ Rated down twice for imprecision.

^v^ Indirect estimate was rated down because one of the direct estimates (PPIs vs. H2RAs) in the first‐order loop, which contributed to the indirect estimate, was rated down for risk of bias. No direct evidence.

^w^ The 95% CrI is very wide and includes an important increase and reduction in *Clostridioides difficile* infection.

The SCCM guideline recommends that “critically ill adults with coagulopathy, shock, or chronic liver disease be considered at risk for overt UGIB” and suggests using SUP in this population (conditional recommendation; low to moderate certainty in evidence) [3]. The 2020 BMJ Rapid guideline suggests SUP “in critically ill patients at high (4% to 8%) or highest (above 8%) risk of gastrointestinal bleeding,” while advising against prophylaxis in those at low (< 2%) or moderate (2% to 4%) bleeding risk [11]—an approach that closely parallels the current panel's recommendation, both in terms of population focus and the conditional nature of the recommendation based on moderate certainty of benefit and uncertain harms.

### 3.2 PICO 2: Should Critically Ill Adults With UGIB Risk Factors Who Are Enterally Fed Receive SUP or no SUP to Prevent UGIB?

#### 3.2.1 Recommendation 2

In critically ill adults who are enterally fed and at high risk for UGIB, the panel suggests using SUP over no SUP (conditional recommendation, very low certainty of evidence).

In critically ill adults who are enterally fed and at low risk for UGIB, the panel suggests not using SUP (conditional recommendation, very low certainty of evidence).

#### 3.2.2 Rationale

We updated the existing systematic review and meta‐analysis by incorporating data from two previous systematic reviews and additional randomized controlled trials. We also evaluated exploratory data from the SUP‐ICU trial (Supplemental Content 2: 2.15–2.17) [40, 41, 42, 43, 44]. Findings were inconclusive, with very low certainty of evidence across outcomes.

In critically ill adults who are enterally fed, SUP may result in little to no difference in CIGIB (RR, 1.03; 95% CI, 0.57–1.86; very low certainty of evidence) [40, 41, 42, 43]. Similarly, SUP may result in little to no difference in overt UGIB (RR, 0.88; 95% CI, 0.51–1.50; very low certainty of evidence), with an absolute risk difference of 6 fewer per 1000 (95% CI, 24 fewer to 24 more) [40, 41, 42, 43]. The effect on mortality was similarly uncertain (RR, 1.23; 95% CI, 0.98–1.54; very low certainty of evidence) [40, 41, 42, 43]. The certainty of the evidence was downgraded due to indirectness, imprecision, and limitations in subgroup analyses, particularly because enteral nutrition (EN) status was inconsistently assessed—often post‐randomization—raising concerns about internal validity.

SUP may increase the risk of hospital‐acquired pneumonia (RR, 1.53; 95% CI, 1.08–2.16; very low certainty of evidence) [40, 41, 42, 43]. SUP may increase the risk of VAP (RR, 1.31; 95% CI, 0.82–2.08; very low certainty of evidence), with an absolute difference of 35 more per 1000 (95% CI, 20 fewer to 123 more); however, the evidence is very uncertain [40, 41, 42, 43]. SUP may result in little to no difference in the risk of *C. difficile* infection (RR, 0.91; 95% CI, 0.21–3.85; very low certainty of evidence), although this estimate was based on very few events and is highly imprecise [40, 41, 42, 43].

Post hoc analyses from the SUP‐ICU trial showed that exposure to EN was associated with a lower risk of GI bleeding, including clinically important (HR, 0.29; 95% CI, 0.19–0.44) and overt bleeding (HR, 0.33; 95% CI, 0.25–0.44) [44]. Pantoprazole also reduced bleeding risk (clinically important bleeding HR, 0.64; 95% CI, 0.43–0.96; overt bleeding HR, 0.58; 95% CI, 0.44–0.76) [44]. EN was associated with an increased risk of pneumonia (HR, 1.44; 95% CI, 1.14–1.82), whereas pantoprazole alone had a neutral effect (HR, 1.00; 95% CI, 0.84–1.19) [44]. EN was associated with reduced all‐cause mortality (HR, 0.22; 95% CI, 0.18–0.27), but when combined with pantoprazole, the association was compatible with increased mortality (HR, 1.27; 95% CI, 0.99–1.64; interaction *p* = 0.024) [44]. These findings should be interpreted cautiously, given potential confounding and the lack of randomization by EN status.

The panel judged the undesirable effects of SUP as varying in magnitude and the balance between desirable and undesirable consequences of SUP as dependent on patient‐specific bleeding risk. For high‐risk enterally fed patients, the modest potential bleeding reduction may justify SUP, while in low‐risk patients, the risk of avoidable infections likely outweighs uncertain benefits. SUP medications are inexpensive and widely available, and EN is a standard ICU practice, although interruptions for procedures or intolerance may reduce its protective effect. The panel concluded that SUP may be considered in critically ill, enterally fed adults with high UGIB risk factors but should be avoided in low‐risk patients. Individualized assessment should consider baseline bleeding risk, institutional infection rates, and patterns of EN delivery. This recommendation is consistent with the 2024 SCCM guideline, incorporating EN status and bleeding risk into clinical decision‐making [3]. The 2020 BMJ Rapid Recommendation did not evaluate the effect of EN [11]. Information for Evidence Profile and EtD Framework is available in Supplemental Content 2: 2.18–2.19.

### 3.3 PICO 3: In Critically Ill Adults With Risk Factors for Stress‐Related UGIB, What Are the Comparative Effectiveness and Harms of SUP Agents (PPIs, H2RAs, or Sucralfate) When Used at Low Doses Versus High Doses or Administered via Enteral Versus Intravenous Routes?

#### 3.3.1 Recommendation 3.1: Agent Selection

In critically ill adults with UGIB risk factors, the panel suggests using either a PPI or an H2RA over sucralfate for SUP (conditional recommendation, low to moderate certainty of evidence).

#### 3.3.2 Remark

The choice between PPI and H2RA may be guided by bleeding risk, illness severity, and availability. PPIs may offer greater benefit in higher‐risk patients, whereas H2RAs may be preferred in those with high illness acuity due to potential safety concerns with PPIs.

#### 3.3.3 Rationale

Evidence was drawn from updated meta‐analyses (Wang et al.), three NMAs from the source guideline, and major RCTs (SUP‐ICU, REVISE, and PEPTIC) [3, 25, 26, 27, 28, 29, 45] (Supplemental Content 2: 2.20–2.24).

PPIs likely reduce CIGIB compared with H2RAs, particularly in higher‐risk patients. At low baseline risk (6 per 1000), PPIs probably reduce bleeding slightly (3 fewer per 1000), while in high‐risk groups (≥ 28 per 1000), PPIs likely reduce bleeding by 10–13 per 1000 (low to moderate certainty) [25, 26, 27, 28, 29, 45]. Compared with sucralfate, PPIs probably reduce bleeding to a greater extent, particularly in patients at moderate to high risk. Absolute reductions were larger, ranging from 17 to 32 fewer events per 1000 (moderate certainty) [25, 26, 27, 28, 29, 45]. H2RAs may also reduce bleeding compared with sucralfate (approximately 8 to 12 fewer per 1000; low to moderate certainty), but sucralfate likely results in the least reduction in bleeding across all comparisons [25, 26, 27, 28, 29, 45]. There were no important differences in ICU length of stay or duration of mechanical ventilation observed between any agent classes [25, 26, 27, 28, 29, 45]. Overall, the current evidence suggests that PPIs have little or no effect on mortality compared with H2RAs (RR, 1.05; 95% credible interval [CrI], 0.97–1.14; low certainty of evidence), but some uncertainty remains [25, 26, 27, 28, 29, 45]. Subgroup analyses from the PEPTIC and SUP‐ICU trials suggested a potential increase in mortality among sicker patients receiving PPIs; however, the credibility of this effect is low, as these analyses were exploratory and not sufficiently powered [28, 29, 45]. PPIs may result in little to no difference in pneumonia risk compared with H2RAs (RR, 1.02; 95% CrI, 0.80–1.33; low certainty of evidence) [25, 26, 27, 28, 29, 45]. Sucralfate may slightly reduce pneumonia risk compared to both PPIs and H2RAs, with absolute risk reductions of 25 and 23 per 1000 patients, respectively (moderate to low certainty) [25, 26, 27, 28, 29, 45]. Therefore, undesirable effects related to pneumonia are likely small, with sucralfate offering a modest potential advantage. PPIs likely result in little to no difference in risk of *C. difficile* infection compared with H2RAs (RR, 0.76; 95% CrI, 0.28–2.16; moderate certainty of evidence) [25, 26, 27, 28, 29, 45]. The absolute risk difference was small (4 fewer per 1000; 95% CrI, 31 fewer to 6 more), and undesirable effects were trivial to small, likely reflecting the low baseline risk of infection [25, 26, 27, 28, 29, 45].

#### 3.3.4 Recommendation 3.2: Route of Administration

In critically ill adults receiving SUP, the panel suggests either enteral or intravenous administration, depending on clinical context and feasibility (conditional recommendation, very low to low certainty of evidence).

#### 3.3.5 Remark

Evidence does not favor one route over the other. When feasible, the enteral route may reduce cost and simplify administration.

#### 3.3.6 Rationale

Evidence does not favor one route. Oral PPIs appeared more effective than intravenous (IV) H2RAs or sucralfate in indirect comparisons; however, this likely reflects confounding by illness severity (enterally fed patients were less acutely ill, while IV administration was more common in hemodynamically unstable or mechanically ventilated patients). This finding is based on indirect evidence, and certainty in this comparison was rated as very low to low due to serious imprecision and indirectness [3, 25, 26, 27, 46]. Adverse events did not differ meaningfully by route. The panel judged the overall burden of harms as trivial to small across route comparisons [3, 25, 26, 27].

#### 3.3.7 Statement 3.3: Dosing Strategy

In critically ill adults receiving stress ulcer prophylaxis, low‐dose PPI or H2RA therapy rather than high‐dose regimens should be used (Best Practice Statement—not GRADEd).

#### 3.3.8 Remark

Higher doses provide no additional benefit and may increase cost and adverse effects. Low‐dose PPI therapy is defined as a total daily dose of ≤ 40 mg of esomeprazole, omeprazole, or pantoprazole or ≤ 30 mg of lansoprazole. Low‐dose H2RA therapy is defined as a total daily dose of ≤ 40 mg of famotidine, ≤ 150 mg IV ranitidine, ≤ 300 mg enteral ranitidine, or ≤ 1200 mg of cimetidine.

#### 3.3.9 Rationale

Available comparisons suggest no additional benefit from high‐dose regimens. PPI low‐dose versus high‐dose showed similar bleeding outcomes across trials, with trivial risk differences (< 3 per 1000) [3, 25, 26, 27]. Similar findings were observed in H2RA dose comparisons. Certainty of evidence was rated low to very low due to imprecision and heterogeneity. Thus, escalation to high‐dose regimens does not appear justified in the absence of clear clinical indications. Mortality, pneumonia, and *C. difficile* risk were similar.

Overall, the evidence supports individualized agent selection based on bleeding risk, careful interpretation of administration route data due to confounding, and a general preference for low‐dose regimens. The panel considered PPIs and H2RAs to be broadly available, inexpensive, and feasible in ICU practice. Sucralfate was less acceptable due to limited efficacy, dosing burden, and declining availability. In Saudi Arabia, ranitidine is withdrawn, cimetidine is rarely used, and PPIs are widely available. In the Nordic Countries, H2RAs are unavailable in some countries, leaving PPIs as the default option [38]. Sucralfate, while inexpensive, is less effective and requires more frequent dosing (increasing the administration burden) and may obstruct nasogastric tubes.

The panel concluded that PPIs or H2RAs are preferable to sucralfate, with PPIs offering greater benefit in patients with higher bleeding risk. Enteral or IV routes are acceptable depending on clinical context, although enteral may be preferred for cost and simplicity. Low‐dose regimens are recommended, as higher doses offer no meaningful advantage.

Our recommendations align with the 2024 SCCM guideline, which supports PPIs or H2RAs as first‐line agents and emphasizes low‐dose therapy via either enteral or IV routes based on clinical context [3]. The 2020 BMJ guideline favors PPIs over H2RAs and recommends against sucralfate [11]. Our panel judged the absolute PPI benefit modest and highlighted potential safety concerns in high‐acuity patients, supporting a shared decision‐making approach that incorporates bleeding risk, illness severity, and local drug availability.

### 3.4 PICO 4: Should Critically Ill Adults Whose UGIB Risk Factors Are no Longer Present Continue or Discontinue SUP?

#### 3.4.1 Recommendation 4

In critically ill adults with resolved risk factors for UGIB, the panel suggests discontinuing SUP (conditional recommendation, very low certainty of evidence).

#### 3.4.2 Rationale

This recommendation represents a contextualized adaptation: unlike the SCCM source guideline, which offered a good‐practice statement, our panel developed a formal conditional recommendation based on emerging real‐world evidence and region‐specific implementation challenges. Differences in post‐ICU care continuity between Gulf and Nordic hospitals—such as electronic medication reconciliation systems and pharmacist‐led discharge processes—were explicitly considered to ensure feasibility and equity of SUP discontinuation.

Evidence synthesis for PICOs 4 and 5 was combined, as both address whether SUP should be continued once bleeding risk factors have resolved. The evidence base—derived mainly from observational studies [47, 48, 49, 50, 51, 52], a single RCT, and quality‐improvement initiatives [53, 54, 55, 56, 57, 58, 59, 60]—was indirect and low in certainty (Supplemental Content 2: 2.25–2.28). Across settings, 60%–74% of patients in the ICU continue SUP unnecessarily after transfer or discharge [48, 54]. Deprescribing interventions (e.g., pharmacist‐led reviews and checklists) considerably reduce inappropriate continuation without increasing bleeding and generate large cost savings [53, 58].

Observational data suggest potential harm from unnecessary continuation, including higher risks of pneumonia, cardiovascular events, renal failure, and certain cancers [54]. These findings, though observational, highlight the safety and desirability of discontinuation once UGIB risk factors are resolved. Importantly, no study demonstrated increased UGIB risk following discontinuation in low‐risk or recovered patients [45, 54]. From a contextual standpoint, deprescribing is feasible and highly acceptable. In Gulf ICUs, implementation may require structured discharge checklists and pharmacist engagement, whereas Nordic systems benefit from automated medication reconciliation within integrated registries. Both models support equity and sustainability through reduced drug burden and costs.

### 3.5 PICO 5: Should Critically Ill Adults Without UGIB Risk Factors but Receiving SUP Before ICU Admission Continue or Discontinue SUP?

#### 3.5.1 Recommendation 5

In critically ill adults without UGIB risk factors but receiving SUP prior to ICU admission, the panel suggests discontinuing SUP in the absence of an active indication (conditional recommendation, very low certainty of evidence).

#### 3.5.2 Remarks

SUP indications include recent UGIB, erosive esophagitis, *Helicobacter pylori* therapy, and Zollinger‐Ellison syndrome. When history is unclear, discontinuation may be deferred pending verification from outpatient records or the prior prescribing physician.

#### 3.5.3 Rationale

This recommendation was developed *de novo* to reflect contextual factors unique to the participating regions. In Saudi and Kuwaiti ICUs, variable outpatient follow‐up and formulary continuity necessitate structured deprescribing tools. In Nordic systems, integrated electronic health records and national medication registries facilitate timely medication reconciliation and discontinuation of unnecessary SUP. The panel acknowledged that active pharmacist‐led deprescribing and physician oversight can substantially support safe SUP discontinuation; however, such expertise is not uniformly available across ICUs in either region. Accordingly, feasibility judgments focused on system‐level processes, health‐system infrastructure, and routine care pathways.

Across settings, continuation of SUP without indication contributes to avoidable costs and adverse events. Observational data and FDA pharmacovigilance reports link long‐term PPI use to kidney disease, micronutrient deficiencies, and infections [52, 60]. Conversely, deprescribing is cost‐saving, improves medication safety, and aligns with patient and clinician preferences to minimize unnecessary therapies.

The panel concluded that SUP discontinuation is both safe and cost‐effective, with strong feasibility in both Gulf and Nordic ICUs when embedded within multidisciplinary discharge processes. This adaptation extends the SCCM good‐practice statement into a conditional recommendation grounded in emerging low‐certainty but regionally applicable evidence.

## 4 Discussion

This guideline presents five prioritized recommendations for SUP in critically ill adults, contextualized for Saudi Arabia, Kuwait, and the Nordic countries using the GRADE‐ADOLOPMENT methodology, as outlined in the WHO handbook “Strengthening countries' capacities to adopt and adapt evidence‐based guidelines” [61]. Contextualization ensured that each recommendation is both evidence‐based and adapted to the realities of regional health systems, including differences in healthcare infrastructure, drug availability, and prescribing practices. By leveraging credible source guidelines, this hybrid approach balances methodological rigor with practical efficiency. Each recommendation was developed using an EtD framework that integrated global evidence with regional data and newly identified studies. This initiative marks the first multinational ADOLOPMENT effort between SCCS and SSAI, with endorsement from the KACCS, reflecting broad regional engagement and applicability.

### 4.1 Modifications and Regional Contextualization

Two recommendations (PICOs 4 and 5) were substantially modified via *de novo* EtD frameworks, moving from best practice statements in the source guideline to formal conditional recommendations, reflecting new evidence, local clinical experience, and regional microbiological concerns (e.g., *C.difficile* and VAP). These revisions highlight the panel's emphasis on SUP deprescribing as part of ICU stewardship, with prolonged use deemed potentially harmful when risk factors resolve. Therefore, they were classified as *de novo* due to significant contextual reinterpretation and methodological restructuring.

The remaining three recommendations retained their direction and certainty from the source guideline but were updated with recent evidence and local contextual data, including drug availability, formulary restrictions, and access disparities across sectors and regions. These refinements influenced judgments on agent selection, risk stratification, and implementation feasibility while maintaining methodological integrity.

Together, these modifications illustrate the dynamic capability of the GRADE‐ADOLOPMENT framework to support full or partial adaptation based on the extent to which global evidence maps onto local clinical, economic, and logistical contexts [4, 62]. This approach aligns with experiences in other ADOLOPMENT efforts, such as the American Society of Hematology (ASH) guideline adapted for Latin America, where resource constraints and health system variability justified modifying original recommendations or focusing on different populations [62]. By systematically incorporating contextual factors—cost, equity, feasibility, and healthcare infrastructure—the framework ensures that recommendations remain both evidence‐informed and practically implementable.

Although geographically distant, Saudi Arabia, Kuwait, and the Nordic countries share several system‐level features relevant to contextualized guideline development—organized critical‐care societies and a strong culture of evidence‐based practice supporting multidisciplinary consensus building—yet they differ in healthcare systems, formulary structures, and access [5, 6, 7]. These contrasts were explicitly addressed through the GRADE‐ADOLOPMENT Evidence‐to‐Decision process to ensure that feasibility and equity judgments reflected each region's realities. Therefore, the collaboration represents a pragmatic methodological partnership rather than an assumption of clinical homogeneity. Although tailored for these three regions, the methodological framework and contextual insights provide a transferable model for other health systems seeking to adapt global guidance to their own settings, demonstrating how regional adaptation can balance global evidence with local realities to maximize feasibility, equity, and uptake of SUP recommendations.

### 4.2 Implementation and Clinical Impact

The adapted recommendations aim to standardize SUP use and minimize inappropriate prescribing across ICU settings. EtD frameworks and justification summaries were designed to enhance transparency and support clinical reasoning. Implementation will be driven by national critical care societies, with dissemination through digital platforms, ICU networks, and local policy tools. Tailored strategies—such as integration into ICU protocols, pharmacist‐led stewardship, and staff education—will be critical to uptake, especially given variability in ICU infrastructure and formulary access (Figure 1). As with our prior collaborative initiatives [63, 64, 65, 66, 67, 68, 69, 70, 71, 72, 73], multi‐stakeholder engagement and operational guidance are essential for translating recommendations into practice. Future implementation research should assess guideline uptake, particularly in mixed public‐private systems (Saudi Arabia) and decentralized care models (Nordic countries).

Across major trials, notable heterogeneity of treatment effects has been observed according to baseline bleeding risk, illness severity, and adequacy of enteral feeding. Subgroup analyses from the SUP‐ICU and REVISE trials indicate that the absolute benefit of PPI in preventing clinically important UGIB rises with increasing baseline UGIB risk, whereas potential harms such as infection or mortality may emerge among the most severely ill patients [28, 29]. Similarly, the PEPTIC trial demonstrated variable mortality patterns among patients with the highest illness severity, reinforcing a risk‐stratified rather than universal approach to SUP [45]. Our panel incorporated these heterogeneous effects into the EtD frameworks by explicitly linking benefit estimates to regional risk distributions and patient profiles. Future updates should explore patient‐level meta‐analyses to refine individualized thresholds for prophylaxis.

A secondary analysis of the REVISE trial by Deane et al. further quantified risk variation, identifying patient‐important UGIB in 2.7% of mechanically ventilated adults using outcomes prioritized by ICU survivors and family members [74]. Independent risk factors included higher illness severity (per 5‐point increase in APACHE II; HR, 1.24; 95% CI, 1.12–1.37), vasopressor or inotrope use (HR, 2.05; 95%, CI 1.35–3.12), severe thrombocytopenia (< 50 × 10^9^/L; HR, 2.21; 95% CI, 1.24–3.94), and concurrent antiplatelet therapy (HR, 1.69; 95% CI, 1.11–2.56). Protective factors included pantoprazole prophylaxis (HR, 0.36; 95% CI, 0.25–0.54) and higher volumes of enteral nutrition (HR, 0.81 per 500 mL/day; 95% CI, 0.68–0.97), with no interaction between the two (interaction *p* = 0.94). These findings support our interpretation that acid suppression and adequate enteral feeding exert independent, additive protective effects. Clinically important bleeding remains concentrated among patients with severe physiologic derangements and coagulopathy, whereas fully enterally fed, hemodynamically stable patients derive minimal incremental benefit from pharmacologic prophylaxis. Together, these data substantiate the panel's conditional recommendations (PICOs 1 and 2) to reserve SUP for high‐risk patients and avoid routine prophylaxis in low‐risk, fully fed individuals.

### 4.3 Comparisons With Other Guidelines and Updating Plan

These adapted recommendations largely align with the source guideline framework, but divergence occurred where contextual feasibility or health equity concerns dictated. Such divergence reflects an increasing trend in global guideline adaptation, as evidenced by other efforts (e.g., ASH venous thromboembolism guideline for Latin America and the American College of Rheumatology guidelines for rheumatoid arthritis in both the Eastern Mediterranean Region and Saudi Arabia), where panels shifted the balance of benefits and harms based on local infrastructure or reinterpreted the strength of recommendations to reflect target population relevance [5, 63]. Such departures highlight the importance of EtD transparency, enabling future readers and implementers to trace how setting‐specific realities influenced judgments. Our guideline coordination group will monitor updates to relevant high‐quality systematic reviews to evaluate whether new evidence warrants an update to the adapted recommendations.

### 4.4 Limitations

Limitations include no direct representation from patients and health economists, reducing the diversity of perspectives. However, patient values were incorporated indirectly through the GRADE EtD frameworks, informed by data from SUP‐ICU; REVISE; and describing how patients and families balance the avoidance of UGIB, transfusion, and endoscopic procedures against infection risk and medication burden [28, 29]. Future updates will aim to include formal patient and health economic representation, consistent with evolving Guidelines International Network and WHO adaptation standards [61]. Equity considerations addressed disparities in ICU access between urban and rural areas and between national and expatriate populations. Variable resource availability across institutions may affect implementation consistency. Additionally, the absence of a formal cost‐effectiveness analysis (CEA) limits the economic assessment. Future iterations should incorporate formal CEA and broader stakeholder engagement. Key priorities include evaluating SUP economic impact, procurement logistics, and outcomes of early discontinuation. We recommend prospective data collection on SUP‐related adverse events and benchmarking across ICU subpopulations to guide updates and support implementation.

### 4.5 Future Research Directions

**FIGURE 2:**
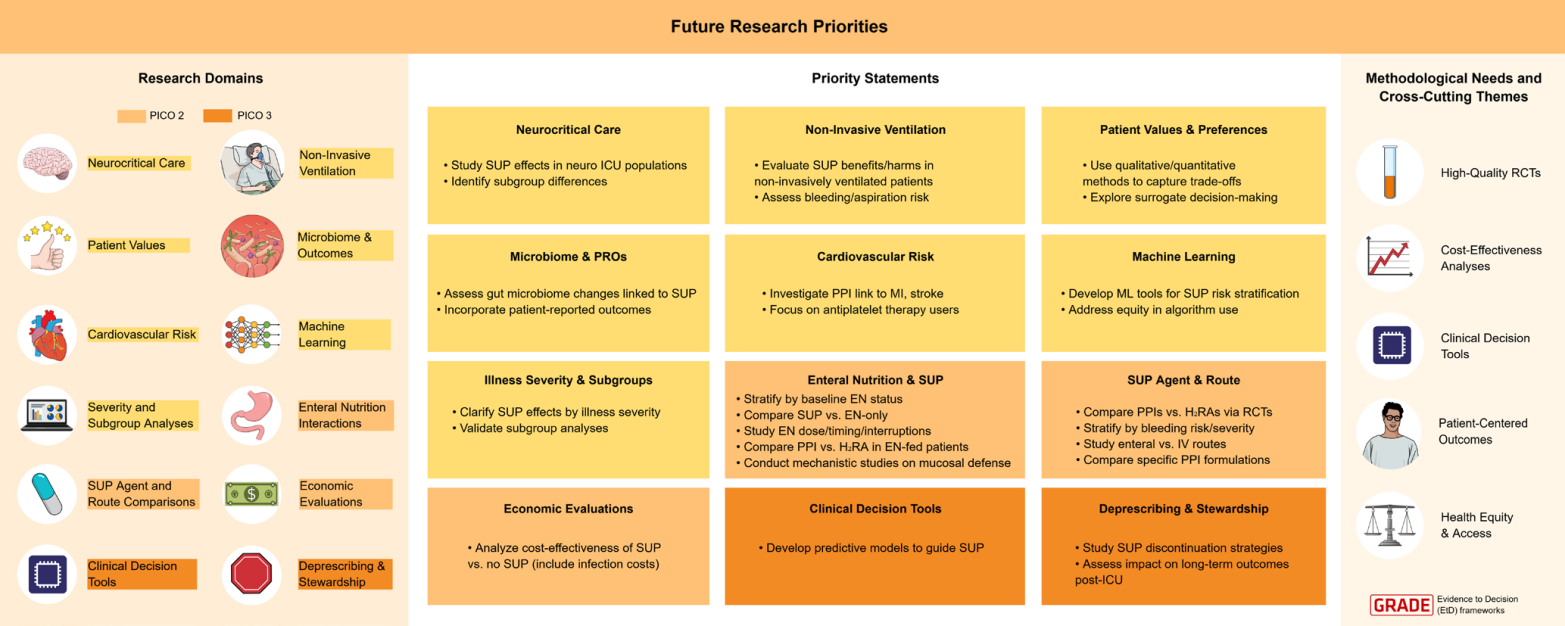
Future research priorities. Priority domains include neurocritical care, non‐invasive ventilation, patient values, microbiome‐related outcomes, cardiovascular safety, EN interactions, pharmacologic agent comparisons, and SUP deprescribing strategies. For neurocritical care, evidence is limited and may not generalize due to altered GI physiology and clinical management. Patients receiving non‐invasive ventilation represent an understudied group with distinct bleeding and aspiration risk profiles. Future studies should evaluate patient and surrogate preferences, focusing on trade‐offs between bleeding and infection risks, and incorporate PROs and gut microbiome data to better understand SUP‐associated complications. Research should also assess possible associations between PPI use and cardiovascular events, particularly in patients on antiplatelet therapy. Within PICO 2, research is needed to stratify patients by EN status at baseline, compare pharmacologic SUP versus EN‐only in different risk groups, and assess how feeding interruptions impact UGIB risk. Mechanistic studies are needed to define the physiologic effects of EN on mucosal defense. For PICO 3, comparative effectiveness trials should assess PPIs versus H_2_RAs stratified by illness severity and bleeding risk, as well as evaluate route of administration and formulation‐specific issues (e.g., omeprazole vs. pantoprazole) in terms of absorption, safety, and cost‐effectiveness. Economic evaluations across diverse healthcare systems are needed, particularly in resource‐limited settings. Clinical decision tools should be developed to enhance individualized risk assessment and support targeted SUP use. PICOs 4 and 5 highlight the need for evidence on deprescribing strategies, including optimal timing for SUP discontinuation, structured risk stratification, and monitoring of post‐ICU outcomes such as readmission and bleeding events. Implementation studies should evaluate EHR‐based alerts, pharmacist‐led reviews, and stop orders to support deprescribing. Additional research should focus on vulnerable subgroups (e.g., older adults, cirrhotic, immunocompromised), as well as long‐term adverse outcomes of unnecessary SUP continuation post‐discharge, such as kidney injury or malabsorption. Equity‐focused research is needed to ensure deprescribing practices are accessible and effective across all ICU populations. EHR, electronic health record; EN, enteral nutrition; H_2_RA, histamine‐2 receptor antagonist; ICU, intensive care unit; PPI, proton pump inhibitor; PRO, patient‐reported outcome; RCT, randomized controlled trial; SUP, stress ulcer prophylaxis; UGIB, upper gastrointestinal bleeding.

The panel identified several potential research priorities (Figure 2, Supplemental Content 2: 2.29). The following are recommendations to address the remaining uncertainties and guide further refinement:

- Multicenter studies in Gulf and Nordic ICUs evaluating SUP discontinuation timing, bleeding risk stratification, and downstream complications.
- Cost‐effectiveness studies assessing the economic impact of SUP protocols across health systems.
- Evaluation of implementation strategies, such as clinical decision support, order set integration, and ICU pharmacist engagement.
- Future work should also explore artificial intelligence (AI)‐enabled decision support to enhance precision in SUP prescribing. AI has the potential to improve risk stratification, therapeutic targeting, and deprescribing by integrating patient‐specific data and adapting to evolving clinical patterns. Embedding such tools into electronic health records, co‐developed with frontline clinicians, could reduce adverse drug events and inappropriate continuation, offering a promising avenue to strengthen safe and efficient SUP use.

## 5 Conclusion

This guideline provides five context‐specific, evidence‐informed recommendations for SUP in critically ill adults in Saudi Arabia, Kuwait, and the Nordic regions. It supports safe and resource‐conscious clinical practice while reducing unnecessary SUP use. The panel also identified key research priorities to address remaining uncertainties and guide future updates.

## Author Contributions

The coordination group included members of the Steering Committee (Marwa Amer [Deputy Chair, SCCS Guideline Chapter], Waleed Alhazzani [Chair, SCCS Guideline Chapter], and Morten Hylander Møller) and two methodologists (Marwa Amer and Fayez Alshamsi). Marwa Amer was the lead methodologist and chair, overseeing the guideline development process, coordinating panel meetings, supervising the adaptation workflow, facilitating communication among stakeholders, and ensuring adherence to GRADE‐ADOLOPMENT standards. Initial collaborative efforts were initiated through a teleconference between Marwa Amer and Morten Hylander Møller to explore the guideline's relevance for SSAI. Ahmed Aljedai contributed as a health policy expert and panel member. Abdulrahman Al‐Fares is representative of the Kuwait Anesthesia and Critical Care Society Endorsement. Yaseen M. Arabi, Morten Hylander Møller, Waleed Alhazzani, Anders Granholm, and Abdulrahman Al‐Fares are co‐authors on major randomized trials (SUP‐ICU and REVISE trials) and brought critical expertise. Morten Hylander Møller, Waleed Alhazzani, and Anders Granholm are key contributors to the SCCM stress ulcer prophylaxis guideline. Faisal A. Al‐Suwaidan and Mohammed Alshahrani are members of the SCCS Guideline Chapter Advisory Committee. None of the panelists disclosed any financial COIs. No panelists received payments from industry. Methodologists (Marwa Amer and Fayez Alshamsi) were free from commercial interests and played a non‐voting facilitation role. All authors read and approved the final manuscript. Additional details of individual contributions are available in Supplemental Content 2: 2.1–2.2.

## Funding

The authors have nothing to report.

## Ethics Statement

The authors have nothing to report.

## Consent

The authors have nothing to report.

## Conflicts of Interest

The authors declare no conflicts of interest.

## Supporting information


**Data 1** AGREE Reporting Checklist 2016.

**Data 2** Recommendations for Stress Ulcer Prophylaxis in Critically Ill Adults: A Contextualized Clinical Practice Guideline from the Saudi Critical Care Society and the Scandinavian Society of Anaesthesiology and Intensive Care Medicine, Endorsed by the Kuwait Anesthesia and Critical Care Society.

**Data 3** Outcomes and PICO Prioritization.

## Data Availability

The data that support the findings of this study are available in the Supporting Information of this article.
