# Supplementary material for: Recommendations for Stress Ulcer Prophylaxis in Critically Ill Adults: A Contextualized Clinical Practice Guideline From the Saudi Critical Care Society and the Scandinavian Society of Anaesthesiology and Intensive Care Medicine, Endorsed by the Kuwait Anesthesia and Critical Care Society
Source: Acta Anaesthesiol Scand. 2026 Feb 8;70(3):e70201. doi: 10.1111/aas.70201 (PMC12883282; doi:10.1111/aas.70201)
Supplement: Supplementary file 2 — Data 2 Recommendations for Stress Ulcer Prophylaxis in Critically Ill Adults: A Contextualized Clinical Practice Guideline from the Saudi Critical Care Society and the Scandinavian Society of Anaesthesiology and Intensive Care Medicine, Endorsed by the Kuwait Anesthesia and Critical Care Society. [file AAS-70-0-s001.pdf]

Supplementary Content 2

Recommendations for Stress Ulcer Prophylaxis in Critically Ill Adults: A Contextualized Clinical Practice Guideline from the Saudi Critical Care Society and the Scandinavian Society of Anaesthesiology and Intensive Care Medicine, Endorsed by the Kuwait Anesthesia and Critical Care Society

Marwa Amer[1,2], Waleed Alhazzani [3,4], Fayez Alshamsi[5], Anders Granholm[6,7], Yaseen M. Arabi[8], Klaus T. Olkkola[9], Marius Rehn[10,11,12], Abdulrahman Al-Fares[13,14], Rakan M. Alqahtani[15], Ahmed Aljedai [2,16], Haifa F. Alotaibi[3], Amr Arafat[3,17], Shadan AlMuhaidib[3], Ali Al Bshabshe[18], Ville Jalkanen[19], Martin Ingi Sigurðsson[20,21], Michelle S. Chew[22], Maija-Liisa Kalliomäki[23], Hasan M. Al-Dorzi[8], Fawziah Alkhaldi[24], Haifa Algethamy[25], Majid A. Almadi[26,27], Namareq Aldardeer[28], Abdullah M. Alhammad[29], Awad Al-Omari[30], Faisal A. Al-Suwaidan[31,32,33,34], Mohammed Alshahrani[35], Morten Hylander Møller[6,36]

[1]Medical/Critical Pharmacy Division, King Faisal Specialist Hospital and Research Center, Riyadh, Saudi Arabia

[2]College of Medicine and Pharmacy, Alfaisal University, Riyadh, Saudi Arabia

[3] Health Research Center of Excellence, Ministry of Defense Health Services, Riyadh, Saudi Arabia

[4]Critical Care and Internal Medicine Department, College of Medicine, Imam Abdulrahman Bin Faisal University, Dammam, Saudi Arabia

[5]Department of Internal Medicine, College of Medicine and Health Sciences, United Arab Emirates University, Al Ain, United Arab Emirates

[6]Department of Intensive Care, Copenhagen University Hospital—Rigshospitalet, Copenhagen, Denmark

[7]Section of Biostatistics, Department of Public Health, University of Copenhagen, Copenhagen, Denmark

[8]Intensive Care Department, Ministry of National Guard Health Affairs, King Abdullah International Medical Research Center, King Saud Bin Abdulaziz University for Health Sciences, Riyadh, Saudi Arabia

[9]Department of Anaesthesiology, Intensive Care and Pain Medicine, University of Helsinki and Helsinki University Hospital, Helsinki, Finland

[10]Division of Prehospital Services, Air Ambulance Department, Oslo University Hospital, Oslo, Norway

[11] The Norwegian Air Ambulance Foundation, Oslo, Norway

[12]Institute of Clinical Medicine, University of Oslo, Oslo, Norway

[13]Department of Anesthesia, Critical Care Medicine and Pain Medicine, Al-Amiri Hospital, Minister of Health, Kuwait City, Kuwait

[14]Kuwait Extracorporeal Life Support Program, Al-Amiri Center for Advance Respiratory and Cardiac Failure, Ministry of Health, Kuwait City, Kuwait

[15]Department of Critical Care Medicine, College of Medicine, King Saud University, Riyadh, Saudi Arabia

[16]Therapeutic Affairs, Ministry of Health, Riyadh, Saudi Arabia

[17]Adult Cardiac Surgery Department, Prince Sultan Cardiac Center, Riyadh, Saudi Arabia

[18]Department of Medicine /Adult Critical Care, College of Medicine, King Khalid University, Abha, Saudi Arabia

[19]Department of Intensive Care Medicine, Tampere University Hospital, Tampere, Pirkanmaa, Finland

[20]Faculty of Medicine, University of Iceland, Iceland

[21]Division of Anaesthesia and Intensive Care Medicine, Landspitali—The National University Hospital of Iceland, Iceland

[22]Department of Perioperative Medicine and Intensive Care, Karolinska University Hospital, Stockholm, Sweden

[23]Department of Anaesthesia, Tampere University Hospital, Tampere, Finland

[24]Department of Intensive Care Nursing, King Faisal Specialist Hospital and Research Centre, Riyadh, Saudi Arabia

[25]Department of Anesthesia and Critical Care, King Abdulaziz University, Jeddah, Saudi Arabia

[26]Division of Gastroenterology, Department of Medicine, College of Medicine, King Khalid University Hospital, King Saud University Medical City, King Saud University, Riyadh, Saudi Arabia.

[27]Division of Gastroenterology, The McGill University Health Center, Montreal General Hospital, McGill University, Montreal, Canada

[28]Medical and Clinical Affairs Department, King Faisal Specialist Hospital and Research Center-Jeddah

[29]Department of Clinical Pharmacy, College of Pharmacy, King Saud University, Riyadh, Saudi Arabia

[30]Chief Executive Officer, Almana Medical Group, Saudi Arabia

[31]Neurology Clinical Lead, Ministry of Health, Saudi Arabia

[32]Division of Neurology, Security Forces Hospitals Program, General Directorate of Medical Services, Ministry of Interior, Saudi Arabia

[33]College of Medicine, Princess Nourah Bint Abdulrahman University, Riyadh, Saudi Arabia

[34]College of Medicine, Dar Al-Uloom University, Riyadh, Saudi Arabia

[35]Department of Emergency and Critical Care, King Fahd Hospital of the University, Imam Abdulrahman Bin Faisal University, Dammam, Saudi Arabia

[36]Department of Clinical Medicine, University of Copenhagen, Copenhagen, Denmark

†**Corresponding author:** Marwa Amer, PharmD, B.S. Pharm, BCPS, BCCCP, FCCM

King Faisal Specialist Hospital & Research Center, Al Mathar Ash Shamali, Riyadh 11564, Saudi Arabia. Adjunct Assistant Professor- Alfaisal University- College of Medicine Tel: +966114647272 Ext 70836 or 48030; Email address: mamer@kfshrc.edu.sa, amerdrmarwa@gmail.com

Table of Contents

Supplemental Content #	Title	Page #
Supplemental Content 2.1	Selection and Organization of Committee Members	5–7
Supplemental Content 2.2	Conflict of Interest Management	8–10
Supplemental Content 2.3	Outcome Prioritization	11–13
Supplemental Content 2.4	GRADE Methodology	14–18
Supplemental Content 2.5	Implications of Different Recommendations to Key Stakeholders and the Voting Process	19–20
Supplemental Content 2.6	Definitions and Criteria for Recommendation Classification and Framework for Contextualization	21–22
Supplemental Content 2.7	Estimate Baseline Risk for Clinically Important GI Bleeding (CIGIB) and Overt Bleeding	23–28
Supplemental Content 2.8	AMSTAR-2 Assessment of Systematic Reviews for PICO 1	29–30
Supplemental Content 2.9	Characteristics of Systematic Reviews Evaluated for PICO 1	31–34
Supplemental Content 2.10	GRADE Summary of Findings for Wang 2020 for PICO 1	35–41
Supplemental Content 2.11	NMA From Source Guideline for PICO 1	42
Supplemental Content 2.12	GRADE Evidence Profile for PICO 1	43–49
Supplemental Content 2.13	PICO 1: Evidence-to-Decision (EtD) Framework	50–82
Supplemental Content 2.14	Stress Ulcer Prophylaxis (SUP) in ICU Subpopulations	83–85
Supplemental Content 2.15	AMSTAR-2 Assessment of Systematic Reviews for PICO 2	86–87
Supplemental Content 2.16	Characteristics of Systematic Reviews Evaluated for PICO 2	88
Supplemental Content 2.17	Updated Forest Plots for PICO 2	89–92
Supplemental Content 2.18	GRADE Evidence Profile for PICO 2	93–94
Supplemental Content 2.19	PICO 2: Evidence-to-Decision (EtD) Framework	95–111
Supplemental Content 2.20	AMSTAR-2 Assessment of Systematic Reviews for PICO 3	112–113
Supplemental Content 2.21	Characteristics of Systematic Reviews Evaluated for PICO 3	114–115
Supplemental Content 2.22	PICO 3: Evidence Profiles from Wang 2020	116–126
Supplemental Content 2.23	NMA from Source Guideline for PICO 3	127–131
Supplemental Content 2.24	PICO 3: Evidence-to-Decision (EtD) Framework	132–158
Supplemental Content 2.25	Characteristics of Studies Identified for PICOs 4 and 5	159–160
Supplemental Content 2.26	Risk of Bias Assessment Table (The Risk Of Bias In Non-Randomized Studies – of Interventions, Version 2 [ROBINS-I V2]) for PICOs 4 and 5	161–162
Supplemental Content 2.27	GRADE Evidence Profile for PICOs 4 and 5	163–167
Supplemental Content 2.28	PICOs 4 and 5: Evidence-to-Decision (EtD) Frameworks	168–189
Supplemental Content 2.29	Research Priorities for Future SUP Studies in Critically Ill Adults	190–193
References		194–198

Supplemental Content 2.1 Selection and Organization of Committee Members

This joint guideline was collaboratively developed by the SCCS and SSAI, in partnership with *Acta Anaesthesiologica Scandinavica*. Discussions were initiated by teleconference to review the relevance and potential application of the Rapid Practice Guideline for the Scandinavian Society and Nordic practitioners, determine the feasibility of developing a joint guideline to enhance the guideline's credibility, enrich the guideline with diverse insights, and ensure its applicability across different regions. We followed the Guidelines International Network (GIN) guidance for rapid recommendation development, the collaboration toolkit developed by GIN Guideline Collaborations Working Group, and the International Glossary for Clinical Guidelines Collaboration [1].

Steering committee	Affiliation	Region	Role
**Marwa Amer (MA)**	Critical Care Clinical Pharmacy Consultant<br>King Faisal Specialist Hospital & Research Center<br>Adjunct Assistant Professor- Alfaisal University-College of Medicine<br>Deputy chair, Saudi Critical Care Society Guideline Chapter	Riyadh, Saudi Arabia	Steering committee, Chair/Primary methodologist for SCCS, SSAI Adolopment guideline
Waleed Alhazzani (WA)	Director of Scientific Research Center<br>General Directorate of Armed Forces Health Services, Saudi Arabia<br>Honorary Professor, Department of Critical Care, King Saud University, Saudi Arabia<br>Chair, Saudi Critical Care Society Guideline Chapter	Riyadh, Saudi Arabia	Steering committee, Key contributor in SCCM stress ulcer prophylaxis guideline
Morten Hylander Møller (MHM)	Intensive Care, Copenhagen University Hospital — Rigshospitalet, Copenhagen, Denmark	Copenhagen, Denmark	Steering committee, Key contributor in SCCM stress ulcer prophylaxis guideline
Fayez Alshamsi (FA)	Department of Internal<br>Medicine, College of Medicine and Health Sciences, United Arab Emirates<br>University, Alain, United Arab Emirates	United Arab Emirates	Secondary methodologist for SCCS, SSAI Adolopment guideline

Name	Professional Background	Role
Ali AL Bshabshe	Critical Care	Panel Member
Ahmed Aljedai	Health Policy Expert	Panel Member
Anders Granholm	Intensive Care, Copenhagen University Hospital — Rigshospitalet, Copenhagen, Denmark	Panel Member/ Key contributor in SCCM

Name	Professional Background	Role
		stress ulcer prophylaxis guideline
Amr Arafat	Cardiothoracic Surgery	Panel Member
Awad Al-Omari	Critical Care	Panel Member
Fawzia Alkhaleedi	Critical Car Nurse	Panel Member
Faisal A. Al-Suwaidan	Neurology - Critical Care – Neurocritical, Saudi Critical Care Society Executive Board Member. Neurology Clinical Lead – Ministry of Health - Saudi Arabia	Panel Member
Haifa Algethamy	Critical Care- Neurocritical	Panel Member
Haifa Al-Otaibi	Family Medicine- Master of Public Health in Epidemiology and Biostatistics	Panel Member
Shadan AlMuhaidib	Clinical Laboratory Sciences and Public Health Research	Panel Member
Hasan M. Al-Dorzi	Critical Care	Panel Member
Klaus T. Olkkola	Anaesthesiology, Intensive Care and Pain Medicine, University of Helsinki and Helsinki University Hospital, Helsinki, Finland	Panel Member
Majid Almadi	Medicine and Gastroenterology	Panel Member
Maija-Liisa Kalliomäki	Department of Anaesthesia, Tampere University Hospital, Tampere, Finland	Panel Member
Ville Jalkanen	Intensivists, Finland	Panel Member
Marius Rehn	Prehospital Services, Air Ambulance Department, Oslo University Hospital, Oslo, Norway	Panel Member
Martin Ingi Sigurðsson	Anaesthesia and Intensive Care Medicine, Landspitali—The National University Hospital of Iceland, Iceland	Panel Member
Michelle S Chew	Anaesthesia and Intensive Care, Biomedical and Clinical Sciences, Linköping University, Linköping, Sweden	Panel Member
Mohammed Alshahrani	Critical Care, Emergency Medicine	Panel Member

Name	Professional Background	Role
Morten Hylander Møller	Intensive Care, Copenhagen University Hospital — Rigshospitalet, Copenhagen, Denmark	Panel Member
Namareq Aldardeer	Critical Care, Clinical Pharmacy	Panel Member
Rakan Alqahtani	Critical Care	Panel Member
Yaseen Arabi	Critical Care	Panel Member
Abdullah M Alhammad	Critical Care, Clinical Pharmacy	Panel Member
Abdulrahman Al-Fares	Department of Anesthesia, Critical Care Medicine and Pain Medicine, Al-Amiri Hospital, Minister of Health, Kuwait City, Kuwait; Kuwait Extracorporeal Life Support Program, Al-Amiri Center for Advance Respiratory and Cardiac Failure, Ministry of Health, Kuwait City, Kuwait	Representative for Kuwait Anesthesia and Critical Care Society Endorsement

Supplemental Content 2.2 Conflict of Interest Management

The COIs included financial, intellectual, and personal aspects. The Steering Committee reviewed all disclosures and adjudicated any potential conflicts before initiating the guideline process and panel voting. Direct financial and industry-related COIs were not permitted. Intellectual COI was defined as leading clinical research directly relevant to the given recommendation or topic. Several panel members (WA, MHM, AG, and YA) were co-authors on major randomized trials in stress ulcer prophylaxis (e.g., the SUP-ICU and REVISE trials) as well as on the 2024 SCCM and ASHP "Guideline for the Prevention of Stress-Related Gastrointestinal Bleeding in Critically Ill Adults." None of the panelists disclosed any financial COIs. No other disclosures were directly related to the SUP PICO questions that necessitated abstention from recommendation voting.

**Panel Member**	**COI link**
Faisal Al-Suwaidan	https://gdt.gradepro.org/forms/#who-coi-preview/p_be8e13be-a115-5271-9e0b-d85ae7682fcd_bec5725a-a389-4aed-92b6-ae8f233adb5f_da9e4959-ac89-432a-a84e-0d197802ea99_bbbea100-83e7-4d0c-83b4-6b2042e4ee17/sections
Mohammed Alshahrani	https://gdt.gradepro.org/forms/#who-coi- https://gdt.gradepro.org/forms/#who-coi-preview/p_be8e13be-a115-5271-9e0b-d85ae7682fcd_bec5725a-a389-4aed-92b6-ae8f233adb5f_b4c5988c-59cb-4f0d-88e9-ca55c96e5fed_d9f88bb5-e81f-45db-8e3d-2248a193cbd2/sections/3
Ali Bshabshe	https://gdt.gradepro.org/forms/#who-coi-preview/p_be8e13be-a115-5271-9e0b-d85ae7682fcd_bec5725a-a389-4aed-92b6-ae8f233adb5f_756d2c88-536b-4ef6-a3f9-9ce44caf5a11_24db64a8-acc6-478b-b1f0-34d2af6897c7/sections
Yaseen Arabi	https://gdt.gradepro.org/forms/#who-coi-preview/p_be8e13be-a115-5271-9e0b-d85ae7682fcd_bec5725a-a389-4aed-92b6-ae8f233adb5f_5f72eaf2-e3c3-4c73-9bce-bd7c7f4fc016_46e0989e-74a0-4de9-92a0-c94ab1efdc74/sections
waleed.al-hazzani	https://gdt.gradepro.org/forms/#who-coi-preview/p_be8e13be-a115-5271-9e0b-d85ae7682fcd_bec5725a-a389-4aed-92b6-ae8f233adb5f_42531be5-49c7-4b78-84b4-53fcafe57a64_31e0e55a-9377-4a26-a40f-398bf2aadaf8/sections
Hasan Al-Dorzi	https://gdt.gradepro.org/forms/#who-coi-preview/p_be8e13be-a115-5271-9e0b-d85ae7682fcd_bec5725a-a389-4aed-92b6-ae8f233adb5f_d0615a3e-9d93-4064-afbf-0127ab4e99df_1d2559b4-384a-4d74-a2c7-6296ff26a014/sections
Abdulrahman Alfares	https://gdt.gradepro.org/forms/#who-coi-preview/p_be8e13be-a115-5271-9e0b-d85ae7682fcd_bec5725a-a389-4aed-92b6-ae8f233adb5f_84abe960-32b5-42a9-b0f7-a94c0de645e7_874aac81-6585-4355-8bf7-694eaa6cf12b/sections
Shadan Al-Muhaidib	https://gdt.gradepro.org/forms/#who-coi-preview/p_be8e13be-a115-5271-9e0b-d85ae7682fcd_bec5725a-a389-4aed-92b6-ae8f233adb5f_2743bd8b-659b-425c-b4af-80cfd0f08c02_44578918-67aa-4ddb-b870-b4bb384c175c/sections/2
Michelle Chew	https://gdt.gradepro.org/forms/#who-coi-preview/p_be8e13be-a115-5271-9e0b-d85ae7682fcd_bec5725a-a389-4aed-92b6-ae8f233adb5f_94d64179-eb81-4f66-930e-f2962effa3f0_f0d3c93b-9c7f-4e39-b33d-c079c8d8e6a6/sections

Morten Hylander	https://gdt.gradepro.org/forms/#who-coi-preview/p_be8e13be-a115-5271-9e0b-d85ae7682fcd_bec5725a-a389-4aed-92b6-ae8f233adb5f_9d159e17-8628-441a-8dcf-dec51b8b0626_42ff8648-173e-4dec-a485-52df74e2608a/sections
Amr Arafat	https://gdt.gradepro.org/forms/#who-coi-preview/p_be8e13be-a115-5271-9e0b-d85ae7682fcd_bec5725a-a389-4aed-92b6-ae8f233adb5f_8d0f6c8a-2781-49cb-be77-53c557d99e67_31497bfb-7d1f-4a09-a609-216e08b323fa/sections
Maija-Liisa Kalliomaki	https://gdt.gradepro.org/forms/#who-coi-preview/p_be8e13be-a115-5271-9e0b-d85ae7682fcd_bec5725a-a389-4aed-92b6-ae8f233adb5f_1f251326-5a64-439c-a7b0-29f32a57f4e9_684f5fda-cd15-4198-a2d3-1fed33c100c2/sections
Anders Granholm	https://gdt.gradepro.org/forms/#who-coi-preview/p_be8e13be-a115-5271-9e0b-d85ae7682fcd_bec5725a-a389-4aed-92b6-ae8f233adb5f_852ac4b7-6ece-4f16-93fc-8a6dbfe2b875_53b31623-1171-4c39-9034-9b131f1e38b5/sections/1
Namareq Aldardeer	https://gdt.gradepro.org/forms/#who-coi-preview/p_be8e13be-a115-5271-9e0b-d85ae7682fcd_bec5725a-a389-4aed-92b6-ae8f233adb5f_e7b3cb7f-d716-480e-bb59-2b331584f0f6_4a2257b7-12ed-4756-92ff-1f710720b3ab/sections
Haifa Algethamy	https://gdt.gradepro.org/forms/#who-coi-preview/p_be8e13be-a115-5271-9e0b-d85ae7682fcd_bec5725a-a389-4aed-92b6-ae8f233adb5f_4696d963-b780-41f5-a219-9507189b65bf_0ced916a-de80-4af9-9c86-db4c63e545be/sections
Abdullah Alhammad	https://gdt.gradepro.org/forms/#who-coi-preview/p_be8e13be-a115-5271-9e0b-d85ae7682fcd_bec5725a-a389-4aed-92b6-ae8f233adb5f_5d98500b-4854-4abd-9703-0edb1d752038_38d5d971-3bb7-4ddd-b292-0c015224c49f/sections
Klaus Olkkola	https://gdt.gradepro.org/forms/#who-coi-preview/p_be8e13be-a115-5271-9e0b-d85ae7682fcd_bec5725a-a389-4aed-92b6-ae8f233adb5f_28e6e799-6280-4d19-a996-f563eacdf24e_3af7aa7f-43a5-4997-8e1a-7c6d9f04332e/sections
Haifa Al-Otaibi	https://gdt.gradepro.org/forms/#who-coi-preview/p_be8e13be-a115-5271-9e0b-d85ae7682fcd_bec5725a-a389-4aed-92b6-ae8f233adb5f_00185ab4-2734-4a65-b6dd-e7c256d37568_b8c0696a-8363-4560-82da-5c6196b464d6/sections
Marius Rehn	https://gdt.gradepro.org/forms/#who-coi-preview/p_be8e13be-a115-5271-9e0b-d85ae7682fcd_bec5725a-a389-4aed-92b6-ae8f233adb5f_bd6c4241-c754-4eef-977c-521c61e6e5c7_f607575c-d304-4ba4-b952-4744f55da910/sections
Fawziah alkhaldi	https://gdt.gradepro.org/forms/#who-coi-preview/p_be8e13be-a115-5271-9e0b-d85ae7682fcd_bec5725a-a389-4aed-92b6-ae8f233adb5f_4a436cbe-e987-43e3-8df6-cecec4564a93_c1ad7d81-f869-4ed2-ae63-e183d9deb54f/sections
Martin Sigurðsson	https://gdt.gradepro.org/forms/#who-coi-preview/p_be8e13be-a115-5271-9e0b-d85ae7682fcd_bec5725a-a389-4aed-92b6-ae8f233adb5f_690cea00-2ffb-419f-8bbf-e918c2e64368_9e34688d-0483-4896-b998-e591906b162d/sections
Ahmed Aljedai	https://gdt.gradepro.org/forms/#who-coi-preview/p_be8e13be-a115-5271-9e0b-d85ae7682fcd_bec5725a-a389-4aed-92b6-ae8f233adb5f_793e84cd-7890-4776-920f-387f90b62a4a_c09ac1d3-45f6-462e-8049-fe2c6bfc486c/sections
Majid Almadi	https://gdt.gradepro.org/forms/#who-coi-preview/p_be8e13be-a115-5271-9e0b-d85ae7682fcd_bec5725a-a389-4aed-92b6-ae8f233adb5f_94936976-9d12-499c-abc8-e3b79fa74686_af327ed7-06f8-4ad6-9a28-5c4473216ee1/sections

Rakan Alqahtani	https://gdt.gradepro.org/forms/#who-coi-preview/p_be8e13be-a115-5271-9e0b-d85ae7682fcd_bec5725a-a389-4aed-92b6-ae8f233adb5f_4e977797-b1ee-44a4-8632-dd6240ffe42f_594c63ef-bcae-4e47-9841-f36df0de795b/sections
Ville Jalkanen	https://gdt.gradepro.org/forms/#who-coi-preview/p_be8e13be-a115-5271-9e0b-d85ae7682fcd_bec5725a-a389-4aed-92b6-ae8f233adb5f_3d6fd789-25e2-4212-88a7-fbed878b95e6_1f237e6d-6c61-4fdb-83e8-605106165971/sections

Supplemental Content 2.3 Outcome Prioritization

We identified a list of outcomes considered pertinent to the actionable PICO statements. Using the GRADE approach to outcome prioritization, we sent an electronic survey asking each panelist to rate each of the listed outcomes on a scale of 1 to 9 (1= least important; 9 = critical to decision making). Panel members were asked to rate the importance of each of the listed outcomes from the perspectives of patients. Outcomes with a mean rating of 7 or more were considered critical and were included under each question. Mean scores were then calculated for each outcome and categorized based on the scoring guide presented below. The final outcome ratings are also displayed in the table below the scoring guide. In accordance with GRADE methods, we used terminology that reflects the overall certainty of evidence, employing stronger language for high-certainty evidence and less certain terms ("likely" or "may") for moderate- or low-certainty evidence.

Scoring Guide

SCORES	IMPORTANCE
**1–3**	**Limited Importance**
**4–6**	**Important**
**7–9**	**Critically important**

Outcome	Relevance to SUP in Critically Ill Patients
**Clinically Important UGIB**	Primary outcome directly targeted by SUP interventions.
**Overt UGIB**	Visible and clinically significant gastrointestinal bleeding.
**Pneumonia/Ventilator-Associated Pneumonia**	Monitored as a potential adverse effect of pharmacologic SUP.
***Clostridioides difficile* Infection**	Risk associated with PPI overuse and critical for SUP safety.
**Gastric pH Level – Not Important**	Biomarker to assess effectiveness of SUP interventions.
**Ventilator-Free Days or Duration of Mechanical Ventilation**	Reflects the impact of SUP on respiratory complications and recovery.
**ICU Length of Stay**	Proxy for resource utilization and overall patient recovery.

**Hospital Length of Stay**	Broader measure of resource use and recovery duration.
**Renal Failure Requiring Renal Replacement Therapy**	Secondary outcome relevant for critically ill patients.
**Acute Kidney Failure**	Complication that may influence SUP strategies.
**Diarrhea**	Common side effect of pharmacologic agents used in SUP.
**Delirium**	Relevant to the overall management of critically ill patients.
**Thrombocytopenia**	Possible adverse event associated with certain interventions.
**Any UGIB**	Broader cases of UGIB that may provide trends for analysis.
**Mortality**	Importance of survival during and after ICU stay.

Gastrointestinal Bleeding Definition [2]

Gastrointestinal (GI) bleeding is defined as the loss of blood from any location within the gastrointestinal tract. It is identified by the macroscopic presence of blood in vomited fluids, gastric aspirate, or stools and/or through evidence of active bleeding detected via CT scan, angiography, or endoscopy.

Clinically Important GI Bleeding

Clinically important GI bleeding is characterized by overt bleeding accompanied by one or more of the following criteria:

1. Significant hemodynamic changes.
2. Requirement for blood transfusions (two or more units of blood within a 24-h period).
3. Notable reductions in hemoglobin levels (a decrease of at least 2 g/dL).
4. Necessity for surgical, endoscopic, or endovascular intervention to control the bleeding.

Results of Outcomes Prioritization Survey

Outcome	Median	Mean	SD	Importance
Clinically Important UGIB	9	8.08	1.14	Critically Important
Overt UGIB	9	7.54	1.80	Critically Important
Pneumonia/VAP	8	7.08	1.19	Critically Important
C. difficile	6	5.92	1. 96	Critically Important *

Gastric pH Level	3	3.77	2.27	Limited Importance
Ventilator-Free Days	6	6.46	1.21	Important
ICU Length of Stay	6	5.77	0.73	Important
Hospital Length of Stay	5	5.38	1.02	Important
Renal Failure Requiring Renal Replacement Therapy	7	6.47	2.13	Important
Acute Kidney Failure	6	6.27	1.97	Important
Diarrhea	5	4.87	1.75	Important
Delirium	6	5.80	1.92	Important
Thrombocytopenia	5	4.73	1.12	Important
Any UGIB	6	5.93	1.69	Important
Mortality	6	6.31	1.83	Critically Important *

* After reviewing the individual survey scores, the panel conducted a calibration discussion to reconcile differences in outcome ratings, as recommended by GRADE-ADOLOPMENT methodology. Although *C. difficile* and mortality initially received a median score of 6 ("important"), the panel reached consensus to classify them as critical for decision-making, reflecting patient-centered priorities and alignment with other international critical-care guidelines and GRADE guidance 39: Using GRADE-ADOLOPMENT to adopt, adapt or create contextualized recommendations J Clin Epidemiol. 2024;174:111494. doi: 10.1016/j.jclinepi.2024.111494.

Panel comments:
Length of stay-based outcomes have been rated low; these outcomes are very problematic in the ICU setting. Days alive without X-type outcome should always be preferred. MID 1 day for days-based outcomes, 2% for key binary outcomes (CIGIB, mortality, delirium, etc)
Note that there are no PROMs or PREMs. Perhaps nausea? Abdominal pain?

Supplemental Content 2.4 GRADE Methodology

1.Certainty in the Evidence

We applied well-established GRADE approaches to determine the overall certainty of evidence for each outcome separately. A clinician-methodologist then generated an Evidence Profile using the GDT software (www.GRADEPRO.com). In the GRADE approach, randomized controlled trials are initially considered to yield "high" certainty evidence, which may then be downgraded if there are concerns around one or more of the following domains: (1) risk of bias, (2) inconsistency, (3) indirectness of the evidence, (4) imprecision, and (5) "other" factors, which include publication bias, presence of a dose-response relationship, magnitude of the effect, and assessment of the effect of plausible residual confounding or bias. Non-randomized studies are initially considered to yield "low" certainty evidence, which may then be upgraded or further downgraded based on the assessment of the same 5 domains. The purpose of assessing these domains is to produce an overall rating of the certainty of evidence to reflect the confidence in the pooled effect estimate for the chosen outcomes and its likelihood of proximity to the "true effect" of an intervention.

The certainty of the evidence for each outcome was then categorized as "high," "moderate," "low," or "very low": In accordance with the GRADE methods, we used terminology that reflects the overall certainty of evidence, employing stronger language for high-certainty evidence and less certain terms ("likely" or "may") for moderate- or low-certainty evidence.

**Certainty Level**	**Description**
⨁⨁⨁⨁<br>High	We are very confident that the true effect lies close to that of the estimate of the effect.
⨁⨁⨁◯<br>Moderate	We are moderately confident in the effect estimate: The true effect is likely to be close to the estimate of that effect, but there is a possibility that it is substantially different.
⨁⨁◯◯<br>Low	Our confidence in the effect estimate is limited: The true effect may be substantially different from the estimate of the effect.
⨁◯◯◯<br>Very Low	We have very little confidence in the effect estimate: The true effect is likely to be substantially different from the estimate of effect.

Addressing Publication Bias:

Publication bias is a systematic under-estimation or an over-estimation of the underlying beneficial or harmful effect due to the **selective publication of studies**. Confidence in the combined estimates of effects from a systematic review can be reduced when publication bias is suspected, even when the included studies themselves have a low risk of bias.

Some systems for assessing the quality of the body of evidence use the term “reporting bias” with 2 subcategories: selective outcome reporting and publication bias. However, GRADE considers *selective outcome reporting* under *risk of bias* (study limitations) since it can be addressed in single studies. In contrast, when an entire study remains unpublished (unreported), one can assess the likelihood of *publication bias* by looking at a group of studies. Currently, GRADE follows the Cochrane Collaboration’s approach and consider *selective outcome reporting* as an issue in risk of bias in individual studies. [3]

Larger studies and those with statistically significant findings are more likely to be published than smaller studies or those yielding inconclusive, neutral, or negative results. This trend can introduce publication bias into systematic reviews. Various methods have been proposed to detect publication bias, such as visual inspection of funnel plots and statistical tests like Egger's test and Begg's test, which apply to all types of outcomes, and Harbord's test, which is specific to binary outcomes. [4,5] However, these methods come with several limitations and must be used with caution. These include their low power to detect true asymmetry, particularly in cases of significant heterogeneity or when only a few studies are involved, the inconsistent agreement among different publication bias tests, and the potential for incorrect application to various outcome types, which could yield misleading conclusions. Furthermore, if fewer than ten studies are included, effectively assessing publication bias becomes challenging. Consequently, GRADE recommends adjusting the certainty of evidence for potential publication bias downward by no more than one level.

Possible sources of publication bias throughout the publication process<br>Adapted from the handbook for grading the quality of evidence and the strength of recommendations using the GRADE approach	
Phases of research publication	Actions contributing to or resulting in bias.
Preliminary and pilot studies	Small studies more likely to be “negative” (e.g. those with discarded or failed hypotheses) remain unpublished; companies classify some as proprietary information.
Report completion	Authors decide that reporting a “negative” study is uninteresting and do not invest the time and effort required for submission.

Journal selection	Authors decide to submit the “negative” report to a nonindexed, non-English, or limited-circulation journal.
Editorial consideration	Editor decides that the “negative” study does not warrant peer review and rejects manuscript.
Peer review	Peer reviewers conclude that the “negative” study does not contribute to the field and recommend rejecting the manuscript. Author gives up or moves to lower impact journal. Publication delayed.
Author revision and resubmission	Author of rejected manuscript decides to forgo the submission of the “negative” study or to submit it again later to another journal (see “journal selection” above).
Report publication	Journal delays the publication of the “negative” study.<br>Proprietary interests lead to report getting submitted to, and accepted by, different journals.

Studies with **small sample sizes** are more likely to remain unpublished or ignored. Discrepancies between results of meta-analyses of small studies and subsequent large trials may occur as often as 20% of the time, and publication bias may be a major contributor to such discrepancies. Therefore, one should suspect publication bias when published evidence is limited to a small number of small trials. This is especially true if many of these small studies show benefits of certain intervention.

When to downgrade the quality of evidence because of suspicion of publication bias

Guideline panels and authors of systematic reviews should consider the extent to which they are uncertain about the magnitude of the effect due to selective publication of studies and may downgrade the quality of evidence by one level. Consider:

- Study design (experimental vs. observational)
- Study size (small studies vs. large studies)
- Lag bias (early publication of positive results)
- Search strategy (was it comprehensive?)
- Asymmetry in funnel plot

Application of GRADE guidance on optimizing the integration of evidence from randomized and non-randomized studies in evidence syntheses and health guidelines [6]

- **Use of NRSI (Non-Randomized Studies of Interventions):**
    - Complements RCTs especially if RCTs have moderate to very low certainty.
    - Useful when assessing baseline risks, harm outcomes, or serious/rare adverse events.
- **Assessment Process:**
    - Start with RCTs to establish a baseline body of evidence.
    - Evaluate the Certainty of Evidence (CoE) in both RCTs and NRSI.
    - Determine if NRSI can reduce uncertainty in RCT findings, or vice versa.
- **Decision-Making on Evidence Use:**
    - Decide based on overall judgment and CoE:
        - Use only the evidence with higher CoE.
        - Present both RCTs and NRSI separately (e.g., in subgroups or separate meta-analyses).
        - Present a single pooled estimate if appropriate.
- **Handling Inconsistency:**
    - Avoid combining RCTs and NRSI if one shows significant inconsistency.
    - Use NRSI to explore potential causes of unexplained heterogeneity in RCTs.
    - If no specific causes are identified, keep RCTs and NRSI separate.
- **Addressing Imprecision:**
    - When risks of bias are similar between RCTs and NRSI, assess if NRSI can improve imprecise RCT estimates.
    - Do not pool data if there are additional concerns like inconsistency, indirectness, or publication bias in the evidence.
- **Dealing with Indirectness:**
    - Explore using NRSI for more direct evidence if RCTs are rated down due to indirectness.
    - Use indirect RCT estimates without downgarding for indirectness if they are congruent with NRSI, assuming no other GRADE concerns.
    - Avoid using or pooling RCTs and NRSI together if effects are incongruent or if there is additional inconsistency, imprecision, or publication bias.

2. Evidence-to-Decision Framework

For each PICO question, panel members held one web-based meetings via Zoom video conferencing platform to review the Evidence Profile, discuss the evidence and various factors that may influence decision-making, and generate a recommendation. The GRADE Evidence-to-Decision (EtD) framework was used to help organize panel discussions during deliberation meetings. The EtD incorporates panel judgment across 12 domains. Cost data were collected using two sources: the Saudi Food Drug Authority (SFDA) for the private sector and the National Unified Procurement Company (NUPCO) for the governmental sector. For other contextual factors (i.e., impact on equity, acceptability, and feasibility), no formal assessment was conducted; instead, the panel relied on their professional knowledge and experience.

**Domain**	**Question**
**Priority of the problem**	Is the problem a priority?
**Desirable effects**	How substantial are the desirable effects?
**Undesirable effects**	How substantial are the undesirable effects?
**Certainty of evidence**	What is the overall certainty of the evidence of effects?
**Values**	Is there important uncertainty or variability in how much people value the main outcome?
**Balance of effects**	Does the balance between desirable and undesirable effects favor the intervention or the comparison?
**Resources required**	How large are the resource requirements (costs)?
**Certainty of evidence of required resources**	What is the certainty of the evidence of resource requirements (costs)?
**Cost effectiveness**	Does cost-effectiveness of the intervention favor the intervention or the comparison?
**Equity**	What would be the impact on health equity?
**Acceptability**	Is the intervention acceptable to key stakeholders?
**Feasibility**	Is the intervention feasible to implement?

3. Recommendation Generation

After reviewing the Evidence Profile and discussing each consideration in the EtD for a PICO question, the panel deliberated and decided on a recommendation direction (for, against, or neutral) and strength (strong vs. conditional). By convention, strong recommendations are phrased as “We recommend…” and conditional recommendations as “We suggest…” **Supplemental Content 7** demonstrates implications of different recommendations to key stakeholders.

Supplemental Content 2.5 Implications of Different Recommendations to Key Stakeholders and the Voting Process

Recommendation	Meaning	Implication to patients	Implications to clinicians	Implications to policymakers
Strong recommendation "We recommend"<br><br>Desirable effects of intervention clearly outweigh undesirable effects or clearly do not	Must do or must avoid	Almost all individuals in this situation would want the recommended intervention, and only a small proportion would not want it	Most individuals should receive the recommended course of action	Can be adapted as policy in most situations, including the use as performance indicators
Conditional recommendation "We suggest"<br>Trade-offs are less certain, either because of low-certainty evidence or because evidence suggests desirable and undesirable effects are closely balanced	Consider doing or consider avoiding	Most individuals in this situation would want the recommended intervention, but many would not	Different choices are likely to be appropriate for different patients, and the recommendation should be tailored to the individual patient's circumstances, including the values and preferences of patients and their families	Policies will likely be variable between regions

Final Voting Process

The methodologists drafted the preliminary recommendation for each PICO question. After all draft recommendations were generated, all panel members were electronically polled to indicate their agreement with each recommendation. Panelists were asked to select from three options: “Agree,” “Disagree,” or “Abstain.” Panel members were given the opportunity to provide comments explaining their selections for each recommendation, and these comments were reviewed and, where appropriate, addressed by the panel leadership.

The final recommendation was completed by the group via conference calls and email. Recommendations were formulated by consensus, and no voting was required at this stage. Consensus was defined as ≥ 75% agreement rate and ≥ 70% response rate. Recommendations that failed to achieve ≥ 80% agreement were sent electronically for an additional round of voting, for up to a maximum of three rounds. If no consensus could not be reached after the third round of voting, the panel would not issue a recommendation. Panelists who did not participate in the voting process could not be listed as authors on the manuscript but were acknowledged as contributors.

Supplemental Content 2.6 Definitions and Criteria for Recommendation Classification and Framework for Contextualization

Classification	Definition	Criteria
**Adopt**	**Use the original recommendation as is.**	**- EtD judgments are appropriate to the local context<br>- No new evidence impacts the balance of effects<br>- Panel agrees with original conclusion**
**Adapt**	**Modify the recommendation to suit the local context.**	**- Some EtD judgments differ (e.g., costs, feasibility)<br>- Local evidence requires contextualization<br>- Recommendation wording or strength may be modified**
De novo	**Create a new recommendation from scratch using the full GRADE process.**	**- No relevant source recommendation exists<br>- New question identified by the panel<br>- Local health system factors or population characteristics require an entirely new evidence review and recommendation<br>- Newly developed recommendation addressed a regional need: "high-risk populations specific to Saudi Arabia and Nordic countries." The original EtD framework requires substantial changes, making the process conceptually equivalent to creating a new recommendation using GRADE methods**

This guideline was developed using the **GRADE-ADOLOPMENT methodology**, in accordance with the WHO Handbook *"Strengthening countries' capacities to adopt and adapt evidence-based guidelines."* The contextualization process was structured to ensure that each recommendation reflects the best available evidence while being feasible, acceptable, and relevant to health systems in Saudi Arabia and Nordic countries. [7]

To enhance transparency and methodological rigor, the development process incorporated the following WHO-recommended steps:

1. **Source Guideline Appraisal**: The source guideline (SCCM-ASHP) was assessed using AGREE II to confirm its credibility, relevance, and methodological robustness.
2. **Panel Formation**: A multidisciplinary panel was established with regional representation and balanced expertise across clinical, pharmacy, and methodological domains.
3. **Conflict of Interest Management**: All panelists submitted conflict of interest (COI) disclosures, which were reviewed and managed per SSAI/SCCS policies.
4. **PICO Prioritization**: Priority questions were selected based on clinical relevance, resource impact, and evidence gaps.

5. **Outcome Prioritization**: Outcomes were ranked according to importance to patients and clinical decision-making, aligned with GRADE guidance.
6. **Evidence Appraisal and Updating**: Source evidence profiles were assessed with AMSTAR-2; updated systematic reviews were conducted when necessary.
7. **EtD Framework Use**: GRADE Evidence-to-Decision (EtD) frameworks guided the judgment process, incorporating local data on cost, access, feasibility, and equity.
8. **Adoption vs. Adaptation vs. *De Novo* Classification**: Each recommendation was categorized as adopted, adapted, or developed *de novo* based on contextual needs.
9. **Stakeholder Engagement**: While formal patient representation was not available, regional stakeholders including ICU policy leads and formulary directors were consulted.
10. **Implementation Planning**: Recommendations were designed to support integration into ICU workflows, national protocols, and EMR-based decision aids.
11. **Quality Assurance**: The final recommendations underwent internal peer review, with structured voting via Panel Voice to ensure ≥ 80% consensus.
12. **Dissemination and Monitoring (Planned)**: A dissemination strategy includes publication, webinar presentations, and integration into national ICU initiatives. Monitoring indicators include adherence to SUP recommendations and deprescribing rates.

Comparison With Other Guideline Adolopment Efforts

Our use of the GRADE-ADOLOPMENT methodology aligns with internationally established processes seen in diverse settings, including the Czech Republic, Latin America, and the Eastern Mediterranean Region. Similar to the Czech breast cancer screening and spine surgery guidelines, we prioritized a limited number of questions for focused adaptation, balancing methodological rigor with resource constraints. As with the Latin American adolopment of venous thromboembolism guidelines, we maintained alignment with source recommendations when contextual factors (e.g., feasibility, equity, and resource use) were consistent across regions. Our inclusion of region-specific drug formulary data and ICU discharge practices parallels the Saudi adolopment of rheumatoid arthritis guidelines, where adaptations were informed by national treatment norms and access disparities.

Moreover, as in other case studies, we applied structured prioritization (e.g., Delphi-like voting and outcome surveys) and encouraged the use of new local evidence, even when source systematic reviews were recent. This hybrid model—adopting where suitable, adapting based on contextual divergence, and developing *de novo* where gaps exist—was mirrored in other global initiatives and reinforced the flexibility and transferability of the GRADE-ADOLOPMENT model.

These parallels validate our approach and demonstrate the adaptability of this framework across diverse healthcare systems, reinforcing the need for transparent contextual judgment and regional stakeholder engagement to ensure successful implementation.

Supplemental content 2.7 Estimate Baseline Risk for Clinically Important Gastrointestinal Bleeding (CIGIB) and Overt Bleeding

To estimate baseline risk for clinically important gastrointestinal bleeding (CIGIB) and overt bleeding, we applied the same approach used in prior clinical practice guidelines (BMJ 2020; 368 doi: https://doi.org/10.1136/bmj.l6722) [8]. Our approach integrates clinical trial data from SUP-ICU [9] and REVISE [10] (updated the estimates for mechanically ventilated patients), observational studies, and meta-analyses [11] to estimate baseline risks of CIB and overt GIB across different patient risk groups.

Approach to Estimating Baseline Risk of Clinically Important GI Bleeding (CIGIB):

1. **Starting Baseline Risk:**
    - The event rate in the placebo group of the SUP-ICU trial was selected as the baseline due to its size.
    - This trial included patients with at least one hypothesized risk factor for GI bleeding, meaning the estimated risk reflects a higher-risk population.
    - Baseline risk of CIB with any risk factor = 4.2%.
2. **Adjusting for Common Risk Factors:**
    - The most prevalent risk factors in the SUP-ICU trial were:
        - Invasive mechanical ventilation (78.7%)
        - Vasopressors or inotropes (66.7%)
        - Use of anticoagulants (30.3%)
        - Coagulopathy (19.8%)
    - Based on observational studies, the risk ratios (RRs) for these risk factors ranged from 2.5 (septic shock and anticoagulation) to 4.5 (mechanical ventilation and coagulopathy).
    - The median RR of 3.5 (2.5 to 4.5) was used to estimate the baseline risk in patients without these risk factors.

The baseline risk of CIB in patients without any risk factors was estimated by dividing the risk of CIB in patients with risk factors (4.2%) by the relative risk associated with having a risk factor (3.5) identified from individual observational studies . This calculation yields an approximate baseline risk of 1.2% (range: 0.9%–1.7%).

3. **Estimating Risk in Patients with Specific Risk Factors:**

  - Specific relative risks from low-risk-of-bias observational studies were applied.
  - Estimates were derived from a concurrent systematic review and meta-analysis for Granholm A et al. [13]

Table 1: Clinically Important Gastrointestinal Bleeding

Potential Predictor	Relative Effect Estimate (95% CI)	Certainty of Evidence	Baseline Risk per 1000
**No Risk Factor**	-	-	12
**Mechanical Ventilation**	4.09 (0.37–45.67)	Very Low	We used REVISE data
**Coagulopathy**	4.76 (2.62–8.63)	Moderate	57
**Shock**	2.60 (1.25–5.42)	Low	31
**Sepsis**	2.00 (0.74–5.37)	Low	24
**Acute Hepatic Failure**	1.60 (0.70–3.67)	Low	19

**Chronic Liver Disease**	7.64 (3.32–17.58)	Moderate	92
**Acute Kidney Injury**	3.26 (0.78–13.63)	Low	39
**Enteral Nutrition**	0.30 (0.13–0.68)	Moderate	-
**Use of Steroids/Immunosuppression**	1.39 (0.71–2.71)	Low	17
**Use of Anticoagulants**	1.42 (0.65–3.10)	Low	17
**Cancer**	1.36 (0.28–6.54)	Low	16
**Male Sex**	0.85 (0.39–1.87)	Low	10

Table 2: Overt Gastrointestinal Bleeding

**Potential Predictor**	**Relative Effect Estimate (95% CI)**	**Certainty of Evidence**	**Baseline Risk per 1000**
**Mechanical Ventilation**	4.24 (0.43–42.09)	Very Low	We used REVISE data
**Coagulopathy**	4.14 (2.49–6.90)	Moderate	108
**Shock**	2.56 (1.44–4.54)	Low	67
**Sepsis**	2.00 (0.74–5.37)	Low	52

**Acute Hepatic Failure**	3.10 (0.77–12.51)	Moderate	81
**Chronic Liver Disease**	4.51 (2.30–8.85)	Moderate	117
**Acute Kidney Injury**	3.46 (0.78–15.41)	Low	90
**Chronic Renal Failure**	1.94 (0.72–5.24)	Low	40
**Use of Steroids/Immunosuppression**	1.52 (0.83–2.76)	Moderate	-
**Transplants**	1.50 (0.57–3.95)	Low	-
**Use of Anticoagulants**	1.79 (0.93–3.44)	Moderate	47
**Cancer**	0.83 (0.11–6.13)	Very Low	22
**Male Sex**	0.80 (0.44–1.45)	Low	21
**Heart Failure**	1.15 (0.37–3.58)	Low	-
**No Risk Factor**	-	-	26

4. **Accounting for Enteral Nutrition as a Protective Factor:** to be covered in PICO 2
5. **Risk Stratification:**
    - To improve usability, four risk categories were defined:
        - Low risk (0–20 bleeds per 1000 patients) → 12 per 1000
        - Moderate risk (21–40 bleeds per 1000 patients) → 30 per 1000
        - High risk (41–80 bleeds per 1000 patients) → 60 per 1000

    - Highest risk (81–100 bleeds per 1000 patients) → 90 per 1000

Estimating Risk for Overt Gastrointestinal Bleeding (Overt GIB):

- The same approach was applied for overt bleeding.
- Patients in the placebo group of the SUP-ICU trial had an overt bleeding risk of 9.0%.

Updated estimated risks from the **REVISE trial** provided the baseline risk of clinically important bleeding (CIB) and overt bleeding in patients with mechanical ventilation.

Baseline Risk of Clinically Important Gastrointestinal Bleeding (CIGIB) Based on REVISE Trial Data

1. Event Rate in the Placebo Group (Baseline Risk)

- According to the **REVISE trial**, the event rate of **clinically important upper gastrointestinal bleeding (CIGIB)** in the **placebo group** was **3.5% (84/2377 patients)**

2. Adjusting for Common Risk Factors: Invasive Mechanical Ventilation

- The relative risk (RR) for **mechanical ventilation** in relation to clinically important GI bleeding is **4.09 (95% CI: 0.37–45.67)**.
- To estimate the **baseline risk in patients without this risk factor**, we applied the following formula:

$$\text{Baseline Risk without Mechanical Ventilation} = \frac{\text{Baseline Risk with Mechanical Ventilation}}{\text{Relative Risk Increase}}$$

$$= \frac{3.5\%}{4.09} \approx 0.86\% \quad \text{(per 1000 patients: 9 per 1000)}$$

3. Estimating Risk in Patients with Mechanical Ventilation

- Using the **REVISE trial data**, we were able to estimate the **CIGIB risk for patients requiring invasive mechanical ventilation**:

$$\text{Baseline Risk with Mechanical Ventilation} = 3.5\% \quad (35 per 1000 patients)$$

- Given the **updated risk estimates from the REVISE trial**, mechanical ventilation **increases the baseline risk** of:
    - **Clinically important GI bleeding (CIGIB): 4.5%** (**45 per 1000 patients**)
    - **Overt GI bleeding: 5.1%** (**51 per 1000 patients**)

Risk Factor	Baseline Risk of CIB (%)	Baseline Risk of Overt Bleeding (%)
Any Risk Factor (Placebo Arm in REVISE)	3.5%	4.2%
No Risk Factor	$3.5\% \times \frac{1}{3.5}$ = 1.0%	$4.2\% \times \frac{1}{3.5}$ = 1.2%
Mechanical Ventilation (100%)	$1.0\% \times 4.5$ = 4.5%	$1.2\% \times 4.24$ = 5.1%
Vasopressors/Inotropes (71%)	$1.0\% \times 2.5$ = 2.5%	$1.2\% \times 2.56$ = 3.1%

Baseline Risk per 1000 Patients

Risk Factor	Baseline Risk of CIB (per 1000 patients)	Baseline Risk of Overt Bleeding (per 1000 patients)
Any Risk Factor (Placebo Arm in REVISE)	$3.5\% \times 10$ = 35 per 1000	$4.2\% \times 10$ = 42 per 1000
No Risk Factor	$35 \times \frac{1}{3.5}$ = 10 per 1000	$42 \times \frac{1}{3.5}$ = 12 per 1000
Mechanical Ventilation (100%)	10×4.5 = 45 per 1000	12×4.24 = 51 per 1000
Vasopressors/Inotropes (71%)	10×2.5 = 25 per 1000	12×2.56 = 31 per 1000

Relative risk of MV 4.5 (from previous litrature and meta-analysis) [13]

Mechanical ventilation increases the **CIB risk to 4.5%** and **overt bleeding risk to 5.1%** → (**45 per 1000 for CIB**, **51 per 1000 for overt bleeding**).
Vasopressors/inotropes moderately increase the **CIB risk to 2.5%** and **overt bleeding risk to 3.1%** → (**25 per 1000 for CIB**, **31 per 1000 for overt bleeding**).

Supplemental Content 2.8 AMSTAR-2 Assessment of Systematic Reviews for PICO 1

Question		Ying Wang 2020 (1)	Ying Wang 2024 (2)	Ying Wang 2020-updated SRMA (3)	Granholm 2019 (4)
1.	Did the research questions and inclusion criteria for the review include the components of PICO?	Yes	Yes	Yes	Yes
2.	Did the report of the review contain an explicit statement that the review methods were established prior to the conduct of the review and did the report justify any significant deviations from the protocol?	Yes PROSPERO (CRD42019126656)	Yes PROSPERO (CRD4202346169 5)	Yes PROSPERO: CRD42020169989	No PROSPERO registration
3.	Did the review authors explain their selection of the study designs for inclusion in the review?	Yes	Yes	Yes	Yes
4.	Did the review authors use a comprehensive literature search strategy?	Yes	Yes	Yes	Yes
5.	Did the review authors perform study selection in duplicate?	Yes	Yes	Yes	Yes
6.	Did the review authors perform data extraction in duplicate?	Yes	Yes	Yes	Yes
7.	Did the review authors provide a list of excluded studies and justify the exclusions?	No	No	No	No
8.	Did the review authors describe the included studies in adequate detail?	Yes	Yes	Yes	Yes
9.	Did the review authors use a satisfactory technique for assessing the RoB in individual studies that were included in the review?	Yes	Yes	Yes	Yes
10.	Did the review authors report on the sources of funding for the studies included in the review?	No	No	No	No
11.	If meta-analysis was performed, did the review authors use appropriate methods for statistical combination of results?	Yes	Yes	Yes	Yes

12.	If meta-analysis was performed, did the review authors assess the potential impact of RoB in individual studies on the results of the meta-analysis or other evidence synthesis?	Yes	Yes	Yes	Yes
13.	Did the review authors account for RoB in individual studies when interpreting/discussing the results of the review?	Yes	Yes	Yes	Yes
14.	Did the review authors provide a satisfactory explanation for, and discussion of, any heterogeneity observed in the results of the review?	Yes	Yes	Yes	Yes
15.	If quantitative synthesis was performed, did the review authors carry out an adequate investigation of publication bias (small study bias) and discuss its impact on the results of the review?	Yes	Yes	Yes	No<br>Funnel plots were planned but not done due to the low number of studies
16.	Did the review authors report any potential sources of conflict of interest, including any funding they received for conducting the review?	Yes	Yes	Yes	Yes

PICO = patient, intervention, comparator, outcome; RoB = risk of bias

Supplemental Content 2.9 Characteristics of Systematic Reviews Evaluated for PICO 1

Study	Date of Search and Databases Examined	# Trials and # participants included	Population	Comparison# randomized<br>Group 1: SUP<br>Group 2: Comparator	Main findings
Wang Y, 2020 (1)	Up to September 2019; MEDLINE, Embase, CENTRAL, CINAHL, trial registries	72 randomized controlled trials including 12,660 patients	Critically ill adults in intensive care units	**Interventions (Group 1)**: Stress ulcer prophylaxis (SUP)—Proton Pump Inhibitors (PPIs), Histamine-2 Receptor Antagonists (H2RAs), and sucralfate<br>**Comparators (Group 2)**: Placebo or no prophylaxis	See Supplemental Content 9 below
Wang Y, 2024 (2)	September 15, 2023; Medline (Ovid), Embase (Ovid), CENTRAL, CINAHL, Scopus, Web of Science	24 RCTs; 7,172 patients	Critically ill adults in ICU settings	**Group 1:** Proton Pump Inhibitor (PPI) as Stress Ulcer Prophylaxis (SUP).<br>**Group 2:** Placebo or No Prophylaxis	**Clinically Important Gastrointestinal (GI) Bleeding**:<br>High-certainty evidence showed that PPIs **significantly reduced the risk** of clinically important upper GI bleeding compared to placebo or no prophylaxis.<br>• **Relative Risk (RR): 0.51 (95% CI: 0.34 to 0.76)**<br>• **Absolute Risk Reduction**: 18 fewer events per 1,000 patients (95% CI: 25 fewer to 5 fewer)<br>**Mortality**:<br>Low-certainty evidence indicated that PPIs **may have little to no effect on all-cause mortality** in critically ill patients.<br>• **RR: 0.99 (95% CI: 0.93 to 1.05)**<br>• **Absolute Risk Difference**: 3 fewer deaths per 1,000 patients (95% CI: 22 fewer to 15 more)

Study	Date of Search and Databases Examined	# Trials and # participants included	Population	Comparison# randomized<br>Group 1: SUP<br>Group 2: Comparator	Main findings
					• Subgroup analysis (intermediate credibility) suggested a potential **benefit in less severely ill patients** (RR: 0.89) and possible **harm in more severely ill patients** (RR: 1.08), though this was not definitive.<br>**Pneumonia**:<br>Low-certainty evidence showed that PPIs **may have no effect** on the incidence of pneumonia.<br>• **RR: 1.00 (95% CI: 0.92 to 1.09)**<br><br>***Clostridioides difficile*** **Infection**:<br>Low-certainty evidence suggested PPIs **may have little or no effect** on the risk of *C. difficile* infection.<br>• **RR: 1.20 (95% CI: 0.66 to 2.16)**<br><br>**Other Outcomes** (all low-certainty evidence):<br>PPIs may have little to no effect on:<br>• **Duration of mechanical ventilation** (MD: +1.5 days; 95% CI: -0.9 to +3.9)<br>• **Length of ICU stay** (MD: -0.1 days; 95% CI: -0.8 to +0.6)<br>• **Hospital length of stay** (MD: -0.7 days; 95% CI: -2.5 to +1.1)
Ying Wang 2020-updated SRMA	January 2019 – February 2020 (updated); Databases:	74 RCTs; 39,569 participants	Adult critically ill patients in ICU at risk of	**Comparison - Group 1 (SUP)**<br>**Proton Pump Inhibitors (PPIs), Histamine-2**	- Both PPIs (RR 1.03, 95% CrI 0.93–1.14, moderate certainty) and H2RAs (RR 0.98, 0.89–1.08, moderate certainty) probably have little or

Study	Date of Search and Databases Examined	# Trials and # participants included	Population	Comparison# randomized<br>Group 1: SUP<br>Group 2: Comparator	Main findings
	MEDLINE, Embase, Web of Science, CENTRAL, ICTRP, LILACS, clinicaltrials.gov		gastrointestinal bleeding	**Receptor Antagonists (H2RAs), Sucralfate**<br><br>**Comparison - Group 2 (Comparator)**<br>**Placebo or no prophylaxis; alternative pharmacological agent (e.g., PPI vs. H2RA)**	No impact on mortality compared with no prophylaxis.<br>- There may be no important difference in mortality between PPIs and H2RAs (RR 1.05, 0.97–1.14, low certainty), although a possible mortality increase with PPIs cannot be excluded.<br>- Both PPIs (RR 0.46, 0.29–0.66) and H2RAs (RR 0.67, 0.48–0.94) probably reduce clinically important GI bleeding.<br>- The reduction is probably greater with PPIs vs. H2RAs (RR 0.69, 0.45–0.93), especially in high-risk patients.<br>- PPIs (RR 1.08, 0.88–1.45, low certainty) and H2RAs (RR 1.07, 0.85–1.37, low certainty) may have no important impact on pneumonia compared with no prophylaxis.<br>- No important difference among interventions for *C. difficile* infection, ICU/hospital stay, or duration of mechanical ventilation.
Granholm 2019 (4)	March 12–14, 2019; MEDLINE, EMBASE, CENTRAL, ClinicalTrials.gov, WHO ICTRP	8 studies; 116,497 participants This is a meta-analysis of **observational cohort studies**, including secondary	Adult patients in the ICU (critically ill, various ICU types; both general and neurocritical care)	Not an intervention comparison; this SRMA identifies predictors of GI bleeding. However, the majority of patients (in some studies all) received SUP	Identified predictors of clinically important GI bleeding (CIB) and overt GI bleeding in ICU patients.<br>Four studies (including 74,456 patients) assessed potential predictors of CIB, and we meta-analyzed 12 potential predictors from these studies. **Acute kidney injury** (RE 2.38, 95% CI 1.07–5.28, *moderate certainty*) and **male sex** (RE 1.24, 95% CI 1.03–1.50, *low certainty*) were

Study	Date of Search and Databases Examined	# Trials and # participants included	Population	Comparison# randomized<br>Group 1: SUP<br>Group 2: Comparator	Main findings
		analyses of RCT cohorts		**Group 1 (SUP):** Most participants received SUP (e.g., PPIs or H2RAs); 3 studies had 100% receiving SUP<br><br>**Group 2 (Comparator):** Not applicable (no direct comparator group; focus was on predictors, not intervention efficacy)	associated with increased incidence of CIB.<br>After excluding high risk of bias studies, **coagulopathy** (RE 4.76, 95% CI 2.62–8.63, *moderate certainty*), **shock** (RE 2.60, 95% CI 1.25–5.42, *low certainty*), and **chronic liver disease** (RE 7.64, 95% CI 3.32–17.58, *moderate certainty*) were associated with increased incidence of CIB.<br>The effect of **mechanical ventilation** on CIB was unclear (RE 1.93, 0.57–6.50, *very low certainty*).<br>Other **predictors of overt GI bleeding** (moderate certainty) included **coagulopathy, sepsis, acute kidney injury, chronic liver disease,** and **acute hepatic failure**.<br>**Pooled estimate (all studies)**: Relative Effect (RE) 0.63; 95% CI 0.17–2.37 → suggests a **potential protective effect**, but the confidence interval includes both benefit and no effect.<br>**Certainty of evidence**: **Very low**<br>**One study not at high risk of bias** (Cook et al. [19]) found a significant protective association (RE 0.30; 95% CI 0.13–0.68), rated as **moderate certainty** in the subgroup of moderate–low RoB studies.

Supplemental Content 2.10 GRADE Summary of Findings for Wang 2020 For PICO 1

GRADE Summary of Findings for Clinically Important Gastrointestinal Bleeding (CIB)

**Comparison**	**Study results (95% CI)**	**Baseline risk (per 1000)**	**Absolute difference (95% CI) (per 1000)**	**Certainty in effect estimates**	**Plain text summary**
**PPIs vs. Placebo**	RR 0.46 (0.29 to 0.66)	Low risk: 12	–6 (–9 to –4)	Moderate[a]	PPIs likely reduce CIB by less than the amount most people would need to choose a PPI
		Moderate risk: 30	–16 (–21 to –10)	Low[a,b]	PPIs may reduce CIB by less than the amount most people would need to choose a PPI
		High risk: 60	–32 (–43 to –20)	Moderate[a]	PPIs likely reduce CIB
		Highest risk: 90	–49 (–64 to –31)	Moderate[a]	PPIs likely reduce CIB
**H2RAs vs. Placebo**	RR 0.67 (0.48 to 0.94)	Low risk: 12	–4 (–6 to –1)	Moderate[a]	H2RAs likely reduce CIB by less than the amount most people would need to choose an H2RA
		Moderate risk: 30	–10 (–16 to –2)	Moderate[a]	H2RAs likely reduce CIB by less than the amount most people would need to choose an H2RA
		High risk: 60	–20 (–31 to –4)	Moderate[b]	H2RAs likely reduce CIB
		Highest risk: 90	–30 (–47 to –5)	Moderate[b]	H2RAs likely reduce CIB
**Sucralfate vs. Placebo**	RR 0.82 (0.53 to 1.29)	Low risk: 12	–2 (–6 to 3)	Low[a,d]	Sucralfate may not have an important effect
		Moderate risk: 30	–5 (–14 to 9)	Low[a,d]	Sucralfate may not have an important effect
		High risk: 60	–11 (–28 to 17)	Low[a,d]	Sucralfate may not have an important effect
		Highest risk: 90	–16 (–42 to 26)	Very low[a,d]	Whether there is an important difference or not is very uncertain

PPIs = proton pump inhibitors; H2RAs = histamine-2 receptor antagonists; CIB = clinically important bleeding; RR = risk ratio

[a] Rated down due to uncertainty in baseline risk for some risk factors

[b] Rated down for imprecision

[c] Rated down for the differences in results from different analyses/models as well as the uncertainty in baseline risk

[d] Rated down for risk of bias

GRADE Summary of Findings for Overt Gastrointestinal Bleeding

Comparison	Study results (95% CI) and measurements	Absolute effect estimates (per 1000)		Absolute difference (95% CI) (per 1000)	Certainty in effect estimates	Plain text summary
PPIs vs. placebo	RR 0.50 (0.31 to 0.72)	Low risk	Placebo: 26	–13 (–18 to –7)	Moderate[a]	PPIs likely reduce overt bleeding
		Moderate risk	75	–37 (–52 to –21)	Moderate[a]	PPIs likely reduce overt bleeding
		High risk	125	–62 (–86 to –35)	Moderate[a]	PPIs likely reduce overt bleeding
		Highest risk	190	–95 (–131 to –53)	Moderate[a]	PPIs likely reduce overt bleeding
H2RAs vs. placebo	RR 0.66 (0.48 to 0.89)	Low risk	Placebo: 26	–9 (–14 to –3)	Moderate[a]	H2RAs likely reduce overt bleeding
		Moderate risk	75	–25 (–39 to –8)	Moderate[a]	H2RAs likely reduce overt bleeding
		High risk	125	–42 (–65 to –14)	Moderate[a]	H2RAs likely reduce overt bleeding
		Highest risk	190	–65 (–99 to –21)	Moderate[a]	H2RAs likely reduce overt bleeding
Sucralfate vs. placebo	RR 1.00 (0.61 to 1.68)	Low risk	Placebo: 26	0 (–10 to 18)	Moderate[b]	Sucralfate likely has no impact on overt bleeding
		Moderate risk	75	0 (–29 to 51)	Moderate[b]	Sucralfate likely has no impact on overt bleeding
		High risk	125	0 (–49 to 85)	Low[c]	Sucralfate may have no impact on overt bleeding
		Highest risk	190	0 (–74 to 129)	Low[c]	Sucralfate may have no impact on overt bleeding

CI = confidence interval; GI = gastrointestinal; PPIs = proton pump inhibitors; RR = risk ratio; H2RAs = histamine-2 receptor antagonists

[a] Rated down for the differences in results from different analyses as well as the uncertainty in baseline risk

[b] Rated down for imprecision

[c] Rated down 2 levels for imprecision

GRADE Summary of Findings for Length of ICU Stay – Complete PEPTIC Analysis

Comparison	Direct estimate (95% CrI); Certainty of evidence[a]	Indirect estimate (95% CrI); Certainty of evidence[a]	Network estimate (95% CrI); Certainty of evidence[b]	Baseline risk (days)	Absolute difference (95% CrI) (days)
PPIs vs. placebo	0.93 (0.81 to 1.08); High	1.02 (0.83 to 1.24); Moderate[c]	0.96 (0.86 to 1.08); Moderate[d]	Placebo: 7.7[e]	-0.3 (-1.1 to 0.6)
H2RAs vs. placebo	1.09 (0.85 to 1.40); Moderate[c]	0.92 (0.80 to 1.06); Moderate[c]	0.96 (0.85 to 1.09); Low[c,d]	Placebo: 7.7[e]	-0.3 (-1.2 to 0.7)
Sucralfate vs. placebo	0.89 (0.70 to 1.16); Moderate[c]	0.95 (0.80 to 1.12); Moderate[c]	0.93 (0.81 to 1.08); Low[c,d]	Placebo: 7.7[e]	-0.5 (-1.5 to 0.6)

CrI = credible interval; PPIs = proton pump inhibitors; H2RAs = histamine-2 receptor antagonists

[a] We did not consider imprecision when rating for direct and indirect estimates because they were only used to inform the network estimates which we believed were the best estimates

[b] Higher of direct or indirect confidence (without consider imprecision), followed by consideration of imprecision and incoherence

[c] Rated down for risk of bias

[d] Rated down for imprecision

[e] Baseline risk comes from median of the placebo group in the included studies

GRADE Summary of Findings for Duration of Mechanical Ventilation – Complete PEPTIC Analysis

Comparison	Direct estimate (95% CrI); Certainty of evidence[a]	Indirect estimate (95% CrI); Certainty of evidence[a]	Network estimate (95% CrI); Certainty of evidence[b]	Baseline risk (days)	Absolute difference (95% CrI) (days)
PPIs vs. placebo	1.01 (0.84 to 1.22); High	0.94 (0.79 to 1.14); Moderate[c]	0.97 (0.86 to 1.11); Moderate[d]	Placebo: 10.2[e]	-0.3 (-1.4 to 1.1)
H2RAs vs. placebo	0.90 (0.74 to 1.11); Moderate[c]	0.99 (0.84 to 1.17); Moderate[c]	0.95 (0.84 to 1.08); Low[c,d]	Placebo: 10.2[e]	-0.5 (-1.6 to 0.8)
Sucralfate vs. placebo	1.02 (0.80 to 1.33); High	0.99 (0.83 to 1.17); Moderate[c]	1.00 (0.87 to 1.14); Low[c,d,f]	Placebo: 10.2[e]	0 (-1.3 to 1.4)

CrI = credible interval; PPIs = proton pump inhibitors; H2RAs = histamine-2 receptor antagonists

[a] We did not consider imprecision when rating for direct and indirect estimates because they were only used to inform the network estimates which we believed were the best estimates

[b] Higher of direct or indirect confidence (without consider imprecision), followed by consideration of imprecision and incoherence

[c] Rated down for risk of bias

[d] Rated down for imprecision

[e] Baseline risk comes from median of the placebo group in the included studies

[f] The indirect evidence contributed much more than direct evidence; thus, certainty of network started rating from the certainty of indirect estimate

Mortality – Complete PEPTIC Analysis

Comparison	Direct estimate (95% CrI); certainty of evidence	Indirect estimate (95% CrI); certainty of evidence	Network estimate (95% CrI); certainty of evidence	Baseline risk (per 1000)	Absolute difference (95% CrI) (per 1000)
**PPIs vs. placebo**	1.02 (0.90 to 1.18); **High**	1.04 (0.87 to 1.24); **Moderate**[d]	1.03 (0.93 to 1.14); **Moderate**[e]	Placebo: 304[c]	9 (–21 to 43)
**H2RAs vs. placebo**	0.97 (0.84 to 1.14); **High**	0.98 (0.86 to 1.14); **Moderate**[d]	0.98 (0.89 to 1.08); **Moderate**[e]	Placebo: 304[c]	–6 (–33 to 24)
**Sucralfate vs. placebo**	0.96 (0.71 to 1.30); **High**	0.91 (0.76 to 1.09); **High**	0.93 (0.80 to 1.07); **Very low**[f,g]	Placebo: 304[c]	–21 (–61 to 21)

CrI = credible interval; PPIs = proton pump inhibitors; H2RAs = histamine-2 receptor antagonists

[c] Baseline risk from placebo group in the SUP-ICU trial

[d] Rated down for risk of bias

[e] Rated down for imprecision

[f] Rated down 2 levels for imprecision

[g] Rated down for inconsistency between analyses/models

GRADE Summary of Findings for Pneumonia

Comparison	Direct estimate (95% CrI); Certainty of evidence[a]	Indirect estimate (95% CrI); Certainty of evidence[a]	Network estimate (95% CrI); Certainty of evidence[b]	Baseline risk (per 1000)	Absolute difference (95% CrI) (per 1000)
PPIs vs. placebo	1.06 (0.82 to 1.63); High	1.19 (0.78 to 1.83); High	1.08 (0.88 to 1.45); Low[d,e]	Placebo: 162[c]	13 (-19 to 73)
H2RAs vs. placebo	1.09 (0.80 to 1.48); High	1.04 (0.70 to 1.66); High	1.07 (0.85 to 1.37); Low[d,e]	Placebo: 162[c]	11 (-24 to 60)
Sucralfate vs. placebo	1.31 (0.42 to 4.09); High	0.89 (0.60 to 1.36); High	0.93 (0.65 to 1.38); Low[d,e]	Placebo: 162[c]	-11 (-57 to 62)

CrI = credible interval; PPIs = proton pump inhibitors; H2RAs = histamine-2 receptor antagonists

[a] We did not consider imprecision when rating for direct and indirect estimates because they were only used to inform the network estimates which we believed were the best estimates

[b] Higher of direct or indirect confidence (without consider imprecision), followed by consideration of imprecision and incoherence

[c] Baseline risk in the placebo group of the SUP-ICU trial

[d] Rated down for imprecision

[e] Rated down because results dependent on a series of methods judgments (believe the meta-regression and use only the blinded studies).

GRADE Summary of Findings for *Clostroides difficile* Infection – Complete PEPTIC Analysis

Comparison	Direct estimate (95% CrI); Certainty of evidence[a]	Indirect estimate (95% CrI); Certainty of evidence[a]	Network estimate (95% CrI); Certainty of evidence[b]	Baseline risk (per 1000)	Absolute difference (95% CrI) (per 1000)
PPIs vs. placebo	0.76 (0.29 to 2.15); High	NA	0.82 (0.34 to 2.21); Moderate[e]	Placebo: 15[c]	-3 (-10 to 18)
H2RAs vs. placebo	NA	1.08 (0.28 to 4.43); Moderate[d]	1.08 (0.28 to 4.43); Low[d,e]	Placebo: 15[c]	1 (-11 to 51)
PPIs vs. H2RAs	0.76 (0.29 to 2.15); Moderate[d]	NA	0.76 (0.28 to 2.16); Low[d,e]	PPIs: 12[f]	-4 (-31 to 6)

CrI = credible interval; PPIs = proton pump inhibitors; NA = not applicable; H2RAs = histamine-2 receptor antagonists

[a] We did not consider imprecision when rating for direct and indirect estimates because they were only used to inform the network estimates which we believed were the best estimates

[b] Higher of direct or indirect confidence (without consider imprecision), followed by consideration of imprecision and incoherence

[c] Baseline risk in the placebo group of the SUP-ICU trial

[d] Rated down for risk of bias

[e] Rated down for imprecision

[f] We used the point estimate of the PPIs group event rate in the comparison between PPIs and placebo as the baseline risk in the PPIs group in the PPIs vs. H2RAs comparator to calculate the absolute effect for PPIs vs. H2RAs

Supplemental Content 2.11 NMA From Source Guideline for PICO 1

https://gdt.gradepro.org/presentations/#/nma/nma_question_1f57d3a1-f4e6-43a3-9f1e-f7900d9566e3

Desirable effect

Intervention	clinically important gastrointestinal bleeding (CIB) for highest risk population– complete PEPTIC analysis<br>T/S -- per 1000<br>S/M -- per 1000<br>M/L -- per 1000	CIB – High risk population - complete PEPTIC analysis<br>T/S -- per 1000<br>S/M -- per 1000<br>M/L -- per 1000	CIB- moderate risk population- complete PEPTIC analysis<br>T/S -- per 1000<br>S/M -- per 1000<br>M/L -- per 1000	Overt gastrointestinal bleeding- highest risk of bleeding<br>T/S -- per 1000<br>S/M -- per 1000<br>M/L -- per 1000	Overt gastrointestinal bleeding-High risk population<br>T/S -- per 1000<br>S/M -- per 1000<br>M/L -- per 1000	Overt gastrointestinal bleeding- moderate risk population<br>T/S -- per 1000<br>S/M -- per 1000<br>M/L -- per 1000	length of ICU stay- complete PEPTIC analysis<br>Difference -- or more<br>Difference -- or more<br>Difference -- or more	Duration of mechanical ventilation- complete PEPTIC analysis<br>Difference -- or more<br>Difference -- or more<br>Difference -- or more
Proton pump inhibitor vs placebo	NMA<br>**49 fewer**<br>64 fewer to 31 fewer<br>**RR 0.46**<br>(0.29, 0.66)<br>**MODERATE**	NMA<br>**32 fewer**<br>43 fewer to 20 fewer<br>**RR 0.46**<br>(0.29, 0.66)<br>**MODERATE**	NMA<br>**16 fewer**<br>21 fewer to 10 fewer<br>**RR 0.46**<br>(0.29, 0.66)<br>**LOW**	NMA<br>**95 fewer**<br>131 fewer to 53 fewer<br>**RR 0.5**<br>(0.31, 0.72)<br>**MODERATE**	NMA<br>**62 fewer**<br>86 fewer to 35 fewer<br>**RR 0.5**<br>(0.31, 0.72)<br>**MODERATE**	NMA<br>**37 fewer**<br>52 fewer to 21 fewer<br>**RR 0.5**<br>(0.31, 0.72)<br>**MODERATE**	NMA<br>**MD 0.96**<br>(0.86, 1.08)<br>**MODERATE**	NMA<br>**MD 0.97**<br>(0.86, 1.11)<br>**MODERATE**
histamine-2 receptor antagonists vs placebo	NMA<br>**30 fewer**<br>47 fewer to 5 fewer<br>**RR 0.67**<br>(0.48, 0.94)<br>**MODERATE**	NMA<br>**20 fewer**<br>31 fewer to 4 fewer<br>**RR 0.67**<br>(0.48, 0.94)<br>**MODERATE**	NMA<br>**10 fewer**<br>16 fewer to 2 fewer<br>**RR 0.67**<br>(0.48, 0.94)<br>**MODERATE**	NMA<br>**65 fewer**<br>99 fewer to 21 fewer<br>**RR 0.66**<br>(0.48, 0.89)<br>**MODERATE**	NMA<br>**42 fewer**<br>65 fewer to 14 fewer<br>**RR 0.66**<br>(0.48, 0.89)	NMA<br>**25 fewer**<br>39 fewer to 8 fewer<br>**RR 0.66**<br>(0.48, 0.89)<br>**MODERATE**	NMA<br>**MD 0.96**<br>(0.85, 1.09)<br>**LOW**	NMA<br>**MD 0.95**<br>(0.84, 1.08)<br>**LOW**
sucralfate vs placebo	NMA<br>**16 fewer**<br>42 fewer to 26 more<br>**RR 0.82**<br>(0.53, 1.29)<br>**VERY LOW**	NMA<br>**11 fewer**<br>28 fewer to 17 more<br>**RR 0.82**<br>(0.53, 1.29)<br>**LOW**	NMA<br>**5 fewer**<br>9 more to 14 more<br>**RR 0.82**<br>(0.53, 1.29)<br>**LOW**	NMA<br>**0 fewer**<br>74 fewer to 129 more<br>**RR 1**<br>(0.61, 1.68)<br>**LOW**	NMA<br>**--**<br>49 fewer to 85 more<br>**RR 1**<br>(0.61, 1.68)<br>**LOW**	NMA<br>**--**<br>29 fewer to 51 more<br>**RR 1**<br>(0.61, 1.68)<br>**MODERATE**	NMA<br>**MD 0.93**<br>(0.81, 1.08)<br>**LOW**	NMA<br>**MD 1**<br>(0.87, 1.14)

Undesirable effect

Intervention	mortality—complete PEPTIC analysis<br>T/S 12 per 1000<br>S/M 30 per 1000<br>M/L 60 per 1000	Pneumonia<br>T/S 175 per 1000<br>S/M -- per 1000<br>M/L -- per 1000	Clostroides difficile infection – complete PEPTIC analysis<br>T/S -- per 1000<br>S/M -- per 1000<br>M/L -- per 1000
Proton pump inhibitor vs placebo	NMA<br>**9 more**<br>21 fewer to 43 more<br>**RR 1.03**<br>(0.93, 1.14)<br>**MODERATE**	NMA<br>**13 more**<br>19 fewer to 73 more<br>**RR 1.08**<br>(0.88, 1.45)<br>**LOW**	NMA<br>**3 fewer**<br>10 fewer to 18 more<br>**RR 0.82**<br>(0.34, 2.21)<br>**MODERATE**
histamine-2 receptor antagonists vs placebo	NMA<br>**6 fewer**<br>33 fewer to 24 more<br>**RR 0.98**<br>(0.89, 1.08)	No data	NMA<br>**1 more**<br>11 fewer to 51 more<br>**RR 1.08**<br>(0.28, 4.43)<br>**LOW**
sucralfate vs placebo	NMA<br>**21 fewer**<br>61 fewer to 21 more<br>**RR 0.93**<br>(0.8, 1.07)<br>**VERY LOW**	No data	No data

Supplemental Content 2.12 GRADE Evidence Profile for PICO 1

Question: ***Should critically ill adults with coagulopathy, shock, or chronic liver disease receive stress ulcer prophylaxis (SUP) or no SUP to prevent upper gastrointestinal bleeding?***
Setting: ICU, inpatients
Bibliography: [1,2,3,4,5,6]

Certainty assessment							№ of patients		Effect		Certainty	Importance
№ of studies	Study design	Risk of bias	Inconsistency	Indirectness	Imprecision	Other considerations	SUP	No SUP	Relative (95% CI)	Absolute (95% CI)		
**Clinically important GI bleeding: Proton pump inhibitor vs. No prophylaxis NMA of RCTs**												
8	randomized trials	not serious	not serious	not serious	serious[a]	None	82/4317 (1.9%)	3.0%	**RR 0.61** (0.42 to 0.89)[c,d]	**12 fewer per 1,000** (from 17 fewer to 3 fewer)	⨁⨁⨁◯ Moderate[a]	CRITICAL [aj]
								6.0%[b]		**23 fewer per 1,000** (from 35 fewer to 7 fewer)		
								9.0%[b]		**35 fewer per 1,000** (from 52 fewer to 10 fewer)		
**Clinically important GI bleeding: Proton pump inhibitor vs. No prophylaxis Granholm et al. - 2021 - secondary, post-hoc SUP ICU trial**												
1	non-randomized studies	not serious	not serious	not serious	not serious	None	This was a secondary, post-hoc study of the Stress Ulcer Prophylaxis in the Intensive Care Unit (SUP-ICU) trial. The sample size was fixed to the size of the SUP-ICU trial, which included 110 CIB events (41 in the pantoprazole group vs. 69 in the placebo group) and 236 overt GI bleeding events (88 in the pantoprazole group vs. 148 in the placebo group)<br>**Chronic Liver Disease**<br>• The overall effect of chronic liver disease on CIGIB was not statistically significant, with an odds ratio (OR) of 0.94 (95% CI: 0.29–3.02).<br>• In the placebo subgroup, the OR was 0.97 (95% CI: 0.23–4.09), while in the pantoprazole subgroup, the OR was 0.89 (95% CI: 0.12–6.59).<br>• The p-value for interaction was 0.96, and the S-value was 3.11, indicating no significant interaction between treatment allocation and chronic liver disease in predicting CIGIB.<br>**Acute Coagulopathy**<br>• The presence of acute coagulopathy was associated with a higher risk of CIGIB, with an overall OR of 2.36 (95% CI: 1.08–5.20).<br>• In the placebo subgroup, the OR was 2.42 (95% CI: 0.68–8.21), while in the pantoprazole subgroup, the OR was 2.29 (95% CI: 0.30–17.40).<br>• The p-value for interaction was 0.94, and the S-value was 3.67, suggesting no significant difference in the effect of acute coagulopathy between treatment groups.<br>**Circulatory Support [Shock]**<br>• The need for circulatory support was significantly associated with an increased risk of CIGIB, with an overall OR of 2.17 (95% CI: 1.34–3.52).<br>• In the placebo subgroup, the OR was 2.04 (95% CI: 1.13–3.71), while in the pantoprazole subgroup, the OR was 2.43 (95% CI: 1.07–5.51).<br>• The p-value for interaction was 0.74, and the S-value was 0.44, suggesting no significant interaction between treatment allocation and circulatory support in predicting CIGIB.<br>• Interpretation: The use of circulatory support is a strong independent predictor of CIGIB, with increased risk observed in both placebo and pantoprazole groups, although pantoprazole use does not appear to significantly alter this risk.				⨁⨁◯◯ Low	CRITICAL
**Clinically important GI bleeding: Proton pump inhibitor vs. No prophylaxis Pairwise SRMA NEJM 2024 (including REVISE)**												
9	randomized trials	not serious	not serious	not serious	not serious	None	71/4550 (1.6%)	158/4529 (3.5%)	**RR 0.51** (0.34 to 0.76)	**17 fewer per 1,000** (from 23 fewer to 8 fewer)		CRITICAL

Certainty assessment							№ of patients		Effect		Certainty	Importance
№ of studies	Study design	Risk of bias	Inconsistency	Indirectness	Imprecision	Other considerations	SUP	No SUP	Relative (95% CI)	Absolute (95% CI)		
								6.0%		**29 fewer per 1,000** (from 40 fewer to 14 fewer)	⨁⨁⨁⨁ High	
								9.0%		**44 fewer per 1,000** (from 59 fewer to 22 fewer)		
**Clinically important GI bleeding: Histamine-2 receptor antagonist vs. No prophylaxis NMA**												
14	randomized trials	not serious	not serious	not serious	serious[a]	none	17/1242 (1.4%)	3.0%	**OR 0.46** (0.27 to 0.79)[e,f]	**16 fewer per 1,000** (from 22 fewer to 6 fewer)	⨁⨁⨁◯ Moderate[a]	CRITICAL [ak]
								6.0%		**31 fewer per 1,000** (from 43 fewer to 12 fewer)		
								9.0%		**46 fewer per 1,000** (from 64 fewer to 18 fewer)		
**Clinically important GI bleeding: Sucralfate vs. No prophylaxis NMA**												
6	randomized trials	not serious	not serious	not serious	very serious[g]	none	20/874 (2.3%)	3.0%	**OR 0.76** (0.36 to 1.62)	**7 fewer per 1,000** (from 19 fewer to 18 more)	⨁⨁◯◯ Low[g]	CRITICAL [al]
								6.0%		**14 fewer per 1,000** (from 38 fewer to 34 more)		
								9.0%		**20 fewer per 1,000** (from 56 fewer to 48 more)		
**Overt GI bleeding: Proton pump inhibitor vs. No prophylaxis NMA**												
5	randomized trials	not serious	not serious	not serious	not serious	none	178/3867 (4.6%)	7.5%	**OR 0.59** (0.45 to 0.76)[h,i]	**29 fewer per 1,000** (from 40 fewer to 17 fewer)	⨁⨁⨁⨁ High	CRITICAL [am]
								12.5%		**47 fewer per 1,000** (from 65 fewer to 27 fewer)		
								19.0%		**68 fewer per 1,000** (from 95 fewer to 39 fewer)		
**Overt GI bleeding: Proton pump inhibitor vs. No prophylaxis Pairwise SRMA NEJM 2024 (including REVISE)**												
11	randomized trials	not serious	not serious	not serious	not serious	none	132/4709 (2.8%)	252/4682 (5.4%)	**RR 0.52** (0.38 to 0.70)	**26 fewer per 1,000** (from 33 fewer to 16 fewer)	⨁⨁⨁⨁ High	CRITICAL
								12.5%		**60 fewer per 1,000** (from 78 fewer to 38 fewer)		
								19.0%		**91 fewer per 1,000** (from 118 fewer to 57 fewer)		
**Overt GI bleeding: Proton pump inhibitor vs. No prophylaxis Granholm et al. - 2021 - secondary, post-hoc SUP ICU trial**												

Certainty assessment							№ of patients		Effect		Certainty	Importance
№ of studies	Study design	Risk of bias	Inconsistency	Indirectness	Imprecision	Other considerations	SUP	No SUP	Relative (95% CI)	Absolute (95% CI)		
1	non-randomized studies	not serious	not serious	not serious	not serious	none	This was a secondary, post-hoc study of the Stress Ulcer Prophylaxis in the Intensive Care Unit (SUP-ICU) trial. The sample size was fixed to the size of the SUP-ICU trial, which included 110 CIB events (41 in the pantoprazole group vs. 69 in the placebo group) and 236 overt GI bleeding events (88 in the pantoprazole group vs. 148 in the placebo group)<br>**Chronic Liver Disease**<br>• The overall effect of chronic liver disease on overt GIB was not statistically significant, with an odds ratio (OR) of 1.03 (95% CI: 0.47–2.26).<br>• In the placebo subgroup, the OR was 1.43 (95% CI: 0.60–3.42), while in the pantoprazole subgroup, the OR was 0.39 (95% CI: 0.05–2.90).<br>• The p-value for interaction was 0.19, and the S-value was 2.40, suggesting no statistically significant interaction between treatment allocation and chronic liver disease in predicting overt GIB.<br>**Acute Coagulopathy**<br>• The presence of acute coagulopathy was associated with a higher risk of overt GIB, with an overall OR of 1.95 (95% CI: 0.93–3.80).<br>• In the placebo subgroup, the OR was 1.65 (95% CI: 1.10–2.49), while in the pantoprazole subgroup, the OR was 0.86 (95% CI: 0.25–3.10).<br>• The p-value for interaction was 0.06, and the S-value was 3.97, suggesting a potential but not statistically significant interaction between treatment allocation and acute coagulopathy in predicting overt GIB.<br>**Circulatory Support**<br>• The need for circulatory support was significantly associated with an increased risk of overt GIB, with an overall OR of 1.80 (95% CI: 1.31–2.47).<br>• In the placebo subgroup, the OR was 1.94 (95% CI: 1.29–2.91), while in the pantoprazole subgroup, the OR was 1.60 (95% CI: 0.97–2.64).<br>• The p-value for interaction was 0.56, and the S-value was 0.82, indicating no significant interaction between treatment allocation and circulatory support in predicting overt GIB.				⨁⨁◯◯<br>Low	CRITICAL
**Overt GI bleeding: Histamine-2 receptor antagonist vs. No prophylaxis NMA**												
29	randomized trials	not serious	serious[j]	not serious	not serious	none	73/2428 (3.0%)	7.5%	**OR 0.38** (0.24 to 0.59)[j,k]	**45 fewer per 1,000** (from 56 fewer to 29 fewer)	⨁⨁⨁◯<br>Moderate[j]	CRITICAL [an]
								12.5%		**74 fewer per 1,000** (from 92 fewer to 47 fewer)		
								19.0%		**108 fewer per 1,000** (from 137 fewer to 68 fewer)		
**Overt GI bleeding: Sucralfate vs. No prophylaxis NMA**												
7	randomized trials	not serious	not serious	not serious	serious[l]	none	41/900 (4.6%)	7.5%	**OR 0.58** (0.30 to 1.11)	**30 fewer per 1,000** (from 51 fewer to 8 more)	⨁⨁⨁◯<br>Moderate[l]	CRITICAL [ao]
								12.5%		**48 fewer per 1,000** (from 84 fewer to 12 more)		
								19.0%		**70 fewer per 1,000** (from 124 fewer to 17 more)		

Certainty assessment							№ of patients		Effect		Certainty	Importance
№ of studies	Study design	Risk of bias	Inconsistency	Indirectness	Imprecision	Other considerations	SUP	No SUP	Relative (95% CI)	Absolute (95% CI)		
**Mortality: Proton pump inhibitor vs. No prophylaxis NMA**												
9	randomized trials	not serious	not serious	not serious	serious[m]	none	1329/4194 (31.7%)	30.4%	**OR 1.06** (0.90 to 1.28)[n]	**12 more per 1,000** (from 22 fewer to 55 more)	⨁⨁⨁◯ Moderate[m]	CRITICAL [ap]
**Mortality: Proton pump inhibitor vs. No prophylaxis Pairwise SRMA NEJM 2024 (including REVISE)**												
12	randomized trials	serious[o]	not serious	not serious	serious[o]	none	1460/4771 (30.6%)	1450/4692 (30.9%)	**RR 0.99** (0.93 to 1.05)	**3 fewer per 1,000** (from 22 fewer to 15 more)	⨁⨁◯◯ Low[o]	CRITICAL [as]
**Mortality- Histamine-2 receptor antagonist vs. No prophylaxis NMA**												
22	randomized trials	not serious	not serious	not serious	serious[m]	none	541/1835 (29.5%)	30.4%	**OR 0.96** (0.79 to 1.19)[p]	**9 fewer per 1,000** (from 47 fewer to 38 more)	⨁⨁⨁◯ Moderate[m]	CRITICAL [ar]
**Mortality- Sucralfate vs. No prophylaxis NMA**												
6	randomized trials	not serious	not serious	not serious	extremely serious[q]	none	244/872 (28.0%)	30.4%	**OR 0.89** (0.71 to 1.14)[r]	**24 fewer per 1,000** (from 67 fewer to 28 more)	⨁◯◯◯ Very low[q]	CRITICAL [aq]
**Pneumonia: Proton pump inhibitor vs. No prophylaxis NMA**												
6	randomized trials	serious[s]	not serious	not serious	serious[t]	none	842/3974 (21.2%)	16.2%	**OR 1.39** (0.98 to 2.10)[u]	**50 more per 1,000** (from 3 fewer to 127 more)	⨁⨁◯◯ Low[s,t]	CRITICAL [at]
**Pneumonia Proton pump inhibitor vs. No prophylaxis Pairwise SRMA NEJM 2024 (including REVISE)**												
8	randomized trials	not serious	not serious	not serious	very serious[v]	none	1067/4483 (23.8%)	1063/4466 (23.8%)	**RR 1.00** (0.92 to 1.09)	**0 fewer per 1,000** (from 19 fewer to 21 more)	⨁⨁◯◯ Low[v]	CRITICAL
**Pneumonia: Histamine-2 receptor antagonist vs. No prophylaxis NMA**												
11	randomized trials	serious[s]	not serious	not serious	serious[w]	none	227/1159 (19.6%)	16.2%	**OR 1.26** (0.89 to 1.85)[x]	**34 more per 1,000** (from 15 fewer to 101 more)	⨁⨁◯◯ Low[s,w]	CRITICAL [at]
**Pneumonia: Sucralfate vs. No prophylaxis NMA**												
3	randomized trials	serious[y]	not serious	not serious	serious[w]	none	52/370 (14.1%)	16.2%	**OR 0.85** (0.56 to 1.33)[z]	**21 fewer per 1,000** (from 64 fewer to 43 more)	⨁⨁◯◯ Low[w,y]	CRITICAL [at]
**Clostridium difficile infection-Proton pump inhibitor vs. No prophylaxis NMA**												
4	randomized trials	not serious	not serious	not serious	very serious[aa]	none	45/3720 (1.2%)	1.5%	**OR 0.82** (0.31 to 2.47)[ab]	**3 fewer per 1,000** (from 10 fewer to 21 more)	⨁⨁◯◯ Low[aa]	CRITICAL [au]
**Clostridium difficile infection-Proton pump inhibitor vs. No prophylaxis Pairwise SRMA NEJM 2024 (including REVISE)**												
6	randomized trials	not serious	not serious	not serious	very serious[aa]	none	35/4346 (0.8%)	30/4336 (0.7%)	**RR 1.20** (0.66 to 2.16)	**1 more per 1,000** (from 2 fewer to 8 more)	⨁⨁◯◯ Low[aa]	CRITICAL [au]
**Clostridium difficile infection- Histamine-2 receptor antagonist vs. No prophylaxis NMA**												

Certainty assessment							№ of patients		Effect		Certainty	Importance
№ of studies	Study design	Risk of bias	Inconsistency	Indirectness	Imprecision	Other considerations	SUP	No SUP	Relative (95% CI)	Absolute (95% CI)		
0	non-randomized studies	very serious[ac]	not serious	not serious	very serious[ad]	none	Indirect estimate was rated down because one of the direct estimates (PPIs versus H2RAs) in the first order loop which contributed to the indirect estimate was rated down for risk of bias. No direct evidence.<br>Odds ratio 0.94 (CI 95% 0.06 — 14.99). Difference: **0 fewer per 1000** (CI 95% 14 fewer — 226 more)				⊕◯◯◯<br>Very low[ac,ad]	CRITICAL
**Duration of MV (days)-Proton pump inhibitor vs. No prophylaxis Pairwise SRMA NEJM 2024 (including REVISE)**												
7	randomized trials	not serious	serious	not serious	serious[ae]	none	2880	2864	-	MD **1.5 higher**<br>(0.9 lower to 3.9 higher)	⊕⊕◯◯<br>Low[ae]	IMPORTANT[av]
**Duration of MV- (days) - Histamine-2 receptor antagonist vs. No prophylaxis NMA**												
6	randomized trials	serious[af]	not serious	not serious	serious[ag]	none	593	0	-	MD **0.8 lower**<br>(1.9 lower to 0.3 higher)	⊕⊕◯◯<br>Low[af,ag]	IMPORTANT[av]
**Duration of ICU stay (days)-Proton pump inhibitor vs. No prophylaxis Pairwise SRMA NEJM 2024 (including REVISE)**												
7	randomized trials	not serious	not serious	not serious	very serious[ah]	none	2903	2842	-	MD **0.1 lower**<br>(0.8 lower to 0.6 higher)	⊕⊕◯◯<br>Low[ah]	IMPORTANT[av]
**Duration of ICU stay- (days) - Histamine-2 receptor antagonist vs. No prophylaxis NMA**												
4	randomized trials	not serious	not serious	not serious	serious[ai]	none	436	0	-	MD **0.4 lower**<br>(1.4 lower to 0.6 higher)	⊕⊕⊕◯<br>Moderate[ai]	IMPORTANT[av]
**Other Outcomes not reported and rated important by panel**												
0							Ventilator-Free Days- Not reported<br>Renal Failure and AKI - Not reported<br>Delirium - Not reported<br>Thrombocytopenia - Not reported				-	IMPORTANT

CI: confidence interval; **MD:** mean difference; **OR:** odds ratio; **RR:** risk ratio

Explanations

a. Rated down by 1 level for imprecision. The 95% confidence interval includes an unimportant difference in clinically important GI bleeding.
b. We grouped patients into categories according to risk of clinically important gastrointestinal bleeding: low risk (<2%), moderate risk (2-4%), high risk (>4-8%), and highest risk (>8%) and calculated absolute effects for each category for clinically important gastrointestinal bleeding and overt bleeding. We used event rate in the placebo group of the SUP-ICU trial (refer to the guideline outline- PICO #1 supplementary material).
c. 2020 updated SR and NMA showed the following for patients at highest or high risk of bleeding: moderate certainty evidence from the complete PEPTIC analysis (risk ratio 0.46, 95% credible interval 0.29 to 0.66, 32 fewer per 1000 for high-risk patients, and 49 fewer per 1000 for highest-risk patients).
d. SCCM NMA conducted by the guideline panel was not stratified by baseline bleeding risk, which may influence the applicability of the estimates to populations at different risk levels. For the PPI versus placebo comparison, the direct estimate from conventional MA was 0.62 (95% CrI: 0.43, 0.89), while the estimate from node splitting was 0.65 (95% CrI: 0.37, 1.30), both with moderate certainty. The indirect estimate showed a lower effect size at 0.30 (95% CrI: 0.12, 0.68), also rated as moderate certainty, and the NMA estimate was 0.52 (95% CrI: 0.30, 0.81), with moderate certainty.

e. SCCM NMA conducted by the guideline panel was not stratified by baseline bleeding risk, which may influence the applicability of the estimates to populations at different risk levels. For CIGIB, the comparison between placebo and H2RA showed a direct estimate from conventional meta-analysis (MA) of 1.84 (95% CrI: 0.74, 4.56) and from node splitting analysis of 1.78 (95% CrI: 0.84, 3.88), both with high certainty of evidence. The indirect estimate was 0.83 (95% CrI: 0.31, 1.70) with moderate certainty, while the network meta-analysis (NMA) estimate was 1.22 (95% CrI: 0.74, 1.98) with low certainty.

f. 2020 updated SR and NMA showed moderate certainty evidence that H2RAs probably reduce CIB (complete: 0.67, 0.48 to 0.94, 20 fewer for high-risk patients).

g. Rated down by 2 levels due to very serious imprecision. The 95% confidence interval is wide and includes an important increase and reduction in clinically important GI bleeding.

h. SCCM NMA conducted by the guideline panel was not stratified by baseline bleeding risk, which may influence the applicability of the estimates to different patient populations. For Overt GIB: PPI vs. placebo, the direct estimate from conventional MA was 0.62 (95% CrI: 0.43, 0.89), while node splitting analysis yielded 0.65 (95% CrI: 0.37, 1.30), both with moderate certainty. The indirect estimate was 0.30 (95% CrI: 0.12, 0.68), also with moderate certainty, while the NMA estimate was 0.52 (95% CrI: 0.30, 0.81), again with moderate certainty.

i. 2020 updated SR and NMA showed that Both PPIs (risk ratio 0.50, 95% credible interval 0.31 to 0.72, moderate certainty) and H2RAs (0.66, 0.48 to 0.89, moderate certainty) probably reduce overt bleeding.

j. Serious inconsistency I2 = 55%.

k. SCCM NMA conducted by the guideline panel was not stratified by baseline bleeding risk, which may influence the applicability of the estimates to different patient populations. For Overt GIB: Placebo and H2RA showed a direct estimate from conventional meta-analysis (MA) of 1.84 (95% CrI: 0.74, 4.55) and from node splitting analysis of 1.78 (95% CrI: 0.86, 3.88), both with high certainty of evidence. The indirect estimate was 0.84 (95% CrI: 0.31, 1.70) with moderate certainty, while the network meta-analysis (NMA) estimate was 1.22 (95% CrI: 0.74, 1.98) with low certainty.

l. Rated down by 1 level due to serious imprecision. The 95% confidence interval includes no difference in overt GI bleeding.

m. Rated down by 1 level for imprecision. The 95% credible interval includes an important increase and reduction in mortality.

n. 2020 updated SR and NMA showed the following for mortality, including data from the PEPTIC trial: risk ratio 1.03, 95% credible interval 0.93 to 1.14, moderate certainty. SCCM NMA conducted by the panel showed similar results: in the PPI vs. placebo comparison, the direct estimate from conventional MA was 1.03 (95% CrI: 0.94, 1.14), while the estimate from node splitting was 1.04 (95% CrI: 0.81, 1.22), both with high certainty. The indirect estimate was 0.98 (95% CrI: 0.80, 1.19) with high certainty, and the NMA estimate was 1.02 (95% CrI: 0.92, 1.40) with moderate certainty. These results indicate that PPI use does not significantly impact overall mortality compared to placebo.

o. Rated down for imprecision and ROB. Because the point estimate was very close to the null, we switched to rate certainty in little to no effect. Uncertainty arose from the subgroup analysis based on disease severity.

p. 2020 updated SR and NMA showed the following for mortality, including data from the PEPTIC trial: risk ratio 0.98, 95% credible interval 0.89 to 1.08, moderate certainty. SCCM NMA conducted by the panel showed similar results: Placebo and H2RA demonstrated a direct estimate from conventional MA of 1.05 (95% CrI: 0.87, 1.27) and from node splitting analysis of 1.06 (95% CrI: 0.88, 1.25), with high certainty of evidence. The indirect estimate was 1.02 (95% CrI: 0.85, 1.23) with high certainty, while the network meta-analysis (NMA) estimate was 1.04 (95% CrI: 0.93, 1.16) with moderate certainty.

q. The 95% credible interval is very wide and includes an important increase and reduction in mortality.

r. 2020 updated SR and NMA showed the following for mortality, including data from the PEPTIC trial: risk ratio 0.93, 95% credible interval 0.8 to 1.07, very low certainty. SCCM NMA conducted by the panel showed similar results: Sucralfate vs. placebo comparison, the direct estimate from conventional MA was 0.89 (95% CrI: 0.64, 1.24), while node splitting analysis yielded 0.89 (95% CrI: 0.64, 1.24), both with high certainty. The indirect estimate was 0.95 (95% CrI: 0.77, 1.17), also with high certainty, while the NMA estimate was 0.93 (95% CrI: 0.78, 1.11), with low certainty.

s. Rated down by 1 level. We are skeptical of the result because the pooled result including smaller studies conflicts with the evidence from the largest study (SUP-ICU).

t. Rated down by 1 level. The 95% credible interval includes no difference in pneumonia.

u. 2020 updated SR and NMA showed the following for pneumonia, including data from the PEPTIC trial risk ratio 1.08, 95% credible interval 0.88 to 1.45, low certainty. SCCM NMA conducted by the panel showed similar results: PPI vs. placebo comparison, the direct estimate from conventional MA was 1.02 (95% CrI: 0.88, 1.19), while the estimate from node splitting was 1.05 (95% CrI: 0.82, 1.48), both with high certainty. The indirect estimate was 1.39 (95% CrI: 0.93, 2.07) with high certainty, and the NMA estimate was 1.14 (95% CrI:

0.93, 1.54) with moderate certainty. These results indicate that PPI use does not significantly impact pneumonia risk compared to placebo, although there is some uncertainty in indirect estimates.
v. Rated down twice for imprecision. The point estimate was very close to the null, and we switched to rate certainty in little to no effect.
w. Rated down by 1 level. The 95% credible interval includes an important increase and reduction in pneumonia.
x. 2020 updated SR and NMA showed the following for pneumonia, including data from the PEPTIC trial: risk ratio 1.07, 95% credible interval 0.85 to 1.37, low certainty, low certainty. SCCM NMA conducted by the panel showed similar results: Placebo and H2RA demonstrated a direct estimate from conventional meta-analysis of 0.89 (95% CrI: 0.60, 1.33) and from node splitting analysis of 0.86 (95% CrI: 0.62, 1.23), both with high certainty of evidence. The indirect estimate was 1.09 (95% CrI: 0.72, 1.54) with high certainty, while the network meta-analysis (NMA) estimate was 0.96 (95% CrI: 0.73, 1.21) with moderate certainty. These findings suggest no significant difference in pneumonia risk between H2RA and placebo.
y. Both direct and indirect estimates were rated down for risk of bias. Direct estimate was rated down because high risk of bias studies dominated the result. Indirect estimate was rated down because the direct estimates (H2RAs vs. placebo and H2RAs vs. sucralfate) in the first order loop which contributed to the indirect estimate were rated down for risk of bias.
z. 2020 updated SR and NMA showed the following for pneumonia, including data from the PEPTIC trial: risk ratio 0.93, 95% credible interval 0.65 to 1.38, low certainty, low certainty. SCCM NMA conducted by the panel showed similar results: Sucralfate vs. placebo comparison, the direct estimate from conventional MA was 1.49 (95% CrI: 0.81, 2.73), while node splitting analysis yielded 1.47 (95% CrI: 0.98, 2.78), both with high certainty. The indirect estimate was 0.74 (95% CrI: 0.3, 1.01), also with high certainty, while the NMA estimate was 0.84 (95% CrI: 0.64, 1.14), with very low certainty. These findings suggest a potential increased risk of pneumonia with sucralfate, though the confidence intervals are wide, indicating uncertainty.
aa. Rated down twice for imprecision.
ab. 2020 updated SR and NMA showed the following for CDI, including data from the PEPTIC trial: risk ratio 0.82, 95% credible interval 0.34 to 2.21, moderate certainty. SCCM NMA conducted by the panel showed similar results: comparison between PPI and placebo showed a direct estimate from conventional MA of 0.73 (95% CrI: 0.42, 1.26). However, the effect was not estimable using node splitting analysis, and there were no available indirect estimates. The certainty of evidence for the direct estimate was moderate, while the NMA was also not estimable, leading to moderate certainty overall.
ac. Indirect estimate was rated down because one of the direct estimates (PPIs vs. H2RAs) in the first order loop which contributed to the indirect estimate was rated down for risk of bias. No direct evidence.
ad. The 95% credible interval is very wide and includes an important increase and reduction in *C. difficile* infection.
ae. Rated down for inconsistency and imprecision. Point estimate was very close to the null, and we switched to rate certainty in little to no effect.
af. Both direct and indirect estimates were rated down for risk of bias. Direct estimate was rated down because high risk of bias studies dominated the result. Indirect estimate was rated down because one of the direct estimates (PPIs vs. H2RAs) in the first order loop which contributed to the indirect estimate was rated down for risk of bias.
ag. The 95% credible interval includes an important increase and reduction in duration of mechanical ventilation.
ah. Rated down twice for imprecision
ai. The 95% credible interval includes an important reduction in length of ICU stay.

Supplemental Content 2.13 PICO 1: Evidence-to-Decision (EtD) Framework

**Should SUP vs. No SUP be used for critically ill adults in the ICU with coagulopathy, shock, or chronic liver disease?**	
**POPULATION:**	Critically ill adults in the ICU with coagulopathy, shock, or chronic liver disease
**INTERVENTION:**	SUP
**COMPARISON:**	No SUP
**MAIN OUTCOMES:**	**Clinically important gastrointestinal bleeding** is typically defined as evidence of upper gastrointestinal bleeding with any of the following: significant haemodynamic changes not explained by other causes, need for transfusion of more than two units of blood, significant decrease in haemoglobin level, evidence of bleeding on upper gastrointestinal endoscopy, or need for surgery to control bleeding.<br>**Overt bleeding** (*that is visible as haematemesis, haematochezia, or melaena*) does not always have important consequences: overt bleeding, which includes important and unimportant bleeding, is more common than clinically important bleeding. The absolute reduction of overt bleeding achieved with prophylaxis is approximately twice that of clinically important bleeding.
**SETTING:**	Inpatient setting (Excluded population: pediatric patients and those receiving treatment [e.g., for peptic ulcer, gastroesophageal reflux disease, Zollinger-Ellison syndrome, or eradication of *Helicobacter pylori*] who require acid suppressing therapy do not fall under the scope of this guideline).
**PERSPECTIVE:**	Clinicians
**BACKGROUND:**	Stress ulcers, characterized by gastric or duodenal mucosal erosions, are a known complication in critically ill patients, often leading to upper gastrointestinal (GI) bleeding. Although stress ulcer-related mortality is rare, GI bleeding can result in increased morbidity, prolonged ICU stays, and additional healthcare interventions such as blood transfusions and endoscopy. As a preventive strategy, **stress ulcer prophylaxis (SUP)** is widely implemented in ICU settings, primarily using **proton pump inhibitors (PPIs) or histamine-2 receptor antagonists (H2RAs)**. Historically, PPIs have been the preferred choice due to their superior efficacy in reducing clinically significant GI bleeding. However, recent trials, including **SUP-ICU and REVISE**, have questioned the universal use of SUP, suggesting that while PPIs decrease GI bleeding incidence, they may not improve **overall mortality** and could be associated with adverse effects. Given the evolving evidence, **a risk-stratified approach** to SUP has been proposed, balancing the benefits of preventing GI bleeding against potential harms. This guideline aims to provide updated recommendations based on **current evidence, clinical effectiveness, patient safety, and resource implications** to ensure the **judicious and individualized use of SUP in patients in the ICU**. (1, 2)
**CONFLICT OF INTERESTS:**	Some panel members are co-authors of one of the largest trials on stress ulcer prophylaxis (the SUP-ICU trial), REVISE trial, or the 2024 SCCM and ASHP "Guideline for the Prevention of Stress-Related Gastrointestinal Bleeding in Critically Ill Adults"

ASSESSMENT

**Problem**<br>Is the problem a priority?		
**JUDGEMENT**	**RESEARCH EVIDENCE**	**ADDITIONAL CONSIDERATIONS**
○ No<br>○ Probably no<br>○ Probably yes<br>● Yes<br>○ Varies<br>○ Don't know	• Upper gastrointestinal (GI) mucosal lesions are commonly observed in patients in the ICU, with endoscopic findings present in up to 90% of cases. The incidence of overt GI bleeding in patients in the ICU varies significantly based on definitions and patient populations, ranging from 0.6% to 8.5% in all patients in the ICU and reaching up to 15% in those not receiving stress ulcer prophylaxis (SUP). (3)(4)<br>• However, much of the available data originates from older studies, with more recent trials suggesting a declining incidence. Variability in bleeding definitions and clinical significance continues to complicate interpretation of past findings. Large, contemporary cohorts such as **SUP-ICU** provide **valuable insights into the epidemiology of stress ulcer bleeding**.<br>• The study found that **clinically significant GI bleeding occurred in only 2.6% (95% CI: 1.6–3.6%)** of patients in the ICU.<br>• This supports the notion that **stress ulcer bleeding is relatively rare**, reinforcing the need for a **targeted, risk-based approach** rather than universal prophylaxis.<br>• **73% of patients in the ICU** received acid suppression therapy, primarily PPIs.<br>• Given the evolving evidence, this guideline aims to provide **clear, evidence-based recommendations** for the **judicious use of stress ulcer prophylaxis**, ensuring that **benefits outweigh potential harms** while addressing patient safety and resource utilization.<br>• **To estimate baseline risk for clinically important gastrointestinal bleeding (CIGIB) and overt bleeding,** we applied the same approach as used in prior clinical practice guidelines (BMJ 2020; 368 doi: https://doi.org/10.1136/bmj.l6722). (5)<br>• Our approach integrates clinical trial data from SUP-ICU and REVISE trials (updated the estimates for mechanically ventilated patients), observational studies, and meta-analyses by Granholm et al. for estimate baseline risks of CIB and overt GIB across different patient risk groups (refer to the guideline outline- PICO #1 supplementary material). (6, 7, 8)	**Panel comments:**<br><br>• Agree with the problem being a priority, but "making the current true incidence of stress ulcer bleeding in ICU patients largely unknown" is a statement I do not fully agree with - "largely unknown" is too harsh a statement, IMHO. The SUP-ICU cohort (https://doi.org/10.1007/s00134-015-3725-1) is relatively recent, relatively large, and had broad geographical coverage.<br><br>• Are there data about the presence of multiple factors, each of which carrying a low risk or moderate risk? Is there a definition for coagulopathy (cutoff for INR, plts, ...)?

Baseline Risk of Clinically Important Gastrointestinal Bleeding and Overt Gastrointestinal Bleeding for Each Risk Group

Risk Group	Risk Factors	Clinically Important Gastrointestinal Bleeding	Overt Gastrointestinal Bleeding
**Low Risk**	- Critically ill patients without any risk factor<br>- Acute hepatic failure<br>- Use of steroids/immunosuppression<br>- Use of anticoagulants<br>- Cancer<br>- Male gender	**Baseline Risk:** 10-20 per 1000<br>**Representative Risk chosen for evidence profile:** 12 per 1000	**Baseline Risk:** 20-60 per 1000<br>**Representative Risk chosen for evidence profile:** 26 per 1000
**Moderate Risk**	- Shock<br>- Sepsis<br>- Acute kidney injury	**Baseline Risk:** 21-40 per 1000<br>**Representative Risk chosen for evidence profile:** 30 per 1000	**Baseline Risk:** 61-90 per 1000<br>**Representative Risk chosen for evidence profile:** 75 per 1000
**High Risk**	- Coagulopathy<br>- Mechanical ventilation (based on REVISE trial data)#	**Baseline Risk:** 41-80 per 1000<br>**Representative Risk chosen for evidence profile:** 60 per 1000	**Baseline Risk:** 91-160 per 1000<br>**Representative Risk chosen for evidence profile:** 125 per 1000
**Highest Risk**	- Chronic liver disease	**Baseline Risk:** 81-100 per 1000<br>**Representative Risk chosen for evidence profile:** 90 per 1000	**Baseline Risk:** 161-220 per 1000<br>**Representative Risk chosen for evidence profile:** 190 per 1000

Granholm 2019 et al demonstrated no conclusive evidence supporting mechanical ventilation as an independent risk factor for UGIB may be explained by modern lung-protective ventilation strategies, including lower tidal volumes and airway pressures. Updated estimated risks from the **REVISE trial** for the baseline risk (event rate in the placebo group) of clinically important bleeding (CIB) is 4.5% (45 per 1000 patients-High risk) in patients with mechanical ventilation (refer to the guideline outline- PICO #1 supplementary material for detailed calculation).

The **SUP-ICU study** demonstrated that **patients with three or more co-existing diseases had an 8.9-fold higher risk** of GI bleeding > the presence of multiple risk factors likely **compounds the risk.**

Study-level definitions of coagulopathy, shock, and chronic liver disease were heterogeneous or unreported. Panel interpretations follow descriptive criteria adapted from the BMJ 2020 Rapid Recommendation and acknowledge that universal thresholds do not exist. Coagulopathy = one or more of the following: platelet count $< 50 \times 10^9$/L, INR > 1.5, or prothrombin time > 20 s. Shock = one or more of the following: continuous vasopressor or inotrope infusion, SBP < 90 mm Hg, MAP < 70 mm Hg, or lactate ≥ 4 mmol/L. Chronic liver disease = one or more of the following: portal hypertension; cirrhosis proven by biopsy or imaging; history of variceal bleeding or hepatic encephalopathy. Exact cut-offs vary among studies; clinicians should apply local clinical judgment and laboratory standards.

Desirable Effects

How substantial are the desirable anticipated effects?

JUDGEMENT

- ○ Trivial
- ○ Small
- ○ Moderate
- ○ Large
- ● Varies
- ○ Don't know

RESEARCH EVIDENCE

Proton pump inhibitor vs. No prophylaxis

- PPIs reduce the risk of GI bleeding. For people with high and highest risk of clinically important GI bleeding, the effect is probably large enough that most people would choose to use them.
- In patients at **low risk** of clinically significant GI bleeding, PPIs reduce the absolute risk from 12 per 1000 to 7 per 1000, a difference **5 fewer per 1000 (OR 0.61, 95% CI 0.42–0.89).**
- Probably do not have an important effect on duration of MV and ICU LOS.

Outcomes	№ of participants (studies) Follow-up	Certainty of the evidence (GRADE)	Relative effect (95% CI)	Anticipated absolute effects* (95% CI): Risk with No SUP	Anticipated absolute effects* (95% CI): Risk difference with SUP
Clinically important GI bleeding: Proton pump inhibitor vs. No prophylaxis NMA of RCTs	4317 (8 RCTs)	⊕⊕⊕◯ Moderate[a]	**RR 0.61** (0.42 to 0.89)[b,c]	Moderate	
				30 per 1,000	**12 fewer per 1,000** (17 fewer to 3 fewer)
				high	
				60 per 1,000[d]	**23 fewer per 1,000** (35 fewer to 7 fewer)
				Highest	
				90 per 1,000[d]	**35 fewer per 1,000** (52 fewer to 10 fewer)
Clinically important GI bleeding: Proton pump inhibitor vs. No prophylaxis Granholm et al. - 2021 - secondary, post-hoc SUP ICU trial	0 (1 non-randomized study)	⊕⊕◯◯ Low	-	This was a secondary, post-hoc study of the Stress Ulcer Prophylaxis in the Intensive Care Unit (SUP-ICU) trial. The sample size was fixed to the size of the SUP-ICU trial, which included 110 CIB events (41 in the pantoprazole group vs. 69 in the placebo group) and 236 overt GI bleeding events (88 in the pantoprazole group vs. 148 in the placebo group)<br>**Chronic Liver Disease**<br>• The overall effect of chronic liver disease on CIGIB was not statistically significant, with an odds ratio (OR) of 0.94 (95% CI: 0.29–3.02).	

ADDITIONAL CONSIDERATIONS

Panel comments:

- Difficult to judge what "substantial" is. From the evidence synthesis, I would say at least moderate, but would be ok with large effect also.
- "0 participants" and "non-randomized study" seems misleading for our post-hoc analysis of SUP-ICU - analyses were conducted respecting the randomization but also considering baseline factors, so these are comparable to subgroup analysis in approach. A challenge with the overall statement is that while SUP reduces CIB/overt GIB, it does not substantially affect other outcomes, and both outcomes can be treated relatively efficiently. Those with highest risk are also those most severely ill, and although the evidence is somewhat limited, the three major recent trials (SUP-ICU, REVISE, PEPTIC) indicate that beneficial effects on mortality with PPIs are most likely in those least ill (= lowest risk), and that PPIs may increase mortality in those most ill. While the evidence is far from perfect, the signal is the same in the three trials.

The panel pointed that the magnitude of reduction in GI bleeding varies based on:

<table>
<tr>
<td></td>
<td>
<table>
<tr>
<td></td>
<td></td>
<td></td>
<td></td>
<td colspan="2">

In the placebo subgroup, the OR was 0.97 (95% CI: 0.23–4.09), while in the pantoprazole subgroup, the OR was 0.89 (95% CI: 0.12–6.59).
The p-value for interaction was 0.96, and the S-value was 3.11, indicating no significant interaction between treatment allocation and chronic liver disease in predicting CIGIB.

Acute Coagulopathy

The presence of acute coagulopathy was associated with a higher risk of CIGIB, with an overall OR of 2.36 (95% CI: 1.08–5.20).
In the placebo subgroup, the OR was 2.42 (95% CI: 0.68–8.21), while in the pantoprazole subgroup, the OR was 2.29 (95% CI: 0.30–17.40).
The p-value for interaction was 0.94, and the S-value was 3.67, suggesting no significant difference in the effect of acute coagulopathy between treatment groups.

Circulatory Support [Shock]

The need for circulatory support was significantly associated with an increased risk of CIGIB, with an overall OR of 2.17 (95% CI: 1.34–3.52).
In the placebo subgroup, the OR was 2.04 (95% CI: 1.13–3.71), while in the pantoprazole subgroup, the OR was 2.43 (95% CI: 1.07–5.51).
The p-value for interaction was 0.74, and the S-value was 0.44, suggesting no significant interaction between treatment allocation and circulatory support in predicting CIGIB.
Interpretation: The use of circulatory support is a strong independent predictor of CIGIB, with increased risk observed in both placebo and pantoprazole groups, although pantoprazole use does not appear to significantly alter this risk.

</td>
</tr>
<tr>
<td rowspan="3">Clinically important GI bleeding: Proton pump inhibitor vs. No prophylaxis Pairwise</td>
<td rowspan="3">9079
(9 RCTs)</td>
<td rowspan="3">⊕⊕⊕⊕
High</td>
<td rowspan="3">RR 0.51
(0.34 to 0.76)</td>
<td colspan="2">Study population</td>
</tr>
<tr>
<td>35 per 1,000</td>
<td>17 fewer per 1,000
(23 fewer to 8 fewer)</td>
</tr>
<tr>
<td colspan="2">high</td>
</tr>
</table>
</td>
<td>
Baseline risk category (low, moderate, high).

Moderate-risk patients: Absolute risk reduction of 1.2% → categorized as small.
High-risk patients: Absolute risk reduction of 2.3% → categorized as moderate.
Highest-risk patients: Absolute risk reduction of 3.5% → categorized as moderate to large.
Low-risk patients: Absolute risk reduction of 0.5%, considered very small/trivial

Specific SUP agent used (PPI vs. H2RA vs. placebo).

Definition of GI bleeding (overt vs. clinically significant).

Two panels selected moderate
</td>
</tr>
</table>

SRMA NEJM 2024 (including REVISE)				60 per 1,000	**29 fewer per 1,000** (40 fewer to 14 fewer)
				Highest	
				90 per 1,000	**44 fewer per 1,000** (59 fewer to 22 fewer)
Overt GI bleeding: Proton pump inhibitor vs. No prophylaxis NMA	3867 (5 RCTs)	⊕⊕⊕⊕ High	**OR 0.59** (0.45 to 0.76)[e,f]	Moderate	
				75 per 1,000	**29 fewer per 1,000** (40 fewer to 17 fewer)
				High	
				125 per 1,000	**47 fewer per 1,000** (65 fewer to 27 fewer)
				Highest	
				190 per 1,000	**68 fewer per 1,000** (95 fewer to 39 fewer)
Overt GI bleeding: Proton pump inhibitor vs. No prophylaxis Pairwise SRMA NEJM 2024 (including REVISE)	9391 (11 RCTs)	⊕⊕⊕⊕ High	**RR 0.52** (0.38 to 0.70)	Study population	
				54 per 1,000	**26 fewer per 1,000** (33 fewer to 16 fewer)
				High	
				125 per 1,000	**60 fewer per 1,000** (78 fewer to 38 fewer)
				Highest	
				190 per 1,000	**91 fewer per 1,000** (118 fewer to 57 fewer)
Overt GI bleeding: Proton pump inhibitor vs. No prophylaxis Granholm et al. - 2021 - secondary, post-hoc SUP ICU trial	0 (1 non-randomized study)	⊕⊕◯◯ Low	-	This was a secondary, post-hoc study of the Stress Ulcer Prophylaxis in the Intensive Care Unit (SUP-ICU) trial. The sample size was fixed to the size of the SUP-ICU trial, which included 110 CIB events (41 in the pantoprazole group vs. 69 in the placebo group) and 236 overt GI bleeding events (88 in the pantoprazole group vs. 148 in the placebo group)<br>**Chronic Liver Disease**<br>• The overall effect of chronic liver disease on overt GIB was not statistically significant, with an odds ratio (OR) of 1.03 (95% CI: 0.47–2.26).	

				• In the placebo subgroup, the OR was 1.43 (95% CI: 0.60–3.42), while in the pantoprazole subgroup, the OR was 0.39 (95% CI: 0.05–2.90).<br>• The p-value for interaction was 0.19, and the S-value was 2.40, suggesting no statistically significant interaction between treatment allocation and chronic liver disease in predicting overt GIB.<br><br>**Acute Coagulopathy**<br>• The presence of acute coagulopathy was associated with a higher risk of overt GIB, with an overall OR of 1.95 (95% CI: 0.93–3.80).<br>• In the placebo subgroup, the OR was 1.65 (95% CI: 1.10–2.49), while in the pantoprazole subgroup, the OR was 0.86 (95% CI: 0.25–3.10).<br>• The p-value for interaction was 0.06, and the S-value was 3.97, suggesting a potential but not statistically significant interaction between treatment allocation and acute coagulopathy in predicting overt GIB.<br><br>**Circulatory Support**<br>• The need for circulatory support was significantly associated with an increased risk of overt GIB, with an overall OR of 1.80 (95% CI: 1.31–2.47).<br>• In the placebo subgroup, the OR was 1.94 (95% CI: 1.29–2.91), while in the pantoprazole subgroup, the OR was 1.60 (95% CI: 0.97–2.64).<br>• The p-value for interaction was 0.56, and the S-value was 0.82, indicating no significant interaction between treatment allocation and circulatory support in predicting overt GIB.	
Duration of MV (days)-Proton pump inhibitor vs. No prophylaxis Pairwise SRMA NEJM 2024 (including REVISE)	5744<br>(7 RCTs)	⊕⊕◯◯<br>Low[g]	-	The mean duration of MV (days)-Proton pump inhibitor vs. No prophylaxis Pairwise SRMA NEJM 2024 (including REVISE) was **0**	MD **1.5 higher**<br>(0.9 lower to 3.9 higher)
Duration of ICU stay (days)-Proton pump inhibitor vs. No	5745<br>(7 RCTs)	⊕⊕◯◯<br>Low[h]	-	The mean duration of ICU stay (days)-Proton pump inhibitor vs. No prophylaxis Pairwise SRMA	MD **0.1 lower**<br>(0.8 lower to 0.6 higher)

prophylaxis Pairwise SRMA NEJM 2024 (including REVISE)				NEJM 2024 (including REVISE) was **0**	

a. Rated down by 1 level for imprecision. The 95% confidence interval includes an unimportant difference in clinically important GI bleeding.
b. 2020 updated SR and NMA showed moderate certainty evidence from the complete PEPTIC analysis for patients at highest or high risk of bleeding (risk ratio 0.46, 95% credible interval 0.29 to 0.66, 32 fewer per 1000 for high risk patients, and 49 fewer per 1000 for highest risk).
c. SCCM NMA conducted by the guideline panel was not stratified by baseline bleeding risk, which may influence the applicability of the estimates to populations at different risk levels. For the PPI versus placebo comparison, the direct estimate from conventional MA was 0.62 (95% CrI: 0.43, 0.89), while the estimate from node splitting was 0.65 (95% CrI: 0.37, 1.30), both with moderate certainty. The indirect estimate showed a lower effect size at 0.30 (95% CrI: 0.12, 0.68), also rated as moderate certainty, and the NMA estimate was 0.52 (95% CrI: 0.30, 0.81), with moderate certainty.
d. We grouped patients into categories according to risk of clinically important gastrointestinal bleeding: low risk (<2%), moderate risk (2–4%), high risk (>4–8%), and highest risk (>8%) and calculated absolute effects for each category for clinically important gastrointestinal bleeding and overt bleeding. We used event rate in the placebo group of the SUP-ICU trial (refer to the guideline outline- PICO #1 supplementary material).
e. SCCM NMA conducted by the guideline panel was not stratified by baseline bleeding risk, which may influence the applicability of the estimates to different patient populations. For Overt GIB: PPI vs. placebo, the direct estimate from conventional MA was 0.62 (95% CrI: 0.43, 0.89), while node splitting analysis yielded 0.65 (95% CrI: 0.37, 1.30), both with moderate certainty. The indirect estimate was 0.30 (95% CrI: 0.12, 0.68), also with moderate certainty, while the NMA estimate was 0.52 (95% CrI: 0.30, 0.81), again with moderate certainty.
f. 2020 updated SR and NMA showed that both PPIs (risk ratio 0.50, 95% credible interval 0.31 to 0.72, moderate certainty) and H2RAs (0.66, 0.48 to 0.89, moderate certainty) probably reduce overt bleeding.
g. Rated down for inconsistency and imprecision. Point estimate was very close to the null, and we switched to rate certainty in little to no effect.
h. Rated down twice for imprecision.

Histamine-2 receptor antagonist vs. No prophylaxis

- Histamine-2 receptor antagonist reduce the risk of GI bleeding. For people with high and highest risk of clinically important GI bleeding, the effect is probably large enough that most people would choose to use them.
- For patients at low risk of clinically significant GI bleeding, H2RAs reduce the absolute risk from 12 per 1000 to 6 per 1000, a difference of 6 fewer per 1000 (OR 0.46, 95% CI 0.27–0.79).

Outcomes	№ of participants (studies) Follow-up	Certainty of the evidence (GRADE)	Relative effect (95% CI)	Anticipated absolute effects* (95% CI)	
				Risk with No SUP	Risk difference with SUP
Clinically important GI bleeding: Histamine-2 receptor antagonist vs. No prophylaxis NMA	1242 (14 RCTs)	⊕⊕⊕◯ Moderate[a]	**OR 0.46** (0.27 to 0.79)[b,c]	Moderate	
				30 per 1,000	**16 fewer per 1,000** (22 fewer to 6 fewer)
				High	
				60 per 1,000	**31 fewer per 1,000** (43 fewer to 12 fewer)
				Highest	
				90 per 1,000	**46 fewer per 1,000** (64 fewer to 18 fewer)
Overt GI bleeding: Histamine-2 receptor antagonist vs. No prophylaxis NMA	2428 (29 RCTs)	⊕⊕⊕◯ Moderate[d]	**OR 0.38** (0.24 to 0.59)[e,f]	Moderate	
				75 per 1,000	**45 fewer per 1,000** (56 fewer to 29 fewer)
				High	
				125 per 1,000	**74 fewer per 1,000** (92 fewer to 47 fewer)
				Highest	
				190 per 1,000	**108 fewer per 1,000** (137 fewer to 68 fewer)
Duration of MV- (days) - Histamine-2 receptor antagonist vs. No prophylaxis NMA	593 (6 RCTs)	⊕⊕◯◯ Low[g,h]	-	The mean duration of MV- (days) - Histamine-2 receptor antagonist vs. No prophylaxis NMA was **0**	MD **0.8 lower** (1.9 lower to 0.3 higher)
Duration of ICU stay- (days) - Histamine-2 receptor antagonist vs. No prophylaxis NMA	436 (4 RCTs)	⊕⊕⊕◯ Moderate[i]	-	The mean duration of ICU stay- (days) - Histamine-2 receptor antagonist vs. No prophylaxis NMA was **0**	MD **0.4 lower** (1.4 lower to 0.6 higher)

a. Rated down by 1 level for imprecision. The 95% confidence interval includes an unimportant difference in clinically important GI bleeding.

b. SCCM NMA conducted by the guideline panel was not stratified by baseline bleeding risk, which may influence the applicability of the estimates to populations at different risk levels. For CIGIB, the comparison between Placebo and H2RA showed a direct estimate from conventional meta-analysis (MA) of 1.84 (95% CrI: 0.74, 4.56) and from node splitting analysis of 1.78 (95% CrI: 0.84, 3.88), both with high certainty of evidence. The indirect estimate was 0.83 (95% CrI: 0.31, 1.70), with moderate certainty, while the network meta-analysis (NMA) estimate was 1.22 (95% CrI: 0.74, 1.98), with low certainty.
c. 2020 updated SR and NMA showed moderate certainty evidence that H2RAs probably reduce CIB (complete: 0.67, 0.48 to 0.94, 20 fewer for high-risk patients).
d. Serious inconsistency I2 = 55%.
e. SCCM NMA conducted by the guideline panel was not stratified by baseline bleeding risk, which may influence the applicability of the estimates to different patient populations. For Overt GIB: Placebo and H2RA showed a direct estimate from conventional meta-analysis (MA) of 1.84 (95% CrI: 0.74, 4.55) and from node splitting analysis of 1.78 (95% CrI: 0.86, 3.88), both with high certainty of evidence. The indirect estimate was 0.84 (95% CrI: 0.31, 1.70), with moderate certainty, while the network meta-analysis (NMA) estimate was 1.22 (95% CrI: 0.74, 1.98), with low certainty.
f. 2020 updated SR and NMA showed that both PPIs (risk ratio 0.50, 95% credible interval 0.31 to 0.72, moderate certainty) and H2RAs (0.66, 0.48 to 0.89, moderate certainty) probably reduce overt bleeding.
g. Both direct and indirect estimate were rated down for risk of bias. Direct estimate was rated down because high risk of bias studies dominated the result. Indirect estimate was rated down because one of the direct estimates (PPIs vs. H2RAs) in the first order loop which contributed to the indirect estimate was rated down for risk of bias.
h. The 95% credible interval includes an important increase and reduction in duration of mechanical ventilation.
i. The 95% credible interval includes an important reduction in length of ICU stay.

Sucralfate vs. No prophylaxis

- Sucralfate may not have an important effect on clinically important GI bleeding.

Outcomes	№ of participants (studies) Follow-up	Certainty of the evidence (GRADE)	Relative effect (95% CI)	Anticipated absolute effects[*] (95% CI)	
				Risk with No SUP	Risk difference with SUP
Clinically important GI bleeding - high risk: Sucralfate vs. No prophylaxis NMA	874 (6 RCTs)	⨁⨁◯◯ Low[a]	**OR 0.76** (0.36 to 1.62)	moderate	
				30 per 1,000	**7 fewer per 1,000** (19 fewer to 18 more)
				High	

				60 per 1,000	**14 fewer per 1,000** (38 fewer to 34 more)
				Highest	
				90 per 1,000	**20 fewer per 1,000** (56 fewer to 48 more)
Overt GI bleeding - high risk: Sucralfate vs. No prophylaxis NMA	900 (7 RCTs)	⨁⨁⨁◯ Moderate[b]	**OR 0.58** (0.30 to 1.11)	Moderate	
				75 per 1,000	**30 fewer per 1,000** (51 fewer to 8 more)
				High	
				125 per 1,000	**48 fewer per 1,000** (84 fewer to 12 more)
				Highest	
				190 per 1,000	**70 fewer per 1,000** (124 fewer to 17 more)

a. Rated down by 2 levels due to very serious imprecision. The 95% confidence interval is wide and includes an important increase and reduction in clinically important GI bleeding.
b. Rated down by 1 level due to serious imprecision. The 95% confidence interval includes no difference in overt GI bleeding.

Intervention	clinically important gastrointestinal bleeding (CIB) for highest risk population– complete PEPTIC analysis (T/S -- per 1000; S/M -- per 1000; M/L -- per 1000)	CIB – High risk population - complete PEPTIC analysis (T/S -- per 1000; S/M -- per 1000; M/L -- per 1000)	CIB- moderate risk population- complete PEPTIC analysis (T/S -- per 1000; S/M -- per 1000; M/L -- per 1000)	Overt gastrointestinal bleeding- highest risk of bleeding (T/S -- per 1000; S/M -- per 1000; M/L -- per 1000)	Overt gastrointestinal bleeding-High risk population (T/S -- per 1000; S/M -- per 1000; M/L -- per 1000)	Overt gastrointestinal bleeding- moderate risk population (T/S -- per 1000; S/M -- per 1000; M/L -- per 1000)	length of ICU stay- complete PEPTIC analysis (Difference -- or more; Difference -- or more; Difference -- or more)	Duration of mechanical ventilation- complete PEPTIC analysis (Difference -- or more; Difference -- or more; Difference -- or more)
Proton pump inhibitor vs placebo	NMA 49 fewer 64 fewer to 31 fewer RR 0.46 (0.29, 0.66) MODERATE	NMA 32 fewer 43 fewer to 20 fewer RR 0.46 (0.29, 0.66) MODERATE	NMA 16 fewer 21 fewer to 10 fewer RR 0.46 (0.29, 0.66) LOW	NMA 95 fewer 131 fewer to 53 fewer RR 0.5 (0.31, 0.72) MODERATE	NMA 62 fewer 86 fewer to 35 fewer RR 0.5 (0.31, 0.72) MODERATE	NMA 37 fewer 52 fewer to 21 fewer RR 0.5 (0.31, 0.72) MODERATE	NMA MD 0.96 (0.86, 1.08) MODERATE	NMA MD 0.97 (0.86, 1.11) MODERATE
histamine-2 receptor antagonists vs placebo	NMA 30 fewer 47 fewer to 5 fewer RR 0.67 (0.48, 0.94) MODERATE	NMA 20 fewer 31 fewer to 4 fewer RR 0.67 (0.48, 0.94) MODERATE	NMA 10 fewer 16 fewer to 2 fewer RR 0.67 (0.48, 0.94) MODERATE	NMA 65 fewer 99 fewer to 21 fewer RR 0.66 (0.48, 0.89) MODERATE	NMA 42 fewer 65 fewer to 14 fewer RR 0.66 (0.48, 0.89)	NMA 25 fewer 39 fewer to 8 fewer RR 0.66 (0.48, 0.89) MODERATE	NMA MD 0.96 (0.85, 1.09) LOW	NMA MD 0.95 (0.84, 1.08) LOW
sucralfate vs placebo	NMA 16 fewer 42 fewer to 26 more RR 0.82 (0.53, 1.29) VERY LOW	NMA 11 fewer 28 fewer to 17 more RR 0.82 (0.53, 1.29) LOW	NMA 5 fewer 9 more to 14 more RR 0.82 (0.53, 1.29) LOW	NMA 0 fewer 74 fewer to 129 more RR 1 (0.61, 1.68) LOW	NMA -- 49 fewer to 85 more RR 1 (0.61, 1.68) LOW	NMA -- 29 fewer to 51 more RR 1 (0.61, 1.68) MODERATE	NMA MD 0.93 (0.81, 1.08) LOW	NMA MD 1 (0.87, 1.14)

NMA link
https://gdt.gradepro.org/presentations/#/nma/nma_question_1f57d3a1-f4e6-43a3-9f1e-f7900d9566e3

Undesirable Effects

How substantial are the undesirable anticipated effects?

JUDGEMENT	RESEARCH EVIDENCE	ADDITIONAL CONSIDERATIONS

JUDGEMENT

○ Trivial
○ Small
○ Moderate
○ Large
● Varies
○ Don't know

RESEARCH EVIDENCE

Proton pump inhibitor vs. No prophylaxis

- PPIs may have little or no effect on mortality.
- PPIs may have no important impact on pneumonia compared with no prophylaxis. The credible intervals include no difference, and the most recent and largest blinded RCT suggested that there may not be a difference in risk of pneumonia between the PPI and placebo groups.
- PPIs may have little or no effect on *C. difficile* infection.
- **REVISE and SUP-ICU trials also suggest that the effect of proton pump inhibitors on mortality may differ according to baseline illness severity.** In the SUP-ICU trial, the within-subgroup effects suggest the possibility of increase in mortality in patients with high illness acuity while the REVISE trial data suggest that the effect may predominantly drive a decrease in mortality in patients with low illness acuity. (8, 7)

ADDITIONAL CONSIDERATIONS

Panel comments

- See comment above, also re. PEPTIC. Some concerns re. undesirable effects due to signals in all three major new trials re. mortality in least/most severely ill patients.
- 0-50 more complications per 1000 seems to be more than trivial, considering pneumonia to be a serious complication. However, there is uncertainty in the evidence, and I do not

Outcomes	№ of participants (studies) Follow-up	Certainty of the evidence (GRADE)	Relative effect (95% CI)	Anticipated absolute effects* (95% CI)	
				Risk with No SUP	Risk difference with SUP
Mortality: Proton pump inhibitor vs. No prophylaxis NMA	4194 (9 RCTs)	⊕⊕⊕◯ Moderate[a]	**OR 1.06** (0.90 to 1.28)[b]	risk per 1000	
				304 per 1,000	**12 more per 1,000** (22 fewer to 55 more)
Mortality: Proton pump inhibitor vs. No prophylaxis Pairwise SRMA NEJM 2024 (including REVISE)	9463 (12 RCTs)	⊕⊕◯◯ Low[c]	**RR 0.99** (0.93 to 1.05)	Study population	
				309 per 1,000	**3 fewer per 1,000** (22 fewer to 15 more)
Pneumonia: Proton pump inhibitor vs. No prophylaxis NMA	3974 (6 RCTs)	⊕⊕◯◯ Low[d,e]	**OR 1.39** (0.98 to 2.10)[f]	risk per 1000	
				162 per 1,000	**50 more per 1,000** (3 fewer to 127 more)
Pneumonia Proton pump inhibitor vs. No prophylaxis Pairwise SRMA NEJM 2024 (including REVISE)	8949 (8 RCTs)	⊕⊕◯◯ Low[g]	**RR 1.00** (0.92 to 1.09)	Study population	
				238 per 1,000	**0 fewer per 1,000** (19 fewer to 21 more)
Clostridium difficile infection-Proton pump inhibitor vs. No prophylaxis NMA	3720 (4 RCTs)	⊕⊕◯◯ Low[h]	**OR 0.82** (0.31 to 2.47)[i]	risk per 1000	
				15 per 1,000	**3 fewer per 1,000** (10 fewer to 21 more)
Clostridium difficile infection-Proton pump inhibitor vs. No prophylaxis Pairwise SRMA NEJM 2024 (including REVISE)	8682 (6 RCTs)	⊕⊕◯◯ Low[h]	**RR 1.20** (0.66 to 2.16)	Study population	
				7 per 1,000	**1 more per 1,000** (2 fewer to 8 more)

feel comfortable making a judgement either way.

- I believe the primary endpoint of the included RCT was bleeding, and all studies are not powered to detect secondary endpoints such as mortality. What about studies comparing PPI vs. H2B? PMID: 35213716, this trial found increased mortality with PPI.
- Even though there is no impact on mortality or LOS, there are some class-related concerns like hypomagnesemia with PPI. I'd consider the side effects are not trivial, but perhaps small.

Key Justifications for the final decision:

· Pneumonia risk appears inconsistent across meta-analyses, but some evidence suggests a clinically relevant increase.
· Mortality risk may differ based on illness severity, requiring careful consideration in high-risk populations.
· *C. difficile* risk remains trivial, but other potential adverse effects (hypomagnesemia, renal dysfunction) lack strong ICU-specific evidence.
· Comparisons between PPIs and H2 blockers will be addressed in PICO 3.

Other Outcomes not reported and rated important by panel	0<br>(0 studies)	-	-	Ventilator-Free Days- Not reported<br>Renal Failure and AKI - Not reported<br>Delirium - Not reported<br>Thrombocytopenia - Not reported

a. Rated down by 1 level for imprecision. The 95% credible interval includes an important increase and reduction in mortality.
b. 2020 updated SR and NMA showed the following for mortality, including data from the PEPTIC trial: risk ratio 1.03, 95% credible interval 0.93 to 1.14, moderate certainty. SCCM NMA conducted by the panel showed similar results: In the PPI vs. placebo comparison, the direct estimate from conventional MA was 1.03 (95% CrI: 0.94, 1.14), while the estimate from node splitting was 1.04 (95% CrI: 0.81, 1.22), both with high certainty. The indirect estimate was 0.98 (95% CrI: 0.80, 1.19), with high certainty, and the NMA estimate was 1.02 (95% CrI: 0.92, 1.40), with moderate certainty. These results indicate that PPI use does not significantly impact overall mortality compared to placebo.
c. Rated down for imprecision and ROB. Because the point estimate was very close to the null, we switched to rate certainty in little to no effect. Uncertainty arose from the subgroup analysis based on disease severity.
d. Rated down by 1 level. We are skeptical of the result because the pooled result including smaller studies conflicts with the evidence from the largest study (SUP-ICU).
e. Rated down by 1 level. The 95% credible interval includes no difference in pneumonia.
f. 2020 updated SR and NMA showed the following for pneumonia, including data from the PEPTIC trial: risk ratio 1.08, 95% credible interval 0.88 to 1.45, low certainty. SCCM NMA conducted by the panel showed similar results: In the PPI vs. placebo comparison, the direct estimate from conventional MA was 1.02 (95% CrI: 0.88, 1.19), while the estimate from node splitting was 1.05 (95% CrI: 0.82, 1.48), both with high certainty. The indirect estimate was 1.39 (95% CrI: 0.93, 2.07), with high certainty, and the NMA estimate was 1.14 (95% CrI: 0.93, 1.54), with moderate certainty. These results indicate that PPI use does not significantly impact pneumonia risk compared to placebo, although there is some uncertainty in indirect estimates.
g. Rated down twice for imprecision. Because the point estimate was very close to the null, we switched to rate certainty in little to no effect.
h. Rated down twice for imprecision.
i. 2020 updated SR and NMA showed the following for CDI, including data from the PEPTIC trial: risk ratio 0.82, 95% credible interval 0.34 to 2.21, moderate certainty. SCCM NMA conducted by the panel showed similar results: the comparison between PPI and placebo showed a direct estimate from conventional MA of 0.73 (95% CrI: 0.42, 1.26). However, the effect was not estimable using node splitting analysis, and there were no available indirect estimates. The certainty of evidence for the direct estimate was moderate, while the NMA was also not estimable, leading to moderate overall certainty.

Histamine-2 receptor antagonist vs. No prophylaxis

- H2RAs probably do not have an important effect on mortality.

- H2RA may have no important impact on pneumonia compared with no prophylaxis.

Outcomes	№ of participants (studies) Follow-up	Certainty of the evidence (GRADE)	Relative effect (95% CI)	Anticipated absolute effects[*] (95% CI)	
				Risk with No SUP	Risk difference with SUP
Mortality- Histamine-2 receptor antagonist vs. No prophylaxis NMA	1835 (22 RCTs)	⨁⨁⨁◯ Moderate[a]	**OR 0.96** (0.79 to 1.19)[b]	risk per 1000	
				304 per 1,000	**9 fewer per 1,000** (47 fewer to 38 more)
Pneumonia: Histamine-2 receptor antagonist vs. No prophylaxis NMA	1159 (11 RCTs)	⨁⨁◯◯ Low[c,d]	**OR 1.26** (0.89 to 1.85)[e]	risk per 1000	
				162 per 1,000	**34 more per 1,000** (15 fewer to 101 more)
Clostridium difficile infection- Histamine-2 receptor antagonist vs. No prophylaxis NMA	0 (0 non-randomized studies)	⨁◯◯◯ Very low[f,g]	-	Indirect estimate was rated down because one of the direct estimates (PPIs versus H2RAs) in the first order loop which contributed to the indirect estimate was rated down for risk of bias. No direct evidence. Odds ratio 0.94 (CI 95% 0.06 — 14.99). Difference: **0 fewer per 1000** (CI 95% 14 fewer — 226 more)	

a. Rated down by 1 level for imprecision. The 95% credible interval includes an important increase and reduction in mortality.
b. 2020 updated SR and NMA showed the following for mortality, including data from the PEPTIC trial: risk ratio 0.98, 95% credible interval 0.89 to 1.08, moderate certainty. SCCM NMA conducted by the panel showed similar results: Placebo and H2RA demonstrated a direct estimate from conventional MA of 1.05 (95% CrI: 0.87, 1.27) and from node splitting analysis of 1.06 (95% CrI: 0.88, 1.25), with high certainty of evidence. The indirect estimate was 1.02 (95% CrI: 0.85, 1.23), with high certainty, while the network meta-analysis (NMA) estimate was 1.04 (95% CrI: 0.93, 1.16), with moderate certainty.
c. Rated down by 1 level. We are skeptical of the result because the pooled result including smaller studies conflicts with the evidence from the largest study (SUP-ICU).
d. Rated down by 1 level. The 95% credible interval includes an important increase and reduction in pneumonia.
e. 2020 updated SR and NMA showed the following for pneumonia, including data from the PEPTIC trial (risk ratio 1.07, 95% credible interval 0.85 to 1.37, low certainty, low certainty). SCCM NMA conducted by the panel showed similar results: Placebo and H2RA demonstrated a direct estimate from conventional meta-analysis) of 0.89 (95% CrI: 0.60, 1.33) and from node splitting analysis of 0.86 (95% CrI: 0.62, 1.23), both with high certainty of evidence. The indirect estimate was 1.09 (95% CrI: 0.72, 1.54), with high certainty, while the network meta-analysis (NMA) estimate was 0.96 (95% CrI: 0.73, 1.21), with moderate certainty. These findings suggest no significant difference in pneumonia risk between H2RA and placebo.

f. Indirect estimate was rated down because one of the direct estimates (PPIs vs. H2RAs) in the first order loop which contributed to the indirect estimate was rated down for risk of bias. No direct evidence.
g. The 95% credible interval is very wide and includes an important increase and reduction in *C. difficile* infection.

Sucralfate vs. No prophylaxis

- Whether sucralfate has an effect on the risk of death or not is very uncertain.
- Sucralfate may have no important impact on pneumonia compared with no prophylaxis.

Outcomes	№ of participants (studies) Follow-up	Certainty of the evidence (GRADE)	Relative effect (95% CI)	Anticipated absolute effects[*] (95% CI)	
				Risk with No SUP	Risk difference with SUP
Mortality- Sucralfate vs. No prophylaxis NMA	872 (6 RCTs)	⨁◯◯◯ Very low[a]	**OR 0.89** (0.71 to 1.14)[b]	risk per 1000	
				304 per 1,000	**24 fewer per 1,000** (67 fewer to 28 more)
Pneumonia: Sucralfate vs. No prophylaxis NMA	370 (3 RCTs)	⨁⨁◯◯ Low[c,d]	**OR 0.85** (0.56 to 1.33)[e]	Risk per 1000	
				162 per 1,000	**21 fewer per 1,000** (64 fewer to 43 more)

a. The 95% credible interval is very wide and includes an important increase and reduction in mortality.
b. 2020 updated SR and NMA showed the following for mortality, including data from the PEPTIC trial: risk ratio 0.93, 95% credible interval 0.8 to 1.07, very low certainty. SCCM NMA conducted by the panel showed similar results: in the sucralfate vs. placebo comparison, the direct estimate from conventional MA was 0.89 (95% CrI: 0.64, 1.24), while node splitting analysis yielded 0.89 (95% CrI: 0.64, 1.24), both with high certainty. The indirect estimate was 0.95 (95% CrI: 0.77, 1.17), also with high certainty, while the NMA estimate was 0.93 (95% CrI: 0.78, 1.11), with low certainty.
c. Both direct and indirect estimates were rated down for risk of bias. Direct estimate was rated down because high risk of bias studies dominated the result. Indirect estimate was rated down because the direct estimates (H2RAs vs. placebo and H2RAs vs. sucralfate) in the first order loop which contributed to the indirect estimate were rated down for risk of bias.
d. Rated down by 1 level. The 95% credible interval includes an important increase and reduction in pneumonia.
e. 2020 updated SR and NMA showed the following for pneumonia, including data from the PEPTIC trial: risk ratio 0.93, 95% credible interval 0.65 to 1.38, low certainty, low certainty. SCCM NMA conducted

by the panel showed similar results: in the sucralfate vs. placebo comparison, the direct estimate from conventional MA was 1.49 (95% CrI: 0.81, 2.73), while node splitting analysis yielded 1.47 (95% CrI: 0.98, 2.78), both with high certainty. The indirect estimate was 0.74 (95% CrI: 0.3, 1.01), also with high certainty, while the NMA estimate was 0.84 (95% CrI: 0.64, 1.14), with very low certainty. These findings suggest a potential increased risk of pneumonia with sucralfate, though the confidence intervals are wide, indicating uncertainty.

Intervention	mortality–complete PEPTIC analysis<br>T/S 12 per 1000<br>S/M 30 per 1000<br>M/L 60 per 1000	Pneumonia<br>T/S 176 per 1000<br>S/M -- per 1000<br>M/L -- per 1000	Clostroides difficile infection – complete PEPTIC analysis<br>T/S -- per 1000<br>S/M -- per 1000<br>M/L -- per 1000
Proton pump inhibitor vs placebo	NMA<br>**9 more**<br>21 fewer to 43 more<br>**RR 1.03**<br>(0.93, 1.14)<br>MODERATE	NMA<br>**13 more**<br>19 fewer to 73 more<br>**RR 1.08**<br>(0.88, 1.45)<br>LOW	NMA<br>**3 fewer**<br>10 fewer to 18 more<br>**RR 0.82**<br>(0.34, 2.21)<br>MODERATE
histamine-2 receptor antagonists vs placebo	NMA<br>**6 fewer**<br>33 fewer to 24 more<br>**RR 0.98**<br>(0.89, 1.08)	No data	NMA<br>**1 more**<br>11 fewer to 51 more<br>**RR 1.08**<br>(0.28, 4.43)<br>LOW
sucralfate vs placebo	NMA<br>**21 fewer**<br>61 fewer to 21 more<br>**RR 0.93**<br>(0.8, 1.07)<br>VERY LOW	No data	No data

NMA link
https://gdt.gradepro.org/presentations/#/nma/nma_question_1f57d3a1-f4e6-43a3-9f1e-f7900d9566e3

Certainty of evidence

What is the overall certainty of the evidence of effects?

JUDGEMENT	RESEARCH EVIDENCE	ADDITIONAL CONSIDERATIONS
○ Very low<br>○ Low<br>● Moderate<br>○ High<br>○ No included studies	The overall certainty of evidence is moderate to low when balancing desirable and undesirable effects.	• As a rule of thumb, certainty of evidence should not be higher than the lowest quality of evidence of any critical outcome.<br>• Similar to the above comment, the effect on bleeding is evident. A larger sample size or meta-analysis could show benefits in reducing adverse events.<br>• Key Justifications for final decisions: Multiple large RCTs (>6,000 patients) support SUP use, providing a strong evidence base. Despite some inconsistencies, the certainty of evidence aligns with SCCM guideline ratings (low to moderate), recognizing variability across subgroups but maintaining confidence in core trial data.

Outcomes	Importance	Certainty of the evidence (GRADE)
Clinically important GI bleeding: Proton pump inhibitor vs. No prophylaxis NMA of RCTs	CRITICAL	⊕⊕⊕◯ Moderate[a]
Clinically important GI bleeding: Proton pump inhibitor vs. No prophylaxis Pairwise SRMA NEJM 2024 (including REVISE)	CRITICAL	⊕⊕⊕⊕ High
Clinically important GI bleeding: Histamine-2 receptor antagonist vs. No prophylaxis NMA	CRITICAL	⊕⊕⊕◯ Moderate[a]
Overt GI bleeding: Proton pump inhibitor vs. No prophylaxis NMA	CRITICAL	⊕⊕⊕⊕ High
Overt GI bleeding: Proton pump inhibitor vs. No prophylaxis Pairwise SRMA NEJM 2024 (including REVISE)	CRITICAL	⊕⊕⊕⊕ High
Overt GI bleeding: Histamine-2 receptor antagonist vs. No prophylaxis NMA	CRITICAL	⊕⊕⊕◯ Moderate[b]
Mortality: Proton pump inhibitor vs. No prophylaxis Pairwise SRMA NEJM 2024 (including REVISE)	CRITICAL	⊕⊕◯◯ Low[c]
Pneumonia: Proton pump inhibitor vs. No prophylaxis NMA	CRITICAL	⊕⊕◯◯ Low[d,e]
Pneumonia Proton pump inhibitor vs. No prophylaxis Pairwise SRMA NEJM 2024 (including REVISE)	CRITICAL	⊕⊕◯◯ Low[f]
Pneumonia: Histamine-2 receptor antagonist vs. No prophylaxis NMA	CRITICAL	⊕⊕◯◯ Low[d,g]

Clostridium difficile infection-Proton pump inhibitor vs. No prophylaxis NMA	CRITICAL	⊕⊕◯◯<br>Low[h]
Clostridium difficile infection-Proton pump inhibitor vs. No prophylaxis Pairwise SRMA NEJM 2024 (including REVISE)	CRITICAL	⊕⊕◯◯<br>Low[h]
Clostridium difficile infection- Histamine-2 receptor antagonist vs. No prophylaxis NMA	CRITICAL	⊕◯◯◯<br>Very low[i,j]

a. Rated down by 1 level for imprecision. The 95% confidence interval includes an unimportant difference in clinically important GI bleeding.
b. Serious inconsistency I2 = 55%.
c. Rated down for imprecision and ROB. Because the point estimate was very close to the null, we switched to rate certainty in little to no effect. There is uncertainty resulted from subgroup analysis based on disease severity.
d. Rated down by 1 level. We are skeptical of the result because the pooled result including smaller studies conflicts with the evidence from the largest study (SUP-ICU).
e. Rated down by 1 level. The 95% credible interval includes no difference in pneumonia.
f. Rated down twice for imprecision. Because the point estimate was very close to the null, we switched to rate certainty in little to no effect.
g. Rated down by 1 level. The 95% credible interval includes an important increase and reduction in pneumonia.
h. Rated down twice for imprecision.
i. Indirect estimate was rated down because one of the direct estimates (PPIs vs. H2RAs) in the first order loop which contributed to the indirect estimate was rated down for risk of bias. No direct evidence.
j. The 95% credible interval is very wide and includes an important increase and reduction in *C. difficile* infection.

Values

Is there important uncertainty about or variability in how much people value the main outcomes?

JUDGEMENT	RESEARCH EVIDENCE	ADDITIONAL CONSIDERATIONS
○ Important uncertainty or variability<br>● Possibly important uncertainty or	**Literature Review on Patient Values and Preferences for Gastric Acid Suppression**<br>We searched MEDLINE, EMBASE, and PsycINFO to identify literature on how much critically ill patients value the prevention of GIB and qualitative data that may inform the decision to use or avoid gastric acid suppressants for GI bleeding prevention.	"Possibly Important Uncertainty or Variability"<br><br>**Key Justifications for the Decision:**<br>Significant individual variability exists in how patients weigh immediate GI bleeding

<table>
<tr><td>variability
○ Probably no important uncertainty or variability
○ No important uncertainty or variability</td><td>Findings
1 study identified: SUP-ICU Trial: 1-Year Outcomes, Resource Use, and Employment Status. (3)
The study found that while pantoprazole reduced GI bleeding in the ICU, it did not improve long-term survival, healthcare costs, or employment status.
This raises important patient-centered concerns: is it worth exposing patients to SUP if long-term outcomes remain unchanged?
Our perspectives:
Patients would consider both the benefits and risks of acid suppression to be small.
There is likely significant variability among patients in how they weigh the importance of bleeding prevention.
Most patients would require a reduction of clinically important bleeding by approximately 15 to 20 per 1000 patients to opt for acid suppression. From patient and family perspective, preventing an immediate complication like GI bleeding is still valuable.
Patient values for outcomes may vary substantially, making individualized decision-making important.
If SUP does not improve long-term employment status, policymakers may question cost-effectiveness in universal administration.</td><td>prevention versus long-term survival and health status.
Pantoprazole reduced GI bleeding but did not improve post-ICU employment or survival, raising concerns about whether patients would prioritize this intervention.
Policymakers may question cost-effectiveness for universal administration, supporting a risk-stratified approach.</td></tr>
<tr><td colspan="3">Balance of effects
Does the balance between desirable and undesirable effects favor the intervention or the comparison?</td></tr>
<tr><td>JUDGEMENT</td><td>RESEARCH EVIDENCE</td><td>ADDITIONAL CONSIDERATIONS</td></tr>
<tr><td>○ Favors the comparison
○ Probably favors the comparison
○ Does not favor either the intervention or the comparison
● Probably</td><td>Probably favors the use of SUP compared to no SUP. For people with high risk of clinically important GI bleeding, the effect is probably large enough that most people would choose to use them.
While SUP reduces clinically important bleeding (CIB) and overt GI bleeding, it does not substantially impact other key clinical outcomes, and both bleeding and its consequences can be treated efficiently when they occur. Given these considerations, routine PPI use in low-risk patients remains questionable
Evidence from SUP-ICU, REVISE, and PEPTIC trials suggests that the effect of proton pump inhibitors (PPIs) on mortality varies by illness severity. While PPIs may provide a mortality benefit in less</td><td>In general, yes, but some caution re. mortality and severity of illness seems warranted.
In patients with risk factors for GIB
One panel member favors intervention</td></tr>
</table>

favors the intervention<br>o Favors the intervention<br>o Varies<br>o Don't know	**severely ill patients**, there is a potential **increase in mortality among the most critically ill**. This pattern has been consistently observed across major trials, raising concerns about **universal PPI use.**	

Resources required

JUDGEMENT	RESEARCH EVIDENCE	ADDITIONAL CONSIDERATIONS
o Large costs<br>o Moderate costs<br>o Negligible costs and savings<br>o Moderate savings<br>o Large savings<br>● Varies<br>o Don't know	PPIs and H2RAs are generally inexpensive compared to the overall expense of an ICU stay and are widely available. H2RAs may be slightly less expensive, and health providers may prefer it in some resource-limited settings. (5)	**Panel Comments**<br><br>I suggest we use the cost analyses Halling et al.<br><br>One panel member chose large savings

Category	Proton Pump Inhibitors (PPIs)	Histamine-2 Receptor Antagonists (H2RAs)
Medication Routine	- Typically administered once per day<br>- Includes esomeprazole, Pantoprazole, and omeprazole, available in oral and IV formulations.<br>- **No dose adjustment required for renal or liver dysfunction.**	- Typically administered two or three times per day<br>- **Dosing adjustments required for renal impairment.**<br>- Available in oral and IV formulations.
Interactions	- may alter absorption of medications affected by gastric pH changes<br>- Likely interactions with clopidogrel, HIV protease inhibitors, methotrexate, magnesium	- Cimetidine inhibits P450 enzymes but is rarely used for prophylaxis<br>- Ranitidine and famotidine have negligible effects on cytochrome system<br>- H2RAs may alter absorption of pH-dependent medications, but likely less than PPIs
**Costs & Access**	**- Inexpensive**<br>**- IV formulations are usually more expensive than enteral forms**<br>**- Costs vary by specific agent**	**- Inexpensive**<br>**- IV formulations are usually more expensive than enteral forms**<br>**- Costs vary by specific agent**

Cost/Resource Use Measure	Difference	Key Finding
**Total healthcare costs (€)**	**€1,953.67 higher** (95% CI: −€2,992 to €6,899)	No significant cost savings with pantoprazole.
**Bed days (all hospital stays, median)**	**+1.37 days** (95% CI: −2.57 to 5.32)	No difference in hospital stay duration.
**General hospital stays (median visits)**	**+0.64 visits** (95% CI: −0.47 to 1.74)	No significant difference.
**Psychiatric hospital stays (median visits)**	**−0.63 visits** (95% CI: −1.30 to 0.04)	No significant change.
**Primary healthcare visits (GP, specialists, etc.)**	**−0.34 visits** (95% CI: −2.65 to 1.98)	No major difference.
**Contacts with general practitioners (GPs, median)**	**−0.56 contacts** (95% CI: −1.98 to 0.87)	No significant difference.
**Readmission with GI bleeding (%)**	**−0.005 percentage points** (95% CI: −0.016 to 0.005)	No impact on post-discharge GI bleeding risk.
**Employment rate at 1-year (weeks worked)**	**−0.178 weeks** (95% CI: −0.390 to 0.034)	No improvement in employment outcomes.
**Sickness leave at 1-year (weeks)**	**−8.84 weeks** (95% CI: −43 to 25)	No meaningful difference in return to work.
**Salary (€)**	**−€787.19** (95% CI: −€1,799 to €224)	No significant salary improvement.
**90-day mortality (%)**	**No significant change**	No mortality benefit from pantoprazole.
**1-year mortality (%)**	**No significant change**	No long-term survival advantage.

Certainty of evidence of required resources

What is the certainty of the evidence of resource requirements (costs)?

<table>
<tr><th>JUDGEMENT</th><th>RESEARCH EVIDENCE</th><th>ADDITIONAL CONSIDERATIONS</th></tr>
<tr>
<td>○ Very low
● Low
○ Moderate
○ High
○ No included studies</td>
<td>The SUP-ICU trial cost analyses (3) found that while pantoprazole reduced clinically important GI bleeding in patients in the ICU, this did not lead to cost savings, reduced healthcare resource use, or improved long-term outcomes. Total healthcare costs were slightly higher in the pantoprazole group, and there was no significant difference in hospital stays, primary care visits, or psychiatric care. Similarly, employment outcomes and salary levels remained unchanged, suggesting no long-term economic benefit. Additionally, pantoprazole use did not improve mortality at 90 days or 1 year. These findings reinforce that routine use of pantoprazole for stress ulcer prophylaxis may not be justified in patients in the ICU, especially those at lower risk of GI bleeding.

Indirect evidence in non-ICU
The study by Tan et al. (2016) investigated the incidence and cost implications of continuing stress ulcer prophylaxis (SUP) after patients were discharged from the intensive care unit (ICU). This retrospective analysis highlighted a significant trend of inappropriate continuation of SUP therapy, leading to unnecessary healthcare expenditures.
Key Findings:
High Incidence of Inappropriate SUP Continuation: A substantial number of patients discharged from the ICU remained on SUP without a valid medical indication, suggesting a gap in medication reconciliation processes.
Increased Healthcare Costs: The unwarranted extension of SUP therapy post-ICU discharge resulted in considerable additional costs, emphasizing the need for targeted interventions to curb unnecessary medication use.
These findings highlight the importance of re-evaluating SUP prescriptions during transitions of care to prevent unnecessary medication use and associated costs.

Additional consideration:
The financial burden of managing GI bleeding in ICUs is substantial, primarily driven by hospitalization and procedural costs. While specific data from Europe and Saudi Arabia are limited, available studies suggest that the average cost per major GI bleeding event ranges from approximately $11,941 to $13,093 in Europe, with hospitalization costs constituting a significant portion. In Saudi Arabia, although direct data on GI bleeding are</td>
<td>I found a study regarding costs of PPI after ICU. Maybe it is not enough to raise the judgment to very low as it involves the time after ICU, not ICU. Tan B, Norman R, Litton E, Heath C, Hawkins DJ, Krishnamurthy R, Sonawane R, Anstey MH. Incidence and cost of stress ulcer prophylaxis after discharge from the intensive care unit: a retrospective study. Crit Care Resusc. 2016 Dec;18(4):270-274. PMID: 27903209.
Halling's study of SUP-ICU is relevant here: https://doi.org/10.1007/s00134-022-06631-2
I suggest we use the cost analyses Halling et al. 2022
Please look for GI bleeding cost of care in the ICU (including length of stay and procedures such as endoscopy)</td>
</tr>
</table>

	lacking, ICU admissions for severe conditions can cost up to **SAR 79,418.30** (approximately USD 21,178) per patient.<br>The cost of endoscopic procedures varies significantly across different regions and healthcare systems. Below is a summary of estimated costs for common endoscopic procedures in **Europe** and **Saudi Arabia**:<br>**1. Europe:**<br>• **Gastroscopy (Upper Endoscopy):** In Germany, the average cost is approximately **€230.56**.<br>• **Colonoscopy:** The average cost in Germany is around **€276.23**.<br>• **Endoscopic Retrograde Cholangiopancreatography (ERCP):** Costs vary based on complexity:<br>• ERCP with papillotomy and plastic stent insertion: **€844.07**<br>• ERCP with self-expanding metal stent: **€1,602.37**<br>• **Private Healthcare Costs in the UK:** At Practice Plus Group, the costs are:<br>• Gastroscopy: **£1,349**<br>• Colonoscopy: **£1,749**<br>• Flexible sigmoidoscopy: **£1,349**<br><br>**2. Saudi Arabia:**<br>• **Capsule Endoscopy:** Specific cost data is not provided; however, the package typically includes surgeon fees, hospitalization, and anesthesia.<br>• **Endoscopic Sleeve Gastroplasty:** The procedure costs between **10,000 to 15,000 SAR**.<br>• **Endoscopy and Colonoscopy Package:** The package is priced at **AED 3,700** and includes:<br>• Gastroenterology consultation<br>• Single biopsy<br>• Anesthesia consultation<br>• Sedation<br>• Recovery room stay<br>• One follow-up with gastroenterology for results (if biopsy is taken)<br>• Routine consumables	

Cost effectiveness

Does the cost-effectiveness of the intervention favor the intervention or the comparison?

JUDGEMENT	RESEARCH EVIDENCE	ADDITIONAL CONSIDERATIONS
○ Favors the comparison<br>○ Probably favors the comparison<br>○ Does not favor either the intervention or the comparison<br>○ Probably favors the intervention<br>○ Favors the intervention<br>● Varies<br>○ No included studies	Probably SUP cost effectiveness given the reduction in CIB. However, variability may exist.<br><br>Secondary cost–consequence analysis of the Danish participants included in the SUP-ICU trial (1-year outcomes, resource use of SUP ICU) showed that use of pantoprazole in patients in the ICU at risk of GI bleeding reduced this risk. However, **this did not translate into improved 1-year outcomes or reduced health care resource use and associated costs.** (3)<br><br>Four pharmacoeconomic analyses have been published each using different assumptions and variables in their decision trees along with different outcome measures for effectiveness. The first (Udeh et al.) compared the cost per bleeding event avoided and included PPIs, H2RAs, and sucralfate. Factors included in the model were drug acquisition costs, consumables and labor costs, costs associated with clinically important bleeding, and costs to evaluate ADEs (diarrhea, thrombocytopenia, and mental status). Enteral omeprazole had the lowest cost-effectiveness ratio, $12,391 per case avoided. The cost-effectiveness ratios for enteral famotidine and sucralfate were $14,752 and $37,881, respectively. The second (Barkun et al.) compared the cost per complication avoided between PPI's and H2RA's. Complications included in this model were bleeding and pneumonia. The cost-effectiveness ratio in this report was $58,699 for PPI and $63,921 for H2RA, favoring PPIs. The third (MacLaren et al.) compared the cost of PPI vs. H2RA using mortality as the effectiveness variable. Factors included in this model were bleeding, pneumonia, and CDI. The cost associated with PPI's and H2RA therapies were $7,802 and $6,707, respectively. Mortality rates were similar between the two groups (3.8%), resulting in a cost saving of $1,095 with H2RA.The fourth (Hammond et al.) compared PPI vs. H2RA using mortality and complication rate for effectiveness. Complications were CIB, pneumonia, and CDI. The costs, complications rate, and mortality rate were $11,249, 22%, and 3.34% for PPIs and $9,039, 17.6%, and 2.5% for H2RAs, favoring H2RA therapy (9)	One panel member chose probably favors the intervention<br>One panel member chose Favors the intervention<br><br>Majority of the panel agreed that cost-effectiveness varies based on:<br>• The agent used.<br>• Post-ICU cost implications (as seen in the Sub-ICU cost analysis).<br><br>Final Decision: Classification of "Varies" retained.

Equity

What would be the impact on health equity?

JUDGEMENT	RESEARCH EVIDENCE	ADDITIONAL CONSIDERATIONS
○ Reduced<br>○ Probably reduced	Probably no impact. However, implementing deprescribing programs could improve equitable use of medications, ensuring that only those who truly need SUP receive it.	**Panel Comments & Debate:**

● Probably no impact<br>○ Probably increased<br>○ Increased<br>○ Varies<br>○ Don't know		• One panel disagreed with “Probably No Impact” and argued that:<br>• ICU complications (e.g., pneumonia, GI bleeding) disproportionately affect vulnerable populations.<br>• In LMIC settings or resource-strained ICUs, one complication could influence decisions on continuing or withdrawing care. Suggested a reclassification to “Varies.”<br>• One panel acknowledged that equity depends on the global vs. local perspective and noted that PPIs are more expensive than sucralfate and H2 blockers, which could impact access in LMICs.<br>• One panel defended “increase equity as the cost is low for the intervention” and emphasized that the guideline targets Saudi Arabia and other medium-to-high-income countries. Marwa raised a concern about this as may lead to universal PPI use, not selective use for high-risk patients.<br>• One panel supported “Probably No Impact,” arguing that SUP is widely available in critical care settings and that pneumonia risk and GI bleeding severity may outweigh cost concerns.<br>• Warned that a “Varies” classification could mislead practitioners into underusing SUP.<br><br>**Final Decision: Retained “Probably No Impact” but included concerns on LMIC access as an additional consideration.**

**Acceptability**<br>Is the intervention acceptable to key interest-holders?		
**JUDGEMENT**	**RESEARCH EVIDENCE**	**ADDITIONAL CONSIDERATIONS**
○ No<br>○ Probably no<br>● Probably yes<br>○ Yes<br>○ Varies<br>○ Don't know	The panel acknowledged variability in practice preferences but overall agreed that clinicians are likely to accept SUP for this population.	One panel chose “yes, acceptable.”

**Feasibility**<br>Is the intervention feasible to implement?		
**JUDGEMENT**	**RESEARCH EVIDENCE**	**ADDITIONAL CONSIDERATIONS**
○ No<br>○ Probably no<br>● Probably yes<br>○ Yes<br>○ Varies<br>○ Don't know	Implementing risk-based SUP strategies is feasible with electronic decision-support tools. Targeted approach to SUP use could ensure only high-risk patients in the ICU receive it, reducing unnecessary use.<br>The pharmacist-led intervention study across 26 ICUs in China provides strong evidence that reducing stress ulcer prophylaxis (SUP) is both feasible and safe. Key findings include:<br><br>• SUP Use Can Be Reduced Without Increasing GI Bleeding:<br>• SUP use was lowered from 49.5% to 45.5% with the intervention.<br>• No significant increase in overt GI bleeding (3.7% vs. 4.0%), proving deprescribing is safe in selected patients.<br>• Structured Interventions Are Effective and Scalable:<br>• The intervention combined guideline-based education, pharmacist-led reviews, and real-time recommendations.<br>• Successfully implemented across multiple hospital settings, making it adaptable for broader use.<br>• Clinician Acceptance is High:<br>• 60.1% of pharmacist recommendations were fully or partially accepted by physicians.<br>• This shows that targeted deprescribing efforts are clinically acceptable and practical.<br>• No Negative Impact on Patient Outcomes:<br>• No significant changes in mortality, hospital-acquired pneumonia, or *C. difficile* infection, supporting safe deprescribing.	One panel chose “yes.”<br><br>Evidence supporting that reducing SUP is feasible:<br>https://doi.org/10.1097/ccm.0000000000006589 (COI: involved in the study).

	This study confirms that reducing unnecessary SUP in ICUs is feasible, widely accepted, and does not compromise patient safety. A structured, risk-based approach can help minimize overuse while maintaining clinical effectiveness.	

SUMMARY OF JUDGEMENTS

	JUDGEMENT						
PROBLEM	No	Probably no	Probably yes	**Yes**		Varies	Don't know
DESIRABLE EFFECTS	Trivial	Small	Moderate	Large		**Varies**	Don't know
UNDESIRABLE EFFECTS	Trivial	Small	Moderate	Large		**Varies**	Don't know
CERTAINTY OF EVIDENCE	Very low	Low	**Moderate**	High			No included studies
VALUES	Important uncertainty or variability	**Possibly important uncertainty or variability**	Probably no important uncertainty or variability	No important uncertainty or variability			
BALANCE OF EFFECTS	Favors the comparison	Probably favors the comparison	Does not favor either the intervention or the comparison	**Probably favors the intervention**	Favors the intervention	Varies	Don't know
RESOURCES REQUIRED	Large costs	Moderate costs	Negligible costs and savings	Moderate savings	Large savings	**Varies**	Don't know
CERTAINTY OF EVIDENCE OF REQUIRED RESOURCES	Very low	**Low**	Moderate	High			No included studies
COST EFFECTIVENESS	Favors the comparison	Probably favors the comparison	Does not favor either the intervention or the comparison	Probably favors the intervention	Favors the intervention	**Varies**	No included studies

	JUDGEMENT						
**EQUITY**	Reduced	Probably reduced	**Probably no impact**	Probably increased	Increased	Varies	Don't know
**ACCEPTABILITY**	No	Probably no	**Probably yes**	Yes		Varies	Don't know
**FEASIBILITY**	No	Probably no	**Probably yes**	Yes		Varies	Don't know

TYPE OF RECOMMENDATION

Strong recommendation against the intervention	Conditional recommendation against the intervention	Conditional recommendation for either the intervention or the comparison	**Conditional recommendation for the intervention**	Strong recommendation for the intervention
○	○	○	●	○

CONCLUSIONS

Recommendation

In critically ill patients with coagulopathy, shock, or chronic liver disease, we suggest using SUP compared to no SUP (conditional recommendation, moderate certainty of evidence).

Remark:

- Patients with coagulopathy, shock, or chronic liver disease are at an increased absolute risk of stress-related clinically important upper gastrointestinal bleeding (UGIB).
- Clinicians may consider mechanically ventilated patients as an "at-risk" population for stress-related UGIB, warranting individualized assessment for SUP initiation.
- Clinicians and patients may opt not to use SUP in low-risk populations (with a 1–2% risk of clinically important GI bleeding).
- Some evidence suggests PPIs may increase mortality in high illness acuity. Given these considerations, a risk-stratified approach is essential. SUP should be reserved for those at the highest risk of GI bleeding, while deprescribing should be considered in low-risk patients to avoid unnecessary exposure and resource waste.

Justification

The provision of stress ulcer prophylaxis over no stress ulcer prophylaxis in patients with risk factors does not reduce mortality but can reduce the incidence of clinically important and overt bleeding. There is no difference in the incidence of pneumonia.

While the majority of panel members supported a conditional recommendation, one panel member favored a strong recommendation consistent with the Portuguese guidelines, citing clarity, ease of implementation, and concern about potential legal implications in malpractice cases where SUP was not used in a high-risk patient. Given the moderate certainty of evidence and variability in clinician and patient preferences, we retained a conditional recommendation.

Subgroup considerations

ICU Subpopulations: Neurocritical Care Patients (10)

- Patients with aneurysmal subarachnoid hemorrhage (aSAH), traumatic brain injury (TBI), ischemic stroke, intracerebral hemorrhage (ICH), spinal cord injury (SCI), or anoxic brain injury.
- Patients with elevated intracranial pressure (ICP) or cerebral vasospasm.

Efficacy of SUP in Neurocritical Care: **(refer to Supplemental Content 2.14. Stress Ulcer Prophylaxis (SUP) in ICU Subpopulations).**

- A meta-analysis of 8 RCTs (829 neurocritical care patients) found that pharmacologic SUP reduced clinically important UGIB (RR, 0.31; 95% CI, 0.20–0.47) despite high bleeding rates (11–33%).
- All-cause mortality was lower with SUP (RR, 0.70; 95% CI, 0.50–0.98), but there was no significant difference in nosocomial pneumonia (RR, 1.14; 95% CI, 0.67–1.94).
- Another meta-analysis of 14 RCTs found that both PPIs and H2RAs significantly reduced UGIB, but neither had a significant impact on mortality or pneumonia rates.

Recommendation:

- Conditional recommendation (very low certainty of evidence): We suggest using pharmacologic SUP in neurocritical care adults to reduce clinically important UGIB compared to no SUP

ICU Subpopulations: Trauma, Burn, and Cardiothoracic Patients: **(refer to Supplemental Content 2.14. Stress Ulcer Prophylaxis (SUP) in ICU Subpopulations).**

- **No high-quality RCTs** have evaluated **patient-centered outcomes** in these specific ICU subgroups.
- Due to the **limited quantity and quality of overall evidence**, no specific recommendations can be made.

Implementation considerations

Stress ulcer prophylaxis should only be administered to patients with risk factors recognizing that not all patients in the ICU will qualify. Standard ICU admission order sets that include medications for stress ulcer prophylaxis should not be pre-checked so their use can be individualized based on the presence of risk factors.

When adding SUP to patients in the ICU, suggest documentation of which risk factor the individual patient must help with de-escalation efforts at care transitions.

Clinical decision support (CDS) from EMR should aid clinicians to identify patients with risk factors for targeted initiation of SUP. In addition, this CDS should help in de-escalation of SUP for patients who no longer have the risk factors.
Medications utilized for SUP should be re-evaluated at the time of transition out of the ICU.

Monitoring and evaluation

Unless there is another indication for gastric acid suppression, **PPIs may interact with antiplatelet therapy (e.g., clopidogrel), potentially increasing cardiovascular risk**. **The clinical significance of these interactions in patients in the ICU remains uncertain**. SUP-ICU trial showed no increase in CV events with PPI.
Specific medications that interact with proton pump inhibitors (e.g., **HIV protease inhibitors atazanavir or nelfinavir)** (2)

Duration of Prophylaxis

- Patients should be reassessed regularly for the continued need for stress ulcer prophylaxis.
- Consider re-evaluation of SUP:
- When the patient's condition changes.
- Upon discharge from the hospital.
- Upon transfer to a different level of care.
- Clinicians should ensure that SUP is stopped when the patient is no longer critically ill or the risk factor triggering prophylaxis is no longer present.

Research priorities

- More research is needed on the outcomes of **SUP in the neurocritical care population** and difference in subgroups if neurocritical care.
- Future research is needed in **non-invasive mechanical ventilation**.
- **Patient values and preferences research** to balance bleeding prevention with potential adverse effects.
- Future research is needed to determine **patient-reported outcomes or microbiome changes** as a mechanism for infection risk.
- Future research is needed on the association between **PPIs and myocardial infarction, stroke, and cardiovascular events, especially in combination with antiplatelet therapy**.
- Applying **machine learning models could help predict which patients are most likely to benefit from SUP** in preventing upper gastrointestinal bleeding while also identifying those at the lowest risk of adverse outcomes. Integrating machine learning into risk stratification and treatment decision-making could optimize patient selection for SUP, improving personalized critical care strategies.

Supplemental Content 2.14 Stress Ulcer Prophylaxis (SUP) in ICU Subpopulations

Neurocritical Care

- **At-Risk Populations:**
  - Patients with aneurysmal subarachnoid hemorrhage (aSAH), traumatic brain injury (TBI), ischemic stroke, intracerebral hemorrhage (ICH), spinal cord injury (SCI), or anoxic brain injury.
  - Patients with elevated intracranial pressure (ICP), cerebral vasospasm, coagulopathy, or renal impairment.
  - Patients on mechanical ventilation, prolonged ICU stay, or receiving anticoagulation therapy.
- **Rationale for SUP:**
  - Neurocritical care patients have unique physiological risks for stress-related gastrointestinal bleeding (UGIB), including gastric acid hypersecretion and impaired mucosal defense mechanisms.
- **Incidence and Risk Factors:**
  - Clinically important GI bleeding (CIB) occurs in approximately 4.9% of patients with aSAH.
  - Common contributing factors include mechanical ventilation, vasopressor use, and anticoagulation therapy.
  - Elevated ICP, cerebral vasospasm, renal impairment, and coagulopathy have been independently associated with increased risk.
- **Efficacy of SUP in Neurocritical Care:** [12,13,14]
  - A meta-analysis of 8 RCTs (829 neurocritical care patients) found that pharmacologic SUP reduced clinically important UGIB (RR, 0.31; 95% CI, 0.20–0.47) despite high bleeding rates (11–33%).
  - All-cause mortality was lower with SUP (RR, 0.70; 95% CI, 0.50–0.98), but there was no significant difference in nosocomial pneumonia (RR, 1.14; 95% CI, 0.67–1.94).
  - Another meta-analysis of 14 RCTs found that both PPIs and H2RAs significantly reduced UGIB, but neither had a significant impact on mortality or pneumonia rates.
- **Clinical Considerations for SUP in Neurocritical Care:**

- Risk-Benefit Analysis:
    - Risk of nosocomial pneumonia is inconclusive, with some studies showing no significant increase.
    - No clear superiority of PPIs over H2RAs in reducing clinically significant UGIB.
    - Limited data on *Clostridioides difficile* infection (CDI) risk in neurocritical care patients.

- **Recommendation:**
  - Conditional recommendation (very low certainty of evidence): We suggest using pharmacologic SUP in neurocritical care adults to reduce clinically important UGIB compared to no SUP.

Evidence profile for neurocritical care patients

Outcomes	Anticipated absolute effects* (95% CI)		Relative effect (95% CI)	№ of participants (studies)	Certainty of the evidence (GRADE)
	Risk with no stress ulcer prophylaxis	Risk with stress ulcer prophylaxis			
Clinically Important Bleeding	Study population		**RR 0.31** (0.19 to 0.52)	779 (7 RCTs)	⊕⊕◯◯ LOW [a,b,c]
	295 per 1,000	**92 per 1,000** (56 to 154)			
Mortality	Study population		**RR 0.71** (0.51 to 1.00)	381 (5 RCTs)	⊕⊕◯◯ LOW [c,d,e]
	301 per 1,000	**213 per 1,000** (153 to 301)			
Pneumonia	Study population		**RR 1.17** (0.80 to 1.70)	521 (4 RCTs)	⊕◯◯◯ VERY LOW [c,d,e,f]
	169 per 1,000	**198 per 1,000** (135 to 287)			
Clinically important bleeding-PPI Alone	Study population		**RR 0.26** (0.08 to 0.90)	345 (2 RCTs)	⊕◯◯◯ VERY LOW [b,c,d,g]
	438 per 1,000	**114 per 1,000** (35 to 394)			
Mortality-PPI Alone	Study population		**RR 0.73** (0.46 to 1.16)	165 (1 RCT)	⊕⊕◯◯ LOW [c,e]
	377 per 1,000	**275 per 1,000** (174 to 438)			
Pneumonia-PPI Alone	Study population		**RR 1.54** (0.75 to 3.17)	165 (1 RCT)	⊕⊕◯◯ LOW [c,e]
	151 per 1,000	**232 per 1,000** (113 to 478)			
Clinically Important Bleeding-H2RA Alone	Study population		**RR 0.34** (0.22 to 0.52)	484 (6 RCTs)	⊕⊕◯◯ LOW [b,c,d]
	288 per 1,000	**98 per 1,000** (63 to 150)			
Mortality-H2RA Alone	Study population		**RR 0.68** (0.41 to 1.13)	216 (4 RCTs)	⊕⊕◯◯ LOW [b,c,d]
	264 per 1,000	**179 per 1,000** (108 to 298)			
Pneumonia-H2RA Alone	Study population		**RR 0.98** (0.46 to 2.08)	356 (3 RCTs)	⊕◯◯◯ VERY LOW [c,d,h,i]
	174 per 1,000	**171 per 1,000** (80 to 362)			

a. Rated down 1 level for ROB as 2/7 studies were at high risk of bias.
b. Rated down 1 level for small sample size and number of events.
c. Publication bias not formally assessed due to the small number of studies.
d. Rated down for 1 level for ROB between the studies.
e. Rated down for small number of events and the CI includes 1.
f. Rated down for wide confidence intervals that include the line of no effect and moderate heterogeneity (I2 42%).

g. Rated down for wide confidence intervals and substantial heterogeneity (I2 87%).
h. Rated down for wide variance in point estimates.
i. Rated down for small number of events and moderate heterogeneity (I2 56%).

Subgroup Consideration: Stress Ulcer Prophylaxis (SUP) in Cardiac Surgery Patients

Patients undergoing cardiac surgery are at risk of gastrointestinal (GI) complications, including stress ulcers, upper GI bleeding (UGIB), and hemorrhagic gastritis. The overall incidence of GI bleeding following cardiac surgery is low (0.45%) but is associated with significant morbidity and mortality. Given the complexity of perioperative management, the role of stress ulcer prophylaxis (SUP) in this subgroup warrants careful consideration.

Key Findings from the Literature (PMID: 22345061 & Related Studies)[15]

Efficacy of PPIs vs. H2 Blockers: A prospective randomized trial comparing PPIs (rabeprazole), H2 blockers (ranitidine), and teprenone in cardiac surgery patients found that PPIs were more effective at preventing gastric complications, active ulcers, and hemorrhagic gastritis. Hemorrhagic gastritis rates:

• PPI group: 2.9%
• H2 blocker group: 15.7%
• Teprenone group: 22.9% (p = 0.0003)

Active ulcers:

• PPI group: 4.3%
• H2 blocker group: 21.4%
• Teprenone group: 28.6% (p = 0.0001)
• The study concluded that PPIs significantly reduced the incidence of stress ulceration following cardiac surgery.

Pneumonia Risk & Other Adverse Effects

• A meta-analysis associated gastric acid suppression (PPI & H2 blockers) with an increased risk of pneumonia, with an odds ratio of 1.27 (95% CI 1.11–1.46).
• Routine SUP was not linked to adverse cardiovascular events when co-administered with clopidogrel in cardiac patients undergoing coronary artery bypass grafting (CABG).
• Some studies showed no significant difference between PPIs and H2 blockers in reducing UGIB in cardiac surgery, while others demonstrated a marginal benefit of PPIs.
• Clinical Outcomes & Risk Factors for UGIB
• Older age, prolonged cardiopulmonary bypass (CPB), prolonged hypotension, and re-operation were identified as independent risk factors for GI bleeding post-cardiac surgery.
• Non-pulsatile flow and inflammatory responses post-cardiac surgery may contribute to stress ulcer formation.
• A study of 9199 patients undergoing CPB found that 34.3% of patients who developed UGIB died, compared to 0% mortality in the control group.

Cost & Implementation Considerations

• Cost-effectiveness of routine SUP post-cardiac surgery remains unclear.
• Some cost-analysis studies found no significant hospital cost savings with routine SUP.
• The risk-benefit balance should consider individual patient factors (e.g., GI bleeding risk, pneumonia risk, and overall prognosis).

Guideline Implications for Cardiac Surgery Subgroup

• Conditional Recommendation: In cardiac surgery patients, particularly those undergoing CABG, valve replacement, or prolonged CPB, stress ulcer prophylaxis (SUP) should be considered
• Certainty of Evidence: Low to moderate

Remarks:

• PPIs are more effective than H2 blockers at reducing UGIB but may increase pneumonia risk.
• Patient-specific risk factors (age, reoperation, prolonged CPB, and hemodynamic instability) should guide SUP decisions.
• Routine, universal SUP is not warranted for all cardiac surgery patients but may be appropriate in high-risk groups.
• Clinicians should evaluate the need for continued acid suppression postoperatively, particularly in long-term ICU stays.

Supplemental Content 2.15 AMSTAR-2 Assessment of Systematic Reviews for PICO 2

Question		Huang H-B 2018 (1)	Reynolds 2019 (2)
1.	Did the research questions and inclusion criteria for the review include the components of PICO?	Yes	Yes
2.	Did the report of the review contain an explicit statement that the review methods were established prior to the conduct of the review and did the report justify any significant deviations from the protocol?	No<br>Does not explicitly state protocol registration or deviations from a protocol.	Yes<br>PROSPERO (ID CRD 42017079581).
3.	Did the review authors explain their selection of the study designs for inclusion in the review?	Yes	Yes
4.	Did the review authors use a comprehensive literature search strategy?	Yes	Yes
5.	Did the review authors perform study selection in duplicate?	Yes	Yes
6.	Did the review authors perform data extraction in duplicate?	Yes	Yes
7.	Did the review authors provide a list of excluded studies and justify the exclusions?	Yes	No
8.	Did the review authors describe the included studies in adequate detail?	Yes	Yes
9.	Did the review authors use a satisfactory technique for assessing the RoB in individual studies that were included in the review?	Yes	Yes
10.	Did the review authors report on the sources of funding for the studies included in the review?	No	No
11.	If meta-analysis was performed, did the review authors use appropriate methods for statistical combination of results?	Yes	Yes

12.	If meta-analysis was performed, did the review authors assess the potential impact of RoB in individual studies on the results of the meta-analysis or other evidence synthesis?	Yes	Yes
13.	Did the review authors account for RoB in individual studies when interpreting/discussing the results of the review?	Partially Yes<br>Limitations due to RoB and small sample sizes are acknowledged but not deeply integrated into the interpretation of findings.	Yes
14.	Did the review authors provide a satisfactory explanation for, and discussion of, any heterogeneity observed in the results of the review?	Yes	Yes
15.	If quantitative synthesis was performed, did the review authors carry out an adequate investigation of publication bias (small study bias) and discuss its impact on the results of the review?	No<br>Authors stated publication bias assessment (e.g., funnel plots) was not done due to <10 studies.	No<br>No funnel plot or other formal assessment of publication bias mentioned.
16.	Did the review authors report any potential sources of conflict of interest, including any funding they received for conducting the review?	Yes	Yes

PICO =patient, intervention, comparator, outcome; RoB = risk of bias

Supplemental Content 2.16 Characteristics of Systematic Reviews Evaluated for PICO 2

Study	Date of Search and Databases Examined	# Trials and # participants included	Population	Group 1: SUP<br>Group 2: Comparator	Main findings
Huang H-B 2018 (1)	Through September 30, 2017; PubMed, Embase, Cochrane Central	7 RCTs; 889 participants	Adult patients in the ICU (≥18 years) receiving enteral nutrition	**Group 1: Pharmacologic SUP (PPIs, H2RAs, sucralfate)**<br>**Group 2: Placebo or no prophylaxis**.	**GI Bleeding**: No significant difference (RR 0.80; 95% CI: 0.49–1.31)<br>**Mortality**: No significant difference (RR 1.21; 95% CI: 0.94–1.56)<br>**Hospital-Acquired Pneumonia (HAP)**: Increased risk with SUP (RR 1.53; 95% CI: 1.04–2.27)<br>**C. difficile Infection**: No significant difference (RR 0.89; 95% CI: 0.25–3.19)<br>**ICU Length of Stay**: No significant difference (MD 0.04 days; 95% CI: -0.79 to 0.87)<br>**Duration of Mechanical Ventilation**: No significant difference (MD 0.38 days; 95% CI: -1.48 to 0.72)
Reynolds PM, MacLaren R 2019 (2)	Last search in April 2018<br>MEDLINE, Embase; additional hand-searching of references, guidelines, and conference abstracts	34 randomized controlled trials<br>3220 critically ill adult patients	Critically ill adults in the ICU (medical, surgical/trauma, neurosurgical populations)	**SUP (Stress Ulcer Prophylaxis using PPIs or H2RAs) vs. placebo, control, no therapy, or enteral nutrition**	Studies were subcategorized on the premise of the use of enteral nutrition in most of the studied patients and the most prevalent ICU population included in each study (medical, surgery/trauma, neurosurgical, or burn).<br>**Subgroup Analyses**: **seven trials permitted or encouraged the use of enteral nutrition (960 participants).** SUP maintained a nonsignificant reduction in CIB regardless of the use of enteral nutrition (relative risk 0.57 with nutrition, relative risk 0.39 without nutrition; 95% CI 0.33–1.0, p=0.05).

Supplemental Content 2.17 Updated Forest Plots for PICO 2

CIB

Study or Subgroup	SUP Events	SUP Total	No SUP Events	No SUP Total	Weight	Risk Ratio IV, Random, 95% CI	Year
Van den Berg 1985	5	14	1	14	8.5%	5.00 [0.67, 37.51]	1985
Apte 1992	5	16	6	18	36.2%	0.94 [0.35, 2.49]	1992
Ben-menachem 1994	10	200	6	100	35.8%	0.83 [0.31, 2.23]	1994
Lin 2016	0	60	1	60	3.4%	0.33 [0.01, 8.02]	2016
Selvanderan 2016	0	106	0	108		Not estimable	2016
Alhazzani 2017	3	49	2	42	11.4%	1.29 [0.23, 7.33]	2017
El-Kersh 2017	1	55	1	47	4.6%	0.85 [0.05, 13.29]	2017
GÜNDOĞAN 2020	0	152	0	148		Not estimable	2020
**Total (95% CI)**		**652**		**537**	**100.0%**	**1.03 [0.57, 1.86]**	
Total events	24		17				

Heterogeneity: Tau² = 0.00; Chi² = 3.14, df = 5 (P = 0.68); I² = 0%
Test for overall effect: Z = 0.11 (P = 0.91)

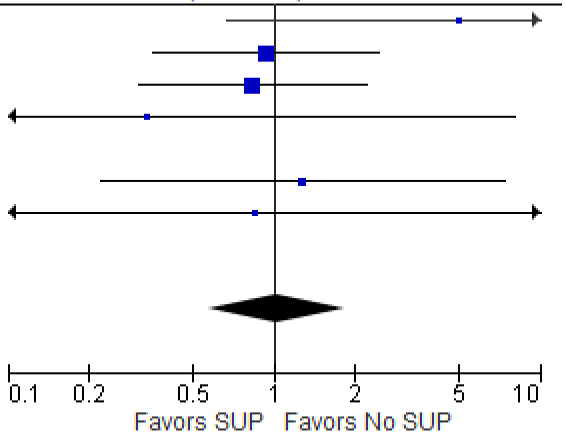

Overt GIB

Study or Subgroup	SUP Events	SUP Total	No SUP Events	No SUP Total	Weight	Risk Ratio IV, Random, 95% CI	Year
Van den Berg 1985	5	14	1	14	7.1%	5.00 [0.67, 37.51]	1985
Apte 1992	5	16	6	18	30.0%	0.94 [0.35, 2.49]	1992
Ben-menachem 1994	10	200	6	100	29.6%	0.83 [0.31, 2.23]	1994
Lin 2016	0	60	5	60	3.5%	0.09 [0.01, 1.61]	2016
Selvanderan 2016	3	106	6	108	15.5%	0.51 [0.13, 1.98]	2016
Alhazzani 2017	1	49	1	42	3.8%	0.86 [0.06, 13.29]	2017
El-Kersh 2017	1	55	1	47	3.8%	0.85 [0.05, 13.29]	2017
Nourian 2018	1	25	1	25	3.9%	1.00 [0.07, 15.12]	2018
GÜNDOĞAN 2020	1	152	0	148	2.8%	2.92 [0.12, 71.15]	2020
**Total (95% CI)**		**677**		**562**	**100.0%**	**0.88 [0.51, 1.50]**	
Total events	27		27				

Heterogeneity: Tau² = 0.00; Chi² = 6.45, df = 8 (P = 0.60); I² = 0%
Test for overall effect: Z = 0.47 (P = 0.64)

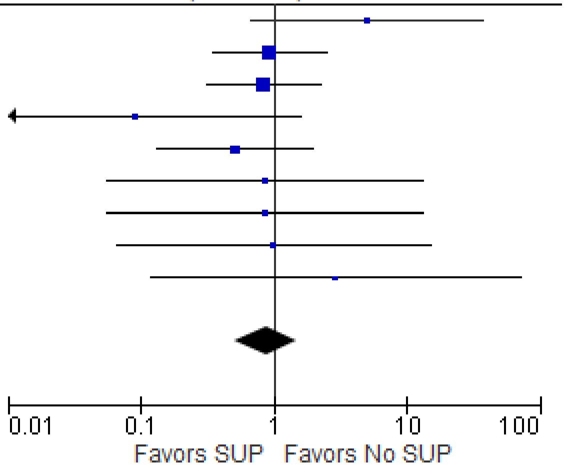

Mortality

Study or Subgroup	SUP Events	SUP Total	No SUP Events	No SUP Total	Weight	Risk Ratio IV, Random, 95% CI	Year
Apte 1992	11	16	7	18	11.4%	1.77 [0.91, 3.44]	1992
Ben-menachem 1994	45	200	19	100	22.1%	1.18 [0.73, 1.91]	1994
Lin 2016	2	60	0	60	0.6%	5.00 [0.25, 102.00]	2016
Selvanderan 2016	30	106	25	108	24.2%	1.22 [0.77, 1.93]	2016
Alhazzani 2017	17	49	13	42	14.4%	1.12 [0.62, 2.03]	2017
El-Kersh 2017	7	55	8	47	5.8%	0.75 [0.29, 1.91]	2017
Nourian 2018	8	32	5	25	5.2%	1.25 [0.47, 3.35]	2018
GÜNDOĞAN 2020	24	152	19	148	16.3%	1.23 [0.70, 2.15]	2020
**Total (95% CI)**		**670**		**548**	**100.0%**	**1.23 [0.98, 1.54]**	
Total events	144		96				

Heterogeneity: Tau² = 0.00; Chi² = 3.17, df = 7 (P = 0.87); I² = 0%
Test for overall effect: Z = 1.78 (P = 0.07)

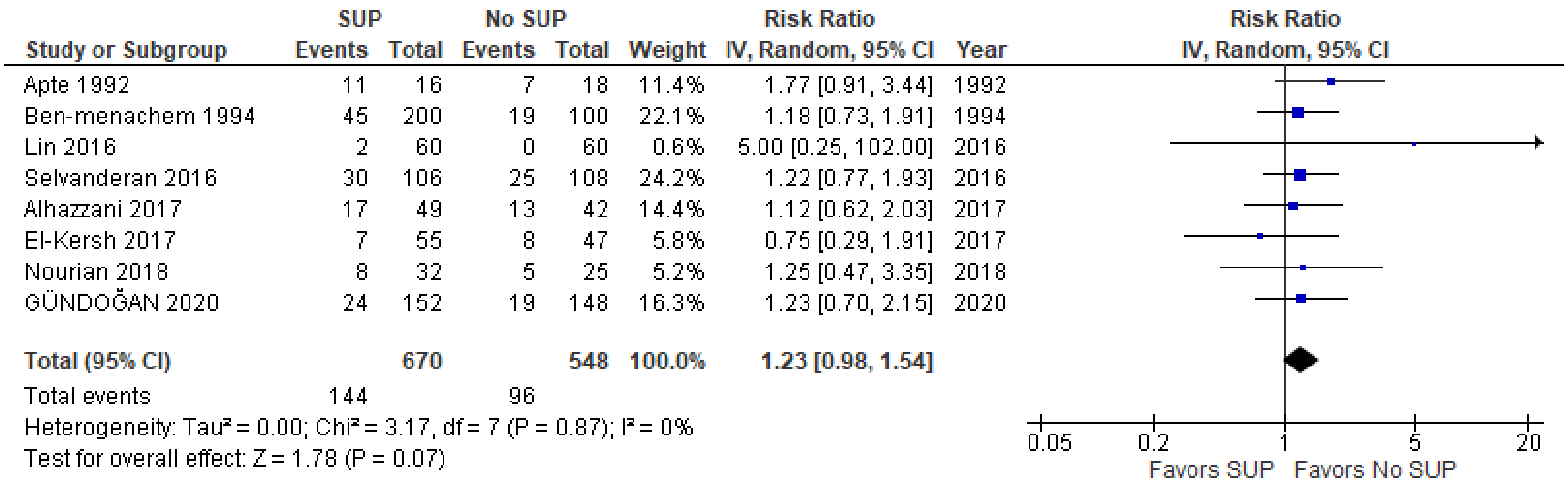

CDI

Study or Subgroup	SUP Events	SUP Total	No SUP Events	No SUP Total	Weight	Risk Ratio IV, Random, 95% CI	Year
Selvanderan 2016	1	106	0	108	20.6%	3.06 [0.13, 74.19]	2016
Alhazzani 2017	2	49	1	42	37.4%	1.71 [0.16, 18.24]	2017
El-Kersh 2017	1	55	3	47	42.1%	0.28 [0.03, 2.65]	2017
Nourian 2018	0	25	0	25		Not estimable	2018
**Total (95% CI)**		**235**		**222**	**100.0%**	**0.91 [0.21, 3.85]**	
Total events	4		4				

Heterogeneity: Tau² = 0.00; Chi² = 1.87, df = 2 (P = 0.39); I² = 0%
Test for overall effect: Z = 0.13 (P = 0.90)

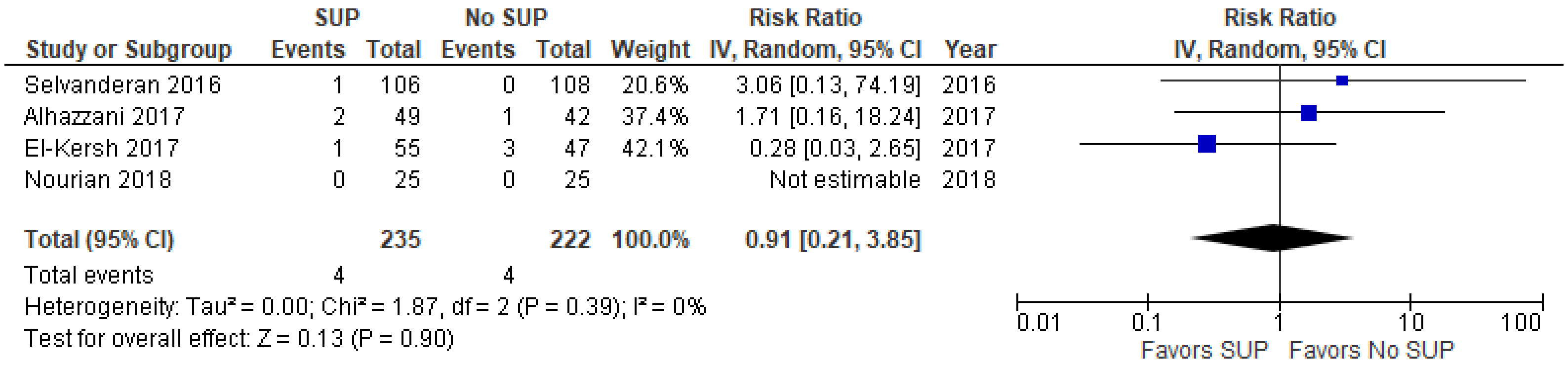

ICU LOS

Study or Subgroup	SUP Mean	SUP SD	SUP Total	No SUP Mean	No SUP SD	No SUP Total	Weight	Mean Difference IV, Random, 95% CI
Alhazzani 2017	12	11.1	49	8.5	8.9	42	7.4%	3.50 [-0.61, 7.61]
Ben-menachem 1994	3.5	4.9	200	3	4.4	100	42.5%	0.50 [-0.60, 1.60]
El-Kersh 2017	6	4.1	55	7	5.9	47	22.9%	-1.00 [-3.00, 1.00]
GÜNDOĞAN 2020	5	25.2	152	3	76.3	148	0.8%	2.00 [-10.93, 14.93]
Selvanderan 2016	6	5.9	106	7	7.4	108	26.4%	-1.00 [-2.79, 0.79]
**Total (95% CI)**			**562**			**445**	**100.0%**	**-0.01 [-1.19, 1.18]**

Heterogeneity: Tau² = 0.54; Chi² = 5.81, df = 4 (P = 0.21); I² = 31%
Test for overall effect: Z = 0.01 (P = 0.99)

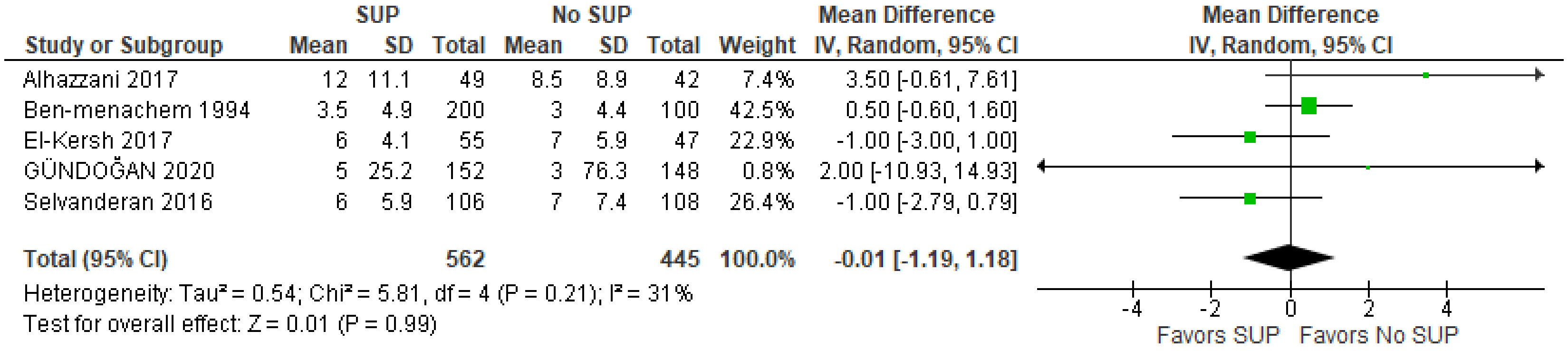

Duration of MV

Study or Subgroup	SUP Mean	SUP SD	SUP Total	No SUP Mean	No SUP SD	No SUP Total	Weight	Mean Difference IV, Random, 95% CI	Year
Ben-menachem 1994	7.7	9.1	200	7.9	9.6	100	22.2%	-0.20 [-2.47, 2.07]	1994
Selvanderan 2016	7	18.5	106	7	15.7	108	5.6%	0.00 [-4.60, 4.60]	2016
Alhazzani 2017	9	8.9	49	6.5	7.4	42	10.4%	2.50 [-0.85, 5.85]	2017
El-Kersh 2017	4	3.6	55	5	3.7	47	52.5%	-1.00 [-2.42, 0.42]	2017
GÜNDOĞAN 2020	4	21.5	152	3	5.9	148	9.3%	1.00 [-2.55, 4.55]	2020
**Total (95% CI)**			**562**			**445**	**100.0%**	**-0.22 [-1.31, 0.88]**	

Heterogeneity: Tau² = 0.07; Chi² = 4.15, df = 4 (P = 0.39); I² = 4%
Test for overall effect: Z = 0.39 (P = 0.70)

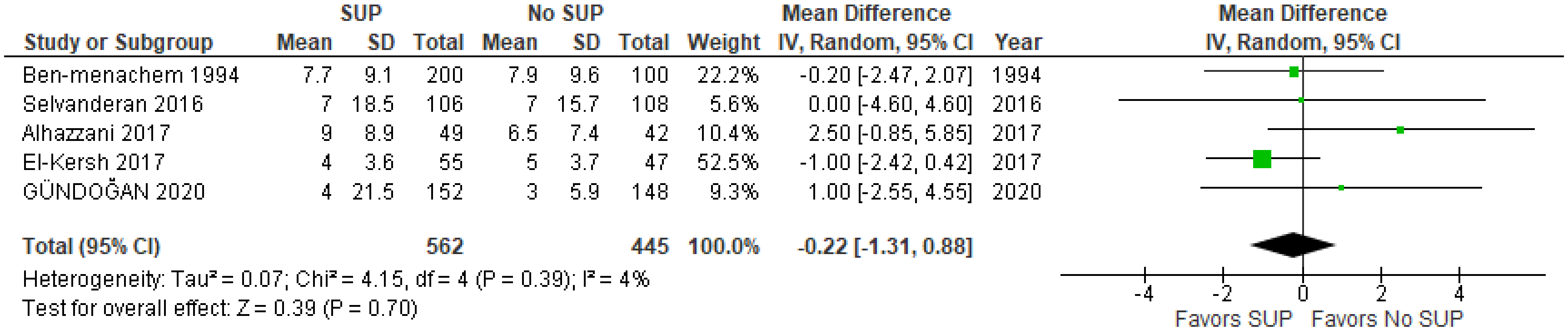

HAP

Study or Subgroup	SUP Events	SUP Total	No SUP Events	No SUP Total	Weight	Risk Ratio IV, Random, 95% CI	Year
Apte 1992	13	16	9	18	44.8%	1.63 [0.97, 2.73]	1992
Ben-menachem 1994	25	200	6	100	16.4%	2.08 [0.88, 4.91]	1994
Lin 2016	4	60	6	60	8.2%	0.67 [0.20, 2.24]	2016
Selvanderan 2016	12	106	8	108	16.5%	1.53 [0.65, 3.59]	2016
Alhazzani 2017	10	49	6	42	14.1%	1.43 [0.57, 3.60]	2017
**Total (95% CI)**		**431**		**328**	**100.0%**	**1.53 [1.08, 2.16]**	
Total events	64		35				

Heterogeneity: Tau² = 0.00; Chi² = 2.37, df = 4 (P = 0.67); I² = 0%
Test for overall effect: Z = 2.40 (P = 0.02)

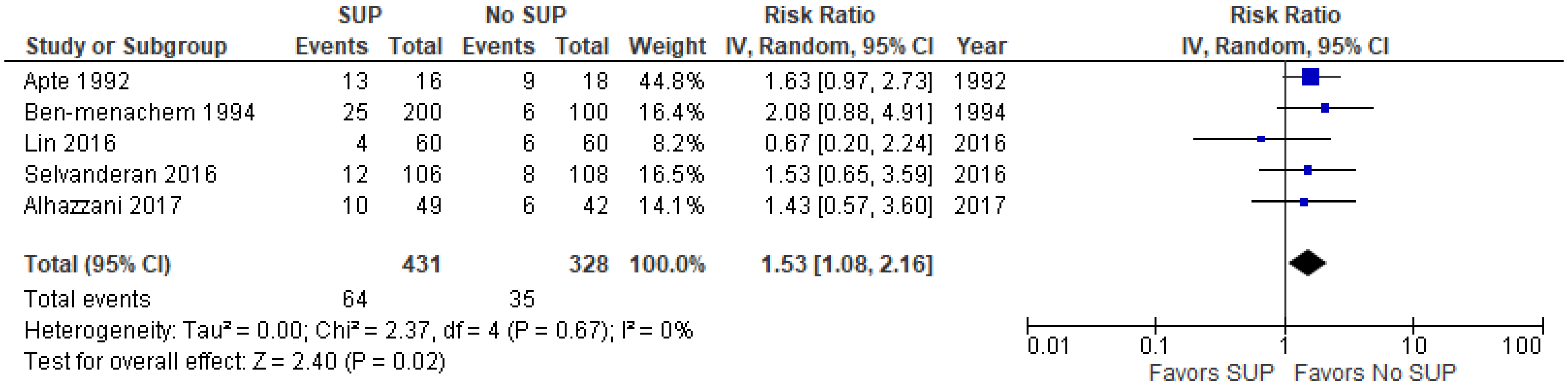

VAP

Study or Subgroup	SUP Events	SUP Total	No SUP Events	No SUP Total	Weight	Risk Ratio IV, Random, 95% CI	Year
Lin 2016	4	60	6	60	14.5%	0.67 [0.20, 2.24]	2016
Selvanderan 2016	12	106	8	108	29.3%	1.53 [0.65, 3.59]	2016
Alhazzani 2017	10	49	6	42	25.0%	1.43 [0.57, 3.60]	2017
Nourian 2018	9	25	7	28	31.2%	1.44 [0.63, 3.29]	2018
**Total (95% CI)**		**240**		**238**	**100.0%**	**1.31 [0.82, 2.08]**	
Total events	35		27				

Heterogeneity: Tau² = 0.00; Chi² = 1.40, df = 3 (P = 0.71); I² = 0%
Test for overall effect: Z = 1.14 (P = 0.25)

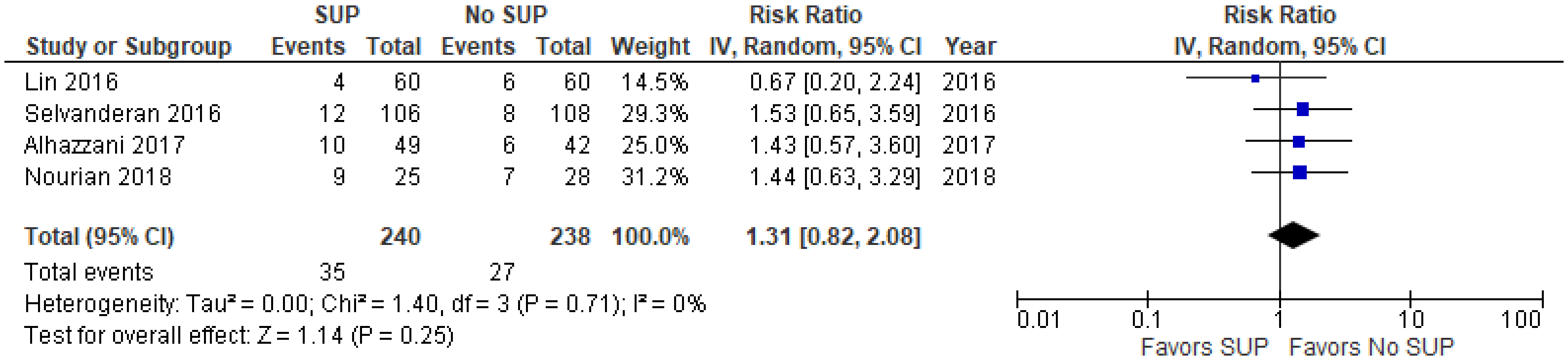

Supplemental Content 2.18 GRADE Evidence Profile PICO 2

Author(s): Dr Fayez Alshamsi

Question: SUP compared to No SUP for Critically ill adults with risk factors for developing stress-related UGIB who are enterally fed during ICU admission

Certainty assessment							№ of patients		Effect		Certainty	Importance
№ of studies	Study design	Risk of bias	Inconsistency	Indirectness	Imprecision	Other considerations	SUP	No SUP	Relative (95% CI)	Absolute (95% CI)		
**CIB**												
8	randomized trials	serious[a]	serious[b]	serious[c]	serious[d]	none	24/652 (3.7%)	17/537 (3.2%)	**RR 1.03** (0.57 to 1.86)	**1 more per 1,000** (from 14 fewer to 27 more)	⨁◯◯◯ Very low[a,b,c,d]	CRITICAL
**Mortality**												
8	randomized trials	serious[a]	not serious	serious[c]	serious[e]	none	144/670 (21.5%)	96/548 (17.5%)	**RR 1.23** (0.98 to 1.54)	**40 more per 1,000** (from 4 fewer to 95 more)	⨁◯◯◯ Very low[a,c,e]	CRITICAL
**CDI**												
4	randomized trials	serious[a]	serious[b]	serious[c]	very serious[f]	none	4/235 (1.7%)	4/222 (1.8%)	**RR 0.91** (0.21 to 3.85)	**2 fewer per 1,000** (from 14 fewer to 51 more)	⨁◯◯◯ Very low[a,b,c,f]	CRITICAL
**VAP**												
4	randomized trials	serious[a]	not serious	serious[c]	very serious[g]	none	35/240 (14.6%)	27/238 (11.3%)	**RR 1.31** (0.82 to 2.08)	**35 more per 1,000** (from 20 fewer to 123 more)	⨁◯◯◯ Very low[a,c,g]	CRITICAL
**HAP**												
5	randomized trials	serious[a,h]	not serious	serious[c]	serious[h]	none	64/431 (14.8%)	35/328 (10.7%)	**RR 1.53** (1.08 to 2.16)	**57 more per 1,000** (from 9 more to 124 more)	⨁◯◯◯ Very low[a,c,h]	CRITICAL
**Overt GIB**												
9	randomized trials	serious[a]	not serious	serious[c]	very serious[i]	none	27/677 (4.0%)	27/562 (4.8%)	**RR 0.88** (0.51 to 1.50)	**6 fewer per 1,000** (from 24 fewer to 24 more)	⨁◯◯◯ Very low[a,c,i]	CRITICAL
**ICU LOS**												
5	randomized trials	serious[a]	serious[j]	serious[c]	serious[e]	none	562	445	-	MD **0.01 lower** (1.19 lower to 1.18 higher)	⨁◯◯◯ Very low[a,c,e,j]	IMPORTANT
**Duration of MV**												
5	randomized trials	serious[a]	serious[k]	serious[c]	serious[e]	none	562	445	-	MD **0.22 lower** (1.31 lower to 0.88 higher)	⨁◯◯◯ Very low[a,c,e,k]	IMPORTANT

CIGIB and Overt GIB- Borthwick et al.: SUP-ICU trial post hoc analyses for associations between enteral nutrition and outcomes

Certainty assessment							№ of patients		Effect		Certainty	Importance
№ of studies	Study design	Risk of bias	Inconsistency	Indirectness	Imprecision	Other considerations	SUP	No SUP	Relative (95% CI)	Absolute (95% CI)		
1	non-randomized studies	serious[l]	not serious	not serious	not serious	none	• n= 3,291 (1 post hoc analysis of RCT). Exposure to EN was statistically significantly associated with a lower HR for both CIGIB and overt GIB (HR: 0.29, 95% CI: 0.19–0.44, p < 0.001 and HR: 0.33, 95% CI: 0.25–0.44, p < 0.001, respectively).<br>• The risk of CIGIB and overt GIB events was also statistically significantly lower in patients randomized to pantoprazole (HR: 0.64, 95% CI: 0.43–0.96, p = 0.028 and HR: 0.58, 95% CI: 0.44–0.76, p < 0.001, respectively). The tests of interaction between exposure to enteral nutrition and SUP treatment allocation demonstrated p = 0.132 and p = 0.848, respectively.				⨁◯◯◯ Very low[l]	CRITICAL
**Pneumonia- Borthwick et al.: SUP-ICU trial post hoc analyses for associations between enteral nutrition and outcomes**												
1	non-randomized studies	serious[l]	not serious	not serious	not serious	none	• n= 3,291 (1 post hoc analysis of RCT). Exposure to enteral nutrition was statistically significantly associated with a greater cause-specific HR for pneumonia (HR: 1.44, 95% CI: 1.14–1.82, p = 0.003)<br>• The risk of pneumonia was also in patients randomized to pantoprazole (HR: 1, 95% CI: 0.84–1.19, p = 0.993). The tests of interaction between exposure to enteral nutrition and SUP treatment allocation demonstrated p = 0.193.				⨁◯◯◯ Very low[l]	CRITICAL
**All-cause mortality- Borthwick et al.: SUP-ICU trial post hoc analyses for associations between enteral nutrition and outcomes**												
1	non-randomized studies	serious[l]	not serious	not serious	not serious	none	• n= 3,291 (1 post hoc analysis of RCT).The HRs for all-cause mortality in patients exposed to EN was statistically significantly reduced (HR: 0.22, 95% CI: 0.18–0.27, p < 0.001 and HR: 0.35, 95% CI: 0.28–0.43, p < 0.001),<br>• No statistically significant difference in patients randomized to SUP with pantoprazole (HR: 1.05, 95% CI: 0.87–1.26, p = 0.631).<br>• Exposure to pantoprazole with enteral nutrition was mostly compatible with an increase in all-cause mortality, although this was not statistically significant (HR: 1.27, 95% CI: 0.99–1.64, p = 0.061).<br>• There was a statistically significant interaction for all-cause mortality between pantoprazole and enteral nutrition (p = 0.024).				⨁◯◯◯ Very low[l]	CRITICAL

CI: confidence interval; **MD:** mean difference; **RR:** risk ratio

Explanations

a. The systematic review by Reynolds had a low risk of bias; however, the RCTs included in the meta-analysis had low-high risk of bias. Nourian & Gundogan were open label; however, randomization was robust, and allocation concealment was likely adequate. Nevertheless, outcomes were hard; therefore, at best, there was an unclear risk of bias. We maintained the same risk of bias.
b. While the I2 was zero, visual inspection of the forest plot showed point estimates on both sides of no difference, despite significant overlap.
c. Downgraded for indirectness due to critical methodological limitations identified in the subgroup analysis by enteral nutrition status. It was noted that definitions of enteral feeding status varied across the included RCTs and were often assessed post-randomization, undermining the credibility of subgroup findings. Concerns were raised that enteral nutrition status was frequently not defined at baseline.
d. We downgraded for imprecision given the very low overall number of events (n=41) and hat confidence interval included significant benefits and harms.
e. We downgraded for imprecision as confidence interval included significant benefits and harms.
f. We downgraded for imprecision by 2 points given the very low overall number of events (n=8) and that very wide confidence interval included significant benefits and harms.
g. We downgraded for imprecision by 2 points given the very low overall number of events (n=62) and that very wide confidence intervals included significant benefits and harms.
h. We downgraded for imprecision by 1 point as overall events number is small (n=99).
i. We downgraded for imprecision by 2 points given the very low overall number of events (n=54) and hat very wide confidence interval included significant benefits and harms.
j. While the I2 was 33%, visual inspection of the forest plot showed point estimates on both sides of no difference, despite significant overlap.
k.While the I2 was 4%, visual inspection of the forest plot showed point estimates on both sides of no difference, despite significant overlap.
l. Subgroup analyses were conducted post hoc, increasing the risk of bias and confounding.

Supplemental Content 2.19 PICO 2: Evidence-to-Decision (EtD) framework

**Should SUP vs. No SUP be used for Critically ill adults with risk factors for developing stress-related UGIB who are enterally fed during ICU admission?**	
**POPULATION:**	Critically ill adults with risk factors for developing stress-related UGIB who are enterally fed during ICU admission
**INTERVENTION:**	SUP
**COMPARISON:**	No SUP
**MAIN OUTCOMES:**	CIB; Mortality; CDI; VAP; HAP; Overt GIB; ICU LOS; Duration of MV; CIGIB and Overt GIB- Borthwick et al.: SUP-ICU trial post hoc analyses for associations between enteral nutrition and outcomes; Pneumonia- Borthwick et al: SUP-ICU trial post hoc analyses for associations between enteral nutrition and outcomes; All-cause mortality- Borthwick et al: SUP-ICU trial post hoc analyses for associations between enteral nutrition and outcomes

ASSESSMENT

Problem
Is the problem a priority?

JUDGEMENT	RESEARCH EVIDENCE	ADDITIONAL CONSIDERATIONS
○ No<br>○ Probably no<br>○ Probably yes<br>● Yes<br>○ Varies<br>○ Don't know	Two recent systematic reviews were identified, one by Reynolds and MacLaren 2019 and another by Haung et al. 2018 that explored SUP versus EN as a subgroup or as the focus of the main systematic review.<br>Another two RCTs (Krag 2018; Nourian 2018) were published and not included in the previous RCTs. Haung et al. included studies where patients were receiving EN >60% of their target. Reynolds 2019 performed subgroups analyses of studies that described EN use.<br><br>In this analysis, we added data from the Nourian 2018 and Gundogan 2020 RCTs	

Desirable Effects

How substantial are the desirable anticipated effects?

JUDGEMENT

- ○ Trivial
- ● Small
- ○ Moderate
- ○ Large
- ○ Varies
- ○ Don't know

RESEARCH EVIDENCE

Outcomes	№ of participants (studies) Follow-up	Certainty of the evidence (GRADE)	Relative effect (95% CI)	Anticipated absolute effects* (95% CI)	
				Risk with No SUP	Risk difference with SUP
CIB	1189 (8 RCTs)	⊕◯◯◯ Very low[a,b,c,d]	**RR 1.03** (0.57 to 1.86)	Study population	
				32 per 1,000	**1 more per 1,000** (14 fewer to 27 more)
Mortality	1218 (8 RCTs)	⊕◯◯◯ Very low[a,c,e]	**RR 1.23** (0.98 to 1.54)	Study population	
				175 per 1,000	**40 more per 1,000** (4 fewer to 95 more)
CDI	457 (4 RCTs)	⊕◯◯◯ Very low[a,b,c,f]	**RR 0.91** (0.21 to 3.85)	Study population	
				18 per 1,000	**2 fewer per 1,000** (14 fewer to 51 more)
VAP	478 (4 RCTs)	⊕◯◯◯ Very low[a,c,g]	**RR 1.31** (0.82 to 2.08)	Study population	
				113 per 1,000	**35 more per 1,000** (20 fewer to 123 more)
HAP	759 (5 RCTs)	⊕◯◯◯ Very low[a,c,h]	**RR 1.53** (1.08 to 2.16)	Study population	
				107 per 1,000	**57 more per 1,000** (9 more to 124 more)
Overt GIB	1239 (9 RCTs)	⊕◯◯◯ Very low[a,c,i]	**RR 0.88** (0.51 to 1.50)	Study population	
				48 per 1,000	**6 fewer per 1,000** (24 fewer to 24 more)
ICU LOS	1007 (5 RCTs)	⊕◯◯◯ Very low[a,c,e,j]	-	The mean ICU LOS was **0**	MD **0.01 lower** (1.19 lower to 1.18 higher)

ADDITIONAL CONSIDERATIONS

With SUP decreased in CIB
Average rate of clinically important bleeding 3.5%

Meta-analysis by Haung et al. included RCTs with at least 50% receiving EN (not clear if before enrollment or after). Post randomization variable in some studies.
Concern about applicability of data if not 100% at baseline.

Downgrade for indirectness:
The panel agreed to downgrade for indirectness due to critical methodological limitations identified in the subgroup analysis by enteral nutrition status. It was noted that definitions of enteral feeding status varied across the included RCTs and were often assessed post-randomization, undermining the credibility of subgroup findings. Concerns were raised that enteral nutrition status was frequently not defined at baseline, and in some trials, subgroup analyses were conducted post hoc, increasing the risk of bias and confounding. There was consensus that these issues significantly reduce the applicability of the evidence to the clinical question, justifying a downgrade for indirectness and reinforcing the need for cautious interpretation of the results.

Duration of MV	1007<br>(5 RCTs)	⨁◯◯◯<br>Very low[a,c,e,k]	-	The mean duration of MV was **0**	MD **0.22 lower**<br>(1.31 lower to 0.88 higher)
CIGIB and Overt GIB- Borthwick et al.: SUP-ICU trial post hoc analyses for associations between enteral nutrition and outcomes	0<br>(1 non-randomized study)	⨁◯◯◯<br>Very low[l]	-	• n= 3,291 (1 post hoc analysis of RCT). Exposure to EN was statistically significantly associated with a lower HR for both CIGIB and overt GIB (HR: 0.29, 95% CI: 0.19–0.44, p < 0.001 and HR: 0.33, 95% CI: 0.25–0.44, p < 0.001, respectively).<br>• The risk of CIGIB and overt GIB events was also statistically significantly lower in patients randomized to pantoprazole (HR: 0.64, 95% CI: 0.43–0.96, p = 0.028 and HR: 0.58, 95% CI: 0.44–0.76, p < 0.001, respectively). The tests of interaction between exposure to enteral nutrition and SUP treatment allocation (p = 0.132; p = 0.848, respectively).	
Pneumonia- Borthwick et al.: SUP-ICU trial post hoc analyses for associations between enteral nutrition and outcomes	0<br>(1 non-randomized study)	⨁◯◯◯<br>Very low[l]	-	• n= 3,291 (1 post hoc analysis of RCT) exposure to enteral nutrition was statistically significantly associated with a greater cause-specific HR for pneumonia (HR: 1.44, 95% CI: 1.14–1.82, p = 0.003)<br>• The risk of pneumonia was also in patients randomized to pantoprazole (HR: 1, 95% CI: 0.84–1.19, p = 0.993). The tests of interaction between exposure to enteral nutrition and SUP treatment allocation (p = 0.193).	
All-cause mortality- Borthwick et al.: SUP-ICU trial post hoc analyses for associations between enteral nutrition and outcomes	0<br>(1 non-randomized study)	⨁◯◯◯<br>Very low[l]	-	• n= 3,291 (1 post hoc analysis of RCT). The HRs for all-cause mortality in patients exposed to EN was statistically significantly reduced (HR: 0.22, 95% CI: 0.18–0.27, p < 0.001 and HR: 0.35, 95% CI: 0.28–0.43, p < 0.001).<br>• No statistically significant difference in patients randomized to SUP with pantoprazole (HR: 1.05, 95% CI: 0.87–1.26, p = 0.631).<br>• Exposure to pantoprazole with enteral nutrition was mostly compatible with an increase in all-cause mortality, although this was not statistically significant (HR: 1.27, 95% CI: 0.99–1.64, p = 0.061).<br>• There was a statistically significant interaction for all-cause mortality between pantoprazole and enteral nutrition (p = 0.024).	

a. The systematic review by Reynolds had a low risk of bias; however, the RCTs included in the meta-analysis had low-high risk of bias. Nourian & Gundogan were open label; however,

One panel member voted for varies: 48 per 1000 versus 42 per 1000 overt GI bleeding. This is trivial to me.

	randomization was robust, and allocation concealment was likely adequate. Nevertheless, outcomes were hard; therefore, at best, unclear risk of bias. We maintained the same risk of bias.<br>b. While the I2 was zero, visual inspection of the forest plot showed point estimates on both sides of no difference, despite significant overlap.<br>c. Downgraded for indirectness due to critical methodological limitations identified in the subgroup analysis by enteral nutrition status. It was noted that definitions of enteral feeding status varied across the included RCTs and were often assessed post-randomization, undermining the credibility of subgroup findings. Concerns were raised that enteral nutrition status was frequently not defined at baseline.<br>d. We downgraded for imprecision given the very low overall number of events (n=41) and that confidence intervals included significant benefits and harms.<br>e. We downgraded for imprecision as confidence intervals included significant benefits and harms.<br>f. We downgraded for imprecision by 2 points given the very low overall number of events (n=8) and that very wide confidence intervals included significant benefits and harms.<br>g. We downgraded for imprecision by 2 points given the very low overall number of events (n=62) and that very wide confidence intervals included significant benefits and harms.<br>h. We downgraded for imprecision by 1 point as overall events number is small (n=99).<br>i. We downgraded for imprecision by 2 points given the very low overall number of events (n=54) and that very wide confidence intervals included significant benefits and harms.<br>j. While the I2 was 33%, visual inspection of the forest plot showed point estimates on both sides of no difference, despite significant overlap.<br>k. While the I2 was 4%, visual inspection of the forest plot showed point estimates on both sides of no difference, despite significant overlap.<br>l. Subgroup analyses were conducted post hoc, increasing the risk of bias and confounding.	

Undesirable Effects

How substantial are the undesirable anticipated effects?

JUDGEMENT	RESEARCH EVIDENCE	ADDITIONAL CONSIDERATIONS
○ Trivial<br>○ Small<br>○ Moderate<br>○ Large<br>● Varies<br>○ Don't know	(see table below)	Increased rate of VAP and HAP with SUP<br><br>**Panel comment:** There are more undesirable effects with SUP and seems to be consistent.

Outcomes	№ of participants (studies) Follow-up	Certainty of the evidence (GRADE)	Relative effect (95% CI)	Anticipated absolute effects* (95% CI)	
				Risk with No SUP	Risk difference with SUP
CIB	1189 (8 RCTs)	⨁◯◯◯ Very low[a,b,c,d]	**RR 1.03** (0.57 to 1.86)	Study population	
				32 per 1,000	**1 more per 1,000** (14 fewer to 27 more)
Mortality	1218 (8 RCTs)	⨁◯◯◯ Very low[a,c,e]	**RR 1.23** (0.98 to 1.54)	Study population	
				175 per 1,000	**40 more per 1,000** (4 fewer to 95 more)
CDI	457 (4 RCTs)	⨁◯◯◯ Very low[a,b,c,f]	**RR 0.91** (0.21 to 3.85)	Study population	
				18 per 1,000	**2 fewer per 1,000** (14 fewer to 51 more)
VAP	478 (4 RCTs)	⨁◯◯◯ Very low[a,c,g]	**RR 1.31** (0.82 to 2.08)	Study population	
				113 per 1,000	**35 more per 1,000** (20 fewer to 123 more)
HAP	759 (5 RCTs)	⨁◯◯◯ Very low[a,c,h]	**RR 1.53** (1.08 to 2.16)	Study population	
				107 per 1,000	**57 more per 1,000** (9 more to 124 more)
Overt GIB	1239 (9 RCTs)	⨁◯◯◯ Very low[a,c,i]	**RR 0.88** (0.51 to 1.50)	Study population	
				48 per 1,000	**6 fewer per 1,000** (24 fewer to 24 more)
ICU LOS	1007 (5 RCTs)	⨁◯◯◯ Very low[a,c,e,j]	-	The mean ICU LOS was **0**	MD **0.01 lower** (1.19 lower to 1.18 higher)

Duration of MV	1007<br>(5 RCTs)	⊕◯◯◯<br>Very low[a,c,e,k]	-	The mean duration of MV was **0**	MD **0.22 lower**<br>(1.31 lower to 0.88 higher)
CIGIB and Overt GIB- Borthwick et al.: SUP-ICU trial post hoc analyses for associations between enteral nutrition and outcomes	0<br>(1 non-randomized study)	⊕◯◯◯<br>Very low[l]	-	• n= 3,291 (1 post hoc analysis of RCT) Exposure to EN was statistically significantly associated with a lower HR for both CIGIB and overt GIB (HR: 0.29, 95% CI: 0.19–0.44, p < 0.001 and HR: 0.33, 95% CI: 0.25–0.44, p < 0.001, respectively).<br>• The risk of CIGIB and overt GIB events was also statistically significantly lower in patients randomized to pantoprazole (HR: 0.64, 95% CI: 0.43–0.96, p = 0.028 and HR: 0.58, 95% CI: 0.44–0.76, p < 0.001, respectively). The tests of interaction between exposure to enteral nutrition and SUP treatment allocation demonstrated p = 0.132 and p = 0.848, respectively.	
Pneumonia- Borthwick et al.: SUP-ICU trial post hoc analyses for associations between enteral nutrition and outcomes	0<br>(1 non-randomized study)	⊕◯◯◯<br>Very low[l]	-	• n= 3,291 (1 post hoc analysis of RCT) Exposure to enteral nutrition was statistically significantly associated with a greater cause-specific HR for pneumonia (HR: 1.44, 95% CI: 1.14–1.82, p = 0.003)<br>• The risk of pneumonia was also in patients randomized to pantoprazole (HR: 1, 95% CI: 0.84–1.19, p = 0.993). The tests of interaction between exposure to enteral nutrition and SUP treatment allocation demonstrated p = 0.193.	
All-cause mortality- Borthwick et al.: SUP-ICU trial post hoc analyses for associations between enteral nutrition and outcomes	0<br>(1 non-randomized study)	⊕◯◯◯<br>Very low[l]	-	• n= 3,291 (1 post hoc analysis of RCT). The HRs for all-cause mortality in patients exposed to EN was statistically significantly reduced (HR: 0.22, 95% CI: 0.18–0.27, p < 0.001 and HR: 0.35, 95% CI: 0.28–0.43, p < 0.001).<br>• No statistically significant difference in patients randomized to SUP with pantoprazole was observed (HR: 1.05, 95% CI: 0.87–1.26, p = 0.631).<br>• Exposure to pantoprazole with enteral nutrition was mostly compatible with an increase in all-cause mortality, although this was not statistically significant (HR: 1.27, 95% CI: 0.99–1.64, p = 0.061).<br>• There was a statistically significant interaction for all-cause mortality between pantoprazole and enteral nutrition (p = 0.024).	

	a. The systematic review by Reynolds had a low risk of bias; however, the RCTs included in the meta-analysis had low-high risk of bias. Nourian & Gundogan were open label; however, randomization was robust, and allocation concealment was likely adequate. Nevertheless, outcomes were hard; therefore, at best, there was an unclear risk of bias. We maintained the same risk of bias.<br>b. While the I2 was zero, visual inspection of the forest plot showed point estimates on both sides of no difference, despite significant overlap.<br>c. Downgraded for indirectness due to critical methodological limitations identified in the subgroup analysis by enteral nutrition status. It was noted that definitions of enteral feeding status varied across the included RCTs and were often assessed post-randomization, undermining the credibility of subgroup findings. Concerns were raised that enteral nutrition status was frequently not defined at baseline.<br>d. We downgraded for imprecision given the very low overall number of events (n=41) and that confidence intervals included significant benefits and harms.<br>e. We downgraded for imprecision as confidence intervals included significant benefits and harms.<br>f. We downgraded for imprecision by 2 points given the very low overall number of events (n=8) and that very wide confidence intervals included significant benefits and harms.<br>g. We downgraded for imprecision by 2 points given the very low overall number of events (n=62) and that very wide confidence intervals included significant benefits and harms.<br>h. We downgraded for imprecision by 1 point as overall events number is small (n=99).<br>i. We downgraded for imprecision by 2 points given the very low overall number of events (n=54) and that very wide confidence intervals included significant benefits and harms.<br>j. While the I2 was 33%, visual inspection of the forest plot showed point estimates on both sides of no difference, despite significant overlap.<br>k. While the I2 was 4%, visual inspection of the forest plot showed point estimates on both sides of no difference, despite significant overlap.<br>l. Subgroup analyses were conducted post hoc, increasing the risk of bias and confounding.	

Certainty of evidence

What is the overall certainty of the evidence of effects?

JUDGEMENT

- ● Very low
- ○ Low
- ○ Moderate
- ○ High
- ○ No included studies

RESEARCH EVIDENCE

Outcomes	Anticipated absolute effects* (95% CI)		Relative effect (95% CI)	№ of participants (studies)	Certainty of the evidence (GRADE)	Comments
	Risk with No SUP	Risk with SUP				
CIB	Study population		**RR 1.03** (0.57 to 1.86)	1189 (8 RCTs)	⨁◯◯◯ Very low[a,b,c,d]	
	32 per 1,000	**33 per 1,000** (18 to 59)				
Mortality	Study population		**RR 1.23** (0.98 to 1.54)	1218 (8 RCTs)	⨁◯◯◯ Very low[a,c,e]	
	175 per 1,000	**215 per 1,000** (172 to 270)				
CDI	Study population		**RR 0.91** (0.21 to 3.85)	457 (4 RCTs)	⨁◯◯◯ Very low[a,b,c,f]	
	18 per 1,000	**16 per 1,000** (4 to 69)				
VAP	Study population		**RR 1.31** (0.82 to 2.08)	478 (4 RCTs)	⨁◯◯◯ Very low[a,c,g]	
	113 per 1,000	**149 per 1,000** (93 to 236)				
HAP	Study population		**RR 1.53** (1.08 to 2.16)	759 (5 RCTs)	⨁◯◯◯ Very low[a,c,h]	
	107 per 1,000	**163 per 1,000** (115 to 230)				
Overt GIB	Study population		**RR 0.88** (0.51 to 1.50)	1239 (9 RCTs)	⨁◯◯◯ Very low[a,c,i]	
	48 per 1,000	**42 per 1,000** (25 to 72)				
ICU LOS	The mean ICU LOS was **0**	MD **0.01 lower** (1.19 lower to 1.18 higher)	-	1007 (5 RCTs)	⨁◯◯◯ Very low[a,c,e,j]	

ADDITIONAL CONSIDERATIONS

Duration of MV	The mean duration of MV was **0**	MD **0.22 lower** (1.31 lower to 0.88 higher)	-	1007 (5 RCTs)	⊕◯◯◯ Very low[a,c,e,k]	

a. The systematic review by Reynolds had a low risk of bias; however, the RCTs included in the meta-analysis had low-high risk of bias. Nourian & Gundogan were open label; however, randomization was robust, and allocation concealment was likely adequate. Nevertheless, outcomes were hard; therefore, at best, there was an unclear risk of bias. We maintained the same risk of bias.
b. While the I2 was zero, visual inspection of the forest plot showed point estimates on both sides of no difference, despite significant overlap.
c. Downgraded for indirectness due to critical methodological limitations identified in the subgroup analysis by enteral nutrition status. It was noted that definitions of enteral feeding status varied across the included RCTs and were often assessed post-randomization, undermining the credibility of subgroup findings. Concerns were raised that enteral nutrition status was frequently not defined at baseline.
d. We downgraded for imprecision given the very low overall number of events (n=41) and that confidence intervals included significant benefits and harms.
e. We downgraded for imprecision as confidence intervals included significant benefits and harms.
f. We downgraded for imprecision by 2 points given the very low overall number of events (n=8) and that very wide confidence intervals included significant benefits and harms.
g. We downgraded for imprecision by 2 points given the very low overall number of events (n=62) and that very wide confidence intervals included significant benefits and harms.
h. We downgraded for imprecision by 1 point as overall events number is small (n=99).
i. We downgraded for imprecision by 2 points given the very low overall number of events (n=54) and that very wide confidence intervals included significant benefits and harms.
j. While the I2 was 33%, visual inspection of the forest plot showed point estimates on both sides of no difference, despite significant overlap.
k. While the I2 was 4%, visual inspection of the forest plot showed point estimates on both sides of no difference, despite significant overlap.

Values

Is there important uncertainty about or variability in how much people value the main outcomes?

JUDGEMENT	RESEARCH EVIDENCE	ADDITIONAL CONSIDERATIONS
○ Important uncertainty or variability<br>● Possibly important uncertainty or variability<br>○ Probably no important uncertainty or variability<br>○ No important uncertainty or variability	No value and preference studies available to inform.<br><br>There is no direct evidence on patient or surrogate preferences regarding the outcomes of interest. Panel members highlighted that in critically ill patients, especially those unable to communicate, assumptions must be made about what outcomes patients would value. It was noted that some patients or families might prioritize avoiding complications such as pneumonia over the prevention of upper gastrointestinal bleeding. Given these considerations, the panel concluded that there is possibly important uncertainty or variability in how patients value these outcomes.	

Balance of effects

Does the balance between desirable and undesirable effects favor the intervention or the comparison?

JUDGEMENT	RESEARCH EVIDENCE	ADDITIONAL CONSIDERATIONS
○ Favors the comparison<br>○ Probably favors the comparison<br>○ Does not favor either the intervention or the comparison<br>○ Probably favors the intervention<br>○ Favors the intervention<br>● Varies<br>○ Don't know	Discussion among the panel<br><br>The patient's baseline risk of bleeding influences the balance of effects. In low-risk patients, clinicians may wish to avoid exposing them to the potential increased risk of HAP or VAP. Conversely, in patients at high risk of bleeding, the elevated bleeding risk associated with enteral nutrition alone may lead prescribers to accept a higher risk of HAP/VAP to reduce the likelihood of clinically important bleeding.	The panel reviewed relative risk estimates and found no strong or consistent benefit of SUP in enterally fed critically ill patients. The reduction in gastrointestinal bleeding appeared small and statistically non-significant, while there was concern that SUP might increase the risk of pneumonia. Methodological limitations were noted, particularly the fact that enteral feeding was initiated post-randomization in many studies, introducing potential bias. Additionally, the panel viewed findings from post hoc subgroup analyses as unreliable. Based on these considerations, the panel concluded that the balance of effects varies.

Resources required

JUDGEMENT	RESEARCH EVIDENCE	ADDITIONAL CONSIDERATIONS
○ Large costs<br>○ Moderate costs<br>● Negligible costs and savings<br>○ Moderate savings<br>○ Large savings<br>○ Varies<br>○ Don't know	No cost effectiveness studies available.	**Panel discussion**<br>SUP agents, such as proton pump inhibitors, are generally considered low-cost interventions. However, multiple panel members emphasized that cost-effectiveness encompasses more than the acquisition price of the medication. It also includes the cost of managing potential adverse effects, preparation, administration, and broader healthcare system impacts. Given the absence of formal cost-effectiveness studies, several panelists noted the difficulty in assessing the balance between the cost of complications and the potential benefits of preventing stress-related gastrointestinal bleeding. The panel acknowledged that the cost-benefit relationship may vary depending on the clinical outcome of interest.<br>**Final Judgment:**<br>• Resource use: Negligible 81% agreement 14% voted for "Don't know."<br>• Cost-effectiveness is uncertain due to lack of studies and complexity of factors involved.

Certainty of evidence of required resources

What is the certainty of the evidence of resource requirements (costs)?

JUDGEMENT	RESEARCH EVIDENCE	ADDITIONAL CONSIDERATIONS
o Very low<br>o Low<br>o Moderate<br>o High<br>● No included studies		

Cost effectiveness

Does the cost-effectiveness of the intervention favor the intervention or the comparison?

JUDGEMENT	RESEARCH EVIDENCE	ADDITIONAL CONSIDERATIONS
o Favors the comparison<br>o Probably favors the comparison<br>o Does not favor either the intervention or the comparison<br>o Probably favors the intervention<br>o Favors the intervention<br>o Varies<br>● No included studies		

Equity

What would be the impact on health equity?

JUDGEMENT	RESEARCH EVIDENCE	ADDITIONAL CONSIDERATIONS
○ Reduced<br>○ Probably reduced<br>● Probably no impact<br>○ Probably increased<br>○ Increased<br>○ Varies<br>○ Don't know		

Acceptability

Is the intervention acceptable to key stakeholders?

JUDGEMENT	RESEARCH EVIDENCE	ADDITIONAL CONSIDERATIONS
○ No<br>○ Probably no<br>● Probably yes<br>○ Yes<br>○ Varies<br>○ Don't know	Acceptable to use EN or SUP	

Feasibility

Is the intervention feasible to implement?

JUDGEMENT	RESEARCH EVIDENCE	ADDITIONAL CONSIDERATIONS
○ No<br>○ Probably no<br>○ Probably yes<br>● Yes<br>○ Varies<br>○ Don't know		**Panel comments**<br>**Panel Discussion Highlight:** The panel raised concerns about the frequent interruptions of enteral nutrition (EN) in real-life ICU settings due to clinical procedures, intolerance, or other factors. This may limit the practical feasibility of relying solely on EN to provide protection against stress-related gastrointestinal bleeding.

		**Final Judgment:** While the intervention (enteral nutrition with or without SUP) is technically feasible, real-world practice variability—particularly interruptions in EN delivery—should be taken into account when interpreting the applicability of the recommendation.

SUMMARY OF JUDGEMENTS

	JUDGEMENT						
PROBLEM	No	Probably no	Probably yes	**Yes**		Varies	Don't know
DESIRABLE EFFECTS	Trivial	**Small**	Moderate	Large		Varies	Don't know
UNDESIRABLE EFFECTS	Trivial	Small	Moderate	Large		**Varies**	Don't know
CERTAINTY OF EVIDENCE	**Very low**	Low	Moderate	High			No included studies
VALUES	Important uncertainty or variability	**Possibly important uncertainty or variability**	Probably no important uncertainty or variability	No important uncertainty or variability			
BALANCE OF EFFECTS	Favors the comparison	Probably favors the comparison	Does not favor either the intervention or the comparison	Probably favors the intervention	Favors the intervention	**Varies**	Don't know
RESOURCES REQUIRED	Large costs	Moderate costs	**Negligible costs and savings**	Moderate savings	Large savings	Varies	Don't know
CERTAINTY OF EVIDENCE OF REQUIRED RESOURCES	Very low	Low	Moderate	High			**No included studies**
COST EFFECTIVENESS	Favors the comparison	Probably favors the comparison	Does not favor either the	Probably favors the intervention	Favors the intervention	Varies	**No included studies**

	JUDGEMENT						
			intervention or the comparison				
EQUITY	Reduced	Probably reduced	**Probably no impact**	Probably increased	Increased	Varies	Don't know
ACCEPTABILITY	No	Probably no	**Probably yes**	Yes		Varies	Don't know
FEASIBILITY	No	Probably no	Probably yes	**Yes**		Varies	Don't know

TYPE OF RECOMMENDATION

Strong recommendation against the intervention	Conditional recommendation against the intervention	Conditional recommendation for either the intervention or the comparison	**Conditional recommendation for the intervention**	Strong recommendation for the intervention
○	○	○	●	○

CONCLUSIONS

Recommendation

We suggest using pharmacologic SUP for critically ill adults who are enterally fed and possess one or more risk factor(s) for clinically important stress-related UGIB compared to no SUP (Conditional recommendation, very low certainty of evidence).
We suggest not using pharmacologic SUP for critically ill adults who are enterally fed and at low risk for clinically important stress-related UGIB (Conditional recommendation, very low certainty of evidence).

Remark: The phrase "low or no risk factors" is used to reflect a category of patients at minimal baseline risk. For the definition of high-risk patients, please refer to **PICO 1**, which includes criteria such as coagulopathy, shock, and chronic liver disease.

Justification

The use of SUP in patients receiving enteral nutrition (EN) is a priority problem. Consideration must be made in whether a patient is at low risk or high risk for clinically important bleeding (CIB). SUP is associated with decreased risk of bleeding but carries an increased risk of HAP and VAP. In contrast, EN demonstrated no difference in mortality, overt GIB, or CDI and is associated with a lower risk of HAP and VAP compared to SUP. Outcome values vary, highlighting the need to consider each patient's individual risk of CIB. For example, a patient's baseline risk of bleeding effects the balance of effects. In patients at low risk of bleeding, clinicians may wish to avoid exposing them to the potential increased risk of HAP and VAP. Conversely, in patients at high risk of bleeding, the increased risk of bleeding with EN alone may lead prescribers to accept a higher risk of HAP/VAP to reduce the likelihood of clinically important bleeding.

Subgroup considerations

Bleeding risk is a potential factor that may influence clinical decision-making.

Implementation considerations

Consideration of local and patient VAP/HAP risks and rates. Consideration of local sensitivities of multidrug resistant organisms. Education is needed on how to implement these recommendations at an institutional level.

Monitoring and evaluation

Institutions should monitor their HAP and VAP rates with stress ulcer prophylaxis.

Research priorities

Future randomized controlled trials should examine the route, amount, timing and composition of enteral nutrition in preventing clinically important of bleeding. Studies should also examine the amount (trophic versus full feeding) of feeding to a protective benefit. Physiology studies should examine the mechanism of protective benefit or harm. RCTs should also examine the difference in H2RA versus PPI versus EN with no prophylaxis on clinically important bleeding. There is a need for conjoint analyses of patient and provider preferences as well as cost effectiveness analyses. The question of SUP versus enteral nutrition alone is not definitively answered, and higher certainty of evidence is needed.

Supplemental Content 2.20 AMSTAR-2 Assessment of Systematic Reviews for PICO 3

Question		Ying Wang 2020 (1)	Ying Wang 2020, updated SRMA (3)
1.	Did the research questions and inclusion criteria for the review include the components of PICO?	Yes	Yes
2.	Did the report of the review contain an explicit statement that the review methods were established prior to the conduct of the review and did the report justify any significant deviations from the protocol?	Yes PROSPERO (CRD42019126656)	Yes PROSPERO (CRD42020169989)
3.	Did the review authors explain their selection of the study designs for inclusion in the review?	Yes	Yes
4.	Did the review authors use a comprehensive literature search strategy?	Yes	Yes
5.	Did the review authors perform study selection in duplicate?	Yes	Yes
6.	Did the review authors perform data extraction in duplicate?	Yes	Yes
7.	Did the review authors provide a list of excluded studies and justify the exclusions?	No	No
8.	Did the review authors describe the included studies in adequate detail?	Yes	Yes
9.	Did the review authors use a satisfactory technique for assessing the RoB in individual studies that were included in the review?	Yes	Yes
10.	Did the review authors report on the sources of funding for the studies included in the review?	No	No
11.	If meta-analysis was performed, did the review authors use appropriate methods for statistical combination of results?	Yes	Yes
12.	If meta-analysis was performed, did the review authors assess the potential impact of RoB in individual studies on the results of the meta-analysis or other evidence synthesis?	Yes	Yes
13.	Did the review authors account for RoB in individual studies when interpreting/discussing the results of the review?	Yes	Yes
14.	Did the review authors provide a satisfactory explanation for, and discussion of, any heterogeneity observed in the results of the review?	Yes	Yes

15.	If quantitative synthesis was performed, did the review authors carry out an adequate investigation of publication bias (small study bias) and discuss its impact on the results of the review?	Yes	Yes
16.	Did the review authors report any potential sources of conflict of interest, including any funding they received for conducting the review?	Yes	Yes

PICO =patient, intervention, comparator, outcome; RoB = risk of bias

Supplemental Content 2.21 Characteristics of Systematic Reviews Evaluated for PICO 3

Study	Date of Search and Databases Examined	# Trials and # participants included	Population	Comparison# randomized<br>Group 1: SUP<br>Group 2: Comparator	Main findings
Wang Y, 2020 (1)	Up to September 2019; MEDLINE, Embase, CENTRAL, CINAHL, trial registries.	72 RCTs; 12,660 participants	Critically ill adults in intensive care units	**Interventions (Group 1)**: Stress ulcer prophylaxis (SUP)—Proton Pump Inhibitors (PPIs), Histamine-2 Receptor Antagonists (H2RAs), sucralfate.<br>**Comparators (Group 2)**: Placebo or no prophylaxis	See Supplemental Content 2.22 below
Ying Wang 2020-updated SRMA	January 2019 – February 2020 (updated); Databases: MEDLINE, Embase, Web of Science, CENTRAL, ICTRP, LILACS, clinicaltrials.gov	74 RCTs; 39,569 participants	Adult critically ill patients in ICU at risk of gastrointestinal bleeding	**Comparison - Group 1 (SUP)**<br>Proton Pump Inhibitors (PPIs), Histamine-2 Receptor Antagonists (H2RAs), Sucralfate<br><br>**Comparison - Group 2 (Comparator)**<br>Placebo or no prophylaxis; alternative pharmacological agent (e.g., PPI vs. H2RA)	- Both PPIs (RR 1.03, 95% CrI 0.93–1.14, moderate certainty) and H2RAs (RR 0.98, 95% CrI 0.89–1.08, moderate certainty) probably have little or no impact on mortality compared with no prophylaxis.<br>- There may be no important difference in mortality between PPIs and H2RAs (RR 1.05, 95% CrI 0.97–1.14, low certainty), although a possible mortality increase with PPIs cannot be excluded.<br>- Both PPIs (RR 0.46, 95% CrI 0.29–0.66) and H2RAs (RR 0.67, 95% CrI 0.48–0.94) probably reduce clinically important GI bleeding.<br>- The reduction is probably greater with PPIs vs. H2RAs (RR 0.69, 95% CrI 0.45–0.93), especially in high-risk patients.

Study	Date of Search and Databases Examined	# Trials and # participants included	Population	Comparison# randomized Group 1: SUP Group 2: Comparator	Main findings
					- PPIs (RR 1.08, 95% CrI 0.88–1.45, low certainty) and H2RAs (RR 1.07, 95% CrI 0.85–1.37, low certainty) may have no important impact on pneumonia compared with no prophylaxis.<br>- No important difference among interventions for *C. difficile* infection, ICU/hospital stay, or duration of mechanical ventilation.

Supplemental Content 2.22 PICO 3: Evidence Profiles from Wang 2020

GRADE Summary of Findings for Clinically Important Gastrointestinal Bleeding (CIB)

**Comparison**	**Study results (95% CI)**	**Baseline risk (per 1000)**	**Absolute difference (95% CI) (per 1000)**	**Certainty in effect estimates**	**Plain text summary**
**PPIs vs. H2RAs**	RR 0.69 (0.45 to 0.93)	Low risk: 6[f]	–3 (–7 to 0)	Low[ad]	There may be no important difference
		Moderate risk: 14[f]	–6 (–17 to –1)	Low[ad]	Whether there is an important difference or not is very uncertain
		High risk: 28[f]	–13 (–34 to –1)	Low[ad]	There may be no important difference
		Highest risk: 41[f]	–18 (–50 to –3)	Low[ad]	There may be no important difference
**PPIs vs. Sucralfate**	RR 0.56 (0.32 to 0.88)	Low risk: 6[f]	–4 (–8 to –1)	Moderate[b]	There is probably no important difference
		Moderate risk: 14[f]	–11 (–30 to –2)	Low[ab]	There may be no important difference
		High risk: 28[f]	–22 (–60 to –4)	Moderate[b]	PPIs probably reduce CIB compared with sucralfate
		Highest risk: 41[f]	–32 (–87 to –6)	Moderate[b]	PPIs probably reduce CIB compared with sucralfate
**H2RAs vs. Sucralfate**	RR 0.81 (0.55 to 1.20)	Low risk: 8[f]	–2 (–7 to 1)	Low[ad]	There may be no important difference
		Moderate risk: 20[f]	–5 (–16 to 3)	Very low[ad]	Whether there is an important difference or not is very uncertain
		High risk: 40[f]	–9 (–33 to 7)	Low[ad]	There may be no important difference
		Highest risk: 60[f]	–14 (–49 to 10)	Low[ad]	There may be no important difference

[a] Rated down due to uncertainty in baseline risk for some risk factors
[b] Rated down for imprecision
[c] Rated down for the differences in results from different analyses/models as well as the uncertainty in baseline risk
[d] Rated down for risk of bias
[e] Rated down 2 levels for imprecision
[f] Baseline risk based on event rates in comparator groups (PPIs or H2RAs)

GRADE Summary of Findings for Overt Gastrointestinal Bleeding

Comparison	Study results (95% CI) and measurements	Absolute effect estimates (per 1000)		Absolute difference (95% CI) (per 1000)	Certainty in effect estimates	Plain text summary
PPIs vs. H2RAs	RR 0.76 (0.49 to 1.09)	Low risk	PPIs: 13[d]	-4 (-13 to 1)	Moderate[b]	PPIs likely reduce overt bleeding more than H2RAs
		Moderate risk	38[d]	-12 (-39 to 3)	Moderate[b]	PPIs likely reduce overt bleeding more than H2RAs
		High risk	63[d]	-20 (-65 to 5)	Moderate[b]	PPIs likely reduce overt bleeding more than H2RAs
		Highest risk	95[d]	-29 (-98 to 8)	Moderate[b]	PPIs likely reduce overt bleeding more than H2RAs
PPIs vs. sucralfate	RR 0.50 (0.27 to 0.84)	Low risk	PPIs: 13[d]	-13 (-36 to -2)	Moderate[a]	PPIs likely reduce overt bleeding compared with sucralfate
		Moderate risk	38[d]	-38 (-105 to -7)	Moderate[a]	PPIs likely reduce overt bleeding compared with sucralfate
		High risk	63[d]	-63 (-174 to -11)	Moderate[a]	PPIs likely reduce overt bleeding compared with sucralfate
		Highest risk	95[d]	-95 (-262 to -17)	Moderate[a]	PPIs likely reduce overt bleeding compared with sucralfate
H2RAs vs. sucralfate	RR 0.66 (0.41 to 1.02)	Low risk	H2RAs: 17[d]	-9 (-25 to 0)	Moderate[b]	H2RAs likely reduce overt bleeding compared with sucralfate
		Moderate risk	50[d]	-26 (-73 to 1)	Moderate[b]	H2RAs likely reduce overt bleeding compared with sucralfate
		High risk	83[d]	-43 (-121 to 2)	Moderate[b]	H2RAs likely reduce overt bleeding compared with sucralfate
		Highest risk	125[d]	-65 (-183 to 2)	Moderate[b]	H2RAs likely reduce overt bleeding compared with sucralfate

CI=confidence interval; GI= gastrointestinal; PPIs=proton pump inhibitors; RR=risk ratio; H2RAs=histamine-2 receptor antagonists.

[a] Rated down for the differences in results from different analyses as well as the uncertainty in baseline risk.

[b] Rated down for imprecision.
[c] Rated down 2 levels for imprecision.
[d] We used the point estimate of the PPIs group event rate in the comparison between PPIs and placebo as the baseline risk in the PPIs group in the PPIs vs. H2RAs and PPIs vs. sucralfate comparator to calculate the absolute effect for PPIs vs. H2RAs and PPIs vs. sucralfate, respectively. We used the point estimate of the H2RAs group event rate in the comparison between H2RAs and placebo as the baseline risk in the H2RAs group in the H2RAs vs. sucralfate comparator, to calculate absolute effect for H2RAs versus sucralfate.

GRADE Summary of Findings for Length of ICU Stay – Complete PEPTIC Analysis

Comparison	Direct estimate (95% CrI); Certainty of evidence[a]	Indirect estimate (95% CrI); Certainty of evidence[a]	Network estimate (95% CrI); Certainty of evidence[b]	Baseline risk (days)	Absolute difference (95% CrI) (days)
PPIs vs. H2RAs	1.01 (0.94 to 1.09); Moderate[c]	0.94 (0.76 to 1.14); Moderate[c]	1.00 (0.94 to 1.07); Moderate[c]	PPIs: 7.4[f]	0 (-0.5 to 0.5)
PPIs vs. sucralfate	1.01 (0.72 to 1.43); High	1.03 (0.92 to 1.17); Moderate[c]	1.03 (0.92 to 1.16); Moderate[d]	PPIs: 7.4[f]	0.2 (-0.6 to 1.0)
H2RAs vs. sucralfate	1.02 (0.91 to 1.16); High	1.05 (0.84 to 1.32); Moderate[c]	1.02 (0.93 to 1.14); High	H2RAs: 7.4[f]	0.1 (-0.6 to 0.9)

CrI=credible interval; PPIs=proton pump inhibitors; H2RAs=histamine-2 receptor antagonists.
[a] We did not consider imprecision when rating direct and indirect estimates because they were only used to inform the network estimates which we believed were the best estimates.
[b] Higher of direct or indirect confidence (without considering imprecision), followed by considering imprecision and incoherence.
[c] Rated down for risk of bias.
[d] Rated down for imprecision.
[e] Baseline risk comes from median of the placebo group in the included studies.
[f] We used the point estimate of the PPIs group event rate in the comparison between PPIs and placebo as the baseline risk in the PPIs group in the PPIs vs. H2RAs and PPIs vs. sucralfate comparator to calculate the absolute effect for PPIs vs. H2RAs and PPIs vs. sucralfate, respectively. We used the point estimate of the H2RAs group event rate in the comparison between H2RAs and placebo as the baseline risk in the H2RAs group in the H2RAs vs. sucralfate comparator to calculate the absolute effect for H2RAs vs. sucralfate.

GRADE Summary of Findings for Duration of Mechanical Ventilation - Complete PEPTIC Analysis

Comparison	Direct estimate (95% CrI); Certainty of evidence[a]	Indirect estimate (95% CrI); Certainty of evidence[a]	Network estimate (95% CrI); Certainty of evidence[b]	Baseline risk (days)	Absolute difference (95% CrI) (days)
PPIs vs. H2RAs	1.01 (0.93 to 1.12); Moderate[c]	1.09 (0.90 to 1.31); Moderate[c]	1.02 (0.95 to 1.12); Low[cd]	PPIs: 9.9[f]	0.2 (-0.5 to 1.1)
PPIs vs. sucralfate	1.03 (0.81 to 1.32); High	0.96 (0.86 to 1.11); Moderate[c]	0.97 (0.88 to 1.10); Low[cdg]	PPIs: 9.9[f]	-0.3 (-1.4 to 0.9)
H2RAs vs. sucralfate	0.95 (0.87 to 1.06); Moderate[c]	0.97 (0.79 to 1.19); Moderate[c]	0.95 (0.88 to 1.04); Low[cd]	H2RAs: 9.7[f]	-0.5 (-1.3 to 0.4)

CrI=credible interval; PPIs=proton pump inhibitors; H2RAs=histamine-2 receptor antagonists.

[a] We did not consider imprecision when rating direct and indirect estimates because they were only used to inform the network estimates which we believed were the best estimates.

[b] Higher of direct or indirect confidence (without considering imprecision), followed by considering imprecision and incoherence.

[c] Rated down for risk of bias.

[d] Rated down for imprecision.

[e] Baseline risk comes from median of the placebo group in the included studies.

[f] We used the point estimate of the PPIs group event rate in the comparison between PPIs and placebo as the baseline risk in the PPIs group in the PPIs vs. H2RAs and PPIs vs. sucralfate comparator to calculate the absolute effect for PPIs vs. H2RAs and PPIs vs. sucralfate, respectively. We used the point estimate of the H2RAs group event rate in the comparison between H2RAs and placebo as the baseline risk in the H2RAs group in the H2RAs vs. sucralfate comparator to calculate the absolute effect for H2RAs vs. sucralfate.

[g] The indirect evidence contributed much more than direct evidence, so certainty of network started rating from the certainty of indirect estimate.

GRADE Summary of Findings: Desirable Effects Including IV/PO Route & Dose-Based Comparison

Comparison	Outcome	Direct Estimate (95% CrI)	Node-splitting Estimate (95% CrI)	Certainty (Direct)	Indirect Estimate (95% CrI)	Certainty (Indirect)	NMA Estimate (95% CrI)	Certainty (NMA)	Plain text summary
**PPI PO vs. H2RA PO/IV**	CIGIB	0.09 (0.01, 0.68)	Not estimable	Moderate[a]	Not estimable	–	0.15 (0.02, 0.66)	Very low[b]	PPIs PO lead to less bleeding than H2RA or sucralfate. PPIs PO vs. H2RAs IV: the desirable effects were moderate.
	Overt GIB	0.09 (0.01, 0.68)	Not estimable	Moderate[a]	Not estimable	–	0.15 (0.02, 0.66)	Very low[b]	PPIs PO lead to less bleeding than H2RAs or sucralfate. PPIs PO vs. H2RAs IV: the desirable effects were moderate.
**Sucralfate vs. PPI IV**	CIGIB	3.13 (0.33, 29.37)	1.95 (0.28, 15.3)	High	1.41 (0.67, 3.05)	High	1.54 (0.77, 3.18)	Low[b]	PPIs PO lead to less bleeding than H2RAs or sucralfate. Sucralfate vs. PPI IV: the desirable effects were trivial.
	Overt GIB	3.13 (0.33, 29.37)	1.95 (0.28, 15.3)	High	1.41 (0.67, 3.05)	High	1.54 (0.77, 3.18)	Low[b]	PPIs PO lead to less bleeding than H2RAs or sucralfate.
**Sucralfate vs. H2RA IV**	CIGIB	1.11 (0.68, 1.82)	0.95 (0.56, 1.51)	High	0.92 (0.06, 9.76)	High	1.01 (0.60, 1.56)	Moderate[a]	Sucralfate vs. H2RAs IV: the desirable effects were trivial.
	Overt GIB	1.11 (0.68, 1.82)	0.95 (0.56, 1.51)	High	0.92 (0.06, 9.76)	High	1.01 (0.60, 1.56)	Moderate[a]	
**PPI High Dose vs. H2RA Low Dose**	CIGIB	0.22 (0.07, 0.73)	0.21 (0.05, 0.67)	Moderate	0.38 (0.09, 1.48)	Moderate	0.26 (0.10, 0.63)	Low	Low dose and high dose PPIs lead to less bleeding events. PPIs high dose vs. H2RAs low dose: desirable effects are moderate.

	Overt GIB	0.22 (0.07, 0.73)	0.21 (0.05, 0.67)	Moderate	0.38 (0.09, 1.48)	Moderate	0.26 (0.10, 0.63)	Low	Low dose and high dose PPIs lead to less bleeding events.<br>PPIs high dose vs. H2RAs low dose: desirable effects are moderate.
**PPI Low Dose vs. H2RA Low Dose**	CIGIB	0.94 (0.10, 8.86)	0.37 (0.03, 2.77)	Moderate	0.31 (0.11, 0.79)	Moderate	0.32 (0.12, 0.74)	Very low	Low dose and high dose PPIs lead to less bleeding events. PPIs low dose vs. H2RAs low dose: desirable effects are moderate.
	Overt GIB	0.94 (0.10, 8.86)	0.37 (0.03, 2.77)	Moderate	0.31 (0.11, 0.79)	Moderate	0.32 (0.12, 0.74)	Very low	Low dose and high dose PPIs lead to less bleeding events. PPIs low dose vs. H2RAs low dose: desirable effects are moderate.
**H2RA High Dose vs. PPI High Dose**	CIGIB	1.40 (0.55, 3.61)	1.24 (0.37, 4.01)	Moderate	3.46 (0.91, 16.9)	Moderate	1.89 (0.81, 4.96)	Low	H2RAs high dose vs. PPIs high dose: desirable effects are trivial.
	Overt GIB	1.40 (0.55, 3.61)	1.24 (0.37, 4.01)	Moderate	3.45 (0.91, 16.9)	Moderate	1.89 (0.81, 4.96)	Low	H2RAs high dose vs. PPIs high dose: desirable effects are trivial.

CrI=Credible Interval; NMA=Network Meta-analysis; CIGIB=Clinically Important Gastrointestinal Bleeding; PPIs=Proton Pump Inhibitors; H2RAs=Histamine-2 Receptor Antagonists.

[a] Rated down for serious imprecision in direct estimates (e.g., wide CrIs, low event numbers).

[b] Rated down 2 levels for imprecision and/or incoherence in NMA estimates.

Evidence profile and NMA for Undesirable Effect

Mortality - Complete PEPTIC Analysis

Comparison	Direct estimate (95% CrI); certainty of evidence	Indirect estimate (95% CrI); certainty of evidence	Network estimate (95% CrI); certainty of evidence	Baseline risk (per 1000)	Absolute difference (95% CrI) (per 1000)
**PPIs vs. H2RAs**	1.06 (0.95 to 1.18); **Moderate**[d]	1.03 (0.86 to 1.23); **High**	1.05 (0.97 to 1.14); **Low**[eg]	PPIs: 313[h]	15 (–10 to 38)
**PPIs vs. sucralfate**	0.99 (0.61 to 1.61); **High**	1.13 (0.97 to 1.32); **Moderate**[d]	1.11 (0.96 to 1.28); **Low**[eg]	PPIs: 313[h]	31 (–13 to 68)
**H2RAs vs. sucralfate**	1.08 (0.94 to 1.26); **High**	0.99 (0.75 to 1.32); **High**	1.05 (0.93 to 1.20); **Low**[eg]	H2RAs: 298[h]	14 –22 to 50)

CrI=Credible Interval; PPIs=Proton Pump Inhibitors; H2RAs=Histamine-2 Receptor Antagonists.

a: Imprecision not considered for direct/indirect estimates, used only to inform network estimates.

b: Network estimate rating based on higher of direct/indirect certainty, considering imprecision and incoherence.

c: Baseline risk from placebo group in SUP-ICU trial.

d: Rated down for risk of bias.

e: Rated down for imprecision.

f: Rated down 2 levels for imprecision.

g: Rated down for inconsistency between analyses/models.

h: Baseline risk derived from event rates in PPI or H2RA comparator group.

GRADE Summary of Findings for Pneumonia					
**Comparison**	**Direct estimate (95% CrI); Certainty of evidence[a]**	**Indirect estimate (95% CrI); Certainty of evidence[a]**	**Network estimate (95% CrI); Certainty of evidence[b]**	**Baseline risk (per 1000)**	**Absolute difference (95% CrI) (per 1000)**
PPIs vs. H2RAs	1.07 (0.76 to 1.52); High	0.97 (0.68 to 1.53); High	1.02 (0.80 to 1.33); Low[de]	PPIs: 175[f]	3 (-44 to 43)
PPIs vs. sucralfate	1.28 (0.47 to 3.48); High	1.16 (0.77 to 1.80); High	1.17 (0.81 to 1.74); Low[de]	PPIs: 175[f]	25 (-41 to 74)
H2RAs vs. sucralfate	1.17 (0.78 to 1.74); High	1.03 (0.45 to 2.34); High	1.15 (0.83 to 1.57); Low[de]	H2RAs: 173[f]	23 (-35 to 63)

CrI=credible interval; PPIs=proton pump inhibitors; H2RAs=histamine-2 receptor antagonists.

[a] We did not consider imprecision when rating direct and indirect estimates because they were only used to inform the network estimates which we believed were the best estimates.

[b] Higher of direct or indirect confidence (without considering imprecision), followed by considering imprecision and incoherence.

[c] We used as baseline risk in the placebo group of the SUP-ICU trial.

[d] Rated down for imprecision.

[e] Rated down because results dependent on a series of methods judgments (believe the meta-regression and use only the blinded studies).

[f] We used the point estimate of the PPIs group event rate in the comparison between PPIs and placebo as the baseline risk in the PPIs group in the PPIs vs. H2RAs and PPIs vs. sucralfate comparator to calculate the absolute effect for PPIs vs. H2RAs and PPIs vs. sucralfate, respectively. We used the point estimate of the H2RAs group event rate in the comparison between H2RAs and placebo as the baseline risk in the H2RAs group in the H2RAs vs. sucralfate comparator to calculate the absolute effect for H2RAs vs. sucralfate.

GRADE Summary of Findings for *Clostroides difficile* Infection – Complete PEPTIC Analysis					
**Comparison**	**Direct estimate (95% CrI); Certainty of evidence[a]**	**Indirect estimate (95% CrI); Certainty of evidence[a]**	**Network estimate (95% CrI); Certainty of evidence[b]**	**Baseline risk (per 1000)**	**Absolute difference (95% CrI) (per 1000)**
PPIs vs. H2RAs	0.76 (0.29 to 2.15); Moderate[d]	NA	0.76 (0.28 to 2.16); Low[de]	PPIs: 12[f]	-4 (-31 to 6)

CrI=credible interval; PPIs=proton pump inhibitors; NA=not applicable; H2RAs=histamine-2 receptor antagonists.

[a] We did not consider imprecision when rating direct and indirect estimates because they were only used to inform the network estimates which we believed were the best estimates.

[b] Higher of direct or indirect confidence (without considering imprecision), Followed by considering imprecision and incoherence.

[c] We used as baseline risk in the placebo group of the SUP-ICU trial.

[d] Rated down for risk of bias.

[e] Rated down for imprecision.

[f] We used the point estimate of the PPIs group event rate in the comparison between PPIs and placebo as the baseline risk in the PPIs group in the PPIs vs. H2RAs comparator to calculate the absolute effect for PPIs vs. H2RAs.

GRADE Summary of Findings: Undesirable Effects Including IV/PO Route & Dose-Based Comparison

Comparison	Outcome	Direct Estimate (95% CrI)	Node-splitting Estimate (95% CrI)	Certainty (Direct)	Indirect Estimate (95% CrI)	Certainty (Indirect)	NMA Estimate (95% CrI)	Certainty (NMA)	Comments
**PPI PO vs. H2RA PO/IV**	Mortality	0.11 (0.01, 2.03)	Not estimable	Moderate[a]	Not estimable	–	0.19 (0.02, 1.25)	Very low[b]	PPIs PO vs. H2RAs IV: the undesirable effects were trivial
	Pneumonia	0.23 (0.05, 1.17)	Not estimable	Moderate[a]	Not estimable	–	0.30 (0.06, 1.38)	Very low[b]	PPIs PO vs. H2RAs IV: the undesirable effects were trivial
**Sucralfate vs. PPI IV**	Mortality	1.32 (0.62, 2.82)	1.26 (0.72, 2.24)	Moderate[a]	0.81 (0.63, 1.03)	High	0.87 (0.70, 1.07)	Low[b]	Sucralfate vs. PPIs IV: the undesirable effects were small
	Pneumonia	0.52 (0.23, 1.20)	0.47 (0.22, 0.95)	Moderate[a]	0.79 (0.53, 1.10)	High	0.72 (0.51, 0.97)	Low[b]	Sucralfate was associated with less pneumonia Sucralfate vs. PPIs IV: the undesirable effects were small
**Sucralfate vs. H2RA IV**	Mortality	0.96 (0.83, 1.11)	0.96 (0.82, 1.12)	High	0.65 (0.21, 1.79)	High	0.97 (0.83, 1.13)	Moderate[a]	Sucralfate vs. H2RAs IV: the undesirable effects were small
	Pneumonia	0.83 (0.71, 0.96)	0.77 (0.50, 0.94)	High	1.63 (0.09, 31.0)	High	0.78 (0.63, 0.95)	High	Sucralfate was associated with less pneumonia Sucralfate vs. H2RAs IV: the undesirable effects were small
**PPI High Dose vs. H2RA Low Dose**	Mortality	0.96 (0.77, 1.20)	0.95 (0.74, 1.21)	Moderate	1.82 (1.18, 2.83)	Moderate	1.10 (0.89, 1.38)	Low	No difference in mortality or pneumonia PPIs high dose vs. H2RAs low dose: undesirable effects are trivial
	Pneumonia	1.20 (0.73, 1.98)	1.16 (0.65, 2.09)	Moderate	1.40 (0.65, 2.63)	Moderate	1.28 (0.85, 1.94)	Low	No difference in mortality or pneumonia PPIs high dose vs. H2RAs low dose:

									undesirable effects are trivial
**PPI Low Dose vs. H2RA Low Dose**	Mortality	2.00 (0.52, 7.63)	1.76 (0.51, 7.01)	Moderate	1.19 (0.89, 1.58)	Moderate	1.21 (0.92, 1.62)	Very low	No difference in mortality or pneumonia PPIs low dose vs. H2RAs low dose: undesirable effects are trivial
	Pneumonia	0.33 (0.14, 0.78)	0.33 (0.12, 0.80)	Moderate	0.87 (0.55, 1.29)	Moderate	0.71 (0.45, 1.04)	Very low	No difference in mortality or pneumonia PPIs low dose vs. H2RAs low dose: undesirable effects are trivial
**H2RA High Dose vs. PPI High Dose**	Mortality	1.22 (0.62, 2.41)	1.26 (0.85, 1.87)	Moderate	1.22 (0.89, 1.67)	Moderate	1.05 (0.81, 1.38)	Low	No difference in mortality or pneumonia H2RAs high dose vs. PPIs high dose: undesirable effects are trivial
	Pneumonia	0.96 (0.61, 1.50)	0.89 (0.52, 1.47)	Moderate	0.82 (0.44, 1.49)	Moderate	0.90 (0.58, 1.34)	Low	No difference in mortality or pneumonia H2RAs high dose vs. PPIs high dose: undesirable effects are trivial

CrI=Credible Interval; NMA=Network Meta-analysis; CIGIB=Clinically Important Gastrointestinal Bleeding; PPIs=Proton Pump Inhibitors; H2RAs=Histamine-2 Receptor Antagonists.

[a] Rated down for serious imprecision in direct estimates (e.g., wide CrIs, low event numbers).

[b] Rated down 2 levels for imprecision and/or incoherence in NMA estimates.

Supplemental Content 2.23 NMA From Source Guideline for PICO 3

1- Agents: https://gdt.gradepro.org/presentations/#/nma/nma_question_1f57d3a1-f4e6-43a3-9f1e-f7900d9566e3
2- Route: https://gdt.gradepro.org/presentations/#/nma/nma_question_8d3fd775-9e1b-4580-8660-da9bf29d1f04
3- Dose: https://gdt.gradepro.org/presentations/#/nma/nma_question_0bd7e2d0-9cbf-4cf1-ada2-ebf3d232498d

Intervention	clinically important gastrointestinal bleeding (CIB) for highest risk population– complete PEPTIC analysis<br>T/S -- per 1000<br>S/M -- per 1000<br>M/L -- per 1000	CIB – High risk population - complete PEPTIC analysis<br>T/S -- per 1000<br>S/M -- per 1000<br>M/L -- per 1000	CIB- moderate risk population-complete PEPTIC analysis<br>T/S -- per 1000<br>S/M -- per 1000<br>M/L -- per 1000	Overt gastrointestinal bleeding- highest risk of bleeding<br>T/S -- per 1000<br>S/M -- per 1000<br>M/L -- per 1000	Overt gastrointestinal bleeding-High risk population<br>T/S -- per 1000<br>S/M -- per 1000<br>M/L -- per 1000	Overt gastrointestinal bleeding- moderate risk population<br>T/S -- per 1000<br>S/M -- per 1000<br>M/L -- per 1000	length of ICU stay- complete PEPTIC analysis<br>Difference -- or more<br>Difference -- or more<br>Difference -- or more	Duration of mechanical ventilation- complete PEPTIC analysis<br>Difference -- or more<br>Difference -- or more<br>Difference -- or more
Proton pump inhibitor vs histamine-2 receptor antagonists	NMA<br>18 fewer<br>50 fewer to 3 fewer<br>RR 0.69<br>(0.45, 0.93)<br>LOW	NMA<br>13 fewer<br>34 fewer to 2 fewer<br>RR 0.69<br>(0.45, 0.93)<br>LOW	NMA<br>6 fewer<br>17 fewer to 1 fewer<br>RR 0.69<br>(0.45, 0.93)<br>VERY LOW	NMA<br>29 fewer<br>98 fewer to 8 more<br>RR 0.76<br>(0.49, 1.09)<br>MODERATE	NMA<br>20 fewer<br>65 fewer to 5 more<br>RR 0.76<br>(0.49, 1.09)<br>MODERATE	NMA<br>12 fewer<br>39 fewer to 3 more<br>RR 0.76<br>(0.49, 1.09)<br>MODERATE	NMA<br>MD 1<br>(0.94, 1.07)<br>MODERATE	NMA<br>MD 1.02<br>(0.95, 1.12)<br>LOW
Proton pump inhibitor vs sucralfate	NMA<br>32 fewer<br>87 fewer to 6 fewer<br>RR 0.56<br>(0.32, 0.88)<br>MODERATE	NMA<br>22 fewer<br>60 fewer to 4 fewer<br>RR 0.56<br>(0.32, 0.88)<br>MODERATE	NMA<br>11 fewer<br>30 fewer to 2 fewer<br>RR 0.56<br>(0.32, 0.88)<br>MODERATE	NMA<br>95 fewer<br>262 fewer to 17 fewer<br>RR 0.5<br>(0.27, 0.84)<br>MODERATE	NMA<br>63 fewer<br>174 fewer to 11 fewer<br>RR 0.5<br>(0.27, 0.84)<br>MODERATE	NMA<br>38 fewer<br>105 fewer to 7 fewer<br>RR 0.5<br>(0.27, 0.84)<br>MODERATE	NMA<br>MD 1.03<br>(0.92, 1.16)<br>MODERATE	NMA<br>MD 0.97<br>(0.88, 1.1)<br>LOW
histamine-2 receptor antagonists vs Proton pump inhibitor	NMA<br>18 more<br>3 more to 50 more<br>RR 1.45<br>(1.08, 2.22)<br>LOW	NMA<br>13 more<br>2 more to 34 more<br>RR 1.45<br>(1.08, 2.22)<br>LOW	NMA<br>6 more<br>1 more to 17 more<br>RR 1.45<br>(1.08, 2.22)<br>VERY LOW	NMA<br>29 more<br>8 fewer to 98 more<br>RR 1.32<br>(0.92, 2.04)<br>MODERATE	NMA<br>20 more<br>5 fewer to 65 more<br>RR 1.32<br>(0.92, 2.04)<br>MODERATE	NMA<br>12 more<br>3 fewer to 39 more<br>RR 1.32<br>(0.92, 2.04)<br>MODERATE	NMA<br>MD -1<br>(-1.07, -0.94)<br>MODERATE	NMA<br>MD -1.02<br>(-1.12, -0.95)<br>LOW
histamine-2 receptor antagonists vs sucralfate	NMA<br>14 fewer<br>49 fewer to 10 more<br>RR 0.81<br>(0.55, 1.2)<br>LOW	NMA<br>9 fewer<br>33 fewer to 7 more<br>RR 0.81<br>(0.55, 1.2)<br>LOW	NMA<br>5 fewer<br>16 fewer to 3 more<br>RR 0.81<br>(0.55, 1.2)<br>VERY LOW	NMA<br>65 fewer<br>183 fewer to 2 more<br>RR 0.66<br>(0.41, 1.02)<br>MODERATE	NMA<br>43 fewer<br>121 fewer to 2 more<br>RR 0.66<br>(0.41, 1.02)<br>MODERATE	NMA<br>26 fewer<br>73 fewer to 1 more<br>RR 0.66<br>(0.41, 1.02)<br>MODERATE	NMA<br>MD 1.02<br>(0.93, 1.14)<br>HIGH	NMA<br>MD 0.97<br>(0.88, 1.1)
sucralfate vs Proton pump inhibitor	NMA<br>32 more<br>6 more to 87 more<br>RR 1.79<br>(1.14, 3.13)<br>MODERATE	NMA<br>22 more<br>4 more to 60 more<br>RR 1.79<br>(1.14, 3.13)<br>MODERATE	NMA<br>11 more<br>2 more to 30 more<br>RR 1.79<br>(1.14, 3.13)<br>MODERATE	NMA<br>95 more<br>17 more to 262 more<br>RR 2<br>(1.19, 3.7)<br>MODERATE	NMA<br>63 more<br>11 more to 174 more<br>RR 2<br>(1.19, 3.7)<br>MODERATE	NMA<br>38 more<br>7 more to 105 more<br>RR 2<br>(1.19, 3.7)<br>MODERATE	NMA<br>MD -1.03<br>(-1.16, -0.92)<br>MODERATE	NMA<br>MD -0.97<br>(-1.1, -0.88)<br>LOW
sucralfate vs histamine-2 receptor antagonists	NMA<br>14 more<br>10 fewer to 49 more<br>RR 1.23<br>(0.83, 1.82)<br>LOW	NMA<br>9 more<br>7 fewer to 33 more<br>RR 1.23<br>(0.83, 1.82)<br>LOW	NMA<br>5 more<br>3 fewer to 16 more<br>RR 1.23<br>(0.83, 1.82)<br>VERY LOW	NMA<br>65 more<br>2 fewer to 183 more<br>RR 1.52<br>(0.98, 2.44)<br>MODERATE	NMA<br>43 more<br>2 fewer to 121 more<br>RR 1.52<br>(0.98, 2.44)<br>MODERATE	NMA<br>26 more<br>1 fewer to 73 more<br>RR 1.52<br>(0.98, 2.44)<br>MODERATE	NMA<br>MD -1.02<br>(-1.14, -0.93)<br>HIGH	NMA<br>MD -0.97<br>(-1.1, -0.88)

Intervention	Clinically Important Gastrointestinal Bleeding<br>T/S -- per 1000<br>S/M -- per 1000<br>M/L -- per 1000	Overt GIB<br>T/S -- per 1000<br>S/M -- per 1000<br>M/L -- per 1000
PPI PO vs H2RA PO/IV	NMA<br>**RR 0.15**<br>(0.02, 0.66)<br>**VERY LOW**	NMA<br>**RR 0.15**<br>(0.02, 0.66)<br>**VERY LOW**
PPI PO vs PPI IV	No data	No data
PPI PO vs Sucralfate	No data	No data
Sucralfate vs PPI PO	No data	No data
Sucralfate vs H2RA PO/IV	NMA<br>**RR 1.01**<br>(0.6, 1.56)<br>**MODERATE**	NMA<br>**RR 1.01**<br>(0.6, 1.56)<br>**MODERATE**
Sucralfate vs PPI IV	NMA<br>**RR 1.54**<br>(0.77, 3.18)<br>**LOW**	NMA<br>**RR 1.54**<br>(0.77, 3.18)<br>**LOW**

Intervention	CIGIB<br>T/S -- per 1000<br>S/M -- per 1000<br>M/L -- per 1000	Overt GIB<br>T/S -- per 1000<br>S/M -- per 1000<br>M/L -- per 1000
PPI low dose vs PPI high dose	No data	No data
PPI low dose vs H2RA high dose	No data	No data
PPI low dose vs H2RA low dose	NMA<br>**RR 0.32**<br>(0.12, 0.74)<br>**VERY LOW**	NMA<br>**RR 0.32**<br>(0.12, 0.74)<br>**LOW**

Intervention	mortality—complete PEPTIC analysis<br>T/S 12 per 1000<br>S/M 30 per 1000<br>M/L 60 per 1000	Pneumonia<br>T/S 175 per 1000<br>S/M -- per 1000<br>M/L -- per 1000	Clostroides difficile infection – complete PEPTIC analysis<br>T/S -- per 1000<br>S/M -- per 1000<br>M/L -- per 1000
Proton pump inhibitor vs histamine-2 receptor antagonists	NMA<br>**15 more**<br>10 fewer to 38 more<br>**RR 1.05**<br>(0.97, 1.14)<br>**LOW**	NMA<br>**3 more**<br>44 fewer to 43 more<br>**RR 1.02**<br>(0.8, 1.33)<br>**LOW**	NMA<br>**4 fewer**<br>31 fewer to 6 more<br>**RR 0.76**<br>(0.28, 2.16)<br>**LOW**
Proton pump inhibitor vs sucralfate	NMA<br>**31 more**<br>13 fewer to 68 more<br>**RR 1.11**<br>(0.96, 1.28)<br>**LOW**	NMA<br>**25 more**<br>41 fewer to 74 more<br>**RR 1.17**<br>(0.81, 1.74)<br>**LOW**	No data
Proton pump inhibitor vs placebo	NMA<br>**9 more**<br>21 fewer to 43 more<br>**RR 1.03**<br>(0.93, 1.14)<br>**MODERATE**	NMA<br>**13 more**<br>19 fewer to 73 more<br>**RR 1.08**<br>(0.88, 1.45)<br>**LOW**	NMA<br>**3 fewer**<br>10 fewer to 18 more<br>**RR 0.82**<br>(0.34, 2.21)<br>**MODERATE**

Intervention	mortality—complete PEPTIC analysis<br>T/S 12 per 1000<br>S/M 30 per 1000<br>M/L 60 per 1000	Pneumonia<br>T/S 175 per 1000<br>S/M -- per 1000<br>M/L -- per 1000	Clostroides difficile infection – complete PEPTIC analysis<br>T/S -- per 1000<br>S/M -- per 1000<br>M/L -- per 1000
sucralfate vs Proton pump inhibitor	NMA<br>**31 fewer**<br>68 fewer to 13 more<br>**RR 0.9**<br>(0.78, 1.04)<br>**LOW**	NMA<br>**25 fewer**<br>74 fewer to 41 more<br>**RR 0.85**<br>(0.57, 1.23)<br>**LOW**	No data
sucralfate vs histamine-2 receptor antagonists	NMA<br>**14 fewer**<br>50 fewer to 22 more<br>**RR 0.95**<br>(0.83, 1.08)<br>**LOW**	NMA<br>**23 fewer**<br>63 fewer to 35 more<br>**RR 0.87**<br>(0.64, 1.2)<br>**LOW**	No data
sucralfate vs placebo	NMA<br>**21 fewer**<br>61 fewer to 21 more<br>**RR 0.93**<br>(0.8, 1.07)<br>**VERY LOW**	No data	No data

Intervention	Mortality<br>T/S -- per 1000<br>S/M -- per 1000<br>M/L -- per 1000	Pneumonia<br>T/S -- per 1000<br>S/M -- per 1000<br>M/L -- per 1000
PPI PO vs H2RA PO/IV	NMA<br>**RR 0.19**<br>(0.02, 1.25)<br>VERY LOW	NMA<br>**RR 0.3**<br>(0.06, 1.38)<br>VERY LOW
PPI PO vs PPI IV	No data	No data
PPI PO vs Sucralfate	No data	No data
Sucralfate vs PPI PO	No data	No data
Sucralfate vs H2RA PO/IV	NMA<br>**RR 0.97**<br>(0.83, 1.13)<br>MODERATE	NMA<br>**RR 0.78**<br>(0.63, 0.95)<br>HIGH
Sucralfate vs PPI IV	NMA<br>**RR 0.87**<br>(0.7, 1.07)<br>LOW	NMA<br>**RR 0.72**<br>(0.51, 0.97)<br>LOW

Intervention	Mortality<br>T/S -- per 1000<br>S/M -- per 1000<br>M/L -- per 1000	Pneumonia<br>T/S -- per 1000<br>S/M -- per 1000<br>M/L -- per 1000
PPI low dose vs PPI high dose	No data	No data
PPI low dose vs H2RA high dose	No data	No data
PPI low dose vs H2RA low dose	NMA<br>**RR 1.21**<br>(0.92, 1.62)<br>VERY LOW	NMA<br>**RR 0.71**<br>(0.45, 1.04)<br>VERY LOW

Supplemental Content 2.24 PICO 3: Evidence-to-Decision (EtD) Framework

In critically ill adults in the ICU with risk factors for stress-related upper gastrointestinal bleeding (UGIB), what are the comparative effectiveness and harms of stress ulcer prophylaxis (SUP) agents (PPIs, H2RAs, or sucralfate) when used at low doses versus high doses or administered via enteral versus intravenous routes?	
**POPULATION:**	ICU
**INTERVENTION:**	PPI vs. H2RA, PPI vs. Sucralfate, H2RAs vs. Sucralfate, PPIs PO vs. H2RAs PO/IV, Sucralfate vs. PPIs IV, Sucralfate vs. H2RAs IV, PPIs High Dose vs. H2RAs Low Dose, PPIs Low Dose vs. H2RAs Low Dose, H2RAs High Dose vs. PPIs High Dose
**COMPARISON:**	As stated above
**OUTCOMES:**	Desirable effect: Clinically Important Gastrointestinal Bleeding (CIGIB); Desirable effect: Overt bleeding; Desirable effect: Length of ICU stay; Desirable effect: duration of mechanical ventilation; Undesirable effect: mortality; Undesirable effect: pneumonia; Undesirable effect: *Clostroides difficile* infection
**AUTHORS:**	Marwa Amer
**SETTING:**	ICU
**PERSPECTIVE:**	Clinicians
**BACKGROUND:**	There is a growing consensus toward a risk-stratified and individualized approach to SUP, weighing the benefits of GI bleeding prevention against potential harms and resource use. This review aims to assess the comparative effectiveness and safety of various SUP agents—specifically PPIs, H2RAs, and sucralfate—when used at low doses and administered enterally versus intravenously—in critically ill adults with risk factors for stress-related UGIB.
**CONFLICT OF INTERESTS:**	Some panel members are co-authors of one of the largest trials on stress ulcer prophylaxis (the SUP-ICU trial), REVISE trial, or the 2024 SCCM and ASHP "Guideline for the Prevention of Stress-Related Gastrointestinal Bleeding in Critically Ill Adults"

ASSESSMENT

Problem<br>Is the problem a priority?		
**JUDGEMENT**	**RESEARCH EVIDENCE**	**ADDITIONAL CONSIDERATIONS**
○ No<br>○ Probably no<br>○ Probably yes<br>● Yes<br>○ Varies<br>○ Don't know	There is a growing consensus toward a risk-stratified and individualized approach to SUP, weighing the benefits of GI bleeding prevention against potential harms and resource use. This review aims to assess the comparative effectiveness and safety of various SUP agents—specifically PPIs, H2RAs, and sucralfate—when used at low doses and administered enterally versus intravenously in critically ill adults with risk factors for stress-related UGIB.	

Desirable Effects
How substantial are the desirable anticipated effects?

JUDGEMENT

Trivial: H2RAs vs. Sucralfate, Sucralfate vs. H2RAs IV, H2RAs High Dose vs. PPIs High Dose, Sucralfate vs. PPIs IV
Small: PPIs vs. H2RAs
Moderate: PPIs Low Dose vs. H2RAs Low Dose, PPIs High Dose vs. H2RAs Low Dose, PPIs vs. Sucralfate, PPIs PO vs. H2RAs PO/IV
Large:
Varies:
Don't know:

RESEARCH EVIDENCE

How substantial are the desirable anticipated effects?

	Desirable effect: Clinically Important Gastrointestinal Bleeding (CIGIB)	Desirable effect: Overt bleeding	Desirable effect: Length of ICU stay	Desirable effect: duration of mechanical ventilation	Undesirable effect: mortality	Undesirable effect: pneumonia	Undesirable effect: Clostroides difficile infection	Overall
PPI vs. H2RA	SMALL	SMALL	TRIVIAL	TRIVIAL	—	—	—	SMALL
PPI vs. Sucralfate	MODERATE	MODERATE	TRIVIAL	TRIVIAL	—	—	—	MODERATE
H2RAs vs Sucralfate	TRIVIAL	MODERATE	TRIVIAL	TRIVIAL	—	—	—	TRIVIAL
PPI PO vs H2RA PO/IV	MODERATE	MODERATE	DON'T KNOW	DON'T KNOW	—	—	—	MODERATE
Sucralfate vs PPI IV	TRIVIAL	TRIVIAL	DON'T KNOW	DON'T KNOW	—	—	—	TRIVIAL
Sucralfate vs H2RA IV	TRIVIAL	TRIVIAL	DON'T KNOW	DON'T KNOW	—	—	—	TRIVIAL
PPI High Dose vs H2RA Low Dose	MODERATE	MODERATE	DON'T KNOW	DON'T KNOW	—	—	—	MODERATE
PPI Low Dose vs H2RA Low Dose	MODERATE	MODERATE	DON'T KNOW	DON'T KNOW	—	—	—	MODERATE
H2RA High Dose vs PPI High Dose	TRIVIAL	TRIVIAL	DON'T KNOW	DON'T KNOW	—	—	—	TRIVIAL

Agent
PPIs vs. H2RAs vs. Sucralfate for Clinically Important Bleeding (CIB)

- PPIs may reduce clinically important gastrointestinal bleeding (CIB) compared to H2RAs, but the difference is uncertain, particularly in low- and moderate-risk patients. Across all risk strata (low, moderate, high, and highest), the certainty of evidence is low to moderate, and absolute differences are small to modest (e.g., 3 to 13 fewer cases per 1000 patients). For patients at low (6/1000) or moderate (14/1000) baseline risk, there may be no important difference between PPIs and H2RAs.
- In contrast, PPIs probably reduce CIB compared to sucralfate, particularly in patients at high (28/1000) and highest risk (41/1000), with moderate certainty of evidence. The absolute risk reductions in these groups are more substantial—up to 32 fewer cases per 1000 in the highest-risk group—suggesting a clinically meaningful benefit.

ADDITIONAL CONSIDERATIONS

"We acknowledge the concern regarding potential confounding due to differences in illness severity across studies comparing oral and IV agents. The observed benefits of oral PPIs versus IV H2RAs or sucralfate may be influenced by lower baseline risk in patients included in oral trials. As most of the evidence derives from indirect comparisons in NMAs, and population comparability is uncertain, caution is warranted in interpreting these results. We have flagged this as a source of indirectness and potential bias in the evidence assessment."

The panel agreed with the pre-selected judgments on desirable

	• **Conclusion**: **both PPIs and H2RAs likely reduce CIB; the magnitude of the effect is probably greater with PPIs, particularly for patients at higher risk of bleeding. PPIs may also reduce overt bleeding more than both H2RAs and sucralfate. There is no significant difference between the interventions in terms of ICU or hospital stay duration or mechanical ventilation.**<br><br>Route of Administration Comparisons<br><br>• **Oral PPIs** are associated with less bleeding compared to **IV H2RAs** and **sucralfate**.<br>• When comparing **PO PPIs with IV H2RAs**, the **desirable effects are moderate**.<br>• **Sucralfate vs. IV PPIs** and **sucralfate vs. IV H2RAs** showed **trivial benefits**, suggesting minimal differences in bleeding outcomes.<br><br>Dose Comparisons and Bleeding Risk<br><br>• Both **low- and high-dose PPIs** are associated with fewer bleeding events compared to **low-dose H2RAs**; the **desirable effects are moderate**.<br>• When comparing **high-dose H2RAs with high-dose PPIs**, the **desirable effects are trivial**, indicating little or no benefit of high-dose H2RAs over PPIs.<br><br>**Evidence profiles** (1, 2, 3)	**effects across comparisons:**<br><br>• Small to moderate benefit for PPIs vs. H2RAs<br>• Moderate benefit for PPIs vs. sucralfate<br>• Trivial benefit for H2RAs vs. sucralfate and for many dosing/route comparisons<br>• **For Route of Administration:** The panel agreed that these comparisons rely heavily on indirect evidence and that confounding by illness severity (i.e., sicker patients more likely to receive IV therapy) may have biased results. This was flagged as a potential source of indirectness, and caution in interpretation was advised.

Comparison	Study results (95% CI)	Baseline risk (per 1000)	Absolute difference (95% CI) (per 1000)	Certainty in effect estimates	Plain text summary
**PPIs vs H2RAs**	RR 0.69 (0.45 to 0.93)	Low risk: 6[f]	–3 (–7 to 0)	Low[ad]	There may be no important difference in CIB
		Moderate risk: 14[f]	–6 (–17 to –1)	Low[ad]	Whether there is an important difference or not in CIB is very uncertain
		High risk: 28[f]	–13 (–34 to –1)	Low[ad]	Less CIB with PPI. For PPI versus H2RA the desirable effects were small to moderate
		Highest risk: 41[f]	–18 (–50 to –3)	Low[ad]	
	RR 0.76 (0.49 to 1.09)	Low risk: 13[f]	–4 (–13 to 1)	Moderate[b]	PPIs probably reduce overt bleeding more than H2RAs
		Moderate risk: 38[f]	–12 (–39 to 3)	Moderate[b]	PPIs probably reduce overt bleeding more than H2RAs
		High risk: 63[f]	–20 (–65 to 5)	Moderate[b]	PPIs probably reduce overt bleeding more than H2RAs
		Highest risk: 95[f]	–29 (–98 to 8)	Moderate[b]	PPIs probably reduce overt bleeding more than H2RAs
**PPIs vs Sucralfate**	RR 0.56 (0.32 to 0.88)	Low risk: 6[f]	–4 (–8 to –1)	Moderate[b]	There is probably no important difference
		Moderate risk: 14[f]	–11 (–30 to –2)	Low[ab]	There may be no important difference
		High risk: 28[f]	–22 (–60 to –4)	Moderate[b]	PPIs probably reduce CIB compared with sucralfate desirable effects were moderate, favoring PPI
		Highest risk: 41[f]	–32 (–87 to –6)	Moderate[b]	PPIs probably reduce CIB compared with sucralfate
	RR 0.50 (0.27 to 0.84)	Low risk: 13[f]	–13 (–36 to –2)	Moderate[b]	PPIs probably reduce overt bleeding compared with sucralfate
		Moderate risk: 38[f]	–38 (–105 to –7)	Moderate[b]	PPIs probably reduce overt bleeding compared with sucralfate
		High risk: 63[f]	–63 (–174 to –11)	Moderate[b]	PPIs probably reduce overt bleeding compared with sucralfate
		Highest risk: 95[f]	–95 (–262 to –17)	Moderate[b]	PPIs probably reduce overt bleeding compared with sucralfate
**H2RAs vs Sucralfate**	RR 0.81 (0.55 to 1.20)	Low risk: 8[f]	–2 (–7 to 1)	Low[ad]	There may be no important difference
		Moderate risk: 20[f]	–5 (–16 to 3)	Very low[ad]	Whether there is an important difference or not is very uncertain
		High risk: 40[f]	–9 (–33 to 7)	Low[ad]	There may be no important difference/trivial for CIB
		Highest risk: 60[f]	–14 (–49 to 10)	Low[ad]	There may be no important difference/trivial for CIB
	RR 0.66 (0.41 to 1.02)	H2RAs: 17[f]	–9 (–25 to 0)	Moderate[b]	H2RAs probably reduce overt bleeding compared with sucralfate
		Moderate risk: 50[f]	–26 (–73 to 1)	Moderate[b]	H2RAs probably reduce overt bleeding compared with sucralfate
		High risk: 83[f]	–43 (–121 to 2)	Moderate[b]	H2RAs probably reduce overt bleeding compared with sucralfate
		Highest risk: 125[f]	–65 (–183 to 2)	Moderate[b]	H2RAs probably reduce overt bleeding compared with sucralfate

[a] Rated down due to uncertainty in baseline risk for some risk factors
[b] Rated down for imprecision
[c] Rated down for risk of bias
[d] Rated down for differences in results from different analyses/models and uncertainty in baseline risk
[e] Rated down 2 levels for imprecision
[f] Baseline risk based on event rates in comparator groups (PPIs or H2RAs)

GRADE Summary of Findings: ICU Length of Stay & Duration of Mechanical Ventilation (Complete PEPTIC Analysis)

Outcome	Comparison	Direct Estimate (95% CrI)	Certainty (Direct)	Indirect Estimate (95% CrI)	Certainty (Indirect)	Network Estimate (95% CrI)	Certainty (Network)	Baseline Risk (days)	Absolute Difference (95% CrI)
**ICU Length of Stay**	PPIs vs H2RAs	1.01 (0.74 to 1.35)	Moderate[a]	0.94 (0.76 to 1.14)	Moderate[b]	0.96 (0.85 to 1.08)	Moderate[b]	PPIs: 7.4†	–0.3 (–1.2 to 0.5)
	PPIs vs Sucralfate	1.01 (0.70 to 1.45)	High	0.94 (0.76 to 1.14)	Moderate[b]	1.01 (0.91 to 1.13)	Moderate[b]	PPIs: 7.4†	0.2 (–0.6 to 1.0)
	H2RAs vs Sucralfate	1.02 (0.91 to 1.16)	High	1.05 (0.88 to 1.32)	High	1.02 (0.93 to 1.14)	High	H2RAs: 7.4†	0.1 (–0.6 to 0.9)
**Duration of Mechanical Ventilation**	PPIs vs H2RAs	1.01 (0.83 to 1.22)	High	0.97 (0.86 to 1.11)	Moderate[b]	0.95 (0.84 to 1.08)	Moderate[b]	PPIs: 9.5†	–0.3 (–1.4 to 0.9)
	PPIs vs Sucralfate	1.02 (0.80 to 1.31)	High	1.00 (0.87 to 1.14)	Moderate[b]	0.97 (0.88 to 1.10)	Moderate[b]	PPIs: 9.5†	–0.3 (–1.4 to 0.9)
	H2RAs vs Sucralfate	0.95 (0.87 to 1.06)	Moderate[b]	0.97 (0.79 to 1.19)	Moderate[b]	0.95 (0.88 to 1.04)	Moderate[b]	H2RAs: 9.7†	–0.5 (–1.3 to 0.4)

[a] Rated down for risk of bias
[b] Rated down for imprecision
† Baseline risk comes from median event rate in the comparator group of the included studies:

- For comparisons vs **H2RAs or Sucralfate**, baseline is from the **PPIs** group
- For **H2RAs vs Sucralfate**, baseline is from the **H2RAs** group

GRADE Summary of Findings: desirable Effects Including IV/PO route & Dose-based comparison

Comparison	Outcome	Direct Estimate (95% Crl)	Node-splitting Estimate (95% Crl)	Certainty (Direct)	Indirect Estimate (95% Crl)	Certainty (Indirect)	NMA Estimate (95% Crl)	Certainty (NMA)
PPI PO vs H2RA PO/IV	CIGIB	0.09 (0.01, 0.68)	Not estimable	Moderate[a]	Not estimable	–	0.15 (0.02, 0.66)	Very low[b]
	Overt GIB	0.09 (0.01, 0.68)	Not estimable	Moderate[a]	Not estimable	–	0.15 (0.02, 0.66)	Very low[b]
Sucralfate vs PPI IV	CIGIB	3.13 (0.33, 29.37)	1.95 (0.28, 15.3)	High	1.41 (0.67, 3.05)	High	1.54 (0.77, 3.18)	Low[b]
	Overt GIB	3.13 (0.33, 29.37)	1.95 (0.28, 15.3)	High	1.41 (0.67, 3.05)	High	1.54 (0.77, 3.18)	Low[b]
Sucralfate vs H2RA IV	CIGIB	1.11 (0.68, 1.82)	0.95 (0.56, 1.51)	High	0.92 (0.06, 9.76)	High	1.01 (0.60, 1.56)	Moderate[a]
	Overt GIB	1.11 (0.68, 1.82)	0.95 (0.56, 1.51)	High	0.92 (0.06, 9.76)	High	1.01 (0.60, 1.56)	Moderate[a]
PPI High Dose vs H2RA Low Dose	CIGIB	0.22 (0.07, 0.73)	0.21 (0.05, 0.67)	Moderate	0.38 (0.09, 1.48)	Moderate	0.26 (0.10, 0.63)	Low
	Overt GIB	0.22 (0.07, 0.73)	0.21 (0.05, 0.67)	Moderate	0.38 (0.09, 1.48)	Moderate	0.26 (0.10, 0.63)	Low
PPI Low Dose vs H2RA Low Dose	CIGIB	0.94 (0.10, 8.86)	0.37 (0.03, 2.77)	Moderate	0.31 (0.11, 0.79)	Moderate	0.32 (0.12, 0.74)	Very low
	Overt GIB	0.94 (0.10, 8.86)	0.37 (0.03, 2.77)	Moderate	0.31 (0.11, 0.79)	Moderate	0.32 (0.12, 0.74)	Very low
H2RA High Dose vs PPI High Dose	CIGIB	1.40 (0.55, 3.61)	1.24 (0.37, 4.01)	Moderate	3.46 (0.91, 16.9)	Moderate	1.89 (0.81, 4.96)	Low
	Overt GIB	1.40 (0.55, 3.61)	1.24 (0.37, 4.01)	Moderate	3.45 (0.91, 16.9)	Moderate	1.89 (0.81, 4.96)	Low

[a] Rated down for serious imprecision in direct estimates (e.g., wide Crls, low event numbers)

[b] Rated down 2 levels for imprecision and/or incoherence in NMA estimates

Intervention	clinically important gastrointestinal bleeding (CIB) for highest risk population- complete PEPTIC analysis<br>I/S -- per 1000<br>S/M -- per 1000<br>M/L -- per 1000	CIB – High risk population - complete PEPTIC analysis<br>I/S -- per 1000<br>S/M -- per 1000<br>M/L -- per 1000	CIB- moderate risk population-complete PEPTIC analysis<br>I/S -- per 1000<br>S/M -- per 1000<br>M/L -- per 1000	Overt gastrointestinal bleeding- highest risk of bleeding<br>I/S -- per 1000<br>S/M -- per 1000<br>M/L -- per 1000	Overt gastrointestinal bleeding-High risk population<br>I/S -- per 1000<br>S/M -- per 1000<br>M/L -- per 1000	Overt gastrointestinal bleeding- moderate risk population<br>I/S -- per 1000<br>S/M -- per 1000<br>M/L -- per 1000	length of ICU stay-complete PEPTIC analysis<br>Difference -- or more<br>Difference -- or more<br>Difference -- or more	Duration of mechanical ventilation- complete PEPTIC analysis<br>Difference -- or more<br>Difference -- or more<br>Difference -- or more
Proton pump inhibitor vs histamine-2 receptor antagonists	NMA<br>18 fewer<br>50 fewer to 3 fewer<br>RR 0.69<br>(0.45, 0.93)<br>LOW	NMA<br>13 fewer<br>34 fewer to 2 fewer<br>RR 0.69<br>(0.45, 0.93)<br>LOW	NMA<br>6 fewer<br>17 fewer to 1 fewer<br>RR 0.69<br>(0.45, 0.93)<br>VERY LOW	NMA<br>29 fewer<br>98 fewer to 8 more<br>RR 0.76<br>(0.49, 1.09)<br>MODERATE	NMA<br>20 fewer<br>65 fewer to 5 more<br>RR 0.76<br>(0.49, 1.09)<br>MODERATE	NMA<br>12 fewer<br>39 fewer to 3 more<br>RR 0.76<br>(0.49, 1.09)<br>MODERATE	NMA<br>MD 1<br>(0.94, 1.07)<br>MODERATE	NMA<br>MD 1.02<br>(0.95, 1.12)<br>LOW
Proton pump inhibitor vs sucralfate	NMA<br>32 fewer<br>87 fewer to 6 fewer<br>RR 0.56<br>(0.32, 0.88)<br>MODERATE	NMA<br>22 fewer<br>60 fewer to 4 fewer<br>RR 0.56<br>(0.32, 0.88)<br>MODERATE	NMA<br>11 fewer<br>30 fewer to 2 fewer<br>RR 0.56<br>(0.32, 0.88)<br>MODERATE	NMA<br>95 fewer<br>262 fewer to 17 fewer<br>RR 0.5<br>(0.27, 0.84)<br>MODERATE	NMA<br>63 fewer<br>174 fewer to 11 fewer<br>RR 0.5<br>(0.27, 0.84)<br>MODERATE	NMA<br>38 fewer<br>105 fewer to 7 fewer<br>RR 0.5<br>(0.27, 0.84)<br>MODERATE	NMA<br>MD 1.03<br>(0.92, 1.16)<br>MODERATE	NMA<br>MD 0.97<br>(0.88, 1.1)<br>LOW
histamine-2 receptor antagonists vs Proton pump inhibitor	NMA<br>18 more<br>3 more to 50 more<br>RR 1.45<br>(1.08, 2.22)<br>LOW	NMA<br>13 more<br>2 more to 34 more<br>RR 1.45<br>(1.08, 2.22)<br>LOW	NMA<br>6 more<br>1 more to 17 more<br>RR 1.45<br>(1.08, 2.22)<br>VERY LOW	NMA<br>29 more<br>8 fewer to 98 more<br>RR 1.32<br>(0.92, 2.04)<br>MODERATE	NMA<br>20 more<br>5 fewer to 65 more<br>RR 1.32<br>(0.92, 2.04)<br>MODERATE	NMA<br>12 more<br>3 fewer to 39 more<br>RR 1.32<br>(0.92, 2.04)<br>MODERATE	NMA<br>MD -1<br>(-1.07, -0.94)<br>MODERATE	NMA<br>MD -1.02<br>(-1.12, -0.95)<br>LOW
histamine-2 receptor antagonists vs sucralfate	NMA<br>14 fewer<br>40 fewer to 10 more<br>RR 0.81<br>(0.55, 1.2)<br>LOW	NMA<br>9 fewer<br>33 fewer to 7 more<br>RR 0.81<br>(0.55, 1.2)<br>LOW	NMA<br>5 fewer<br>16 fewer to 3 more<br>RR 0.81<br>(0.55, 1.2)<br>VERY LOW	NMA<br>65 fewer<br>183 fewer to 2 more<br>RR 0.66<br>(0.41, 1.02)<br>MODERATE	NMA<br>43 fewer<br>121 fewer to 2 more<br>RR 0.66<br>(0.41, 1.02)<br>MODERATE	NMA<br>26 fewer<br>73 fewer to 1 more<br>RR 0.66<br>(0.41, 1.02)<br>MODERATE	NMA<br>MD 1.02<br>(0.93, 1.14)<br>HIGH	NMA<br>MD 0.97<br>(0.88, 1.1)
sucralfate vs Proton pump inhibitor	NMA<br>32 more<br>6 more to 87 more<br>RR 1.79<br>(1.14, 3.13)<br>MODERATE	NMA<br>22 more<br>4 more to 60 more<br>RR 1.79<br>(1.14, 3.13)<br>MODERATE	NMA<br>11 more<br>2 more to 30 more<br>RR 1.79<br>(1.14, 3.13)<br>MODERATE	NMA<br>95 more<br>17 more to 262 more<br>RR 2<br>(1.19, 3.7)<br>MODERATE	NMA<br>63 more<br>11 more to 174 more<br>RR 2<br>(1.19, 3.7)<br>MODERATE	NMA<br>38 more<br>7 more to 105 more<br>RR 2<br>(1.19, 3.7)<br>MODERATE	NMA<br>MD -1.03<br>(-1.16, -0.92)<br>MODERATE	NMA<br>MD -0.97<br>(-1.1, -0.88)<br>LOW
sucralfate vs histamine-2 receptor antagonists	NMA<br>14 more<br>10 fewer to 49 more<br>RR 1.23<br>(0.83, 1.82)<br>LOW	NMA<br>9 more<br>7 fewer to 33 more<br>RR 1.23<br>(0.83, 1.82)<br>LOW	NMA<br>5 more<br>3 fewer to 16 more<br>RR 1.23<br>(0.83, 1.82)<br>VERY LOW	NMA<br>65 more<br>2 fewer to 183 more<br>RR 1.52<br>(0.98, 2.44)<br>MODERATE	NMA<br>43 more<br>2 fewer to 121 more<br>RR 1.52<br>(0.98, 2.44)<br>MODERATE	NMA<br>26 more<br>1 fewer to 73 more<br>RR 1.52<br>(0.98, 2.44)<br>MODERATE	NMA<br>MD -1.02<br>(-1.14, -0.93)<br>HIGH	NMA<br>MD -0.97<br>(-1.1, -0.88)

NMA links:

1- Agents: https://gdt.gradepro.org/presentations/#/nma/nma_question_1f57d3a1-f4e6-43a3-9f1e-f7900d9566e3

2- Route: https://gdt.gradepro.org/presentations/#/nma/nma_question_8d3fd775-9e1b-4580-8660-da9bf29d1f04

3- Dose: https://gdt.gradepro.org/presentations/#/nma/nma_question_0bd7e2d0-9cbf-4cf1-ada2-ebf3d232498d

Undesirable Effects

How substantial are the undesirable anticipated effects?

JUDGEMENT	RESEARCH EVIDENCE	ADDITIONAL CONSIDERATIONS

JUDGEMENT

Trivial: PPIs Low Dose vs. H2RAs Low Dose, H2RAs vs. Sucralfate, PPIs vs. H2RAs, PPIs High Dose vs. H2RAs Low Dose, H2RAs High Dose vs. PPIs High Dose, PPIs PO vs. H2RAs PO/IV

Small: Sucralfate vs. H2RAs IV, Sucralfate vs. PPIs IV, PPIs vs. Sucralfate

Moderate:

Large:

Varies:

Don't know:

RESEARCH EVIDENCE

How substantial are the undesirable anticipated effects?

	Desirable effect: Clinically Important Gastrointestinal Bleeding (CIGIB)	Desirable effect: Overt bleeding	Desirable effect: Length of ICU stay	Desirable effect: duration of mechanical ventilation	Undesirable effect: mortality	Undesirable effect: pneumonia	Undesirable effect: Clostroides difficile infection	Overall
PPI vs. H2RA	–	–	–	–	TRIVIAL	TRIVIAL	TRIVIAL	TRIVIAL
PPI vs. Sucralfate	–	–	–	–	SMALL	SMALL	DON'T KNOW	SMALL
H2RAs vs Sucralfate	–	–	–	–	TRIVIAL	SMALL	DON'T KNOW	TRIVIAL
PPI PO vs H2RA PO/IV	–	–	–	–	TRIVIAL	TRIVIAL	DON'T KNOW	TRIVIAL
Sucralfate vs PPI IV	–	–	–	–	SMALL	SMALL	DON'T KNOW	SMALL
Sucralfate vs H2RA IV	–	–	–	–	SMALL	SMALL	DON'T KNOW	SMALL
PPI High Dose vs H2RA Low Dose	–	–	–	–	TRIVIAL	TRIVIAL	DON'T KNOW	TRIVIAL
PPI Low Dose vs H2RA Low Dose	–	–	–	–	TRIVIAL	TRIVIAL	DON'T KNOW	TRIVIAL
H2RA High Dose vs PPI High Dose	–	–	–	–	TRIVIAL	TRIVIAL	DON'T KNOW	TRIVIAL

Mortality

- There is **probably no important difference in mortality** between **PPIs and H2RAs** (RR 1.05, 95% CI: 0.97 to 1.14; low certainty). However, the **95% credible interval** does **not exclude the possibility of harm** with PPIs.
- **Subgroup analyses** from the **PEPTIC** and **SUP-ICU** trials suggest a **potential increase in mortality among sicker patients** (e.g., APACHE II score ≥24) receiving **PPIs** compared to H2RAs or placebo, but the **credibility of this effect is low** (low certainty).
- **Network meta-regression** did not show that risk of bias affected mortality results.

	• Overall, **current evidence supports that PPIs and H2RAs likely have little or no effect on mortality**, but further RCTs are needed to clarify potential subgroup effects.<br><br>Pneumonia<br>• There is **no important difference** in pneumonia incidence between **PPIs and H2RAs** (low certainty).<br>• **Sucralfate** may be associated with **lower pneumonia risk** compared to **PPIs** and **H2RAs**, but the **undesirable effects are small**.<br><br>*Clostridium difficile* Infection<br>• **No important difference** was found in the risk of ***C. difficile*** **infection** between **PPIs** and **H2RAs**.<br>• The **absolute effect is small**, likely due to the **low baseline risk** of infection.<br><br>Route of Administration & Dosage Comparisons<br>• **PPIs (oral)** vs. **H2RAs (IV)**: Undesirable effects were **trivial**.<br>• **Sucralfate** vs. **PPIs (IV)** and **H2RAs (IV)**: Undesirable effects were **small**, with a possible reduction in pneumonia.<br>• **PPI high dose vs. H2RA low dose**, **PPI low dose vs. H2RA low dose**, and **H2RA high dose vs. PPI high dose**: All showed **trivial undesirable effects** with **no significant differences in mortality or pneumonia**.<br><br>**Evidence profiles** (4, 3, 2)	

GRADE Summary of Findings: Undesirable Effects (Complete PEPTIC Analysis)

Outcome	Comparison	Direct Estimate (95% CrI)	Certainty (Direct)	Indirect Estimate (95% CrI)	Certainty (Indirect)	Network Estimate (95% CrI)	Certainty (Network)	Baseline Risk (/1000)	Absolute Difference (95% CrI)	Plain text summary
**Mortality**	PPIs vs H2RAs	1.06 (0.95 to 1.18)	Moderate[d]	1.03 (0.86 to 1.23)	High	1.05 (0.97 to 1.14)	Low[fg]	PPIs: 313[h]	15 (–10 to 38)	PPI versus H2RA the undesirable effects were trivial
	PPIs vs Sucralfate	0.99 (0.61 to 1.61)	High	1.13 (0.97 to 1.32)	Moderate[d]	1.11 (0.96 to 1.28)	Low[fg]	PPIs: 313[h]	31 (–13 to 68)	undesirable effects were small
	H2RAs vs Sucralfate	1.08 (0.94 to 1.26)	High	0.99 (0.75 to 1.32)	High	1.05 (0.93 to 1.20)	Low[fg]	H2RAs: 298[h]	14 (–22 to 50)	H2RA versus Sucralfate the undesirable effects were trivial to small.
**Pneumonia**	PPIs vs H2RAs	1.07 (0.76 to 1.52)	High	0.97 (0.68 to 1.35)	Moderate[d]	1.02 (0.80 to 1.33)	Low[de]	PPIs: 175[h]	3 (–44 to 43)	PPI versus H2RA the undesirable effects were trivial
	PPIs vs Sucralfate	1.28 (0.47 to 3.48)	High	1.16 (0.77 to 1.80)	High	1.17 (0.81 to 1.74)	Moderate[e]	PPIs: 175[h]	25 (–24 to 74)	Less pneumonia with sucralfate undesirable effects were small
	H2RAs vs Sucralfate	1.17 (0.78 to 1.74)	High	1.03 (0.45 to 2.34)	High	1.15 (0.83 to 1.57)	Low[e]	H2RAs: 173[h]	23 (–35 to 63)	Less pneumonia with sucralfate H2RA versus Sucralfate the undesirable effects were trial to small.
**C. difficile Infection**	PPIs vs H2RAs	0.76 (0.29 to 2.15)	Moderate[d]	NA	–	0.76 (0.28 to 2.16)	Moderate[d]	PPIs: 12[h]	–4 (–31 to 6)	

[a] Imprecision not considered for direct/indirect estimates, used only to inform network estimates

[b] Network estimate rating based on higher of direct/indirect certainty, considering imprecision and incoherence

[c] Baseline risk from placebo group in SUP-ICU trial

[d] Rated down for risk of bias

[e] Rated down for imprecision

[f] Rated down 2 levels for imprecision

[g] Rated down for inconsistency between models/analyses

[h] Baseline risk derived from event rates in PPI or H2RA comparator group

GRADE Summary of Findings: Undesirable Effects Including IV/PO route & Dose-based comparison

Comparison	Outcome	Direct Estimate (95% CrI)	Node-splitting Estimate (95% CrI)	Certainty (Direct)	Indirect Estimate (95% CrI)	Certainty (Indirect)	NMA Estimate (95% CrI)	Certainty (NMA)
**PPI PO vs H2RA PO/IV**	Mortality	0.11 (0.01, 2.03)	Not estimable	Moderate[a]	Not estimable	–	0.19 (0.02, 1.25)	Very low[b]
	Pneumonia	0.23 (0.05, 1.17)	Not estimable	Moderate[a]	Not estimable	–	0.30 (0.06, 1.38)	Very low[b]
**Sucralfate vs PPI IV**	Mortality	1.32 (0.62, 2.82)	1.26 (0.72, 2.24)	Moderate[a]	0.81 (0.63, 1.03)	High	0.87 (0.70, 1.07)	Low[b]
	Pneumonia	0.52 (0.23, 1.20)	0.47 (0.22, 0.95)	Moderate[a]	0.79 (0.53, 1.10)	High	0.72 (0.51, 0.97)	Low[b]
**Sucralfate vs H2RA IV**	Mortality	0.96 (0.83, 1.11)	0.96 (0.82, 1.12)	High	0.65 (0.21, 1.79)	High	0.97 (0.83, 1.13)	Moderate[a]
	Pneumonia	0.83 (0.71, 0.96)	0.77 (0.50, 0.94)	High	1.63 (0.09, 31.0)	High	0.78 (0.63, 0.95)	High
**PPI High Dose vs H2RA Low Dose**	Mortality	0.96 (0.77, 1.20)	0.95 (0.74, 1.21)	Moderate	1.82 (1.18, 2.83)	Moderate	1.10 (0.89, 1.38)	Low
	Pneumonia	1.20 (0.73, 1.98)	1.16 (0.65, 2.09)	Moderate	1.40 (0.65, 2.63)	Moderate	1.28 (0.85, 1.94)	Low
**PPI Low Dose vs H2RA Low Dose**	Mortality	2.00 (0.52, 7.63)	1.76 (0.51, 7.01)	Moderate	1.19 (0.89, 1.58)	Moderate	1.21 (0.92, 1.62)	Very low
	Pneumonia	0.33 (0.14, 0.78)	0.33 (0.12, 0.80)	Moderate	0.87 (0.55, 1.29)	Moderate	0.71 (0.45, 1.04)	Very low
**H2RA High Dose vs PPI High Dose**	Mortality	1.22 (0.62, 2.41)	1.26 (0.85, 1.87)	Moderate	1.22 (0.89, 1.67)	Moderate	1.05 (0.81, 1.38)	Low
	Pneumonia	0.96 (0.61, 1.50)	0.89 (0.52, 1.47)	Moderate	0.82 (0.44, 1.49)	Moderate	0.90 (0.58, 1.34)	Low

[a] Rated down for serious imprecision in direct estimates (e.g., wide CrIs, low event numbers)
[b] Rated down 2 levels for imprecision and/or incoherence in NMA estimates

Intervention	mortality—complete PEPTIC analysis<br>T/S 12 per 1000<br>S/M 30 per 1000<br>M/L 80 per 1000	Pneumonia<br>T/S 175 per 1000<br>S/M -- per 1000<br>M/L -- per 1000	Clostroides difficile infection – complete PEPTIC analysis<br>T/S -- per 1000<br>S/M -- per 1000<br>M/L -- per 1000
Proton pump inhibitor vs histamine-2 receptor antagonists	NMA<br>**15 more**<br>10 fewer to 38 more<br>**RR 1.05**<br>(0.97, 1.14)<br>LOW	NMA<br>**3 more**<br>44 fewer to 43 more<br>**RR 1.02**<br>(0.8, 1.33)<br>LOW	NMA<br>**4 fewer**<br>31 fewer to 6 more<br>**RR 0.76**<br>(0.28, 2.16)<br>LOW
Proton pump inhibitor vs sucralfate	NMA<br>**31 more**<br>13 fewer to 68 more<br>**RR 1.11**<br>(0.96, 1.28)<br>LOW	NMA<br>**25 more**<br>41 fewer to 74 more<br>**RR 1.17**<br>(0.81, 1.74)<br>LOW	No data
Proton pump inhibitor vs placebo	NMA<br>**9 more**<br>21 fewer to 43 more<br>**RR 1.03**<br>(0.93, 1.14)<br>MODERATE	NMA<br>**13 more**<br>19 fewer to 73 more<br>**RR 1.08**<br>(0.88, 1.45)<br>LOW	NMA<br>**3 fewer**<br>10 fewer to 18 more<br>**RR 0.82**<br>(0.34, 2.21)<br>MODERATE

Intervention	mortality—complete PEPTIC analysis<br>T/S 12 per 1000<br>S/M 30 per 1000<br>M/L 80 per 1000	Pneumonia<br>T/S 175 per 1000<br>S/M -- per 1000<br>M/L -- per 1000	Clostroides difficile infection – complete PEPTIC analysis<br>T/S -- per 1000<br>S/M -- per 1000<br>M/L -- per 1000
sucralfate vs Proton pump inhibitor	NMA<br>**31 fewer**<br>68 fewer to 13 more<br>**RR 0.9**<br>(0.78, 1.04)<br>LOW	NMA<br>**25 fewer**<br>74 fewer to 41 more<br>**RR 0.85**<br>(0.57, 1.23)<br>LOW	No data
sucralfate vs histamine-2 receptor antagonists	NMA<br>**14 fewer**<br>50 fewer to 22 more<br>**RR 0.95**<br>(0.83, 1.08)<br>LOW	NMA<br>**23 fewer**<br>63 fewer to 35 more<br>**RR 0.87**<br>(0.64, 1.2)<br>LOW	No data
sucralfate vs placebo	NMA<br>**21 fewer**<br>61 fewer to 21 more<br>**RR 0.93**<br>(0.8, 1.07)<br>VERY LOW	No data	No data

NMA links:

1- Agents: https://gdt.gradepro.org/presentations/#/nma/nma_question_1f57d3a1-f4e6-43a3-9f1e-f7900d9566e3

2- Route: https://gdt.gradepro.org/presentations/#/nma/nma_question_8d3fd775-9e1b-4580-8660-da9bf29d1f04

3- Dose: https://gdt.gradepro.org/presentations/#/nma/nma_question_0bd7e2d0-9cbf-4cf1-ada2-ebf3d232498d

Net balance

Net Balance

JUDGEMENT	RESEARCH EVIDENCE	ADDITIONAL CONSIDERATIONS
**Large net desirable:**<br>**Moderate net desirable:** H2RAs vs. Sucralfate, PPIs vs. H2RAs, PPIs vs. Sucralfate<br>**Small net desirable:**<br>**Null net balance:** PPIs Low Dose vs. H2RAs Low Dose, Sucralfate vs. H2RAs IV, PPIs High Dose vs. H2RAs Low Dose, H2RAs High Dose vs. PPIs High Dose, Sucralfate vs. PPIs IV, PPIs PO vs. H2RAs PO/IV<br>**Small net undesirable:**<br>**Moderate net undesirable:**<br>**Large net undesirable:**	See table below	

	Desirable Effects	Undesirable Effects	Overall
PPI vs. H2RA	Small	Trivial	MODERATE NET DESIRABLE
PPI vs. Sucralfate	Moderate	Small	MODERATE NET DESIRABLE
H2RAs vs Sucralfate	Trivial	Trivial	MODERATE NET DESIRABLE
PPI PO vs H2RA PO/IV	Moderate	Trivial	NULL NET BALANCE
Sucralfate vs PPI IV	Trivial	Small	NULL NET BALANCE
Sucralfate vs H2RA IV	Trivial	Small	NULL NET BALANCE
PPI High Dose vs H2RA Low Dose	Moderate	Trivial	NULL NET BALANCE
PPI Low Dose vs H2RA Low Dose	Moderate	Trivial	NULL NET BALANCE
H2RA High Dose vs PPI High Dose	Trivial	Trivial	NULL NET BALANCE

Certainty of evidence

What is the overall certainty of the evidence of effects?

JUDGEMENT	RESEARCH EVIDENCE	ADDITIONAL CONSIDERATIONS
**Very low:** PPIs Low Dose vs. H2RAs Low Dose, PPIs PO vs. H2RAs PO/IV<br>**Low:** Sucralfate vs. H2RAs IV, PPIs High Dose vs. H2RAs Low Dose, H2RAs High Dose vs. PPIs High Dose, Sucralfate vs. PPIs IV<br>**Moderate:** H2RAs vs. Sucralfate, PPIs vs. H2RAs, PPIs vs. Sucralfate<br>**High:**<br>**No included studies:**	See table below	

Judgement

What is the overall certainty of the evidence of effects?

	Desirable effect: Clinically Important Gastrointestinal Bleeding (CIGIB)	Desirable effect: Overt bleeding	Desirable effect: Length of ICU stay	Desirable effect: duration of mechanical ventilation	Undesirable effect: mortality	Undesirable effect: pneumonia	Undesirable effect: Clostroides difficile infection	Overall
PPI vs. H2RA	LOW	MODERATE	MODERATE	MODERATE	LOW	LOW	MODERATE	MODERATE
PPI vs. Sucralfate	MODERATE	MODERATE	MODERATE	MODERATE	LOW	MODERATE	NO INCLUDED STUDIES	MODERATE
H2RAs vs Sucralfate	LOW	MODERATE	HIGH	MODERATE	LOW	LOW	NO INCLUDED STUDIES	MODERATE
PPI PO vs H2RA PO/IV	VERY LOW	VERY LOW	NO INCLUDED STUDIES	NO INCLUDED STUDIES	VERY LOW	VERY LOW	NO INCLUDED STUDIES	VERY LOW
Sucralfate vs PPI IV	LOW	LOW	NO INCLUDED STUDIES	NO INCLUDED STUDIES	LOW	LOW	NO INCLUDED STUDIES	LOW
Sucralfate vs H2RA IV	MODERATE	MODERATE	NO INCLUDED STUDIES	NO INCLUDED STUDIES	MODERATE	HIGH	NO INCLUDED STUDIES	LOW
PPI High Dose vs H2RA Low Dose	LOW	LOW	NO INCLUDED STUDIES	NO INCLUDED STUDIES	LOW	LOW	NO INCLUDED STUDIES	LOW
PPI Low Dose vs H2RA Low Dose	VERY LOW	VERY LOW	NO INCLUDED STUDIES	NO INCLUDED STUDIES	VERY LOW	VERY LOW	NO INCLUDED STUDIES	VERY LOW
H2RA High Dose vs PPI High Dose	LOW	LOW	NO INCLUDED STUDIES	NO INCLUDED STUDIES	LOW	LOW	NO INCLUDED STUDIES	LOW

Values

Is there important uncertainty about or variability in how much people value the main outcomes?

JUDGEMENT	RESEARCH EVIDENCE	ADDITIONAL CONSIDERATIONS
**Important uncertainty or variability: Possibly important**		

uncertainty or variability: PPIs Low Dose vs. H2RAs Low Dose, PPIs vs. H2RAs, PPIs High Dose vs. H2RAs Low Dose, H2RAs High Dose vs. PPIs High Dose, PPIs PO vs. H2RAs PO/IV

Probably no important uncertainty or variability: H2RAs vs. Sucralfate, Sucralfate vs. H2RAs IV, Sucralfate vs. PPIs IV, PPIs vs. Sucralfate

No important uncertainty or variability:

	Net balance	Certainty of evidence	Overall
PPI vs. H2RA	Moderate net desirable	Moderate	POSSIBLY IMPORTANT UNCERTAINTY OR VARIABILITY
PPI vs. Sucralfate	Moderate net desirable	Moderate	PROBABLY NO IMPORTANT UNCERTAINTY OR VARIABILITY
H2RAs vs Sucralfate	Moderate net desirable	Low	PROBABLY NO IMPORTANT UNCERTAINTY OR VARIABILITY
PPI PO vs H2RA PO/IV	Null net balance	Very low	POSSIBLY IMPORTANT UNCERTAINTY OR VARIABILITY
Sucralfate vs PPI IV	Null net balance	Low	PROBABLY NO IMPORTANT UNCERTAINTY OR VARIABILITY
Sucralfate vs H2RA IV	Null net balance	Moderate	PROBABLY NO IMPORTANT UNCERTAINTY OR VARIABILITY
PPI High Dose vs H2RA Low Dose	Null net balance	Low	POSSIBLY IMPORTANT UNCERTAINTY OR VARIABILITY
PPI Low Dose vs H2RA Low Dose	Null net balance	Very low	POSSIBLY IMPORTANT UNCERTAINTY OR VARIABILITY
H2RA High Dose vs PPI High Dose	Null net balance	Low	POSSIBLY IMPORTANT UNCERTAINTY OR VARIABILITY

Balance of effects

Does the balance between desirable and undesirable effects favor the intervention or the comparison?

JUDGEMENT	RESEARCH EVIDENCE	ADDITIONAL CONSIDERATIONS
**Favors the comparison:**<br>**Probably favors the comparison:**<br>**Does not favor either the intervention or the comparison:** PPIs vs. H2RAs,<br>**Probably favors the intervention:** H2RAs vs. Sucralfate, PPIs vs. Sucralfate<br>**Favors the intervention:**<br>**Varies:** PPIs PO vs. H2RAs PO/IV<br>**Don't know:** PPIs Low Dose vs. H2RAs Low Dose, Sucralfate vs. H2RAs IV, PPIs High Dose vs. H2RAs Low Dose, H2RAs High Dose vs. PPIs High Dose, Sucralfate vs. PPIs IV	See table below	

Does the balance between desirable and undesirable effects favor the intervention or the comparison?

	Desirable effect: Clinically Important Gastrointestinal Bleeding (CIGIB)	Desirable effect: Overt bleeding	Desirable effect: Length of ICU stay	Desirable effect: duration of mechanical ventilation	Undesirable effect: mortality	Undesirable effect: pneumonia	Undesirable effect: Clostroides difficile infection	Overall
PPI vs. H2RA								DOES NOT FAVOR EITHER THE INT
PPI vs. Sucralfate								PROBABLY FAVORS THE INTERVE
H2RAs vs Sucralfate								PROBABLY FAVORS THE INTERVE
PPI PO vs H2RA PO/IV								VARIES
Sucralfate vs PPI IV								DON'T KNOW
Sucralfate vs H2RA IV								DON'T KNOW
PPI High Dose vs H2RA Low Dose								DON'T KNOW
PPI Low Dose vs H2RA Low Dose								DON'T KNOW
H2RA High Dose vs PPI High Dose								DON'T KNOW

Resources required

JUDGEMENT	RESEARCH EVIDENCE	ADDITIONAL CONSIDERATIONS
**Large costs:**<br>**Moderate costs:**<br>**Negligible costs and savings:** H2RAs vs. Sucralfate, PPIs vs. Sucralfate<br>**Moderate savings:**<br>**Large savings:**<br>**Varies:** PPIs Low Dose vs. H2RAs Low Dose, Sucralfate vs. H2RAs IV, PPIs vs. H2RAs, PPIs High Dose vs. H2RAs Low Dose, H2RAs High Dose vs. PPIs High Dose, Sucralfate vs. PPIs IV, PPIs PO vs. H2RAs PO/IV<br>**Don't know:**	(3) (see table below)	

Category	Proton Pump Inhibitors (PPIs)	Histamine-2 Receptor Antagonists (H2RAs)	Sucralfate
**Route of Administration**	IV or enteral	IV or enteral	Enteral only
**Dosing Frequency**	Once per day	Two or three times per day	Four times per day
**Medication Routine**	Administered once daily	Administered two to three times daily	Administered four times daily
**Values and Preferences**	Most or all patients are likely to prefer an effective gastric acid suppressant	Same as PPIs	Same as PPIs
**Costs**	Intravenous forms usually more expensive than enteral forms; costs vary by agent	Intravenous forms usually more expensive than enteral forms; costs vary by agent	Enteral only; cost varies
**Adverse Effects, Interactions**	- PPIs are **metabolized by CYP450**, may interfere with drug absorption due to altered gastric pH<br>- Likely interactions: **clopidogrel, HIV protease inhibitors, methotrexate, magnesium**	- Cimetidine is a CYP450 inhibitor (rarely used)<br>- **Ranitidine and famotidine** have minimal CYP effects<br>- Less impact on pH-dependent absorption than PPIs	- Minimal systemic absorption<br>- Few drug interactions
**Side Effects**	- Serious side effects are **extremely rare**<br>- No widely known common side effects	- Serious side effects are **extremely rare**<br>- No widely known common side effects	- Side effects minimal due to local gut action
**Crushing for Feeding Tubes**	Tablets can be crushed for administration via feeding tube	Tablets can be crushed for administration via feeding tube	Tablets/suspensions can be given via feeding tube
**Cost & Access**	- Both IV and enteral forms available<br>- IV is more expensive than enteral	- Both IV and enteral forms available<br>- IV is more expensive than enteral	- Enteral only; generally inexpensive

**Certainty of evidence of required resources**<br>What is the certainty of the evidence of resource requirements (costs)?		
JUDGEMENT	RESEARCH EVIDENCE	ADDITIONAL CONSIDERATIONS
**Very low:**<br>**Low:**<br>**Moderate:**<br>**High:**<br>**No included studies:** PPIs Low Dose vs. H2RAs Low Dose, H2RAs vs. Sucralfate, Sucralfate vs. H2RAs IV, PPIs vs. H2RAs, PPIs High Dose vs. H2RAs Low Dose, H2RAs High Dose vs. PPIs High Dose, Sucralfate vs. PPIs IV, PPIs vs. Sucralfate, PPIs PO vs. H2RAs PO/IV	**No included studies**	

Cost effectiveness

Does the cost-effectiveness of the intervention favor the intervention or the comparison?

JUDGEMENT	RESEARCH EVIDENCE	ADDITIONAL CONSIDERATIONS
**Favors the comparison: Probably favors the comparison: Does not favor either the intervention or the comparison: Probably favors the intervention: Favors the intervention: Varies:** PPIs Low Dose vs. H2RAs Low Dose, H2RAs vs. Sucralfate, Sucralfate vs. H2RAs IV, PPIs vs. H2RAs, PPIs High Dose vs. H2RAs Low Dose, H2RAs High Dose vs. PPIs High Dose, Sucralfate vs. PPIs IV, PPIs vs. Sucralfate, PPIs PO vs. H2RAs PO/IV	**SUP-ICU Trial Findings** (5): Cost analyses from the SUP-ICU trial demonstrated that although pantoprazole reduced clinically important gastrointestinal (GI) bleeding in patients in the intensive care unit (ICU), this did not translate into economic or long-term health benefits.<br><br>• **Healthcare Costs:** Total healthcare expenditures were slightly higher in the pantoprazole group.<br>• **Resource Utilization:** No significant differences were found in hospital length of stay, primary care visits, or psychiatric services.<br>• **Employment and Mortality Outcomes:** Pantoprazole use had no effect on employment status, salary levels, or mortality at 90 days or one year.<br><br>These findings suggest that routine use of pantoprazole for stress ulcer prophylaxis may not be justified, especially in patients with ICU at lower risk of GI bleeding, due to the lack of cost savings and absence of long-term health improvements.<br>Indirect Evidence in Non-ICU Settings: A retrospective study by Tan et al. (2016) evaluated the continuation of SUP following ICU discharge and its financial consequences.<br><br>• **Inappropriate Continuation:** A substantial proportion of patients remained on SUP post-ICU despite lacking valid medical indications, highlighting a gap in medication reconciliation.<br>• **Increased Costs:** This inappropriate continuation led to significant, avoidable healthcare expenses. These findings highlight the need for careful re-evaluation of SUP prescriptions during care transitions to prevent unnecessary medication use and its associated costs.<br><br>**Direct Costs in ICU-Related GI Bleeding:**<br><br>• **Europe:** The average cost of managing major GI bleeding events ranges from $11,941 to $13,093, with hospitalization costs forming the bulk.<br>• **Saudi Arabia:** Although specific data on GI bleeding are limited, ICU admissions for severe conditions can cost up to SAR 79,418.30 (approx. USD 21,178) per patient.	**Panel comments**<br>Regional Formulary Context:<br>• **Saudi Arabia:**<br>• Ranitidine is withdrawn due to nitrosamine contamination concerns.<br>• Cimetidine is outdated and rarely used; nizatidine is not widely available.<br>• PPIs are widely available, low-cost due to generics, and easier to manage from a formulary perspective.<br>• The panel agreed that PPIs are the most feasible and cost-effective option under current availability.<br>• **Scandinavia:**<br>• H2RAs are no longer available in Finland and possibly other Scandinavian countries.<br>• This limits comparative cost-effectiveness,

**No included studies:**	**Four pharmacoeconomic evaluations have been conducted, each employing distinct decision-tree models with varying assumptions, variables, and measures of effectiveness.** (2)<br><br>1. **Udeh et al.** This analysis assessed cost per clinically significant bleeding event avoided, comparing proton pump inhibitors (PPIs), histamine-2 receptor antagonists (H2RAs), and sucralfate. The model incorporated drug acquisition costs, consumables, labor, costs related to managing bleeding, and evaluation of adverse drug events (ADEs), including diarrhea, thrombocytopenia, and altered mental status. Enteral omeprazole was the most cost-effective, with a cost-effectiveness ratio (CER) of $12,391 per bleeding event avoided. For enteral famotidine and sucralfate, CERs were $14,752 and $37,881, respectively.<br>2. **Barkun et al.** This study compared the cost per complication avoided between PPIs and H2RAs, considering both gastrointestinal bleeding and pneumonia as complications. The cost-effectiveness ratio favored PPIs, with a CER of $58,699 compared to $63,921 for H2RAs.<br>3. **MacLaren et al.** Focusing on mortality as the primary effectiveness outcome, this analysis compared PPIs and H2RAs while accounting for associated rates of GI bleeding, pneumonia, and *Clostridioides difficile* infection (CDI). The total cost per patient was $7,802 for PPIs and $6,707 for H2RAs. Mortality rates were identical (3.8%) in both groups, resulting in a cost saving of $1,095 per patient with H2RA therapy.<br>4. **Hammond et al.** This evaluation used both mortality and overall complication rates (clinically important bleeding, pneumonia, and CDI) to assess cost-effectiveness. Results showed higher costs and complication rates with PPIs: $11,249 cost, 22% complication rate, and 3.34% mortality. In contrast, H2RA therapy was associated with lower costs ($9,039), fewer complications (17.6%), and reduced mortality (2.5%), favoring H2RAs overall.	with PPIs being the default agent.<br>Overall, resource use and cost-effectiveness vary, but current market availability, simplicity of dosing, and broad generic access make PPIs the most practical option in many settings.<br><br>**Panel consensus**<br>Supports highlighting regional availability and formulary realities in the final recommendation remarks.

Equity
What would be the impact on health equity?

JUDGEMENT	RESEARCH EVIDENCE	ADDITIONAL CONSIDERATIONS
**Reduced: Probably reduced: Probably no impact:** PPIs Low Dose vs. H2RAs Low Dose, H2RAs vs. Sucralfate, Sucralfate vs. H2RAs IV, PPIs vs. H2RAs, PPIs	Specific agent availability varies by region and healthcare system. In both Saudi Arabia and Scandinavian countries, most commonly used agents—such as PPIs and H2RAs—are widely accessible. However, older or less frequently used agents may have limited availability.<br><br>• **Cimetidine**, **Famotidine**, **Ranitidine**, **Lansoprazole**, **Omeprazole**, **Pantoprazole**, **Esomeprazole**, **and Rabeprazole**<br>• **Ranitidine** has been withdrawn from many global markets—including the U.S., EU, and parts of the Middle East and Asia—because of contamination concerns with N-nitrosodimethylamine (NDMA), a probable human carcinogen.<br>• **Sucralfate** is still available but used infrequently in current ICU practice in both settings.<br><br>A comprehensive network meta-analysis of 123 RCTs (6) involving 46,996 patients in the ICU assessed 15 pharmacological interventions for stress ulcer prophylaxis. Compared with placebo, several agents likely reduced the risk of clinically important upper GI bleeding with **moderate certainty**: cimetidine (RR 0.56, 95% CI 0.40–0.77), ranitidine (RR 0.54, 95% CI 0.38–0.76), antacids (RR 0.48, 95% CI 0.33–0.68),	**Panel discussion**<br>• The panel agreed that while SUP agents are generally available, supply chain instability and regional differences may affect equitable access.<br>• Although no direct equity data were available, the

High Dose vs. H2RAs Low Dose, H2RAs High Dose vs. PPIs High Dose, Sucralfate vs. PPIs IV, PPIs vs. Sucralfate, PPIs PO vs. H2RAs PO/IV<br>**Probably increased:**<br>**Increased:**<br>**Varies:**<br>**Don't know:**	sucralfate (RR 0.54, 95% CI 0.39–0.75), and ranitidine + antacids (RR 0.13, 95% CI 0.03–0.62). Some PPIs showed potential benefit but with **low to very low certainty of evidence**: pantoprazole (RR 0.73, 95% CI 0.36–1.47), omeprazole (RR 0.33, 95% CI 0.20–0.56), lansoprazole (RR 0.10, 95% CI 0.01–0.94), esomeprazole (RR 0.19, 95% CI 0.06–0.63), and rabeprazole (RR 0.06, 95% CI 0.00–1.22). No agent showed clear benefit for secondary outcomes including pneumonia, mortality, or ICU length of stay. Evidence for these outcomes was generally low or very low, with no clear differences in adverse events or *C. difficile* risk.<br><br>The interventions assessed are **generally available globally**, including in **high-income settings**. However, older and less commonly used agents like **sucralfate** or **antacids** may not be routinely stocked in all ICUs. This may affect **accessibility and implementation**, especially in settings where **modern SUP practices favor PPIs**. Therefore, while no direct equity data were assessed, **formulation and regional availability may influence access** and thus equity.	formulation-specific and regional availability differences may influence access and implementation.<br>• The panel judged that SUP interventions likely have no major impact on equity, though 3 members selected "Don't know," reflecting ongoing access uncertainty.

Acceptability

Is the intervention acceptable to key interest-holders?

JUDGEMENT	RESEARCH EVIDENCE	ADDITIONAL CONSIDERATIONS
**No:**<br>**Probably no:**<br>**Probably yes:**<br>PPIs Low Dose vs. H2RAs Low Dose, H2RAs vs. Sucralfate, Sucralfate vs. H2RAs IV, PPIs vs. H2RAs, PPIs High Dose vs. H2RAs Low Dose, H2RAs High Dose vs. PPIs High Dose, Sucralfate vs. PPIs IV, PPIs vs.	Most pharmacological agents for stress ulcer prophylaxis are widely familiar to clinicians and are generally acceptable in ICU settings. PPIs and H2RAs are routinely used and are easy to administer via oral or IV routes, which supports high acceptability among providers. However, the acceptability of older agents like **sucralfate** or **ranitidine** may be lower due to reduced familiarity, limited availability, or safety concerns—particularly given **ranitidine's global market withdrawal** due to NDMA contamination. In regions where specific agents are unavailable or discontinued, clinical preferences may shift toward alternatives with more favorable safety profiles or regulatory standing.	**Panel comments**<br><br>• Most SUP agents, particularly PPIs and H2RAs, are familiar and acceptable to ICU clinicians, given their frequent use, easy administration (oral or IV), and strong presence in formularies.<br>• Older agents like sucralfate and ranitidine have lower acceptability due to:<br>• Limited familiarity

Sucralfate, PPIs PO vs. H2RAs PO/IV<br>**Yes:**<br>**Varies:**<br>**Don't know:**		• Discontinued status (ranitidine)<br>• Dosing inconvenience (e.g., sucralfate requires q6h dosing)

Feasibility
Is the intervention feasible to implement?

JUDGEMENT	RESEARCH EVIDENCE	ADDITIONAL CONSIDERATIONS
**No:**<br>**Probably no:**<br>**Probably yes:** PPIs Low Dose vs. H2RAs Low Dose, H2RAs vs. Sucralfate, Sucralfate vs. H2RAs IV, PPIs vs. H2RAs, PPIs High Dose vs. H2RAs Low Dose, H2RAs High Dose vs. PPIs High Dose, Sucralfate vs. PPIs IV, PPIs vs. Sucralfate, PPIs PO vs. H2RAs PO/IV<br>**Yes:**<br>**Varies:**<br>**Don't know:**	SUP agents like PPIs and H2RAs are feasible to use in most ICU settings. A large multicenter trial in Chinese ICUs demonstrated that a structured, pharmacist-led intervention to optimize SUP prescribing was successfully implemented, reduced unnecessary use, and was well accepted by clinicians (60% acceptance). This suggests high feasibility for integrating rationale SUP use into routine practice where clinical pharmacy support is available.	**Panel comments**<br><br>• PPIs and H2RAs are feasible to implement in most ICU settings due to:<br>• Multiple formulations (oral and IV)<br>• Familiarity with dosing<br>• Widespread formulary inclusion

SUMMARY OF JUDGEMENTS

	PPIs vs. H2RAs	PPIs vs. SUCRALFATE	H2RAs vs. SUCRALFATE	PPIs PO vs. H2RAs PO/IV	SUCRALFATE vs. PPIs IV	SUCRALFATE vs. H2RAs IV	PPIs HIGH DOSE vs. H2RAs LOW DOSE	PPIs LOW DOSE vs. H2RAs LOW DOSE	H2RAs HIGH DOSE vs. PPIs HIGH DOSE
**PROBLEM**	**Yes**								
**DESIRABLE EFFECTS**	Small	Moderate	Trivial	Moderate	Trivial	Trivial	Moderate	Moderate	Trivial
**UNDESIRABLE EFFECTS**	Trivial	Small	Trivial	Trivial	Small	Small	Trivial	Trivial	Trivial
**NET BALANCE**	Moderate net desirable	Moderate net desirable	Moderate net desirable	Null net balance	Null net balance	Null net balance	Null net balance	Null net balance	Null net balance
**CERTAINTY OF EVIDENCE**	Moderate	Moderate	Moderate	Very low	Low	Low	Low	Very low	Low
**VALUES**	Possibly important uncertainty or variability	Probably no important uncertainty or variability	Probably no important uncertainty or variability	Possibly important uncertainty or variability	Probably no important uncertainty or variability	Probably no important uncertainty or variability	Possibly important uncertainty or variability	Possibly important uncertainty or variability	Possibly important uncertainty or variability
**BALANCE OF EFFECTS**	Does not favor either the intervention or the comparison	Probably favors the intervention	Probably favors the intervention	Varies	Don't know	Don't know	Don't know	Don't know	Don't know
**RESOURCES REQUIRED**	Varies	Negligible costs and savings	Negligible costs and savings	Varies	Varies	Varies	Varies	Varies	Varies
**CERTAINTY OF EVIDENCE OF REQUIRED RESOURCES**	No included studies	No included studies	No included studies	No included studies	No included studies	No included studies	No included studies	No included studies	No included studies
**COST EFFECTIVENESS**	Varies	Varies	Varies	Varies	Varies	Varies	Varies	Varies	Varies
**EQUITY**	Probably no impact	Probably no impact	Probably no impact	Probably no impact	Probably no impact	Probably no impact	Probably no impact	Probably no impact	Probably no impact

	PPIs vs. H2RAs	PPIs vs. SUCRALFATE	H2RAs vs. SUCRALFATE	PPIs PO vs. H2RAs PO/IV	SUCRALFATE vs. PPIs IV	SUCRALFATE vs. H2RAs IV	PPIs HIGH DOSE vs. H2RAs LOW DOSE	PPIs LOW DOSE vs. H2RAs LOW DOSE	H2RAs HIGH DOSE vs. PPIs HIGH DOSE
**ACCEPTABILITY**	Probably yes	Probably yes	Probably yes	Probably yes	Probably yes	Probably yes	Probably yes	Probably yes	Probably yes
**FEASIBILITY**	Probably yes	Probably yes	Probably yes	Probably yes	Probably yes	Probably yes	Probably yes	Probably yes	Probably yes

CONCLUSIONS

Recommendation(s)

Recommendation #1 (for PPIs vs. H2RAs)

We suggest using either proton pump inhibitors (PPIs) or histamine-2 receptor antagonists (H2RAs) as first-line agents for stress ulcer prophylaxis (SUP) in critically ill adults with risk factors for clinically important upper gastrointestinal bleeding (CIB), compared with no prophylaxis **(conditional recommendation, moderate certainty of evidence).**

Remark: The choice between PPIs and H2RAs should be individualized based on:

- **Patient's baseline risk of UGIB:** PPIs may be more effective, particularly in patients at higher bleeding risk (>4%), where the absolute risk reduction for clinically important bleeding (CIB) and overt bleeding may be meaningful.
- **Baseline severity of illness:** Until more data are available, clinicians should be cautious when using PPIs in patients with high illness acuity. Subgroup analyses from trials such as PEPTIC and SUP-ICU have suggested a possible signal of increased mortality with PPIs in sicker patients—potentially related to disruption of the intestinal microbiota, which may impair host defenses. Although the credibility of this signal is low, **H2RAs may be preferred** in these high-risk subgroups. Clinicians should carefully weigh this uncertainty when selecting an agent for the most critically ill patients.
- **Institutional formulary availability**
- **Potential for clinically significant drug–drug interactions** (See "Clinical Considerations" section for guidance)

Recommendation #2 (for PPIs vs. Sucralfate or H2RAs vs. Sucralfate)

In critically ill patients who are going to receive stress ulcer prophylaxis, we suggest using a proton pump inhibitor (PPI) or histamine-2 receptor antagonist (H2RA) over sucralfate as a first-line agent **(conditional recommendation, low to moderate certainty of evidence)**.

Recommendation #3 (Enteral vs. intravenous routes)

We suggest that pharmacologic SUP may be administered via either the enteral or intravenous route in critically ill adults with risk factors for clinically important stress-related UGIB, based on clinical context and feasibility **(conditional recommendation, low certainty of evidence).**

Remark:

This variation reflects clinical practicality, not evidence of superiority. The recommendation remains flexible, acknowledging both routes are used effectively.

Statement #4 (Dose of pharmacologic SUP)

Best Practice Statement (BPS) 1: Low dose pharmacologic SUP should be administered in critically ill adults with risk factors for clinically important stress-related UGIB compared to high dose **(Best Practice Statement – not GRADEd).**

Remark:

- "Low-dose" PPI therapy is defined as a daily dose of less than or equal to 40 mg esomeprazole, omeprazole, or pantoprazole and less than or equal to 30 mg lansoprazole.
- "Low-dose" H2RA therapy is defined as a daily dose of less than or equal to 40 mg famotidine, less than or equal to 150 mg IV ranitidine, less than or equal to 300 mg enteral ranitidine, and less than or equal to 1200 mg cimetidine.

Good practice statements are unGRADEd statements and reflect the general practice of panel experts. The ultimate judgment regarding any specific care must be made by the treating clinician and the patient, taking into consideration the individual circumstances of the patient, available treatment options, and resources. This best practice aligns with SCCM guidance and meets GRADE BPS criteria. Escalating beyond low-dose therapy offers minimal additional benefit, while increasing cost and side effects. The panel emphasized that clinical judgment remains essential, especially in patients with NPO status or complex pharmacokinetics.

Justification

PPIs are associated with a lower incidence of clinically important and overt gastrointestinal bleeding compared to H2RAs. However, concerns about the methodological quality of the underlying evidence—including variable definitions of bleeding, unusually high event rates, imbalance in patient risk factors, and overlapping interventions—limit the overall certainty of this finding.

Evidence on **mortality**, **pneumonia**, and ***Clostridioides difficile*** **infection** suggests **no important differences between PPIs and H2RAs**. While some subgroup analyses (e.g., among sicker patients) have raised concerns about increased mortality with PPIs, particularly in large trials such as **PEPTIC** and **SUP-ICU**, the **credibility of these subgroup effects remains low** and should be interpreted with caution.

There were **insufficient data** to compare **intravenous versus enteral routes** of administration within drug classes. No advantage was observed for IV administration in any comparison; thus, **enteral therapy is preferred when feasible**.

Similarly, data were **inadequate to assess the comparative effectiveness of high-dose versus low-dose therapy** within medication classes. Available evidence does not suggest that **higher doses offer additional benefit** over standard low-dose regimens.

Subgroup Considerations

Subgroup analyses (e.g., among sicker patients) have raised concerns about increased mortality with PPIs, particularly in large trials such as PEPTIC and SUP-ICU; the credibility of these subgroup effects remains low.
Additional high-quality randomized controlled trials are needed to clarify the balance of benefits and harms across subgroups, particularly those stratified by baseline bleeding risk and severity of illness.

Multiple Chronic Conditions and Polypharmacy

None

Clinical Considerations

1. Agent Selection
When selecting an agent for SUP, clinicians must weigh the potential benefits of reducing gastrointestinal bleeding against possible risks—particularly mortality and infectious complications—in critically ill patients. Clinical decision support (CDS) embedded within electronic medical records (EMRs) can assist in agent selection based on institution-specific risk models.
PPIs may have clinically relevant drug interactions due to their greater impact on gastric pH compared to H2RAs. These interactions can affect the absorption and efficacy of medications such as atazanavir, itraconazole, posaconazole, rilpivirine, high-dose methotrexate, and tyrosine kinase inhibitors (TKIs). In some cases, alternative agents (e.g., H2RAs) or dose adjustments may be necessary. Strategies to manage interactions include spacing administration, using acidic beverages for dilution, or switching therapies. The interaction between omeprazole and clopidogrel remains controversial, with inconsistent evidence on clinical outcomes. While many interactions are described in general populations, data in critically ill patients are limited.

A nested case–control study using >900 cases and >3,600 controls from Vanderbilt's biobank (BioVU) and electronic health record database examined the association between **omeprazole exposure and myopathy**, focusing on both **drug–drug (DDIs)** and **drug–gene interactions (DGIs)** involving **CYP2C19** and **CYP3A4** enzymes. **Key Findings:**

- **DDIs:** Concomitant **omeprazole + fluconazole** significantly increased the odds of myopathy (**adjusted OR 1.75, 95% CI 1.17–2.63**).
- **DGIs:** Risk was markedly higher among patients with **CYP2C19 poor metabolizer / CYP3A4 intermediate metabolizer** phenotypes (**AOR 4.77, 95% CI 1.74–13.1**).
- **Mechanistic Insight:** Fluconazole inhibits both CYP2C19 and CYP3A4, leading to accumulation of omeprazole and possible **dose-dependent myocyte injury**; genetic variants further potentiate this effect.
- **Clinical Interpretation:** While the absolute incidence of omeprazole-related myopathy remains low, findings highlight the importance of **pharmacogenetic variability and drug-interaction vigilance** in critically ill patients exposed to multiple agents metabolized through CYP pathways.

2. Route of Administration

- Intravenous-to-enteral conversion protocols—widely implemented for other medications—should be extended to SUP agents.
- Several enteral PPI formulations are available. Institutions should assess which formulation offers the best safety, efficacy, and cost-effectiveness, especially for patients receiving medications via feeding tubes.
- Standardized order sets should prioritize low-dose options for SUP.
- **Panel comment:** IV is the dominant route in practice, especially in unstable patients. In stable patients with NGT, the enteral route is acceptable and may offer cost savings.

3. Dosing and Duration

- SUP regimens should reflect current clinical practice rather than solely regulatory approvals.
- Special attention is warranted for patients who are NPO (nothing by mouth), as PPIs require active parietal cells for optimal effect. More frequent dosing (e.g., every 12 h) may be more effective than once-daily regimens in this group.
- SUP duration should be tailored to individual patient risk factors and re-evaluated regularly to avoid unnecessary therapy.

Research Priorities

- Head-to-Head RCTs: High-quality randomized controlled trials comparing PPIs and H2RAs in severely ill patients in the ICU are needed, powered for outcomes including mortality, clinically important bleeding, and pneumonia.
- Enteral vs. IV Therapy: Comparative studies assessing the pharmacodynamics, absorption consistency, and complications related to enteral vs. intravenous administration.
- Formulation Comparisons: Evaluate the safety, cost, and efficacy of different enteral PPI formulations to inform formulary decisions.
- Include cost and resource utilization analyses to guide value-based implementation of SUP protocols.
- Evaluate the personalized application of SUP—tailoring treatment to specific patient characteristics or conditions.
- Develop and validate AI predictive models or scoring tools to stratify patients by their risk of stress-related UGIB, incorporating factors such as illness severity, duration of mechanical ventilation, and enteral nutrition (EN) status.
- Integration of AI-based clinical decision support tools to guide SUP agent selection based on patient-specific risk factors.
- Investigate optimal dosage regimens for patients with organ dysfunction, advanced age, or unique pharmacogenomic profiles.
- Explore dosing strategies that align with real-world practice and current risk stratification tools.

Supplemental Content 2.25 Characteristics of Studies Identified for PICOs 4 and 5

Author, Year, Setting	Study type	Sample size (n)	Follow-up period	Intervention	Control	Main outcome(s)	Comments/Notes
Choi et al., 2019, South Korea	Retrospective cohort (pre-post intervention)	317	Hospital stay	Multidisciplinary quality improvement initiative for SUP use	Pre-intervention period	Reduction in inappropriate SUP use and related costs	Effectiveness particularly noted in non-ICU settings
Domond et al., 2017, USA	Quality Improvement Project	54	30 days post discharge	Nutrition consultation and QI strategy for AAH	Usual care	Compliance with nutritional guidelines; mortality, readmission	Very low baseline compliance; noted benefits from hepatology/nutrition input
Palmowski et al., 2024, Germany	Nationwide retrospective cohort, PSM	11576	2 years	Timely cessation of PPI therapy post-discharge	Continued PPI therapy post-discharge	Morbidity, rehospitalization, mortality	Overuse associated with worse outcomes; supported PPI cessation post-SUP
Farrell et al., 2010, Canada	Prospective audit and retrospective review	2153	Not specified	Guideline implementation for SUP prescribing	Historical prescribing pattern	Reduction in inappropriate PPI use and prescribing cost	Educational plus prescriber feedback strategy
Murphy et al., 2008, USA	Retrospective cohort	192	Hospital stay	Pharmacy-led assessment of PPI appropriateness	No control group; observational	Prevalence of inappropriate PPI prescribing	Emphasized role of pharmacists in stewardship
Wohlt et al., 2007, USA	Retrospective cohort	394	Hospital stay and post-discharge (up to 247 days)	Evaluation of SUP continuation post-ICU	Usual care	Proportion discharged inappropriately on gastric acid suppressants; cost analysis	Found 24.4% inappropriate discharge prescriptions; significant cost impact

Author, Year, Setting	Study Type	Sample Size (n)	Follow-up Period	Intervention	Control	Main Outcome(s)	Comments/Notes
Li et al., 2025; 26 ICUs in tertiary hospitals across China	Stepped-wedge, cluster-randomized controlled trial	2,199 (1018 control; 1181 intervention)	During ICU stay	Pharmacist-led multifaceted intervention: training, evidence-based manual, real-time review & recommendations on SUP	Usual care by ICU pharmacists (rounding, retrospective review)	Primary: Proportion receiving SUP and overt GI bleeding. Intervention group had significantly lower SUP use (45.5% vs. 49.5%, p=0.017). No significant difference in overt GI bleeding (3.7% vs. 4.0%).	High-quality design. Co-primary outcomes. Registered trial. Addresses overuse of SUP in ICU. Focused on implementation feasibility in Chinese ICUs.

Supplemental Content 2.26 Risk of Bias Assessment Table (The Risk Of Bias In Non-Randomized Studies – of Interventions, Version 2 [ROBINS-I V2] Assessment Tool for PICOs 4 and 5

Author, Year	Confounding	Selection bias	Classification of interventions	Deviations from intended interventions	Missing data	Measurement of outcomes	Reporting bias
Choi et al., 2019	Moderate<br>Adjusted for known risk factors but limited detail on residual confounding	Low<br>Consecutive patients with clear inclusion criteria	Low<br>Intervention well-described (pre-post design)	Low<br>Multidisciplinary program executed as planned	Low<br>No indication of missing outcome data	Moderate<br>Clinical documentation reviewed retrospectively	Low<br>All planned outcomes appear reported
Domond et al., 2017	Serious<br>No control for confounders, QI project with likely baseline imbalance	Moderate<br>No clear description of sampling strategy	Low<br>Clearly described intervention	Moderate<br>Implementation details partially described	Moderate<br>No formal mention of completeness or handling	Serious<br>Lack of blinded assessment; subjective outcome (nutrition)	Moderate<br>No protocol available; selective reporting possible
Palmowski et al., 2024	Moderate<br>Used propensity score matching, but residual confounding possible	Low<br>National claims database; inclusion criteria clearly applied	Low<br>Continuation vs. cessation clearly categorized	Low<br>Exposure defined post-discharge	Low<br>Only complete cases analyzed	Low<br>Mortality and rehospitalization from administrative data	Low<br>All outcomes prespecified and reported
Farrell et al., 2010	Moderate<br>Historical control without concurrent comparison;	Low<br>Large hospital-wide sample, clear inclusion	Low<br>Intervention and control periods well defined	Low<br>Intervention applied through institutional policy and audit	Low<br>Prescribing data retrieved from administrative system	Low<br>Outcome (prescription rates, costs) objective	Low<br>All key outcomes reported

	potential time trend effects						
Murphy et al., 2008	Serious<br>No control group; no adjustment for confounders	Moderate<br>Unclear sampling procedures; based on EMR review	Low<br>PPI use well documented	Low<br>Observational study of current practice	Moderate<br>Limited info on data completeness	Moderate<br>Based on clinical documentation; some subjectivity	Moderate<br>No protocol; limited scope of outcomes
Wohlt et al., 2007	Moderate<br>Retrospective design without adjustment for prognostic differences	Low<br>All ICU discharges included, broad eligibility	Low<br>Discharge prescriptions clearly categorized	Low<br>Descriptive study	Moderate<br>Post-discharge follow-up incomplete for some patients	Low<br>Use of administrative and clinical records for outcome assessment	Low<br>All key outcomes and cost analysis reported

Cochrane Collaboration Tool for Assessing Risk of Bias (ROB-2) for RCT

Reference	Domain 1: Risk of bias arising from the randomization process	Domain 2: Risk of bias due to deviations from the intended interventions (effect of assignment to intervention)	Domain 3: Risk of bias due to missing outcome data	Domain 4: Risk of bias in measurement of the outcome	Domain 5: Risk of bias in selection of the reported result	Overall risk of bias
Li 2025	Low risk	Some concern	Low risk	Low risk	Low risk	Some concern

Supplemental content 2.27 GRADE Evidence Profile for PICOs 4 and 5

Certainty assessment							№ of patients		Effect		Certainty	Importance
№ of studies	Study design	Risk of bias	Inconsistency	Indirectness	Imprecision	Other considerations	Continued use of SUP	discontinued use of SUP	Relative (95% CI)	Absolute (95% CI)		
**Inappropriate SUP Initiation and Continuation- Domond 2017 et al**												
1	non-randomized studies	serious[a]	not serious	not serious	very serious[b]	none	No comparison data available. Intervention data from cohort study: N= 395 SUP was inappropriately continued on 74% of total patients. Subsequently, 20% were eventually discharged newly on SUP. Of those newly discharged on SUP, 67% were inappropriate. Longer LOS was associated with being newly discharged on inappropriate SUP.				⊕◯◯◯ Very low[a,b]	
**Inappropriate SUP Continuation- (Choi 2019)**												
1	non-randomized studies	serious[c]	not serious	serious[d]	serious[b]	none	**Choi et al.: Pre and post study evaluate the impact of multidisciplinary team efforts to reduce inappropriate use of SUP (N=317).** The incidence of inappropriate use of SUP declined by 12.3% from 58 (37.9%) patients prior to intervention to 42 (25.6%) patients post intervention, and the adjusted OR indicated a statistically significant risk reduction: 0.51 (95% CI 0.31–0.86, P = 0.01). The expenses associated with inappropriate utilization of SUP in ICU showed a greater relative decrease post intervention, almost 92.4%, from US$55.33 to US$4.23.				⊕◯◯◯ Very low[b,c,d]	
**Inappropriate SUP continuation (Li 2025)**												
1	randomized trials	not serious	not serious	not serious	not serious	none	Evaluate the effectiveness of a pharmacist-led intervention in decreasing the overuse of SUP. The proportion of patients receiving SUP in the intervention group was lower than that in the control group (45.5% vs. 49.5%; odds ratio [OR], 0.81; 95% CI, 0.68–0.96; p = 0.017).The proportion of patients with overt gastrointestinal bleeding was similar (3.7% vs. 4.0%; OR, 1.05; 95% CI, 0.65–2.85; p = 0.81).				⊕⊕⊕⊕ High	
**Inappropriate SUP Continuation Post-ICU (Farley 2013)**												

1	non-randomized studies	serious[c]	not serious	not serious	very serious[b,e]	none	329 of the 387 patients (85%) were prescribed SUP medications. Of these, 63% had their SUP continued in the ward without any obvious indication, and many (39%) had their SUP medications inappropriately continued on discharge from hospital.<br>• **Overall**, a substantial proportion of patients received SUP in the ICU without clear indications, and many continued to receive it inappropriately after transfer out of ICU and at discharge.<br>• **Cardiac surgical patients** had the highest rates of both ICU SUP initiation (95%) and inappropriate continuation in the ward (94%) and at discharge (86%).<br>• **Other surgical patients** also showed high rates: 73% received SUP, with 80% inappropriately continuing in the ward and 79% at discharge.<br>• Among **older patients (>65 years)**, 81% received SUP in ICU, and of these, 85% continued in the ward and 84% at discharge without justification.<br>• **Patients ≤65 years old** had similar ICU initiation rates (82%) but slightly lower inappropriate continuation rates—76% in the ward and 69% at discharge.<br>• Males had higher inappropriate continuation rates (84% in the ward; 78% at discharge) compared to females (74% and 71%, respectively).<br>• **Elective admissions** showed particularly high inappropriate continuation in the ward (89%) and discharge (84%), significantly more than **emergency admissions** (74% and 72%).<br>• Patients in the **tertiary ICU** were more likely to receive SUP (92%) than those in **metropolitan ICUs** (69%), although continuation rates were similar.<br>• **Surgical patients** had higher inappropriate continuation in the ward (88%) compared to **medical patients** (68%).<br>Across nearly all subgroups, inappropriate continuation of SUP was common, especially among surgical and cardiac surgery patients, older individuals, and those admitted electively. These findings highlight a critical opportunity for stewardship interventions to discontinue unnecessary SUP after ICU discharge.	⊕◯◯◯ Very low[b,c,e]	

Inappropriate SUP Continuation (Murphy 2008)

1	non-randomized studies	serious[c]	not serious	not serious	serious[e]	none	A total of 248 consecutive adult patients admitted to the SICU continued AST post-SICU vs. AST discontinued when risk factors resolved 24.2% discharged on SUP, only 5% with valid indication. Continuation of SUP during hospitalization outside the SICU occurred in 215 patients (86.7%). Sixty patients (24.2%) were discharged from the hospital receiving SUP. **Presence of risk factors for stress –related GIB at SICU admission appears to influence continuation of SUP after discharge from the hospital.** Patients who continued to receive SUP after hospital discharge required extended mechanical ventilation ($p = 0.001$); had twice as many risk factors for gastrointestinal bleeding ($p < 0.001$); were frequently discharged with anticoagulant therapy ($p < 0.001$); exhibited longer hospital and SICU stays ($p < 0.001$); more frequently demonstrated Glasgow Coma Scale scores of 8 or lower; and/or had head injury ($p < 0.001$), hepatic failure ($p = 0.004$), and major trauma ($p = 0.049$).	⊕◯◯◯ Very low[c,e]	

Inappropriate SUP Continuation (Wohlt 2007)

1	non-randomized studies	serious[c]	not serious	not serious	serious[e]	none	n= 357; 80% continued on gastric acid suppressants on transfer from the ICU, with 60% of the therapy being inappropriate. The percentage of critically ill patients discharged from the hospital with inappropriate prescription of gastric acid suppressants was 24.4%	⊕◯◯◯ Very low[c,e]	

Inappropriate SUP Continuation (Palmowski 2024)

1	non-randomized studies	serious[c]	not serious	not serious	serious[e]	none	11,576 patients in the ICU who received PPI therapy for the first time during their index ICU stay without having an indication for its continuation. **INTERVENTIONS**: The cohort was stratified into two groups: 1) patients without further PPI therapy and 2) patients with continuation of PPI therapy beyond 8 weeks after hospital discharge. The proportion of patients with continued PPI therapy without an objectifiable indication was 41.7%.<br>**Associations With Adverse Effects of Unnecessary PPI Therapy**<br>*Cardiopulmonary Risks*<br>• **27% increased risk of pneumonia** (OR 1.27; 95% CI, 1.15–1.39; p < 0.001)<br>• **17% increased risk of cardiovascular events** (OR 1.17; 95% CI, 1.08–1.26; p < 0.001)<br>*Renal Disease*<br>• **26% increased risk of chronic renal failure** (OR 1.26; 95% CI, 1.12–1.41; p < 0.001)<br>• No significant difference in **acute interstitial nephritis** (OR 1.21; 95% CI, 0.70–2.08; p = 0.69)<br>*Cancer Risks*<br>• **2.7-fold increased risk of esophageal cancer** (OR 2.74; 95% CI, 1.37–5.47; p = 0.004)<br>• **2.4-fold increased risk of pancreatic cancer** (OR 2.44; 95% CI, 1.63–3.64; p < 0.001)<br>• **19% increased risk of colorectal cancer** (OR 1.19; 95% CI, 1.03–1.37; p = 0.006)<br>• No increased risk of **gastric neoplasms** (OR 0.87; 95% CI, 0.56–1.33; p = 0.51)<br>*Malabsorption and Electrolyte Disorders*<br>• **1.3-fold increased risk of vitamin B12 deficiency** (OR 1.30; 95% CI, 1.13–1.49; p < 0.001)<br>• **2.1-fold increased risk of hypomagnesemia** (OR 2.11; 95% CI, 1.24–3.60; p = 0.006)<br>• **1.6-fold increased risk of hypocalcemia** (OR 1.55; 95% CI, 1.22–1.96; p < 0.001)<br>**Health Outcomes**<br>**1-Year Rehospitalization**	⊕◯◯◯ Very low[c,e]	

							• **73.4%** of patients with continued unindicated PPI therapy were rehospitalized<br>• Compared to **67.3%** without PPI therapy<br>• **6.1% absolute risk increase**; **35% higher odds of rehospitalization** (OR 1.35; 95% CI, 1.23–1.47; $p < 0.001$)<br>*2-Year Mortality*<br>• **72.5%** survival rate with continued PPI therapy vs. **74.7%** without PPI		

CI=confidence interval
a. Risk of bias due to lack of comparison data.
b. Small sample size, fragility of estimate.
c. Risk of bias due to lack of randomized comparison and potential confounding in observational designs.
d. Potential issues with generalizability due to single-center and regional focus as well as absence of patient-centered clinical outcome data (e.g., adverse events from continuation).
e. Imprecision due to lack of confidence intervals and reliance on descriptive statistics.

Supplemental content 2.28 PICOs 4 and 5: Evidence-to-Decision (EtD) Frameworks

Question

**Should continued use of stress ulcer prophylaxis (SUP) be recommended for critically ill adults in the ICU when risk factors for stress-related upper gastrointestinal bleeding (UGIB) are no longer present or were present at admission?**	
**Population:**	Critically ill adults
**Intervention:**	PICO 4: Should critically ill adults whose UGIB risk factors are no longer present continue or discontinue SUP?<br><br>PICO 5: Should critically ill adults without UGIB risk factors but receiving SUP before ICU admission continue or discontinue SUP?
**Comparison:**	Discontinuation of SUP
**Outcomes:**	Inappropriate use of SUP

Assessment

**Problem**<br>Is the problem a priority?		
**Judgement**	**Research evidence**	**Additional considerations**
○ No<br>○ Probably no<br>○ Probably yes<br>● Yes<br>○ Varies<br>○ Don't know	The inappropriate continuation of Stress Ulcer Prophylaxis (SUP) after ICU discharge is a **widespread**, **systemic**, and **high-impact** issue, making it a high-priority target for intervention.<br>**Supporting Evidence: (1)(2)(3)(4, 5, 6, 7)**<br>• **Choi et al. (2019)** observed that **80–90%** of patients in the ICU receive SUP, despite the majority **not meeting continued therapy indications**, especially post-ICU.<br>• **Farley (2013)** found that **63%** of patients in the ICU who began SUP without indication continued it on the general ward, and **39%** were discharged inappropriately with it.<br>• **Farrell (2010)** and **Domond (2017)** both reported over **50%** of patients continued SUP beyond ICU without indication. Domond noted that **74%** of patients continued SUP inappropriately after downgrade.<br>• **Palmowski (2024)** emphasized the **long-term implications** of this pattern, with **41.7%** of patients in the ICU discharged on PPIs without an indication, raising risks for polypharmacy and adverse events.<br>• **Krag et al. (2015)**, in a global ICU survey, showed that while **99%** of ICUs used SUP, only **64%** had supporting guidelines, revealing **policy-practice gaps**.	

	• **Buendgens et al. (2016)** and **Li et al. (2025)** further corroborated the trend, highlighting routine use of SUP even in **low-risk patients** where bleeding risk is minimal, indicating **habitual overuse**.<br>• **Bardou (2015)** found that clinically significant UGIB is **rare (<1%)**, yet SUP continues routinely **post-ICU**, signifying a mismatch between **perceived** and **actual** need.<br>• All studies converge on the notion that the **baseline risk of GI bleeding has declined** due to modern ICU practices (e.g., early enteral feeding, sedation protocols), shifting the risk-benefit calculus and making continued SUP **increasingly unjustifiable**.<br>**Consequences:**<br>• This pattern contributes to:<br>• **Polypharmacy** and increased medication burden.<br>• **Unnecessary costs** and resource use.<br>• **Increased risk** of long-term complications (e.g., C. difficile, pneumonia, kidney injury from chronic PPI use).<br>• The inappropriate continuation is **predictable**, **measurable**, and **modifiable**, making it suitable for quality improvement and de-prescribing interventions.	
**Desirable Effects**<br>How substantial are the desirable anticipated effects?		
**Judgement**	**Research evidence**	**Additional considerations**
**Trivial:**<br>**Small:**<br>**Moderate:**<br>**Large:**<br>**Varies:**<br>**Don't know:** PICO 4: Should critically ill adults whose UGIB risk factors are no longer present continue or discontinue SUP?<br>PICO 5: Should critically ill adults without UGIB risk factors but receiving SUP before ICU	A narrative summary of evidence<br><br>**PICO 4: Should critically ill adults whose UGIB risk factors are no longer present continue or discontinue SUP?**<br>**Farrell 2010, Domond 2017, and Boyd 2021:** The evidence is limited to cohort studies comparing characteristics of patients in whom SUP was discontinued during the ICU stay or hospitalization to those in whom it was continued beyond the ICU stay or hospitalization. Factors contributing to the continuation of SUP after ICU discharge were admission diagnoses requiring mechanical ventilation, the presence of multiple traumas, hepatic failure, head injury or spinal cord injury at admission, longer duration of mechanical ventilation, and longer stay in the ICU. The use of PPIs for SUP may contribute to continued use of therapy after ICU discharge. Therefore, SUP should be discontinued when critical illness is no longer evident<br><br>**PICO 5: Should critically ill adults without UGIB risk factors but receiving SUP before ICU admission continue or discontinue SUP?**	

<table>
<tr>
<td>admission continue or discontinue SUP?</td>
<td>
The PEPTIC study included patients receiving SUP therapy before ICU admission and did not find evidence of effects that outcomes were influenced by prior use. Cohort studies also found no evidence of effects on outcomes based on usage before ICU admission.
<table>
<tr><th rowspan="2">Outcomes</th><th colspan="2">Anticipated absolute effects* (95% CI)</th><th rowspan="2">Relative effect (95% CI)</th><th rowspan="2">№ of participants (studies)</th><th rowspan="2">Certainty of the evidence (GRADE)</th><th rowspan="2">Comments</th></tr>
<tr><th>Risk with discontinued use of SUP</th><th>Risk with continued use of SUP</th></tr>
<tr><td>Inappropriate SUP Initiation and Continuation (Domond et al. 2017)</td><td colspan="2">No comparison data available. Intervention data from cohort study: N= 395. SUP was inappropriately continued on 74% of total patients. Subsequently, 20% were eventually discharged newly on SUP. Of those newly discharged on SUP, 67% were inappropriate. Longer LOS was associated with being newly discharged on inappropriate SUP.</td><td>-</td><td>(1 non-randomized study)</td><td>⊕◯◯◯ Very low[a,b]</td><td></td></tr>
<tr><td>Inappropriate SUP Continuation- (Choi 2019)</td><td colspan="2">Choi et al.: Pre and post study evaluate the impact of multidisciplinary team efforts to reduce inappropriate use of SUP (N=317). The incidence of inappropriate use of SUP declined by 12.3% from 58 (37.9%) patients prior to intervention to 42 (25.6%) patients post intervention, and the adjusted OR indicated a statistically significant risk reduction: 0.51 (95% CI 0.31–0.86, P = 0.01) The expenses associated with inappropriate utilization of SUP in ICU showed a greater relative decrease post intervention, almost 92.4%, from US$55.33 to US$4.23.</td><td>-</td><td>(1 non-randomized study)</td><td>⊕◯◯◯ Very low[b,c,d]</td><td></td></tr>
</table>
</td>
<td></td>
</tr>
</table>

	Inappropriate SUP Continuation (Li 2025)	Evaluate the effectiveness of a pharmacist-led intervention in decreasing the overuse of SUP. The proportion of patients receiving SUP in the intervention group was lower than that in the control group (45.5% vs. 49.5%; odds ratio [OR], 0.81; 95% CI, 0.68–0.96; p = 0.017). The proportion of patients with overt gastrointestinal bleeding was similar (3.7% vs. 4.0%; OR, 1.05; 95% CI, 0.65–2.85; p = 0.81).	-	(1 RCT)	⊕⊕⊕⊕ High		
	Inappropriate continuation of SUP Post-ICU (Farley 2013)	329 of the 387 patients (85%) were prescribed SUP medications. Of these, 63% had their SUP continued in the ward without any obvious indication, and many (39%) had their SUP medications inappropriately continued on discharge from hospital.<br>• **Overall**, a substantial proportion of patients received SUP in the ICU without clear indications, and many continued to receive it inappropriately after transfer out of the ICU and at discharge.<br>• **Cardiac surgical patients** had the highest rates of both ICU SUP initiation (95%) and inappropriate continuation in the ward (94%) and at discharge (86%).<br>• **Other surgical patients** also showed high rates: 73% received SUP, with 80% inappropriately continuing in the ward and 79% at discharge.	-	(1 non-randomized study)	⊕◯◯◯ Very low[b,c,e]		

		• Among **older patients (>65 years)**, 81% received SUP in ICUs, and of these, 85% continued in the ward and 84% at discharge without justification.<br>• **Patients ≤65 years old** had similar ICU initiation rates (82%), but slightly lower inappropriate continuation rates—76% in the ward and 69% at discharge.<br>• Males had higher inappropriate continuation rates (84% in the ward; 78% at discharge) compared to females (74% and 71%, respectively).<br>• **Elective admissions** showed particularly high inappropriate continuation in the ward (89%) and discharge (84%), significantly more than **emergency admissions** (74% and 72%).<br>• Patients in the **tertiary ICU** were more likely to receive SUP (92%) than those in **metropolitan ICUs** (69%), although continuation rates were similar.<br>• **Surgical patients** had higher inappropriate continuation in the ward (88%) compared to **medical patients** (68%).<br>Across nearly all subgroups, inappropriate continuation of SUP was common, especially among surgical and cardiac surgery patients, older individuals, and those admitted electively. These findings					

		highlight a critical opportunity for stewardship interventions to discontinue unnecessary SUP after ICU discharge.					
	Inappropriate SUP Continuation (Murphy 2008)	A total of 248 consecutive adult patients admitted to the SICU Continued AST post-SICU vs. AST discontinued when risk factors resolved 24.2% discharged on SUP, only 5% with valid indication. Continuation of SUP during hospitalization outside the SICU occurred in 215 patients (86.7%). Sixty patients (24.2%) were discharged from the hospital receiving SUP. **Presence of risk factors for stress–related GIB at SICU admission appears to influence continuation of SUP after discharge from the hospital.** Patients who continued to receive SUP after hospital discharge required extended mechanical ventilation (p=0.001); had twice as many risk factors for gastrointestinal bleeding (p < 0.001); were frequently discharged with anticoagulant therapy (p < 0.001); exhibited longer hospital and SICU stays (p < 0.001); more frequently demonstrated Glasgow Coma Scale scores of 8 or lower; and/or had head injury (p < 0.001), hepatic failure (p = 0.004), and major trauma (p = 0.049).	-	(1 non-randomized study)	⊕◯◯◯ Very low[c,e]		
	Inappropriate SUP Continuation (Wohlt 2007)	N = 357; 80% continued on gastric acid suppressants on transfer from the ICU, with 60% of the therapy being inappropriate. The percentage of critically ill patients discharged from the hospital	-	(1 non-randomized study)	⊕◯◯◯ Very low[c,e]		

	with inappropriate prescription of gastric acid suppressants was 24.4%.				

a. Risk of bias due to lack of comparison data.
b. Small sample size, fragility of estimate.
c. Risk of bias due to lack of randomized comparison and potential confounding in observational designs.
d. Potential issues with generalizability due to single-center and regional focus as well as absence of patient-centered clinical outcome data (e.g., adverse events from continuation).
e. Imprecision due to lack of confidence intervals and reliance on descriptive statistics.

Undesirable Effects

How substantial are the undesirable anticipated effects?

Judgement	Research evidence	Additional considerations
**Trivial:**<br>**Small:**<br>**Moderate:**<br>**Large:**<br>**Varies:** PICO 4: Should critically ill adults whose UGIB risk factors are no longer present continue or discontinue SUP? PICO 5: Should critically ill adults without UGIB risk factors but receiving SUP before ICU admission continue or discontinue SUP?<br>**Don't know:**	data limited to PPIs<br>no studies on H2 blockers<br>(see table below)	One panel member indicated that the net balance is a moderate undesirable effect.<br><br>**Panel discussion**<br>An important signal identified in a 2021 pharmacovigilance study using the US FDA FAERS database indicated that proton pump inhibitors (PPIs), including pantoprazole and other agents, are significantly associated with both acute kidney injury (AKI) and chronic kidney disease (CKD). Notably, the signal strength for CKD was stronger (ROR = 8.80) than for AKI (ROR = 3.95), with substantial

Outcomes	Anticipated absolute effects* (95% CI)		Relative effect (95% CI)	№ of participants (studies)	Certainty of the evidence (GRADE)	Comments
	Risk with discontinued use of SUP	Risk with continued use of SUP				
Inappropriate SUP Continuation (Palmowski 2024)	11,576 patients in the ICU who received PPI therapy for the first time during their index ICU stay without having an indication for its continuation.<br>**INTERVENTIONS**: The cohort was stratified into two groups: 1) patients without further PPI therapy and 2) patients with continuation of PPI therapy beyond 8 weeks after hospital discharge. The proportion of patients with continued PPI therapy without an objectifiable indication was 41.7%.<br>**Associations With Adverse Effects of Unnecessary PPI Therapy**		-	(1 non-randomized study)	⊕◯◯◯<br>Very low[a,b]	

		**Cardiopulmonary Risks**<br>• **27% increased risk of pneumonia** (OR 1.27; 95% CI, 1.15–1.39; p < 0.001)<br>• **17% increased risk of cardiovascular events** (OR 1.17; 95% CI, 1.08–1.26; p < 0.001)<br><br>**Renal Disease**<br>• **26% increased risk of chronic renal failure** (OR 1.26; 95% CI, 1.12–1.41; p < 0.001)<br>• No significant difference in **acute interstitial nephritis** (OR 1.21; 95% CI, 0.70–2.08; p = 0.69)<br>**Cancer Risks**<br>• **2.7-fold increased risk of esophageal cancer** (OR 2.74; 95% CI, 1.37–5.47; p = 0.004)<br>• **2.4-fold increased risk of pancreatic cancer** (OR 2.44; 95% CI, 1.63–3.64; p < 0.001)<br>• **19% increased risk of colorectal cancer** (OR 1.19; 95% CI, 1.03–1.37; p = 0.006)<br>• No increased risk of **gastric neoplasms** (OR 0.87; 95% CI, 0.56–1.33; p = 0.51)<br>**Malabsorption and Electrolyte Disorders**<br>• **1.3-fold increased risk of vitamin B12 deficiency** (OR 1.30; 95% CI, 1.13–1.49; p < 0.001)<br>• **2.1-fold increased risk of hypomagnesemia** (OR 2.11; 95% CI, 1.24–3.60; p = 0.006)					proportions of adverse events leading to death, hospitalization, and disability. This strengthens the argument for discontinuation of SUP in low-risk ICU patients due to potential serious renal adverse outcomes even outside traditional GI harm concerns [ref: Wu B, Li D, Xu T, Luo M, He Z, Li Y. Proton pump inhibitors associated acute kidney injury and chronic kidney disease: data mining of US FDA adverse event reporting system. Sci Rep. 2021 Feb 11;11(1):3690.].

	• **1.6-fold increased risk of hypocalcemia** (OR 1.55; 95% CI, 1.22–1.96; p < 0.001)<br>**Health Outcomes 1-Year Rehospitalization**<br>• **73.4%** of patients with continued unindicated PPI therapy were rehospitalized<br>• Compared to **67.3%** without PPI therapy<br>• **6.1% absolute risk increase**; **35% higher odds of rehospitalization** (OR 1.35; 95% CI, 1.23–1.47; p < 0.001)<br>**2-Year Mortality**<br>• **72.5%** survival rate with continued PPI therapy vs. **74.7%** without PPI				

a. Risk of bias due to lack of randomized comparison and potential confounding in observational designs.
b. Imprecision due to lack of confidence intervals and reliance on descriptive statistics.

**Net balance**		
**Judgement**	**Research evidence**	**Additional considerations**
**Large net desirable:**<br>**Moderate net desirable:**<br>**Small net desirable:**<br>**Null net balance:**<br>**Small net undesirable:**<br>**Moderate net undesirable:** PICO 4: Should critically ill adults whose UGIB risk factors	• **SUP should be discontinued** when the patient is no longer critically ill or **no longer has risk factors for UGIB**.<br>• SUP is intended to be **short-term**, typically during ICU stay (e.g., median 5 days in the REVISE trial).<br>**Inappropriate continuation is common**:<br>• Up to **70%** of patients continue SUP after ICU discharge.<br>• Up to **44%** continue it inappropriately at hospital discharge.<br>• Often occurs despite no longer being at risk or when the patient was never on SUP prior to admission.<br>**Risks of unnecessary continuation**:	

are no longer present continue or discontinue SUP?<br>PICO 5: Should critically ill adults without UGIB risk factors but receiving SUP before ICU admission continue or discontinue SUP?<br>**Large net undesirable:**	• Associated with **adverse drug events (ADEs) (see table below).**<br>• Most ADEs occur during **long-term use.**<br>**Net Balance**<br>• Once critical illness has been resolved and UGIB risk factors are no longer present, **continuation of SUP offers no net clinical benefit**.<br>• The **known harms and costs** exceed the **benefit**.	

Summary of Reported Adverse Effects of H2RAs and PPIs for SUP

Adverse Effect	Drug Class	Mechanism	Timing	Risk Estimate	Notes
**Delirium**	H2RA	CNS H2 blockade → ↓ cholinergic activity	ICU	RR 1.15–1.36 depending on age/ventilation	Elevated risk particularly in ventilated or older patients
**Vitamin B12 deficiency**	H2RA & PPI	↓ Absorption due to hypochlorhydria	>2 years (mostly chronic use)	OR 1.25–1.65	More pronounced with prolonged therapy
**Acute Kidney Injury (AKI)**	PPI	Multiple proposed, including nephrotoxicity, pyroptosis	Post-discharge (varied)	aRR 1.75; OR ~1.2	Evidence mixed; some studies show no association
**Chronic Kidney Disease (CKD)**	PPI	Renal fibrosis, AKI progression	~6 months+	ROR 8.8 (dexlansoprazole strongest signal)	Strongest signal among all ADEs
**Clostridium difficile**	PPI	Altered gut flora	ICU & post-discharge	OR ~1.6–1.7	Consistent association in ICU & long-term studies
**Pneumonia (VAP or CAP)**	PPI	Altered flora → microaspiration	ICU & post-discharge	OR ~1.27	VAP risk not significantly elevated in some trials
**Cardiovascular Events**	PPI	↓ NO, ↑ endothelial aging, mineral malabsorption	Post-discharge	OR ~1.17	Emerging concern; needs more research
**Hypomagnesemia**	PPI	↓ Absorption	Post-discharge	OR 1.55	Often underrecognized; can lead to serious arrhythmias
**All-Cause Mortality**	PPI	Possibly mediated via other ADEs, microbiome effects	90-day or 1-year mortality	HR/RR ~1.05–1.17 (↑ in severe illness)	Mortality may increase **not confirmed** across all trials; varies by severity (e.g., SAPS >53, APACHE >25)

References: (8)
Shiddapur A et al. *Crit Care Explor*. 2021;3:e0507.
Lam JR et al. *JAMA*. 2013;310:2435–42. Palmowski L et al. *Crit Care Med*. 2024;52:190–9.
Zhang Y et al. *BMC Nephrol*. 2023;24:150. Han CT et al. *J Clin Med*. 2023;12:2467.
Krag M et al. *N Engl J Med*. 2018;379:2199–2208. D'Silva KM et al. *Clin Microbiol Infect*. 2021;27:697–703. Cook D et al. *N Engl J Med*. 2024;391:9–20. Wang Y et al. *NEJM Evid*. 2024;3.
Wu B et al. *Sci Rep*. 2021;11:3690.

	PEPTIC Investigators. *JAMA*. 2020;323:616–26.	
**Certainty of evidence**<br>What is the overall certainty of the evidence of effects?		
**Judgement**	**Research evidence**	**Additional considerations**
**Very low** PICO 4: Should critically ill adults whose UGIB risk factors are no longer present continue or discontinue SUP? PICO 5: Should critically ill adults without UGIB risk factors but receiving SUP before ICU admission continue or discontinue SUP?<br>**Low:**<br>**Moderate:**<br>**High:**<br>**No included studies:**	Data are from cohort studies.	**Panel comment**<br>Agree, but more arguments are needed for 'very low' - cohort studies are initially 'low' until downgraded for other factors.
**Values**<br>Is there important uncertainty about or variability in how much people value the main outcomes?		
**Judgement**	**Research evidence**	**Additional considerations**
**Important uncertainty or variability:**<br>**Possibly important uncertainty or variability:** PICO 4: Should critically ill adults whose UGIB risk factors are no longer present continue or discontinue SUP?	• **No direct data** on patient-reported values were available in the included studies. However, **inferences** from the clinical context and related findings provide insights into likely values and preferences of both **patients** and **clinicians**.<br>**Patient Perspectives (Indirect Evidence) (7, 6)**<br>• **Bardou** and **Buendgens** suggest that while patients may intuitively favor protective measures (e.g., SUP for GI bleeding), they likely place high value on avoiding:<br>• **Infection risks** (e.g., *C. difficile*, pneumonia),<br>• **Unnecessary medications**, and	

PICO 5: Should critically ill adults without UGIB risk factors but receiving SUP before ICU admission continue or discontinue SUP?<br>**Probably no important uncertainty or variability:**<br>**No important uncertainty or variability:**	• **Polypharmacy** and the long-term pill burden, particularly in the post-ICU recovery phase.<br>• These views align with broader **deprescribing literature**, which indicates a **general patient preference** for discontinuing non-essential medications when the risks outweigh the benefits.<br>**Clinician Perspectives and Variability (1, 5, 2, 9)**<br>• **Choi et al. (2019)** observed that clinicians and patients often focus on overt, catastrophic risks (e.g., GI bleeding), while **underappreciating hidden harms** (e.g., infection, delirium).<br>• **Krag et al. (2015)** reported highly variable discontinuation practices across ICUs, reflecting **uncertainty and inconsistent valuation** of GI bleeding prevention versus infection risks.<br>• **Farley (2013)** and **Farrell (2010)** attributed continuation to **habitual practice, lack of deprescribing protocols**, and **cognitive bias** favoring action (i.e., continuation) over inaction (i.e., deprescribing).<br>• **Li et al. (2025)** showed that when clear, evidence-based pathways (e.g., pharmacist feedback) are implemented, clinicians **accept deprescribing without negative outcomes**, indicating that **structured support aligns with clinician values**.<br>**Conclusion:**<br>• While **possibly important variability exists**, especially among clinicians in how benefits and harms are weighed, **most patients and providers** are likely to **favor discontinuation** of SUP when no ongoing risk is present.<br>• There is **probably no important variability** in core values such as safety, avoiding unnecessary treatment, and minimizing harm.<br>• **Structured deprescribing interventions** (e.g., checklists, pharmacist involvement) are not only effective but **acceptable** to both patients and clinicians, helping overcome behavioral inertia.	
**Balance of effects**<br>Does the balance between desirable and undesirable effects favor the intervention or the comparison?		
**Judgement**	**Research evidence**	**Additional considerations**
**Favors the comparison:**<br>**Probably favors the comparison:** PICO 4: Should critically ill adults whose UGIB risk factors are no longer present continue or discontinue SUP?<br>PICO 5: Should critically ill adults without UGIB	**Benefits of Discontinuation (1, 6, 9)**<br>• **Choi et al. (2019):** Discontinuation leads to **fewer adverse drug events** and **substantial cost savings**.<br>• **Buendgens et al. (2016):** Reduces risk of serious **infectious complications** such as *Clostridium difficile*-associated diarrhea and pneumonia.<br>• **Li et al. (2025):** A deprescribing initiative decreased SUP use by 4% **without increasing bleeding**, demonstrating a **safe and effective intervention**.<br>• **Domond (2017):** Inappropriate SUP continuation associated with **longer ICU stays**, suggesting potential for improved resource utilization via discontinuation.<br>**Harms of Continuation (1, 2, 5)**	

risk factors but receiving SUP before ICU admission continue or discontinue SUP?<br>**Does not favor either the intervention or the comparison:**<br>**Probably favors the intervention:**<br>**Favors the intervention:**<br>**Varies:**<br>**Don't know:**	• **Choi et al. (2019):** Ongoing SUP use increases the risk of **nosocomial pneumonia** and ***C. difficile* infection**.<br>• **Farley (2013):** Chronic SUP use linked to a range of complications, including **pneumonia, *C. difficile*, osteoporosis, vitamin B12 deficiency, and drug interactions.**<br>• **Krag et al. (2015):** 19% of ICUs continued SUP post-ICU despite resolved risk, unnecessarily exposing patients to harm.<br>• **Domond (2017):** 67% of patients discharged on acid suppressive therapy (AST) had **no indication**, further illustrating system-level overuse.<br>**Minimal Benefit of Continuation (7, 6)**<br>• **Bardou et al. (2015):** While SUP reduces GI bleeding risk in high-risk patients, the **absolute risk in low-risk or post-ICU patients is very low**. The **number needed to treat (NNT)** is high, diminishing clinical justification.<br>• **Buendgens et al. (2016):** Supports that bleeding risk post-ICU is **negligible**.<br>**Conclusion:**<br>• The **net benefit clearly favors discontinuation** in patients no longer at risk.<br>• Continued SUP in low-risk patients exposes them to **greater harm than benefit**.<br>• While some uncertainty remains in specific subgroups, **the overall balance of effects supports deprescribing** when risk factors resolve.	
**Resources required**		
**Judgement**	**Research evidence**	**Additional considerations**
**Large costs:**<br>**Moderate costs:**<br>**Negligible costs and savings:**<br>**Moderate savings:**<br>**Large savings:** PICO 4: Should critically ill adults whose UGIB risk factors are no longer present continue or discontinue SUP?<br>PICO 5: Should critically ill adults without UGIB risk factors but receiving	**Direct Cost Savings from Discontinuation** (10, 9, 1)<br>• **Choi et al. (2019):** Discontinuing inappropriate SUP use resulted in a **58.5% reduction in cost per 100 patient-days**, primarily by avoiding unnecessary medication and complications.<br>• **Li et al. (2025):** A deprescribing intervention reduced SUP use **without compromising patient safety** and resulted in **meaningful drug cost savings**.<br>• **Shin et al. (2015):**<br>• **57.7% of ICU** and **52.2% of non-ICU** patients were discharged inappropriately on PPIs.<br>• Over 4 years, this single Korean hospital incurred:<br>• **USD $40,175 in total unnecessary outpatient PPI costs**, and<br>• **USD $12,053 in patient out-of-pocket expenses**.<br>• These findings illustrate the **substantial financial burden** even in a localized setting—indicating **potential for large system-wide savings globally**.<br>**Indirect and Downstream Costs (8, 1)**	One panel member chose "Moderate to large."

SUP before ICU admission continue or discontinue SUP?<br>**Varies:**<br>**Don't know:**	• **Wong (2025)** and **Choi et al. (2019)** highlight that the **economic impact extends beyond drug costs**, due to the **adverse effects** of continued SUP.<br>• These complications lead to **high-cost interventions** and prolonged care needs, compounding total healthcare spending.<br>**Cost Inefficiency of Inappropriate Use (2, 7, 6)**<br>• **Bardou (2015):** PPIs can be cost-effective when used appropriately versus H2RAs, but **unnecessary continuation undermines that advantage**, leading to **cumulative cost waste**.<br>• **Buendgens et al. (2016):** Emphasizes the need for **ongoing medication review**, noting overtreatment results in **avoidable resource use**.<br>• **Farley (2013)** and **Domond (2017):** Link continued SUP use to **increased hospital length of stay** and **unjustified pharmaceutical expenditure**.<br>• **Domond (2017):** Cited PPIs as the **third-largest drug class by sales in the U.S. ($13.6 billion/year)**—showcasing the **economic footprint** of this drug class when overused.<br>**Conclusion:**<br>• The evidence consistently supports that **discontinuing SUP when not indicated yields significant cost savings**, both in direct drug costs and by **avoiding costly adverse events**.<br>• In **resource-limited settings**, deprescribing represents a **high-value, low-risk intervention** that aligns with broader **healthcare stewardship and cost-containment goals**.	
**Certainty of evidence of required resources**<br>What is the certainty of the evidence of resource requirements (costs)?		
**Judgement**	**Research evidence**	**Additional considerations**
**Very low:** PICO 4: Should critically ill adults whose UGIB risk factors are no longer present continue or discontinue SUP?<br>PICO 5: Should critically ill adults without UGIB risk factors but receiving SUP before ICU admission continue or discontinue SUP?<br>**Low:**<br>**Moderate:**	See above.	**Panel comment**<br>Two panel members chose "low."<br><br>More arguments are needed for "very low"; cohort studies are initially deemed "low" until downgraded for other factors

**High:**<br>**No included studies:**		

**Cost effectiveness**<br>Does the cost-effectiveness of the intervention favor the intervention or the comparison?		
**Judgement**	**Research evidence**	**Additional considerations**
**Favors the comparison:**<br>**Probably favors the comparison:** PICO 4: Should critically ill adults whose UGIB risk factors are no longer present continue or discontinue SUP?<br>PICO 5: Should critically ill adults without UGIB risk factors but receiving SUP before ICU admission continue or discontinue SUP?<br>**Does not favor either the intervention or the comparison:**<br>**Probably favors the intervention:**	**Continuation Without Indication Is Not Cost-Effective (10, 8)**<br>• **Shin et al. (2015):**<br>• Most continued outpatient PPI prescriptions had **no supporting GI diagnosis (no ICD-10 code)**, implying **no clinical justification**.<br>• The total cost of inappropriate PPI use in a single institution over 4 years was **$40,175**, including **$12,053 in out-of-pocket patient expenses**—representing **clear economic waste** with **no added benefit**.<br>• **Wong (2025):**<br>• Highlights significant costs associated with adverse drug events (ADEs) linked to SUP, including **VAP, CDI, and AKI.**<br>• These conditions require costly interventions, longer hospitalizations, and contribute to **increased healthcare expenditure**.<br>**Discontinuation Is Cost-Effective**<br>• **Discontinuation of SUP after risk factors resolve**:<br>• Is **not associated with increased UGIB risk**, as confirmed in multiple studies.<br>• Avoids both **direct drug costs** and **indirect costs** from preventable complications.<br>• Reduces resource utilization (e.g., pharmacist workload, monitoring, follow-up testing).<br>• Represents a **high-value intervention** with minimal risk and strong economic justification.<br>**Conclusion:**	

**Favors the intervention:**<br>**Varies:**<br>**No included studies:**	• Continued SUP use without indication is **cost-ineffective**, exposing both patients and health systems to **unnecessary costs and avoidable harm**.<br>• **Discontinuation**, when clinically appropriate, yields a **more favorable cost-effectiveness profile**, making it the economically and clinically responsible choice (probably favors the comparison).	
**Equity**<br>What would be the impact on health equity?		
**Judgement**	**Research evidence**	**Additional considerations**
**Reduced:**<br>**Probably reduced:**<br>**Probably no impact:**<br>**Probably increased:**<br>PICO 4: Should critically ill adults whose UGIB risk factors are no longer present continue or discontinue SUP?<br>PICO 5: Should critically ill adults without UGIB risk factors but receiving SUP before ICU admission continue or discontinue SUP?<br>**Increased:**<br>**Varies:**<br>**Don't know:**	**How Inappropriate SUP Use Affects Equity (11)**<br>• **Overuse of SUP** disproportionately affects:<br>• Patients in **resource-limited ICUs**<br>• Those with **fragmented care** or poor **medication reconciliation**<br>• Populations in **under-resourced or rural settings**<br>• **Choi et al. (2019)** noted that curbing unnecessary drug use may help prevent harm in settings where **systemic resources are already stretched**, enhancing patient safety and reducing inequity.<br>• **Li et al. (2025)** demonstrated the successful use of **pharmacist-led deprescribing strategies** across **diverse hospital types** (rural and urban ICUs) in China, showing that equitable implementation is feasible and effective.<br>• **Krag et al. (2015)** highlighted **international variation** in access to SUP guidelines and deprescribing protocols, suggesting that **structured interventions could reduce inequities** stemming from inconsistent practices.<br>• **Farley (2013)** and **Domond (2017)** both pointed out that continuation of SUP often depends more on **provider habits and institutional norms** than on actual patient risk, leading to **unwarranted practice variation**.<br>• Standardizing deprescribing practices would help **ensure consistent, evidence-based care**, regardless of provider or setting—particularly benefiting populations at risk for receiving **lower-quality or less individualized care**.<br>**Conclusion:**	Equity-focused guidelines should consider how continued SUP disproportionately affects vulnerable patients in the ICU (e.g., older adults, polypharmacy, limited follow-up access).<br><br>Ensure input from geriatrics, pharmacists, and patients recovering from ICU care to shape meaningful, equitable deprescribing policies.<br><br>Using structured tools (e.g., **GIN-McMaster Equity Checklist Extension**) to **evaluate and track equity impacts** over time.

	• Reducing inappropriate SUP use can **enhance medication safety**, particularly for underserved populations.<br>• **Structured deprescribing interventions** promote **equity** by standardizing care and reducing avoidable complications that disproportionately impact vulnerable groups.<br>• The evidence supports that **discontinuation efforts are likely to reduce disparities**, making this an equity-enhancing practice.	**Panel comment**<br>Probably improves equity by reducing disparities.
**Acceptability**<br>Is the intervention acceptable to key interest-holders?		
**Judgement**	**Research evidence**	**Additional considerations**
**No:**<br>**Probably no:**<br>**Probably yes:** PICO 4: Should critically ill adults whose UGIB risk factors are no longer present continue or discontinue SUP?<br>PICO 5: Should critically ill adults without UGIB risk factors but receiving SUP before ICU admission continue or discontinue SUP?<br>**Yes:**<br>**Varies:**<br>**Don't know:**	**Clinician Acceptability of Deprescribing** (7, 6, 1, 5, 4, 9)<br>• **Choi et al. (2019):** Deprescribing strategies were **well-accepted** in real-world hospital settings, particularly when risk data were shared with clinical teams.<br>• **Palmowski (2024)** found that clinicians are more likely to accept deprescribing when **evidence is clearly presented**, supporting structured decision-making.<br>• **Li et al. (2025)** reported that **over 80%** of pharmacist-led deprescribing recommendations were accepted by prescribers—demonstrating **strong stakeholder buy-in**.<br>• **Domond (2017)** did not report direct acceptability data but reinforces that when deprescribing is **guided (e.g., via pharmacist input)**, acceptance is high.<br>**Barriers and Facilitators**<br>• **Bardou (2015)** identified **practice inertia** as a barrier—clinicians may continue SUP by habit or due to outdated risk perceptions.<br>• **All studies** acknowledge that SUP continuation is often **automated or embedded** in practice, suggesting that **behavioral defaults** may drive overuse.<br>• However, no studies report significant resistance to deprescribing when supported by: **Electronic Health Record (EHR) prompts, Clinical guidelines, or Pharmacist recommendations.**<br>• **Buendgens (2016)** emphasized the importance of a **culture of medication review**, which supports deprescribing as a clinical norm.<br>• **Krag et al. (2015)** indicated that while 64% of ICUs had SUP guidelines, **variation in uptake** suggests room to improve acceptability via institutional policies, protocols, and training.<br>**Conclusion:**<br>• Deprescribing SUP is **likely acceptable** to clinicians and institutions, especially when it is **structured, evidence-based, and integrated into clinical workflows (e.g., via pharmacist input or EHR flags)**<br>• Resistance is low when support tools are in place, and most stakeholders appear **receptive to change** when presented with clear rationale and systems support.	

**Feasibility**<br>Is the intervention feasible to implement?		
**Judgement**	**Research evidence**	**Additional considerations**
**No:**<br>**Probably no:**<br>**Probably yes:**<br>**Yes:** PICO 4: Should critically ill adults whose UGIB risk factors are no longer present continue or discontinue SUP?<br>PICO 5: Should critically ill adults without UGIB risk factors but receiving SUP before ICU admission continue or discontinue SUP?<br>**Varies:**<br>**Don't know:**	**Summary of evidence:** (1, 9, 10, 4, 2)<br>• **Choi et al. (2019)** demonstrated successful implementation of deprescribing through a **multidisciplinary team model**, integrating deprescribing into routine care.<br>• **Li et al. (2025)** successfully implemented a **multifaceted intervention** (manuals, checklists, pharmacist review) across **26 ICUs during the COVID-19 pandemic**, showing that even under operational strain, deprescribing is **realistically achievable**.<br>• **Shin (2015)** highlighted **pharmacist-led medication reconciliation** as an effective strategy post-ICU to identify and discontinue inappropriate SUP.<br>• **Palmowski (2024)** supported use of **risk stratification during transitions of care**, particularly from ICU to ward and discharge planning.<br>• **Buendgens et al. (2016)** and **Bardou (2015)** called for a stewardship model with **simple algorithms** and **daily review tools** to support decision-making.<br>• **Farley (2013)** recommended integrating **automatic stop orders** at ICU discharge, similar to approaches used in **antibiotic stewardship**.<br>• **Real-time clinical decision support**, such as **EHR prompts** to reassess SUP, has been used effectively in multiple settings to drive deprescribing.<br>• **Domond (2017)** demonstrated that **structured clinical pathways and data collection** tools can be applied to deprescribing initiatives.<br>**Barriers and Dependencies**<br>• **Bardou (2015)** acknowledged that **clinical habit and lack of awareness** remain primary barriers to deprescribing SUP.<br>• **Krag et al. (2015)** noted that while many ICUs used SUP, some lacked formal guidelines or triggers for deprescribing—indicating that feasibility is enhanced with **protocol development and institutional support**.<br>**Deprescribing SUP is feasible, especially when supported by: Pharmacist integration, Clinical checklists or order sets, EHR-based decision support tools, or Standardized protocols across transitions of care.**<br>• Though variability in infrastructure exists, **most barriers are modifiable**, and practical tools already exist to support widespread implementation.	**Panel comment**<br>While we have the necessary infrastructure that favors successful implementation, variability in clinical practice and barriers such as clinical inertia introduce some uncertainty. Therefore, I lean toward "Probably yes" for feasibility, pending additional local data to confirm implementation outcomes.

Summary of judgements

	PICO 4: Should critically ill adults whose UGIB risk factors are no longer present continue or discontinue SUP?	PICO 5: Should critically ill adults without UGIB risk factors but receiving SUP before ICU admission continue or discontinue SUP?
**Problem**	**Yes**	
**Desirable Effects**	Don’t know	Don’t know
**Undesirable Effects**	Varies	Varies
**Net balance**	Moderate net undesirable	Moderate net undesirable
**Certainty of evidence**	Very low	Very low
**Values**	Possibly important uncertainty or variability	Possibly important uncertainty or variability
**Balance of effects**	Probably favors the comparison	Probably favors the comparison
**Resources required**	Large savings	Large savings
**Certainty of evidence of required resources**	Very low	Very low
**Cost effectiveness**	Probably favors the comparison	Probably favors the comparison
**Equity**	Probably increased	Probably increased
**Acceptability**	Probably yes	Probably yes
**Feasibility**	Yes	Yes

Conclusions

Recommendation(s)

Strength of recommendation
Conditional recommendation against the intervention
Certainty Very Low
In critically ill adults with resolved risk factors for UGIB, **the panel suggests against the continuation of stress ulcer prophylaxis (SUP) in patients in the ICU when risk factors for upper gastrointestinal bleeding are no longer present.**
In other words, we suggest **discontinuing stress ulcer prophylaxis (SUP)** in patients in the ICU **when risk factors for upper gastrointestinal bleeding are no longer present**.

Strength of recommendation
Conditional recommendation against the intervention
Certainty Very Low
In critically ill adults without UGIB risk factors but receiving SUP prior to ICU admission, **the panel suggests against the continuation of stress ulcer prophylaxis (SUP) without an active indication.**
In other words, we suggest **discontinuing stress ulcer prophylaxis (SUP)** in patients in the ICU **who are receiving SUP without an identifiable indication at admission.**

Remark

- In **patients already on PPIs or H2 blockers prior to ICU admission**, the **original indication must be reviewed**. Discontinuation should be considered **only if no longer indicated**.
- Common indications requiring continued SUP may include recent UGIB, erosive esophagitis, *H. pylori* therapy, or hypersecretory conditions. In cases of anaphylaxis or angioedema, maintenance therapy with H2RAs may still be necessary.
- In situations where indication is unclear (e.g., comatose patient without records), clinicians should **exercise caution** and avoid automatic discontinuation. Instead, a **consultation with the prescribing physician** or a **review of outpatient history** is advisable.
- These considerations are particularly important in patients in the ICU where intensivists may have **limited knowledge of the patient's long-term GI history**.

Justification

Justification: Discontinuing SUP in low-risk patients avoids known harms (e.g., infections, renal injury) without increasing UGIB risk. While certainty is very low due to limitations of existing studies, the **direction and consistency of evidence strongly favor discontinuation.** Real-world and RCT data (e.g., *Li et al. 2025*) confirm safety and feasibility.

Subgroup Considerations

- In critically ill adults **without risk factors** for developing clinically important stress-related UGIB but who are already on a SUP agent prior to ICU admission, **the original indications for these medications should be reviewed and consideration given to discontinuing them** if no longer needed.

Panel comment: If applicable, discuss with the prescribing physician.

Multiple Chronic Conditions and Polypharmacy

Oen panel member commented: *Multiple Chronic Conditions and Polypharmacy* should be considered especially if the patient had previous GIB and has continued on antiplatelets or anticoagulants.

Clinical Considerations

- Clinical protocols often lack clarity on **timing relative to resolution of UGIB risk factors** (e.g., after extubation, off vasopressors, initiation of enteral feeding).
- Research is needed to define **safe and standardized time points** for deprescribing SUP during ICU or post-ICU transitions.
- Studies should validate **risk assessment tools or checklists** that accurately classify patients as eligible for SUP cessation.

Research Priorities

- Define optimal timing and clinical criteria for discontinuing SUP at key transition points (ICU to ward, ward to discharge, post-discharge).
- Evaluate patient outcomes (UGIB, overt bleeding, ICU LOS, mortality, readmissions rate) associated with deprescribing versus continuation in post-ICU settings.
- Study the safety and effectiveness of deprescribing in high-risk subgroups (e.g., older adults, immunosuppressed, cirrhotic patients).
- Investigate the appropriateness of continuing SUP that was initiated in the outpatient setting, especially when indications are unclear.
- Stratify patients with pre-hospital SUP use based on justified vs. unjustified indications to guide tailored deprescribing approaches.
- Assess the impact of pharmacist-led medication reconciliation and ICU-to-ward discharge tools on SUP discontinuation.
- Examine the use of EHR-based alerts, embedding deprescribing prompts, stop orders, and checklists to prompt deprescribing at discharge or transition points.
- Use AI and clinical decision support systems to detect inappropriate SUP continuation and automate deprescribing recommendations.
- Explore the feasibility and safety of having SUP stewardship protocols to explicit SUP “stop dates,” similar to antimicrobial stewardship protocols.
- Incorporate patient-centered outcomes into future trials to assess acceptability, satisfaction, and quality of life related to SUP deprescribing.

Supplemental content 2.29 Research Priorities for Future SUP Studies in Critically Ill Adults

Research Area	Priority Statement	Rationale
Neurocritical Care	More research is needed to evaluate the impact of SUP in neurocritical care populations and to identify differential effects across neurocritical subgroups.	Evidence for SUP in neurologically injured patients remains sparse and may differ due to altered gastrointestinal perfusion, varying bleeding risk, and differing management strategies.
Non-Invasive Ventilation	Future studies should assess the benefits and harms of SUP in critically ill patients receiving non-invasive mechanical ventilation.	Current evidence predominantly focuses on invasively ventilated patients; non-invasive ventilation presents unique considerations for aspiration and bleeding risk.
Patient Values and Preferences	Qualitative and quantitative studies are needed to assess how patients prioritize bleeding prevention versus potential adverse effects of SUP.	Informed, preference-sensitive decisions depend on understanding patient tradeoffs, particularly for outcomes like infection, mortality, and quality of life.
Microbiome and Patient-Reported Outcomes	Investigate patient-reported outcomes and gut microbiome alterations as mediators of SUP-related infection risk.	Understanding these mechanisms may help identify subgroups at heightened infection risk or differential benefit.
Cardiovascular Risk	Research should explore the association between PPI use and adverse cardiovascular events (e.g., myocardial infarction, stroke), especially in patients receiving antiplatelet therapy.	Concerns about cardiovascular safety have been raised, but evidence remains inconsistent and largely observational.
Machine Learning Applications	Explore the use of machine learning models for risk stratification to guide SUP use and personalize prophylaxis strategies.	Predictive models may identify patients most likely to benefit while avoiding unnecessary prophylaxis in low-risk populations. Equity implications must be considered when applying ML in diverse resource settings.

Research Area	Priority Statement	Rationale
Severity of Illness and SUP Efficacy	Clarify the relationship between illness severity and the efficacy or harm of SUP.	Subgroup analyses suggest SUP may be more beneficial in moderately ill patients and potentially harmful in those with extreme severity; further validation is needed.
PICO 2		
Baseline EN Stratification	Conduct RCTs that stratify patients by enteral nutrition (EN) status **at baseline**, not post hoc.	Most existing studies assessed EN status post-randomization, leading to confounding and unreliable subgroup inferences. Accurate baseline stratification is essential for valid comparisons.
SUP vs. EN-Only Comparisons	Directly compare pharmacologic SUP versus **EN alone** in high-risk and low-risk patients in the ICU.	The interaction between EN and SUP remains unclear; EN may provide mucosal protection that diminishes the need for pharmacologic prophylaxis, especially in low-risk individuals.
Feeding Dose and Continuity	Evaluate the effect of **dose, timing, and interruptions** of EN on UGIB incidence.	EN delivery is frequently interrupted in ICU settings, which may compromise its protective effect against stress-related mucosal injury.
SUP Agent Comparison	Compare **H2 receptor antagonists (H2RAs)** and **proton pump inhibitors (PPIs)** in EN-fed patients.	Existing data are pooled across agents; differences in efficacy and safety profiles between H2RAs and PPIs may be magnified in the context of EN.
Mechanistic Studies	Conduct physiological studies on how EN modifies gastric mucosal defense and acid suppression pathways.	Understanding the biological interplay between EN and mucosal injury may clarify the mechanism by which EN modifies UGIB risk and SUP benefit.
Patient and Surrogate Preferences	Use conjoint analysis or discrete choice experiments to evaluate preferences regarding **UGIB vs. infection risks**.	Critically ill patients cannot express preferences, and surrogate decision-making often lacks clarity on trade-offs between bleeding and pneumonia risks.
Economic Evaluations	Perform **cost-effectiveness analyses** comparing SUP vs. no SUP in EN-fed patients in the ICU, incorporating infection-related costs.	SUP medications are inexpensive, but complications like VAP and CDI incur substantial costs. A full economic picture is necessary to inform institutional protocols.
PICO 3		
Comparative Effectiveness Trials	Conduct high-quality RCTs comparing PPIs and H2RAs in critically ill adults, stratified by baseline bleeding risk and illness severity.	Existing evidence is limited by indirectness and subgroup imbalances. Some signals suggest differential mortality effects in high-acuity patients receiving PPIs.

Research Area	Priority Statement	Rationale
Route of Administration	Evaluate the comparative effectiveness and pharmacokinetics of enteral versus intravenous SUP administration in ICU settings.	Current data rely on indirect comparisons and may be confounded by severity of illness; definitive evidence is lacking to guide route selection.
Formulation-Level Comparisons	Compare specific enteral PPI formulations (e.g., omeprazole vs. pantoprazole) in terms of safety, absorption via feeding tubes, and cost-effectiveness.	Formulation-specific considerations are relevant for critically ill patients receiving medications via enteral tubes, yet evidence is sparse.
Health Economics	Conduct cost-effectiveness analyses across diverse healthcare systems, including resource-limited settings.	Existing studies are based in high-income countries and may not generalize globally; economic and logistical feasibility must be locally contextualized.
Clinical Decision Tools	Develop and validate clinical prediction models or AI-based tools to guide SUP initiation based on individualized bleeding and complication risk.	SUP use remains poorly targeted. Decision support tools may improve precision and reduce unnecessary use.
De-prescribing and Stewardship	Investigate SUP discontinuation strategies and their impact on long-term outcomes post-ICU discharge.	SUP is often continued inappropriately after ICU stay, increasing risks and healthcare costs. De-prescribing protocols remain underexplored.
**PICOs 4 and 5**		
Timing of Discontinuation	Determine optimal timing and clinical milestones for SUP discontinuation during and after ICU stay.	Clinical protocols lack consensus on when SUP should be stopped (e.g., post-extubation, enteral feeding initiation). Standardized timepoints could improve safety and consistency in deprescribing practices.
Risk Stratification	Develop and validate tools/checklists to identify patients eligible for SUP cessation.	Many SUP decisions are made without structured assessments. Tools to evaluate bleeding risk post-ICU could prevent inappropriate continuation and support safe, tailored deprescribing.
Outcomes of Deprescribing	Evaluate patient outcomes (e.g., UGIB, ICU LOS, readmission, mortality) after SUP discontinuation.	There is a need for prospective data to confirm that discontinuation does not increase GI bleeding risk or other adverse outcomes in low-risk ICU populations.
Pre-hospital SUP Continuation	Investigate appropriateness of continuing SUP initiated prior to ICU admission.	Many patients enter the ICU already on SUP without documentation of indication. Research is needed to guide when and how to discontinue legacy SUP use safely.

Research Area	Priority Statement	Rationale
Implementation Strategies	Study the effectiveness of EHR-based alerts, pharmacist-led reviews, and stop orders in prompting deprescribing.	Structured interventions have shown promise, but comparative effectiveness studies are needed to inform scalable models across different health systems and resource settings.
High-Risk Subgroups	Examine deprescribing safety in specific populations (e.g., older adults, cirrhotics, immunosuppressed).	These groups may have elevated UGIB risk or unique vulnerabilities. Data from generalized ICU populations may not apply, necessitating targeted research to ensure safety.
Post-Discharge Continuation	Evaluate long-term outcomes and adverse events associated with SUP continuation beyond hospital discharge.	A significant proportion of SUP continuation extends into outpatient care, often without indication. Longitudinal studies can quantify downstream harms (e.g., AKI, infection, malabsorption).
Decision Support and AI	Explore use of AI and advanced clinical decision support tools to identify inappropriate SUP use and recommend deprescribing.	Automation can improve detection and reduce reliance on manual review, particularly in busy ICU environments. AI tools could flag candidates for SUP cessation based on dynamic clinical data.
Patient-Centered Outcomes	Incorporate patient-reported outcomes (e.g., satisfaction, medication burden, quality of life) in deprescribing trials.	Most studies have focused on prescribing patterns or clinical events. Including patient perspectives will improve shared decision-making and guideline alignment with post-ICU recovery goals.
Equity and Access	Assess how deprescribing protocols impact diverse populations and whether they reduce disparities in SUP continuation.	Inappropriate SUP use may disproportionately affect vulnerable patients. Equity-focused research can identify gaps in implementation and inform interventions that promote fair, evidence-based deprescribing across settings.

References for supplementary appendix

[1] GIN Guideline Collaborations Working Group. Available at: https://macgrade.mcmaster.ca/resources/gin-guideline-collaborations-toolkit-hub/.

[2] Bachmann KF, Jenkins B, Asrani V, et al. Core outcome set of daily monitoring of gastrointestinal function in adult critically ill patients: a modified Delphi consensus process (COSMOGI). Crit Care. 2024;28(1):420.

[3] Handbook for grading the quality of evidence and the strength of recommendations using the GRADE approach. *Available at*: https://gdt.gradepro.org/app/handbook/handbook.html#h.svwngs6pm0f2 Accessed April 11, 2023.

[4] Murad MH, Chu H, Lin L, Wang Z. The effect of publication bias magnitude and direction on the certainty in evidence. *BMJ Evid Based Med*. 2018;23(3):84-86.

[5] Lin L, Chu H, Murad MH, et al. Empirical comparison of publication bias tests in meta-analysis. *J Gen Intern Med*. 2018;33(8):1260 1267.

[6] Cuello-Garcia CA, Santesso N, Morgan RL, et al. GRADE guidance 24: Optimizing the integration of randomized and non-randomized studies of interventions in evidence syntheses and health guidelines. *J Clin Epidemiol*. 2022;142:200-208. doi: 10.1016/j.jclinepi.2021.11.026.

[7] World Health Organization. *Strengthening countries' capacities to adopt and adapt evidence-based guidelines: a handbook for guideline contextualization*. WHO Regional Office for Europe; 2023.

[8] Ye Z, Reintam Blaser A, Lytvyn L, et al. Gastrointestinal bleeding prophylaxis for critically ill patients: a clinical practice guideline. *BMJ*. 2020;368:l6722. doi: 10.1136/bmj.l6722.

[9] Krag M, Marker S, Perner A, et al. Pantoprazole in patients at risk for gastrointestinal bleeding in the ICU. *N Engl J Med*. 2018;379(23):2199-2208

[10] Cook D, Deane A, Lauzier F et al. Stress ulcer prophylaxis during invasive mechanical ventilation. *N Engl J Med*. 2024;391(1):9-20. doi: 10.1056/NEJMoa2404245

[11] Granholm A, Zeng L, Dionne JC, et al. Predictors of gastrointestinal bleeding in adult ICU patients: a systematic review and meta-analysis. *Intensive Care Med*. 2019;45(10):1347-1359

[12] Ali D, Barra ME, Blunck J, et al. Stress-related upper gastrointestinal bleeding in patients with aneurysmal subarachnoid hemorrhage: a multicenter observational study. *Neurocrit Care*. 2021;35(1):39-45.

[13] Liu B, Liu S, Yin A, et al. Risks and benefits of stress ulcer prophylaxis in adult neurocritical care patients: a systematic review and meta-analysis of randomized controlled trials. *Crit Care*. 2015;19:409.

[14] Daou M, Dionne JC, Teng JFT, et al. Prophylactic acid suppressants in patients with primary neurologic injury: a systematic review and meta-analysis of randomized controlled trials. *J Crit Care*. 2022;71:154093

[15] Shin JS, Abah U. Is routine stress ulcer prophylaxis of benefit for patients undergoing cardiac surgery? *Interact Cardiovasc Thorac Surg*. 2012;14(5):622-628. doi: 10.1093/icvts/ivs019.

Systematic reviews for PICO 1

(1) Wang Y, Ye Z, Ge L, et al. Efficacy and safety of gastrointestinal bleeding prophylaxis in critically ill patients: systematic review and network meta-analysis. *BMJ*. 2020;368:l6744. doi: 10.1136/bmj.l6744.
(2) Wang Y, Parpia S, Ge L, et al. Proton-Pump Inhibitors to Prevent Gastrointestinal Bleeding - An Updated Meta-Analysis. *NEJM Evid.* 2024;3(7):EVIDoa2400134. doi:10.1056/EVIDoa2400134.
(3) Wang Y et al., Efficacy and safety of gastrointestinal bleeding prophylaxis in critically ill patients: an updated systematic review and network meta-analysis of randomized trials. *Intensive Care Med.* 2020;46(11):1987-2000. doi: 10.1007/s00134-020-06209-w.
(4) Granholm A, Zeng L, Dionne JC, et al. Predictors of gastrointestinal bleeding in adult ICU patients: a systematic review and meta-analysis. *Intensive Care Med*. 2019;45(10):1347-1359. doi: 10.1007/s00134-019-05751-6.

References for PICO 1 evidence profile

1. Granholm A, Krag M, Marker S, Alhazzani W, Perner A, Møller MH. Predictors of gastrointestinal bleeding in adult ICU patients in the SUP-ICU trial. *Acta Anaesthesiol Scand*. 2021;65(6):792-800. doi:10.1111/aas.13805.
2. Wang Y, Ye Z, Ge L, et al. Efficacy and safety of gastrointestinal bleeding prophylaxis in critically ill patients: systematic review and network meta-analysis. *BMJ*. 2020;368:l6744. doi:10.1136/bmj.l6744.
3. Wang Y, Parpia S, Ge L, et al. Proton-pump inhibitors to prevent gastrointestinal bleeding: an updated meta-analysis. *NEJM Evid*. 2024;3(7):EVIDoa2400134. doi:10.1056/EVIDoa2400134.
4. Granholm A, Zeng L, Dionne JC, et al. Predictors of gastrointestinal bleeding in adult ICU patients: a systematic review and meta-analysis. *Intensive Care Med*. 2019;45(10):1347-1359. doi:10.1007/s00134-019-05751-6.
5. Wang Y, Ge L, Ye Z, et al. Efficacy and safety of gastrointestinal bleeding prophylaxis in critically ill patients: an updated systematic review and network meta-analysis of randomized trials. *Intensive Care Med*. 2020;46(11):1987-2000. doi:10.1007/s00134-020-06209-w.
6. MacLaren R, Dionne JC, Granholm A, et al. Society of Critical Care Medicine and American Society of Health System Pharmacists guideline for the prevention of stress-related gastrointestinal bleeding in critically ill adults. *Crit Care Med*. 2024;52(8):e421-e430. doi:10.1097/CCM.0000000000006330.

Reference for PICO 1 EtD

1. Krag M, Alhazzani W, Møller MH. Prevention of upper gastrointestinal bleeding in critical illness. *Intensive Care Med*. 2023;49(3):334-336. doi: 10.1007/s00134-022-06959-9.
2. Young PJ, Cook DJ, Deane AM. Preventing stress ulcer bleeding. *Intensive Care Med*. 2024;50(12):2162-2165. doi: 10.1007/s00134-024-07674-3.
3. Halling CMB, Møller MH, Marker S, et al. The effects of pantoprazole vs. placebo on 1-year outcomes, resource use and employment status in ICU patients at risk for gastrointestinal bleeding: a secondary analysis of the SUP-ICU trial. Intensive Care Med. 2022;48(4):426-434. doi: 10.1007/s00134-022-06631-2.
4. Madsen KR, Lorentzen K, Clausen N, et al. Guideline for stress ulcer prophylaxis in the intensive care unit. *Danish Med Bull*. 2014;61(3):C4811.
5. Ye Z, Reintam Blaser A, Lytvyn L, et al. Gastrointestinal bleeding prophylaxis for critically ill patients: a clinical practice guideline. *BMJ* 2020;368:l6722. doi: 10.1136/bmj.l6722.
6. Granholm A, Zeng L, Dionne JC, et al. Predictors of gastrointestinal bleeding in adult ICU patients: a systematic review and meta-analysis. *Intensive Care Med*. 2019;45:1347-1359. doi:10.1007/s00134-019-05751-6.
7. Krag M, Marker S, Perner A, et al. Pantoprazole in patients at risk for gastrointestinal bleeding in the ICU. *N Engl J Med*. 2018;379(23):2199-2208. doi:10.1056/NEJMoa1714919.
8. Cook D, Deane A, Lauzier F, et al. Stress ulcer prophylaxis during invasive mechanical ventilation. *N Engl J Med*. 2024;391(1):9-20. doi:10.1056/NEJMoa2404245.
9. MacLaren R, Dionne JC, Granholm A, et al. Society of Critical Care Medicine and American Society of Health-System Pharmacists guideline for the prevention of stress-related gastrointestinal bleeding in critically ill adults. *Crit Care Med*. 2024;52:e421-e430. doi:10.1097/CCM.0000000000006330.
10. Krag M, Perner A, Wetterslev J, et al. Stress ulcer prophylaxis in adult neurocritical care patients—no firm evidence for benefit or harm. *Crit Care* 2016;20:22. doi:10.1186/s13054-016-1188-6.

Systematic reviews evaluated for PICO 2

(1) Huang H-B, Jiang W, Wang C-Y, et al. Stress ulcer prophylaxis in intensive care unit patients receiving enteral nutrition: a systematic review and meta-analysis. *Crit Care*. 2018;22(1):20. doi:10.1186/s13054-017-1937-1.
(2) Reynolds PM, MacLaren R. Re-evaluating the Utility of Stress Ulcer Prophylaxis in the Critically Ill Patient: A Clinical Scenario-Based Meta-Analysis. *Pharmacotherapy*. 2019;39(3):408-420. doi: 10.1002/phar.2172.

Systematic reviews evaluated for PICO 3

(1) Wang Y, Ye Z, Ge L, et al. *Efficacy and safety of gastrointestinal bleeding prophylaxis in critically ill patients: systematic review and network meta-analysis*. BMJ. 2020;368:l6744. doi: 10.1136/bmj.l6744.
(2) Wang Y, Ge L, Ye Z, et al. Efficacy and safety of gastrointestinal bleeding prophylaxis in critically ill patients: an updated systematic review and network meta-analysis of randomized trials. *Intensive Care Med*. 2020;46:1987-2000. doi: 10.1007/s00134-020-06209-w.

Reference for PICO 3 EtD

1. Wang Y, Parpia S, Ge L, et al. Proton-pump inhibitors to prevent gastrointestinal bleeding: an updated meta-analysis. *NEJM Evid*. 2024;3(7):EVIDoa2400134. doi: 10.1056/EVIDoa2400134.
2. MacLaren R, Dionne JC, Granholm A, et al. Society of Critical Care Medicine and American Society of Health-System Pharmacists Guideline for the Prevention of Stress-Related Gastrointestinal Bleeding in Critically Ill Adults. *Crit Care Med.* 2024;52:e421-430. doi: 10.1097/CCM.0000000000006330.
3. Ye Z, Blaser AR, Lytvyn L, et al. Gastrointestinal bleeding prophylaxis for critically ill patients: a clinical practice guideline. *BMJ.* 2020:368:l6722. doi: 10.1136/bmj.l6722.
4. Wang Y, Ge L, Ye Z, et al. Efficacy and safety of gastrointestinal bleeding prophylaxis in critically ill patients: an updated systematic review and network meta-analysis of randomized trials. *Intensive Care Med*. 2020;46:1987-2000. doi: 10.1007/s00134-020-06209-w.
5. Halling CMB, Møller MH, Marker S, et al. The effects of pantoprazole vs. placebo on 1-year outcomes, resource use and employment status in ICU patients at risk for gastrointestinal bleeding: a secondary analysis of the SUP-ICU trial. Int Care Med. 2022;48:426-434. doi: 10.1007/s00134-022-06631-2.
6. Toews I, Hussain S, Nyirenda JLZ, et al. Pharmacological interventions for preventing upper gastrointestinal bleeding in people admitted to intensive care units: a network meta-analysis. *BMJ Evidence-Based Med*. 2024;30:22-35.
7. Jeong E, Shendre A, Su Y, et al. Association of omeprazole-related myopathy with drug–drug and drug–gene interactions involving CYP2C19 and CYP3A4: A nested case–control study. *Pharmacotherapy.* 2025;45(10):654-666.

Studies and reference evaluated for PICOs 4 and 5

1. Choi YJ, Sim J, Jung YT, Shin S. Impact of a multidisciplinary quality improvement initiative to reduce inappropriate usage of stress ulcer prophylaxis in hospitalized patients. *Br J Clin Pharmacol*. 2020;86(5):903-912. doi: 10.1111/bcp.14197.
2. Farley KJ, Barned KL, Crozier TM. Inappropriate continuation of stress ulcer prophylaxis beyond the intensive care setting. *Crit Care Resusc*. 2013;15(2):147-151. doi: 10.1016/S1441-2772(23)01783-0.

3. Farrell CP, Mercogliano G, Kuntz CL. Overuse of stress ulcer prophylaxis in the critical care setting and beyond. *J Crit Care*. 2010;25(2):214-220. doi: 10.1016/j.jcrc.2009.05.014.
4. Palmowski L, Von Busch A, Unterberg M, et al. Timely cessation of proton pump inhibitors in critically ill patients impacts morbidity and mortality: A propensity score-matched cohort study. *Crit Care Med*. 2024;52(2):190-199. doi: 10.1097/CCM.0000000000006104.
5. Krag M, Perner A, Wetterslev J, et al. Stress ulcer prophylaxis in the intensive care unit: an international survey of 97 units in 11 countries. *Acta Anaesthesiol Scand.* 2015;59(5):576-585. doi:10.1111/aas.12508.
6. Buendgens L. Prevention of stress-related ulcer bleeding at the intensive care unit: risks and benefits of stress ulcer prophylaxis prophylaxis. *World J Crit Care Med*. 2016;5(1):57-64. doi: 10.5492/wjccm.v5.i1.57.
7. Bardou M, Quenot J-P, Barkun A. Stress-related mucosal disease in the critically ill patient. *Nat Rev Gastroenterol Hepatol*. 2015;12(2):98-107. doi: 10.1038/nrgastro.2014.235.
8. Wong A, Kane-Gill SL, Barletta JF. Pharmacological prevention of gastrointestinal bleeding in critically Ill patients. *Curr Opin Crit Care*. 2025;31(2):204-211. doi: 10.1097/MCC.0000000000001251.
9. Li H, Zeng L, Xu P, et al. Effectiveness of a pharmacist-led intervention to reduce acid suppression therapy for stress ulcer prophylaxis in ICUs in china: a multicenter, stepped-wedge, cluster-randomized controlled trial. *Crit Care Med*. 2025;53(4):e805-e816. doi: 10.1097/CCM.0000000000006589.
10. Shin S. Evaluation of costs accrued through inadvertent continuation of hospital-initiated proton pump inhibitor therapy for stress ulcer prophylaxis beyond hospital discharge: a retrospective chart review. *Ther Clin Risk Manag.* 2015;11:649-657. doi: 10.2147/TCRM.S81759.
11. Dewidar O, Pardo JP, Peña-Rosas JP, et al. Advancing health equity: Why guideline development must prioritize fairness and justice. 2025. doi: 10.1002/gin2.70015.
