# Supplementary material for: Recommendations for Stress Ulcer Prophylaxis in Critically Ill Adults: A Contextualized Clinical Practice Guideline From the Saudi Critical Care Society and the Scandinavian Society of Anaesthesiology and Intensive Care Medicine, Endorsed by the Kuwait Anesthesia and Critical Care Society
Source: Acta Anaesthesiol Scand. 2026 Feb 8;70(3):e70201. doi: 10.1111/aas.70201 (PMC12883282; doi:10.1111/aas.70201)
Supplement: Supplementary file 3 — Data 3 Outcomes and PICO Prioritization. [file AAS-70-0-s002.pdf]

Supplementary Content 3 Outcomes & PICOs Prioritization

Please complete the following Outcomes and PICOs Prioritization

Panel member name ________________________

Outcomes Prioritization

The outcomes have been extracted from our updated searches and the source guideline "SCCM and ASHP Guideline for the Prevention of Stress-Related Gastrointestinal Bleeding in Critically Ill Adults"

Your scoring will help us identify the most important outcomes to include in each question. We are looking for outcomes that are important or critical to patients, clinicians, and other stakeholders for decision-making.

To facilitate the ranking of outcomes according to their importance, please use the scoring guide provided below. Rate them numerically on a 1 to 9 scale, where 7 to 9 indicates 'critical', 4 to 6 means 'important', and 1 to 3 suggests 'of limited importance'. This will help us distinguish between the levels of importance.

Each section of the survey includes an optional free text field for any additional comments or information you wish to provide.

After the survey's closure, we will calculate and share the median ratings for each outcome in the guideline outline. Based on these findings, we aim to narrow down the number of outcomes to a manageable five to seven, focusing on those that are critical and important.

Outcomes Prioritization

	1 Of least importance	2	3	4	5	6	7	8	9 Of most importance
Clinically Important Upper Gastrointestinal Bleeding (UGIB)	○	○	○	○	○	○	○	○	○
Overt UGIB	○	○	○	○	○	○	○	○	○
Pneumonia/Ventilator-Associated Pneumonia	○	○	○	○	○	○	○	○	○
Clostridioides difficile Infection (C. difficile)	○	○	○	○	○	○	○	○	○
Gastric pH Level	○	○	○	○	○	○	○	○	○
Ventilator-Free Days or Duration of Mechanical Ventilation	○	○	○	○	○	○	○	○	○
ICU Length of Stay	○	○	○	○	○	○	○	○	○
Hospital Length of Stay	○	○	○	○	○	○	○	○	○
Renal Failure Requiring Renal Replacement Therapy	○	○	○	○	○	○	○	○	○

Acute Kidney Failure	○	○	○	○	○	○	○	○	○
Diarrhea	○	○	○	○	○	○	○	○	○
Delirium	○	○	○	○	○	○	○	○	○
Thrombocytopenia	○	○	○	○	○	○	○	○	○
Any UGIB	○	○	○	○	○	○	○	○	○
Mortality	○	○	○	○	○	○	○	○	○

OPTIONAL: Please share any additional comments in the space below. We would also appreciate your input on the Minimal Important Difference (MID)-the smallest change in a clinical outcome that is considered meaningful and important by patients, clinicians, or other stakeholders.

____

PICO Questions Review

PICO Questions Review
Please review the following preliminary PICO questions and provide your feedback.

These preliminary PICO questions were selected based on prioritization criteria, focusing on areas where the source recommendations differ in feasibility, acceptability, resource use, impact on equity, or alignment with current practices in Saudi Arabia and Nordic countries. For example, we are assessing questions related to the use of proton pump inhibitors (PPIs) versus histamine-2 receptor antagonists (H2RAs), the timing of SUP discontinuation, and the role of enteral nutrition.

Criteria Used for Prioritization:
Common practice relevance: question commonly arises in clinical practice.
Uncertainty in practice: variability or uncertainty regarding patient management.
New research evidence: new evidence or studies impact existing practices.
Resource use and costs: The question has significant implications for resource use or costs.
Feasibility: the intervention differs in feasibility between the source and local contexts.
Acceptability: the intervention may be more or less acceptable based on cultural or healthcare system differences.
Equity Impact: the intervention has implications for access and equity.
Alignment with current practice: differences exist between current practices and the recommended interventions.

Preliminary PICO #1: In critically ill adults in ICU with coagulopathy, shock, or chronic liver disease, does the use of SUP compared to no SUP reduce the occurrence of clinically important or overt UGIB?

Population: Critically ill adults in ICU with coagulopathy, shock, or chronic liver disease
Intervention: SUP
Comparator: No SUP
Outcome: Reduced occurrence of clinically important stress-related UGIB, Reduced occurrence of overt UGIB

Subgroup:
Neurocritical care adults in ICU with risk factors for developing stress-related UGIB

○ Agree
○ Disagree
○ Other: comment below

Additional Comments for PICO #1: Please provide any additional comments, suggestions, or concerns related to the preliminary PICO 1.

____

Preliminary PICO #2: In critically ill adults with risk factors for developing stress-related UGIB who are enterally fed, does the use of SUP compared to no SUP reduce the occurrence of clinically important UGIB?<br><br>Population: Critically ill adults with risk factors for developing stress-related UGIB who are enterally fed during ICU admission<br>Intervention: SUP<br>Comparator: No SUP<br>Outcome: Reduced occurrence of clinically important stress-related UGIB<br>Subgroup:<br>Critically ill adults who are enterally fed and at low risk for clinically important stress-related UGIB	◯ Agree<br>◯ Disagree<br>◯ Other: comment below
Additional Comments for PICO #2: Please provide any additional comments, suggestions, or concerns related to the preliminary PICO 2 question	__________________________
Preliminary PICO #3: In critically ill adults in the ICU with risk factors for developing stress-related UGIB, does the use of PPIs or H2RAs compared to no PPIs or H2RAs reduce the occurrence of clinically important UGIB?<br><br>Population: Critically ill adults in the ICU with risk factors for developing stress-related UGIB<br>Intervention: PPIs or H2RAs for SUP<br>Comparator: No PPIs or H2RAs for SUP<br>Outcome: Reduced occurrence of clinically important stress-related UGIB<br><br>Subgroup [how do variations in dose or route of administration impact outcomes]<br>Using either enteral or IV routes when administering SUP in critically ill adults with risk factors<br>Low-dose SUP compared with high-dose SUP	◯ Agree<br>◯ Disagree<br>◯ Other: comment below
Additional Comments for PICO #3: Please provide any additional comments, suggestions, or concerns related to the preliminary PICO 3 question	__________________________
PICO #4: In critically ill adults in ICU whose risk factors for stress-related UGIB are no longer present, does discontinuing SUP compared to continuing SUP reduce the occurrence of clinically important UGIB?<br><br>Population: Critically ill adults in ICU with risk factors for developing stress-related UGIB that are no longer present<br>Intervention: Discontinued use of SUP<br>Comparator: Continued use of SUP<br>Outcome: Reduced occurrence of clinically important stress-related UGIB	◯ Agree<br>◯ Disagree<br>◯ Other: comment below
Additional Comments for PICO #4: Please provide any additional comments, suggestions, or concerns related to the preliminary PICO 4 question	__________________________

PICO #5: In critically ill adults without risk factors for stress-related UGIB but who are on SUP prior to ICU admission, does discontinuing SUP compared to continuing SUP reduce the occurrence of clinically important UGIB?<br><br>Population: Critically ill adults who do not have risk factors for developing stress-related UGIB but are on SUP before ICU admission<br>Intervention: Discontinued use of SUP<br>Comparator: Continued use of SUP<br>Outcome: Reduced occurrence of clinically important stress-related UGIB<br><br>Subgroup:<br>Critically ill patients with risk factors for developing stress-related UGIB and are on SUP before ICU admission	◯ Agree<br>◯ Disagree<br>◯ Other: comment below
Additional Comments for PICO #5: Please provide any additional comments, suggestions, or concerns related to the preliminary PICO 5 question	______________________________
